# A classification of endangered high-THC cannabis (Cannabis sativa subsp. indica) domesticates and their wild relatives

**DOI:** 10.3897/phytokeys.144.46700

**Published:** 2020-04-03

**Authors:** John M. McPartland, Ernest Small

**Affiliations:** 1 College of Medicine, University of Vermont, Burlington, Vermont, 05405 USA University of Vermont Burlington United States of America; 2 GW Pharmaceuticals, Sovereign House, Histon, Cambridge, CB24 9BZ, UK GW Pharmaceuticals Cambridge United Kingdom; 3 Science and Technology Branch, Agriculture and Agri-Food Canada, Saunders Building, Central Experimental Farm, Ottawa, Ontario K1A 0C6, Canada Agriculture and Agri-Food Canada Ottawa Canada

**Keywords:** Cannabinoids, *Cannabis sativa*, classification, ecology, germplasm, marijuana, nomenclature

## Abstract

Two kinds of drug-type *Cannabis* gained layman’s terms in the 1980s. “Sativa” had origins in South Asia (India), with early historical dissemination to Southeast Asia, Africa, and the Americas. “Indica” had origins in Central Asia (Afghanistan, Pakistan, Turkestan). We have assigned unambiguous taxonomic names to these varieties, after examining morphological characters in 1100 herbarium specimens, and analyzing phytochemical and genetic data from the literature in a meta-analysis. “Sativa” and “Indica” are recognized as C. sativa subsp. indica var. indica and C. sativa subsp. indica var. afghanica, respectively. Their wild-growing relatives are C. sativa subsp. indica var. himalayensis (in South Asia), and C. sativa subsp. indica var. asperrima (in Central Asia). Natural selection initiated divergence, driven by climatic conditions in South and Central Asia. Subsequent domestication drove further phytochemical divergence. South and Central Asian domesticates can be distinguished by tetrahydrocannabinol and cannabidiol content (THC/CBD ratios, ≥7 or <7, respectively), terpenoid profiles (absence or presence of sesquiterpene alcohols), and a suite of morphological characters. The two domesticates have undergone widespread introgressive hybridization in the past 50 years. This has obliterated differences between hybridized “Sativa” and “Indica” currently available. “Strains” alleged to represent “Sativa” and “Indica” are usually based on THC/CBD ratios of plants with undocumented hybrid backgrounds (with so-called “Indicas” often delimited simply on possession of more CBD than “Sativas”). The classification presented here circumscribes and names four taxa of *Cannabis* that represent critically endangered reservoirs of germplasm from which modern cannabinoid strains originated, and which are in urgent need of conservation.

## Introduction

*Cannabis* is an ancient domesticate, a triple-use crop. Archaeologists found fruits (“seeds”) in a food context, a kitchen midden, with a calibrated radiocarbon date of 8000 cal BCE (Kudo et al. 2009). Evidence of fiber use is nearly as old, although identifying ancient cordage as *Cannabis* (or pottery impressions of same) is somewhat subjective (McPartland and Hegman 2018). Artifacts from a drug context-burnt residues with cannabinoids in a censer – date to 500 cal BCE (Ren et al. 2019). Early words for *Cannabis* include Chinese *má*, attested *ca.* 750–600 BCE (Qu and Waley 1955), *qunubu*, a Neo-Assyrian loanword from the Scythian language, *ca.* 680 BCE (Seidel 1989), and *κάνναβις*, a Greek loanword from Scythian, *ca.* 440 BCE (Herotodus 2007).

The Latin name *Cannabis sativa* is usually attributed to Leonhart Fuchs, but the binomial was actually coined by Ermolao Barbaro, between 1480 and 1490, published 23 years after he died (Barbaro 1516). Carl Linné adopted the binomial in *Species Plantarum*, the internationally-recognized starting point of botanical nomenclature (Linnaeus 1753). Jean-Baptiste Lamarck broke from Linnaean orthodoxy by recognizing a second species, *C. indica*, for drug-type plants (Lamarck 1785).

Small and Cronquist (1976) proposed a single-species concept. They separated taxa by Linnaeus and Lamarck at the rank of subspecies, as C. sativa subsp. sativa and C. sativa subsp. indica (Lam.) E. Small & Cronq. The subspecies were circumscribed on the basis of ∆^9^-tetrahydrocannabinol (THC) content. They defined C. sativa subsp. sativa as containing <0.3% THC in dried flowering tops of female (pistillate) plants, and C. sativa subsp. indica as containing ≥0.3% THC. Numerous countries have incorporated the 0.3% criterion in regulations governing fiber-type (hemp) plants and drug-type (marijuana) plants.

Some botanists prefer to recognize *C. sativa* L. and *C. indica* Lam. at the rank of species (Hillig 2005a, Clarke and Merlin 2013). Debates over taxonomic rank are notoriously arbitrary. Molecular studies using DNA sequences can make the question of rank less arbitrary. Mandolino et al. (2002) quantified DNA polymorphisms in ten drug- and fiber-type varieties. They found more variability between individuals within a variety than between varieties – data that confirmed “the existence of a single, widely shared gene pool.” In a worldwide collection of *Cannabis*, Gilmore et al. (2007) found a low rate of sequence variation (approximately 1 polymorphism per 1 kb sequenced cpDNA) – consistent with a single species.

McPartland (2018) used DNA barcodes as a metric to place the *Cannabis* question of rank in context with other plants. He examined five plant barcodes (*rbcL*, *matK*, *trnH-psbA*, *trnL-trnF*, and *ITS1*), and calculated a mean divergence (barcode gap) of 0.41% between *C. sativa* and *C. indica*. This nearly equaled the mean divergence of 0.43% between five pairs of plants considered different varieties or subspecies (e.g., Camellia sinensis var. sinensis and C. sinensis var. assamica). In contrast, a 3.0% barcode gap separated five pairs of plants considered different species (e.g., *Humulus lupulus* and *H. japonicus*). Hebert et al. (2004) proposed a 2.7% difference between two *COI* sequences (the “barcode gap”) as the threshold for flagging genetically divergent specimens as distinct animal species.

Sawler et al. (2015) calculated a mean fixation index (*F*_ST_) of 0.156 between populations of fiber- and drug-type plants (n = 43 and 81, respectively). *F*_ST_ values range from 0 to 1; a zero value indicates the two groups freely interbreed; a 1 value indicates the groups are completely isolated from one another. A mean *F*_ST_ of 0.156 is similar to the degree of genetic differentiation between human populations in Europe and East Asia, which belong to a single species.

Lynch et al. (2016) calculated *F*_ST_ = 0.099 between fiber- and drug-type groups (n = 22 and 173, respectively). Grassa et al. (2018) calculated *F*_ST_ = 0.229 between fiber-type accessions and “marijuana,” by concatenating data from Sawler, Lynch, and their own sequencing. Hey and Pinho (2012) proposed *F*_ST_ = 0.35 as a conservative threshold measure for species differentiation; pairs with greater values are identified as separate species, pairs with lesser values are identified as subspecies populations. Clearly, *C. sativa* L. and *C. indica* Lam. are best differentiated at a subspecies rank.

**Figure 1.**
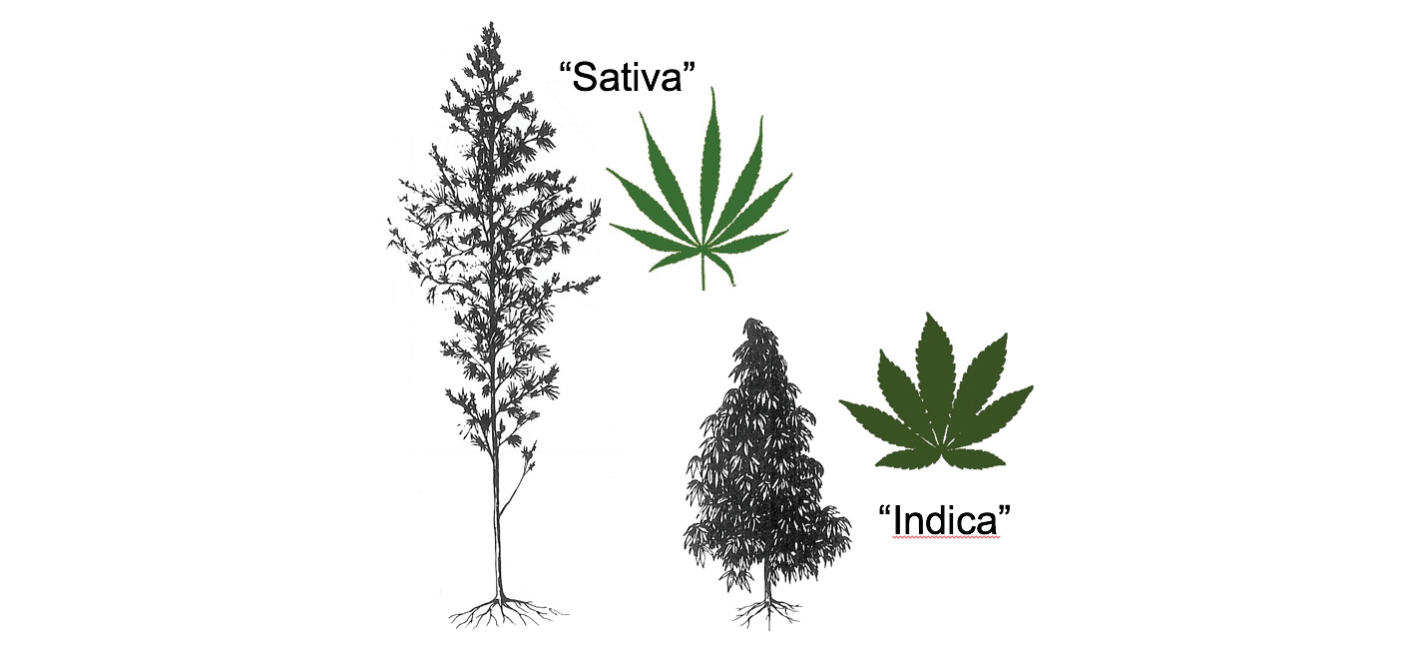
Line drawing adapted from Anderson (1980), courtesy of the Harvard University Herbaria and Botany Libraries.

In the 1980s, drug-type plants came to be divided into two categories, widely known by the ubiquitous labels “Indica” and “Sativa”. This vernacular taxonomy became widespread after Anderson (1980) published a line drawing of the plants (Fig. 1). He differentiated “Indica” and “Sativa” by morphology and geographical provenance. As summarized by de Meijer and van Soest (1992), “Indica” applied to plants with broad leaflets, short and compact habit, and early maturation, and there is evidence that landrace ancestors of such plants came from Central Asia (primarily Afghanistan). “Sativa” applied to plants with narrow leaflets, tall and diffuse habit, and late maturation, and there is evidence that landrace ancestors of such plants came originally from South Asia (primarily India), with early historical distribution to Southeast Asia, Africa, and the Americas.

Clarke (1981) accepted Anderson’s “Indica” concept for plants from Central Asia, “Strains from this area are often used as type examples for *Cannabis indica*.” In addition to morphological differences, he noted a phytochemical trait – Central Asian plants uniquely produced an acrid, skunk-like aroma. Clarke (1987) added an organoleptic quality – plants from Afghanistan produced a “slow flat dreary high.” Hillig (2005a) referred to Central Asian landraces as wide-leaflet diameter (WLD) biotypes, and landraces of South Asian heritage as narrow-leaflet diameter (NLD) biotypes. WLD and NLD biotypes differed in genetics (Hillig (2005a), morphology (Hillig 2005b), THC-to-cannabidiol (CBD) ratios (Hillig and Mahlberg 2004), and terpenoid content (Hillig 2004).

Recent authors have mistakenly equated the vernacular term “Sativa” with the epithet in the scientific name *C. sativa*, and mistakenly equated the vernacular term “Indica” with the epithet in the scientific name *C. indica*, mismatches first noted by McPartland et al. (2000). Small (2007) stated that “Sativa” and “Indica” were “quite inconsistent” with formal nomenclature. Linnaeus’s type specimen of *C. sativa* is a fiber-type (hemp) plant, not a drug-type (marijuana), and so the term “Sativa” has been inappropriately applied to drug-type plants (logically, it should be reserved for fiber-type hemp). Lamarck described *C. indica* for drug-type plants from India, and progenies in Southeast Asia and Africa – now counterintuitively called “Sativa” (logically, “Indica” should be reserved for the drug plants described by Lamarck).

The erroneous equivalences of vernacular “Sativa” (denoting plants with cannabinoids mostly or entirely THC) with “*C. sativa*” (in the narrow nomenclatural sense, denoting low-THC hemp forms), and vernacular “Indica” (denoting plants with substantial THC but also often substantial CBD) with “*C. indica*” (in the narrow nomenclatural sense, denoting high-THC, low-CBD forms) have appeared in taxonomic studies and legal documents. Even the pages of “Nature” have been problematically adorned with “Sativa” and “Indica”, accompanied by a version of Fig. 1 (Gould 2015). Those unfamiliar with the complexities and subtleties of biological classification can be misled, but in principle the issue is simple: the terms “Sativa” and “Indica” have been employed ambiguously and contradictorily.

In past centuries, landraces of South Asian heritage were grown over a much wider geographical range around the world than Central Asian landraces. The latter did not come to the attention of western *Cannabis* breeders until the early 1970s. Since then, breeders have haphazardly hybridized Central Asian and South Asian landraces, and largely obliterated their phenotypic differences (Clarke and Merlin 2013; Small 2017). Already 35 years ago, unhybridized landraces had become difficult to obtain in the USA and Europe (Clarke 1987). Hybrids of “Sativa” and “Indica” have proved overwhelmingly popular. “Indica” genes are useful for increasing cannabinoid yields, accelerating the maturity of outdoor plants at high latitudes, and reducing the height of plants so they are more easily concealed outdoors and more easily grown indoors. In the burgeoning CBD market, “Indica” genes (often from plants mislabeled “Ruderalis”) have increased the proportion of CBD relative to THC in plant products.

Alarmingly, Central and South Asian landraces have been corrupted by the introduction of foreign germplasm into their centers of diversity. Beisler (2006) boasted of importing “Mexican Gold” into Afghanistan around 1972. Casano (2005) noted that Afghani landraces were “disappearing” due to hybridization with other drug-type plants. Conversely, Central Asian landraces were introduced into South Asian centers of diversity in the 1970s – into Nepal (Cherniak 1982), Jamaica (Lamb 2010), and Thailand (Clarke and Merlin 2016). By 1980, Afghani landraces were imported into southern Kashmir, cultivated for sieved *hashīsh*, and escapes grew near crop fields (Clarke 1998). Also in the 1980s, Central Asian genetics were introduced into South Africa (Peterson 2009) and Morocco (Clarke and Merlin 2016). Sharma (1988) wrote about “hybrid *Cannabis*” growing in Kullu, Himachal Pradesh, and he implicated “foreign nationals.”

Central and South Asian landraces face extinction through introgressive hybridization. Wiegand (1935) first described this phenomenon in plants. Introgression refers to the infiltration of genes between taxa through the bridge of F_1_ hybrids. Fertile offspring from these crosses may display hybrid vigor (enhanced fitness), and replace one or both parental populations (Ellstrand 2003). Recent phylogenetic studies of populations allegedly representing “Indica” and “Sativa” show little or no genetic differences, because these studies primarily analyzed hybrid “strains” (Sawler et al. 2015; Dufresnes et al. 2017; Schwabe and McGlaughlin 2018). These results conflict with studies of landraces collected in the 1970s–1990s, which showed much clearer genetic differences (Hillig 2005a; Gilmore et al. 2007).

The use of “strain” names for Indica–Sativa hybrids began with Watson (1985). A database of strain names currently lists 14,348 of them (Seedfinder 2019). This crowd-sourced enterprise – crossing and re-crossing hybrids of largely clandestine parentage – has resulted in a loss of genetic diversity (Mudge et al. 2018). Most strains sold by seed companies are characterized as “Sativa-dominant” or “Indica-dominant.” The arbitrariness of these designations is illustrated by “AK-47”, a hybrid strain that won “Best Sativa” in the 1999 Cannabis Cup, and won “Best Indica” four years later (McPartland 2017). Conceptually, a “strain” is equivalent to a “cultivar,” the latter being a taxonomic rank recognized by the “International Code of Nomenclature for Cultivated Plants” (ICNCP, Brickell et al. 2016). However, few commercial “strains” of drug-type *Cannabis* have met ICNCP requirements for cultivar recognition (Small 2015).

The ICNCP clusters cultivars into “Groups”. Consistent with ICNCP requirements, Small (2015) designated Central Asian landraces as “Cannabis Group Narcotic, THC/CBD Balanced,” and South Asian landraces as “*Cannabis* Group Narcotic, THC Predominant.” Some botanists argue that plants with traits created by human selection should be assigned cultivar status under the ICNCP, rather than assigned taxa under the “International Code of Nomenclature for Algae, Fungi, and Plants” (ICN, Turland 2018). However, for pragmatic reasons, botanists use the ICN framework to assign taxa to artificially selected plants (e.g., Hammer and Gladis 2014).

The above information has dealt basically with domesticated material. In addition, “wild” plants are also of concern. *Cannabis* “wild-type” traits were first described by Zinger (1898): small achene size, a persistent perianth with camouflagic mottling, and an elongated base – drawn out in the shape of a short, tapered stub with a well-developed abscission layer. In contrast, domesticated plants express a suite of phenotypic traits (the “domestication syndrome”) absent in wild-type plants, such as enlarged seed size, a lack of seed shattering (from reduction of the abscission zone), and reduction of perianth adherence.

Domesticated *Cannabis* easily escapes cultivation and goes “feral.” Domesticated *C. sativa* reverted to a wild-type phenotype in Canada just 50 generations (years) after cultivation was prohibited (Small 1975). This rapid phenotypic evolution makes it difficult to distinguish truly wild plants from formerly cultivated plants that have reverted to wild-type phenotypes. Thus *Cannabis* plants growing outside of cultivation could be (1) “volunteers” (escaped very recently from cultivation, maintaining their domesticated characteristics, and growing near where they were cultivated); (2) “escapes” that have readapted to wild existence (growing in various habitats, typically in disturbed or weedy places); or (3) “aboriginal” (unaltered by domestication and growing in their indigenous areas).

Aboriginal populations of several of the world’s most important crops do not seem to have survived, and *Cannabis* may be of this nature. Regardless, the wild-growing plants of Asia that are near (sympatric or parapatric) to the domesticates are of special significance. They may be direct ancestors of the domesticates, although this remains to be ascertained – many ancient domesticates were domesticated in locations distant from their sites of origin (Jarvis et al. 2016). In any event, there is considerable likelihood that the nearby wild plants of the domesticates share genes, since *Cannabis* produces massive quantities of pollen that is distributed for vast distances, and all *Cannabis* populations are capable of cross-pollination and completely interfertile (Small 1972). Accordingly, the wild varieties recognized in this publication represent very significant potential sources of genes representative of the endangered “Sativa” and “Indica” genomes.

This study does not address the European subspecies, C. sativa subsp. sativa. Small and Cronquist (1976) segregated this subspecies into two varieties – domesticated and wild-type plants. The domesticated variety is composed of fiber-type and oilseed landraces and cultivars. The wild-type variety has nomenclatural issues regarding C. sativa var. spontaneaVavilov (1922) and *C. ruderalis* (Janischevsky 1924). Vavilov and Janischevsky assigned these separate taxa to the same population of wild-type plants growing near Saratov, Russia. “Ruderalis” has become a mainstay of today’s vernacular taxonomy (Anderson 1980). See Suppl. material 1: SF.2 for a discussion of these nomenclatural issues, and an elaboration of “wild-type nominalism” in SF.3b.

Worldwide introgressive hybridization of “Indica” and “Sativa” threatens the agrobiodiversity of *C. sativa*. Seen pessimistically, the varieties described here are components of a vanishing world, and classifying them is like an exercise in renaming dinosaurs. Optimistically, the formal recognition of indigenous Central and South Asian varieties will provide them with unambiguous names, and may help prevent their extinction.

## Methods

Taxonomic characters for analysis included aspects of morphology, phytochemistry, genetics, and host-parasite relationships. Some data are new (morphological studies of herbarium specimens), whereas phytochemical and molecular data were extracted from previously published studies. Most of those studies employed common garden experiments (CGEs). CGEs grow plants from different places in a single location, under common environmental conditions, with uniform processing (Grassi and McPartland 2017).

### Morphological characters

Approximately 1,100 herbarium specimens were examined, at 15 herbaria, designated by herbarium acronyms in Index Herbariorum (Suppl. material 1: SF.4). Additionally, we extracted morphological data from CGEs that compared Central and South Asian germplasm collected in the previous century (e.g., Vavilov and Bukinich 1929, Small et al. 1976, Anderson 1980, de Meijer 1994, Hillig 2005b). We also drew on morphological data from archaeobotanical studies. In the spirit of open access, extracted morphological data are provided in Suppl. material 1: SF.8, permitting readers to synthesize the raw data for themselves. CGE studies provided data often absent in herbarium specimens, such as plant height, internode length, stalk thickness, and branch angle or divarication.

Branch angle or divarication measured the angle, in degrees, that a branch came off the vertical shoot; it generally ranged between 35° to 85° from vertical. Branch angle may be a function of internode length, which was also assessed. Branch flexibility is a qualitative measure of the ability of a branch to bend or droop without snapping. Flexibility likely reflects the ratio of bast fiber (flexible) to wood fiber (inflexible). Leaf morphology was assessed in “fan leaves” (i.e. larger palmately compound leaves) near the base of inflorescences. The sampled leaves conformed to the concept of 1^st^ order branching off the main shoot, as presented by Spitzer-Rimon et al. (2019). Central leaflet length/width ratio (L/W) is expressed as a quotient. Leaflet shape was either lanceolate (the widest part is less than midway down the length of the leaflet from its base), or oblanceolate (where the widest location is more than half way down the length). This was measured as the distance to the widest point (WP) divided by the entire length (WP/L). A leaflet with WP/L > 0.5 is oblanceolate (Anderson 1980).

The perigonal bract (also called bracteole, perigonium, or inappropriately “calyx”) is the floral bract enclosing the female flower and later the achene (Small 2015). Inflorescence density was qualitatively assessed using the “perigonal bract-to-leaf index” (i.e., the “calyx-to-leaf ratio,” Clarke 1981). Inflorescences with a low index have a predominance of leaf material – interstitial “sugar leaves” (relatively small leaves with few leaflets occurring in the inflorescence) between clusters, subtending 2^nd^ order to 7^th^ order branchlets (Spitzer-Rimon et al. 2019). A low index is associated, in part, with short internode length and broad leaflet width.

The density of capitate-stalked glandular trichomes (CSGTs) was qualitatively assessed (i.e. visually evaluated) on perigonal bracts. CSGT density was mentioned by Christison (1850) in one of the first CGEs that compared *C. sativa* (Scottish hemp) and *C. indica* (Indian *gunjuh*). He noted that *C. indica* inflorescences felt resinous when touched, “Floral leaves, bracts, and perianth covered with glandular pubescence.” He also noted that *C. indica* leaves produced “both sessile glands and glandular hairs [CSGTs].” CSGT density on sugar leaves was also qualitatively assessed, based on the method by Potter (2009).

As used here, the “fruit” includes the achene and its more or less adherent perianth. In female flowers of *Cannabis*, the perianth does not produce a corolla, but instead adheres to the exocarp (outermost layer of the achene wall). Dimensions and appearance of the fruit were assessed.

For each herbarium specimen, a standardized form was used to record specimen label data (collector name, date, location, annotations) and morphological data. During the course of this study, morphological characters were added (e.g., branch angle, inflorescence density, CSGT density), necessitating return visits to some herbaria (BM, ECON, GH, IND, K). Morphological data were synthesized qualitatively (e.g., branch flexibility, leaf color, inflorescence density, CSGT density, perianth adherence), or quantitatively (e.g., plant height, internode length, leaflet L/W and WP/L ratios, achene size). Quantitative data provided bracket measurements for each described taxon.

### Phytochemical characters

A widely-cited paper by Turner et al. (1980) listed 420 phytochemicals isolated from *C. sativa* – the 420 plant. Few phytochemicals provide useful taxonomic information, however. Our study focused on cannabinoids and terpenoids. In living plants and freshly harvested tissues, cannabinoids exist predominantly in the form of carboxylic acids. THC occurs as tetrahydrocannabinolic acid (THCA); cannabidiol (CBD) occurs as cannabidiolic acid (CBDA). Decarboxylation of the cannabinoids into their neutral counterparts occurs relatively slowly with aging, and rapidly with heat. Thus THCA converts to THC, and CBDA converts to CBD. In addition, when THC ages (unless appropriately stored) it substantially transforms to cannabinol (CBN), an oxidation product. In this paper when THC and CBD are mentioned it should be understood that depending on context, “THC” may mean THCA + THC + CBN, and “CBD” may mean CBDA + CBD.

Rather than cannabinoid *quantity* (i.e., THC% w/w), we report a parameter measuring cannabinoid *quality*: the THC/CBD ratio (THC% w/w divided by CBD% w/w). The THC/CBD ratio is a quite conservative (stable) character, whereas THC% correlates with morphology, such as trichome density (Potter 2009), as well as inflorescence density and gland head size. These morphological differences do not alter the THC/CBD ratio. The ratio is determined by a single gene with codominant alleles (de Meijer et al. 2003), or two tightly-linked yet separate *THCAS* and *CBDAS* genes (Van Bakel et al. 2011, Laverty et al. 2019). Weiblen et al. (2015) identified a single quantitative trait locus (QTL) associated with the THC/CBD ratio.

In contrast, THC% expression is polygenic, altered by many genes that contribute to morphological differences. Environmental factors (light intensity, temperature, soil nutrients, etc.) alter THC%, but have much less effect on THC/CBD. As a dimensionless ratio, THC/CBD provides a more valid comparison of many studies that grew plants under different conditions (Grassi and McPartland 2017).

Tetrahydrocannabivarin (THCV) and cannabidivarin (CBDV) are short-tailed C_19_ analogs of THC and CBD. The biosynthetic pathway leading to THCV and CBDV diverges early, on the resorcinol side of the cannabinoid pipeline. Some researchers add C_19_ analogs to THC/CBD ratios, as THC+THCV/CBD+CBDV (e.g., Turner et al. 1980). Here, the percentage of C_19_ analogs (THCV%+CBDV%) is treated as a separate character.

Terpenoids constitute the “essential oil” of *Cannabis*. Terpenoids include hydrocarbon terpenes and their oxygenated derivatives, which form alcohols, ethers, aldehydes, ketones, and esters. They are volatile, and give the plant its characteristic smell. Christison (1850) noted that Indian *gunjuh* emitted a balsamic odor, lacking in Scottish hemp. South Asian landraces often smell “herbal” or “sweet,” whereas Central Asian landraces give off an acrid or “skunky” aroma (Clarke 1981).

### Genetic characters

Molecular genetic studies of Central and South Asian populations – which have not been significantly hybridized in recent times – are limited in number. Twenty years ago, when unhybridized landraces were much more readily available, molecular methods were blunt instruments. Today, we can decode the DNA sequence of whole genomes, but a good representation of the range of unhybridized biodiversity is not available for analysis, although collection of genuinely representative germplasm from Asia may still be possible. Herbaria of course are invaluable repositories of older specimens, but collections from Asia are relatively limited, and for various reasons, curators have often been unable to allow sampling of older collections.

Herbarium voucher specimens were deposited for some CGE studies (Small and Beckstead 1973; Turner et al. 1973, 1979; de Meijer et al. 1992; de Meijer 1994; Hillig 2004, 2005a; Hillig and Mahlberg 2004; Gilmore et al. 2007), which we examined to ascertain correlations with morphology. For other phytochemical and genetic studies, we relied upon reports of geographic provenance of their accessions.

## Results

The electronic version of this article in Portable Document Format (PDF), in a work with an ISSN or ISBN number, represents a published work according to the ICN (Turland 2018). Hence the new names contained in the electronic publication of this article are effectively published under the ICN from the electronic edition alone. New names contained in this work have been submitted to the International Plant Names Index (IPNI, http://www.ipni.org), from where they will be made available to the Global Names Index.

**Figure 2.**
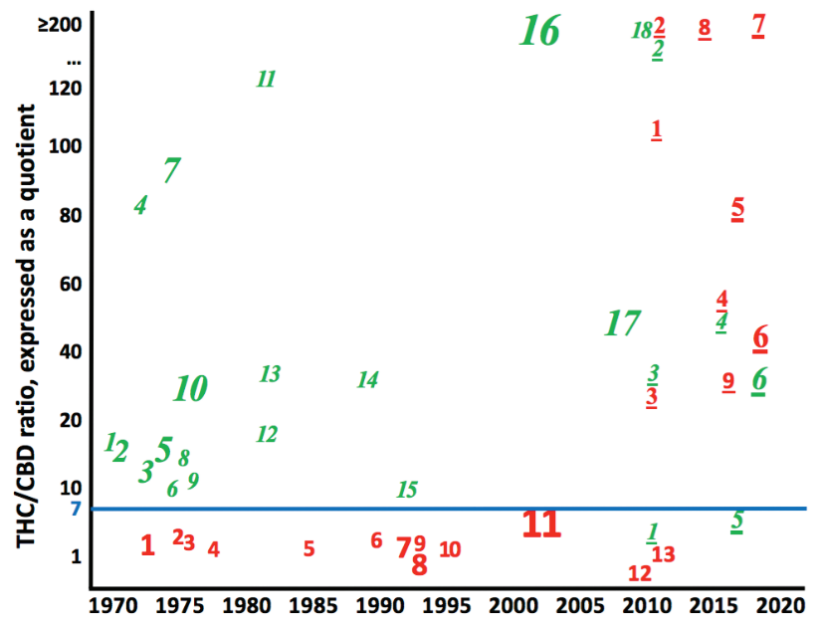
Shifts in THC/CBD ratios over time; data from 47 numbered studies in Suppl. material 1: SF.9. Central Asian landraces in unitalicized red (n =13 studies); “Indica” in underlined unitalicized red (n= 9); South Asian landraces in *italicized green* (n =18 studies); “Sativa” in *underlined italicized green* (n =7 studies). Size of numeral reflects the number of accessions analyzed in that study.

An example of a taxonomic trait shifting over the past 50 years, as Central Asian landraces hybridized into “Indica”, is provided in Fig. 2. It illustrates a convergence in THC/CBD ratios over the past 50 years. In studies of accessions collected in the 1970s–1990s, Central Asian landraces (study numbers in unitalicized red font), the THC/CBD ratio, expressed as a quotient, was always < 7 (study size weighted mean = 3.56). In studies of South Asian landraces collected in the 1970s–1990s (study numbers in italicized green font), the THC/CBD ratio was ≥ 7 (study size weighted mean = 97.14). Since then, THC/CBD ratios have skyrocketed in accessions purportedly representing Central Asia (i.e., “Indica”). Now there is little or no difference between “Indica” and “Sativa”.

### Taxonomic analysis

We classified C. sativa subsp. indica into four varieties (in the formal nomenclatural sense, i.e., varietas). Two varieties express traits of domestication (identical to “Indica” and “Sativa” in the original narrow meanings of these terms), and two varieties have wild-type traits. We followed precedent set by Small and Cronquist (1976) who segregated C. sativa subsp. indica into two varieties – domesticated and wild-type plants. They did not place these varieties in an ancestor–progeny relationship, however, because they could not verify putative ancestral relationships.

### Key to four varieties of C. sativa subsp. indica^1^

**Table:** 

1.	Plants usually with a THC/CBD ratio ≥7; terpenoid profile usually lacks sesquiterpene alcohols, fresh aroma often pleasant. Plants ≥ 2 m tall in good habitats; branches flexible, diverging from the shoot at a relatively acute angle (<45° from vertical). Fresh leaves medium green in color; central leaflets narrow (length/width usually >6), lanceolate to linear-lanceolate; margins with fine to coarse serrations, sometimes biserrate. Mature female inflorescence somewhat compact (flowering stems producing small to medium “buds”), with relatively obscure sugar leaves (a high perigonal bract-to-leaf index); sugar leaves with capitate-stalked glandular trichomes (CSGTs) usually limited to the proximal half of the leaves; perigonal bracts express a moderate to high density of CSGTs. Mature achene exocarp color (beneath the perianth) often green-brown.	
A	THC/CBD ratio always ≥7, often much more. Mature achenes usually ≥ 3.6 mm long (Fig. 3e, f); perianth mostly sloughed off, but often persistent in places (appearing as irregular spots or stripes); exposed exocarp exhibiting prominent venation; lacking a prominent protuberant base; not readily disarticulating from plant	**var. *indica*** (“Sativa” in the historical sense^2^)
B	THC/CBD ratio usually ≥7, sometimes less. Mature achenes usually <3.6 mm long (Fig. 3g, h); perianth persistent (covering exocarp and its venation), with strong pigmentation in a mottled or striped pattern; with a protuberant base; readily disarticulating from plant	**var. himalayensis**
2.	Plants with a THC/CBD ratio <7; terpenoid profile includes sesquiterpene alcohols, fresh aroma often acrid or “skunky.” Plants < 2 m tall in good habitats, and often *ca.* 1 m; branches not flexible, branching sometimes nearly 90° from the stalk axis, producing a menorah-shaped habitus. Fresh leaves dark green in color, leaflets of larger leaves sometimes overlap; central leaflets broad (length/width usually <6), often oblanceolate; margins with coarse serrations, rarely biserrate. Mature female inflorescence compact (flowering stems producing medium to large “buds”) with prominent sugar leaves (a low perigonal bract-to-leaf index); sugar leaves have CSGTs extending more than half way down their length; perigonal bracts densely covered with CSGTs. Mature achene exocarp color (beneath the perianth) often a lighter shade of olive green to gray.	
A	THC/CBD ratio <7 (almost always >2). Mature achenes usually ≥ 3.6 mm long (Fig. 3a, b); perianth mostly sloughed off (appearing as irregular spots or stripes); exposed exocarp exhibiting prominent venation; lacking a prominent protuberant base; not disarticulating from plant, and often trapped in the dense inflorescence	**var. afghanica** (“Indica” in the historical sense^2^)
B	THC/CBD ratio often <2. Mature achenes usually < 3.6 mm long (Fig. 3c, d); perianth persistent (covering exocarp and its venation), with strong pigmentation in a mottled or striped pattern; with a protuberant base; readily disarticulating from plant	**var. asperrima**
^1^ As emphasized in the text, the differences presented here represent unhybridized plants, before extensive recent hybridization between them.		
^2^ Historically, as discussed in the text, “Sativa” formerly represented landraces of South Asian heritage, and “Indica” formerly represented Central Asian landraces. This key is not intended for the identification of “Sativa” and “Indica” strains commercially available today.		

**Figure 3.**
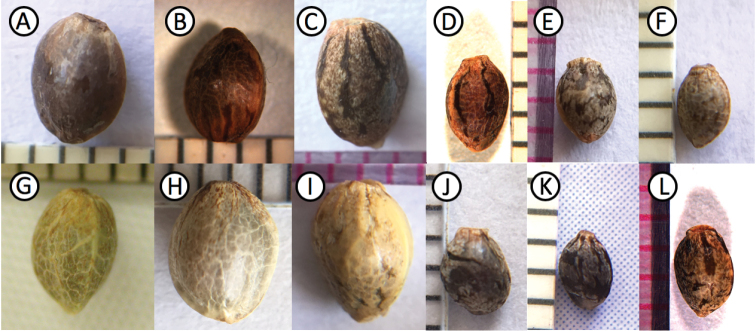
Representative achenes of four varieties **A***indica*, Rajshahi (Bangladesh), *Clarke* 1877 (BM) **B***indica*, Coimbatore (India), *Bircher* 1893 (K) **C***indica*, South Africa, *Hillig* 1996; (IND) **D***himalayensis* neotype **E***himalayensis*, Bareilly (India), *Roxburgh* 1796 (K). **F***himalayensis*, East Bengal (Bangladesh) *Griffith* 1835 (GH) **G***afghanica* neotype **H***afghanica* epitype **I***afghanica* Yarkant (Xīnjiāng), *Henderson* 1871 (LE) **J***asperrima* lectotype **K***asperrima* Nuristān (Afghanistan), *Street* 1965 (F) **L** Kailiyskiy Alatau (Kazakhstan), *Semenov-Tyan-Shansky* 1857 (LE).

### Taxonomic treatment

Please note that light quality varied among herbaria, so photographs of herbarium specimens and achenes at different herbaria varied somewhat in their tint, hue, and tone. For protologues of the four varieties (everything associated with a basionym at its time of publication), see Suppl. material 1: SF.6. For additional representative herbarium specimens of the four varieties, see Suppl. material 1.

### Variety 1: South Asian domesticate

#### Cannabis sativa subsp. indica var. indica

Taxon classificationPlantaeRosalesCannabaceae

(Lam.) Persoon, Synopsis Plantarum 2: 618, 1807.

853D07C1-AFBA-5E84-891A-22FD1ACF4E24

Figure 4a

Cannabis indica Lamarck, Encyclopédie Méthodique 1(2): 694–695, 1785 Basionym. See McPartland (1992) for justification of citing Persoon as the authority in the comb. nov, not Wehmer as treated in Small and Cronquist (1976).
≡ C. sativa var. indica (Lam.) Fristedt, Upsala Läkareförenings Förhandlingar 5: 504, 1869–1870.
≡ C. sativa f. indica (Lam.) Voss in Siebert & Voss, Vilmorin’s Blumengärtnerei 1: 912, 1896.
≡ C. sativa var. indica (Lam.) Wehmer, Die Pflanzenstoffe p. 248, 1911.
= C. sativa var. indica Blume, Bijdragen tot de flora van Nederlandsch Indië, p. 515, 1825.
= C. macrosperma Stokes, *Botanical Materia Medica* 4: 539, 1812.
≡ C. sativa B macrosperma (Stokes) Ascherson & Graebner, Synopsis Mitteleuropäischen Flora 4: 599, 1911.
≡ C. sativa var. macrosperma (Stokes) Chevalier, Revue de Botanique Appliquée et d’Agriculture Coloniale 24: 64, 1944.
= C. sativa γ *crispata* Hasskarl, Neuer Schlüssel zu Rumph’s Herbarium amboinense p. 112, 1886.
= C. sativa β *vulgaris* de Candolle, Prodromus 16(1):31, 1869 (en part, based on plants cultivated in India).
= C. americana Houghton & Hamilton, Proc. Am. Pharm. Assoc. 55: 445, 1907, nomen nudum.
≡ C. americana Wehmer, Die Pflanzenstoffe, 2: 157, 1911, nomen nudum.
= C. madagascar Pearson, Proc. Penna. Pharm. Assoc. 1909: 179, 1909, nomen nudum.
= C. africana Glickman, Mulford’s Veterinary Bulletin 4(2): 88, 1912, nomen nudum.
≡ C. sativa var. africana Wehmer, Die Pflanzenstoffe 2: 39, 1935.
= C. mexicana Stanley, Am. J. Police Science 2(3): 252, 1931, nomen nudum.

##### Holotype.

India, likely Pondicherry, *Lamarck*, no date, annotated “Chanvre rapporte de l’Inde par M. Sonnerat” (herb. P). Most of Pierre Sonnerat’s herbarium specimens at herb. P were collected around Pondicherry between 1775 and 1778.

##### Diagnosis.

Plants with THC% ≥0.3% in inflorescence and a THC/CBD ratio always ≥7, often much more; central leaflet length:width ratio ≥6 in fan leaves near the base of inflorescences; mature achenes usually ≥ 3.6 mm long, the perianth mostly sloughed off, lacking a prominent protuberant base, and lacking a well-developed abscission zone that allows easy disarticulation.

##### Morphology.

Plants usually >2.0 m tall (shorter in inhospitable situations). Central stem (stalk) internodes relatively long (often >12 cm, shorter in shorter plants), somewhat hollow (up to 1/3 stem diameter). Branches flexible, diverging from the stalk at relatively acute angles (around 45°). Leaf palmately compound, largest leaves typically with at least 7 leaflets, leaflet edges not overlapping. Central leaflet long and narrow, lanceolate or linear-lanceolate in shape; margins with moderately coarse serrations, and rare secondary serrations. Female inflorescence (and infructescence) elongated and somewhat diffuse, with relatively obscure sugar leaves (a high perigonal bract-to-leaf index). Sugar leaves with CSGTs limited to the proximal half. Perigonal bract covered with a moderate density of CSGTs. Perianth membranous, hyaline with pigmented areas (brown and mottled or marbled in appearance); mostly sloughed off but sometimes persistent. Achene, usually ≥ 3.6 mm long, globose to elongate, exocarp green-brown; abscission zone poorly developed.

##### Phytochemistry.

Dried female inflorescences: THC ≥0.3%, in late 20^th^ century accessions, nearly always >1.0%; literature weighted x¯ = 3.97%, up to 12.5%. THC/CBD ratio ≥7, and often >100. THCV is commonly present, especially in landraces from South Asia and Africa. Hillig and Mahlberg (2004) report THCV+CBDV% content x¯ = 0.25%. Terpenoid profile often imparts an “herbal” or “sweet” aroma, with terpinolene, *β*-caryophyllene, trans-*β*-farnesene, and *a*-guaiene content significantly higher than Central Asian plants.

##### Genetics.

Landraces of South Asian heritage segregated from Central Asian landraces in an allozyme analysis (Hillig 2005a) and cpDNA haplotype study (Gilmore et al. 2007). “Sativa” and “Indica” were segregated with STR loci (Knight et al. 2010), RAPD markers (Piluzza et al. 2013), and nDNA SNP haplotypes (Henry 2015; Lynch et al. 2016). Other studies showed little or no genetic differences between “Sativa” and “Indica” (Sawler et al. 2015; Dufresnes et al. 2017), or their phenotypes matched poorly with their purported genotypes (Schwabe and McGlaughlin 2018).

##### Other characters.

Generally late maturing; monoecious plants relatively common compared to the other varieties; susceptible to black mildew caused by *Schiffnerula cannabis*.

##### Provenance and uses.

Originally cultivated in India for *gañjā*, and spread at an early date to southeast Asia, Africa, and the Americas.

**Figure 4.**
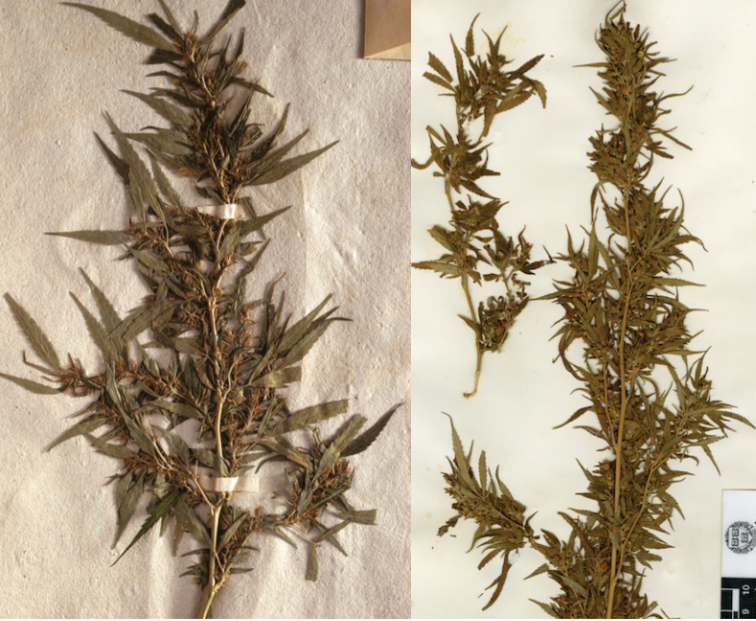
Two varieties of C. sativa subsp. indica from South Asia. On left **a** var. *indica*. On right **b** var. himalayensis.

### Variety 2: South Asian wild-type

#### Cannabis sativa subsp. indica var. himalayensis

Taxon classificationPlantaeRosalesCannabaceae

(Cazzuola) McPartl. & E.Small

69AF8A9C-1FAF-533E-B010-86732B3C9DF2

Figure 4b

Cannabis sativa var. hymalaiensis Cazzuola, Il Regno vegetale tessili e tintoriale, p. 49, 1875 (misspelling corrected *apud* ICN Article 60.1) Basionym.
≡ C. sativa var. hymalaiensis Cazzuola, Nuovo Giornale Botanico Italiano 5: 262, 1873, nomen nudum.
≡ C. sativa var. himalayensis Cazzuola, Dizionario di botanica, p. 105, 1876 (later homonym).
= C. sativa var. himalayensis Koch, Annales des Sciences Naturelles Botanique (Series 4) 1: 352, 1854, nomen nudum.
= C. sativa β *vulgaris* de Candolle, Prodromus 16(1):31, 1869 (*en part*, based on plants growing spontaneous in northern India and Burma).
= C. sativa α indica f. montana Fristedt, Upsala Läkareförenings Förhandlingar 5: 507, 1869- 1870, nomen nudum.
= C. himalyana Zinger, Flora oder Allgemeine Botanische Zeitung 85: 207, 1898, nomen nudum.
= C. sativa subsp. indica sect. spontanea var. spontanea Clarke, Cannabis Evolution p. 224, 1987, nomen invalidum.

##### Neotype.

Designated herein, INDIA: Himachal Pradesh, Shimla or Kinnaur (“Himalaya Boreal. Occident., Regio Temp.”), *T. Thompson*, 1847 (GH). No *himalayensis* specimens exist in the herbaria of Cazzuola or Koch (pers. communications, Lucia Amadei, herb. PI; Robert Vogt, herb. B). Thompson’s specimen was designated as neotype because it represents the best of several collections he made in the Himalaya. It was distributed as an exsiccatum, with duplicates at several herbaria, providing isoneotypes (BM! K! LE! US!).

##### Diagnosis.

Plants with THC% ≥0.3% in inflorescence and a THC/CBD ratio often ≥7, sometimes less; central leaflet length:width ratio ≥6 in fan leaves near the base of inflorescences; mature achenes usually <3.6 mm long, with a persistent perianth and a protuberant base, and readily disarticulating from plant by a well-developed abscission zone.

##### Morphology.

Plants 1.0–3.0 m tall. Central stem (stalk) internodes relatively long (often >10 cm, shorter in shorter plants), somewhat hollow (up to 1/2 stem diameter). Branches flexible, diverging from the stalk at relatively acute angles (around 45°). Leaf palmately compound, larger leaves usually with at least 7 leaflets, leaflet edges not overlapping. Central leaflet long and narrow, lanceolate in shape; margins with moderately coarse serrations, and rare secondary serrations. Female inflorescence (and infructescence) elongated and somewhat diffuse, with relatively obscure sugar leaves (a high perigonal bract-to-leaf index). Sugar leaves with CSGTs limited to the proximal half. Perigonal bract covered with a moderate density of CSGTs. Perianth membranous, hyaline with pigmented areas (brown and mottled or marbled in appearance); always persistent. Achene usually <3.6 mm long, exocarp green-brown; with an elongated base and abscission zone that is relatively narrow.

##### Phytochemistry.

Dried female inflorescences: THC ≥0.3% (although two studies report plants with THC <0.3%); weighted x¯ = 1.49%, range between 0.06% and 9.3%. THC/CBD ratios vary; two studies (those with THC <0.3%), who shared accessions, reported ratios of only 1.28 and 1.56; these accessions may represent East Asian fiber-type domesticates that reacquired wild-type traits. Ratios in other studies are >10, even >100. THC content and THC/CBD ratios are skewed by THCV%+CBDV%, which is higher than any other variety: x¯ = 0.90% (Hillig and Mahlberg 2004). The terpenoid profile is similar to that of var. *indica*, except for higher levels of *β*-myrcene, *cis*-ocimene, and *β*-caryophyllene.

##### Genetics.

Allozyme analysis (Hillig 2005a) partially segregated wild-type accessions from South Asian domesticates. He proposed that wild-type accessions from the Himalaya represented the ancestral source of South Asian domesticates.

##### Other characters.

Generally late maturing; achenes fall from plant at maturity. Bast fiber content (as a percent of stalk dry weight) in Himalayan plants is higher than plants grown exclusively for drugs in southern India (Bredemann 1952; de Meijer 1994).

##### Provenance and uses.

Wild-growing (possibly indigenous) populations occur throughout montane India, Nepal, and Bhutan, where they are harvested for bast fiber (stalks), *bhāng* (leaves), hand-rubbed *charas* (*hashīsh*), or achenes (seeds). Achenes in some herbarium specimens from the Himalaya were relatively large with a reduced abscission mechanism, indicating the presence of genes from domesticated plants.

##### Basionym notes.

Cazzuola spelled the epithet *himalayensis* variously between 1873 and 1876. His earliest publication did not provide a clear diagnosis, a nomen nudum, not validly published (*ICN* Art. 38.2, Turland 2018). Koch also proposed a taxon *himalayensis* without a clear diagnosis, and he equated it with the South Asian domesticate – an erroneous concept.

### Variety 3: Central Asian domesticate

#### Cannabis sativa subsp. indica var. afghanica

Taxon classificationPlantaeRosalesCannabaceae

(Vavilov) McPartl. & E.Small stat. nov.

0B09E283-146C-54FF-9C94-3025BA620455

urn:lsid:ipni.org:names:77208272-1

Figure 5

Cannabis sativa f. afghanica Vavilov, *Trudy po Prikladnoi Botanike*, *Genetike i Selektsii* 16(2): 227, 1926 (Basionym).
≡ C. indica var. afghanica Vavilov in Vavilov & Bukinich, *Trudy Po Prikladnoi Botanike*, *Genetike i Selektsii* 33 (Suppl.): 380, 1929, orthographic variant.
≡ C. indica var. kafiristanica f. afghanica Vavilov in Vavilov & Bukinich, *Trudy Po Prikladnoi Botanike*, *Genetike i Selektsii* 33: 381, 1929.
= C. sativa subsp. culta prol. *asiatica* var. narcotica Serebriakova in Serebriakova & Sizov, *Kul’turnaya Flora SSSR* 5: 36, 1940 (no Latin diagnosis and not typified).
= C. afghanica var. turkistanica Clarke, Cannabis Evolution p. 225, 1987, *nomen invalidum*.
= C. sativa var. afghanica McPartland, *Hemp Diseases & Pests* p. 4, 2000, nomen nudum.
= C. sativa var. afghan, Sands, U.S. patent 6,403,530, 2002, nomen nudum.

##### Neotype.

**Figure 5.**
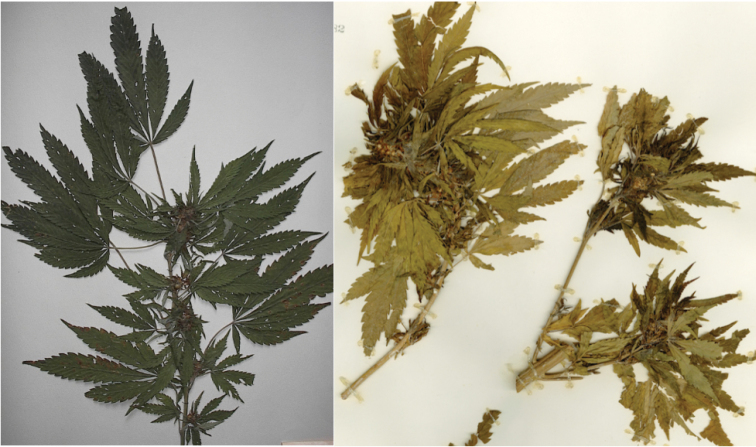
Type specimens of C. sativa subsp. indica var. afghanica. Neotype on left (**a**), epitype on right (**b**).

Designated herein: Afghanistan: Ghazni Province (formerly Kandahar Province), Gui-Akhen (Гуй-Ахен) village near Qala-i Murvardar (Кала-и Мурвардар), on the Ghazni-Kandahar road, *Vavilov*, 1924, from seed sown by Serebriakova in 1926 at North Caucasus Experiment Station, Maikop, Krasnodar Krai (labeled *Cannabis sativa*, WIR 609, 3945). Fig. 5a. No specimen labeled *afghanica* exists at WIR (McPartl., pers. observation, WIR 2010). The achene illustration in Vavilov and Bukinich (1929) cannot serve as lectotype because it is not part of the protologue, which appears in Vavilov (1926).

##### Epitype.

Designated herein, explicitly supporting the neotype: Afghanistan: Kandahar Province, near Kandahar, *Schultes*, XII.13–20.1971 (ECON 26505). Fig. 5b. The ICN defines an epitype as a specimen selected as an interpretive type when the holo-/lecto-/neotype is suboptimal for critical identification (Turland 2018). ECON 26505 serves as an epitype because its morphology unambiguously agrees with the widespread concept of “Indica”. ECON 26505 also serves as a typotype – a photograph of the specimen, when alive and in the ground, which appears in Schultes et al. (1974), and is reproduced in Suppl. material 1: SF.8.

##### Diagnosis.

Plants with THC% ≥0.3% in inflorescence and a THC/CBD ratio <7 (almost always >1); central leaflet length:width ratio <6 in fan leaves near the base of inflorescences; mature achenes usually ≥ 3.6 mm long, the perianth mostly sloughed off, lacking a prominent protuberant base, and lacking a well-developed abscission zone that allows easy disarticulation.

##### Morphology.

Plants usually < 2 m tall, often <1 m. Central stem (stalk) internodes short (often 5–11 cm), mostly solid, central hollow usually less than 20% of stalk diameter. Branches in well-developed plants begin close to ground level, at an angle sometimes nearly 90° from the stalk axis, producing a menorah-shaped habitus. Leaf palmately compound, largest leaves typically with 7–11 leaflets, leaflet edges often overlapping, color dark green (“black hemp” Vavilov 1992). Central leaflet long and broad, often oblanceolate in shape; margins with coarse serrations, secondary serrations rarely seen. Female inflorescence (and infructescence) compact, often agglutinated with trichome exudate, with prominent sugar leaves (a low perigonal bract-to-leaf index); short internode length causes axillary racemes become confluent and coalesce into collective congested colas. Sugar leaves with dense CSGTs on the proximal half, often present beyond the midpoint of the leaflet. Perigonal bract densely covered with CSGTs. Perianth membranous, usually sloughed off, with a fringe of striped or irregularly mottled pigmentation near the base of the fruit. Achene usually ≥ 3.6 mm long, exocarp green to gray; base blunt and lacking well-developed abscission zone.

##### Phytochemistry.

Dried female inflorescences: THC ≥0.3, in late 20^th^ century accessions nearly always >1.0%; literature weighted x¯ = 5.69%, up to 14.5%. This variety expresses the highest total THC%+CBD% (a measure of relative resin content of the plants, since these two cannabinoids usually dominate the resin) of all varieties, which correlates with its dense covering of glandular trichomes. Its THCV%+CBDV% content is lower than South Asian populations; Hillig and Mahlberg (2004) report a mean of 0.14%. Terpenoid profile imparts an acrid or “skunky” aroma, and uniquely expresses sesquiterpene alcohols, such as guaiol, *γ*-eudesmol, *β*-eudesmol, and the monoterpene alcohol nerolidol, as well as hydroxylated terpenoids, such as *γ*-elemene, *a*-terpineol, and *β*-fenchol.

##### Genetics.

Allozyme and DNA studies that segregated Central Asian and South Asian domesticates are detailed in the genetics section of Variety 1. Onofri et al. (2015) identified a SNP in the gene that encodes THCA synthase that was unique in two Afghani accessions and a Moroccan “*hashīsh* landrace” (their SNP accession code no. 1179, A→T transversion). It was not present in 16 other accessions of fiber- and drug-type plants.

##### Other characters.

Generally early maturing, with greater late-season frost tolerance than South Asian domesticates. Late-season cold triggers anthocyanin production in leaves and inflorescences – the sought-after “purple weed.” Achenes are mostly retained on plants, trapped by surrounding parts of the dense infructescence. Plants are more susceptible to gray mold (*Botrytis cinerea*) and powdery mildew (*Golovinomyces cichoracearum*) than South Asian domesticates.

##### Provenance and uses.

Herbarium specimens from the 19^th^-early 20^th^ centuries come from Afghanistan, northwest Pakistan, Turkestan (Uzbekistan, Tajikistan, Kyrgyzstan, Xīnjiāng Region in China), and Iran. These plant were cultivated for sieved *hashīsh* (*nasha*, *charas*) and sometimes for seed oil.

##### Comments.

Vavilov (1926) characterized *afghanica* as “a morphological link between the wild and the cultivated races of hemp.” However, evidence in Vavilov and Bukinich (1929) suggests a domesticated phenotype (argued in Suppl. material 1: SF.6). Small and Cronquist (1976) treated *afghanica* as a domesticate, synonymized under C. sativa subsp. indica var. indica. Small (2018) commented, “The characteristics of indica type marijuana are highly consistent with those of an advanced cultigen. Like modern oilseed cultivars, they are short and compact, an architecture reducing diversion of energy into stem production and increasing harvest index for the desired product (inflorescence). Even the foliage (with very large, wide leaflets) is consistent with the trend described earlier of advanced cultigens often manifesting larger leaves than their wild and more primitive cultivated relatives. When indica type strains are allowed to set seed (they are normally harvested for flowering material) the infructescences are very dense, preventing most of the seeds from falling away and being distributed naturally – another indication of considerable domestication.” The prominent sugar leaves in the inflorescence may be another indication of domestication, as these likely increase photosynthate production very close to the developing flowers and their perigonal bracts.

### Variety 4: Central Asian wild-type

#### Cannabis sativa subsp. indica var. asperrima

Taxon classificationPlantaeRosalesCannabaceae

(Regel) McPartl. & E.Small

F4E77C92-E35C-5EAF-9F2B-5CD4EF3726F2

Figure 6

Cannabis sativa γ asperrima Regel, *Acta Horti Petropolitani* 6 (1): 476, 1879 (Basionym).
≡ C. sativa var. asperrima Regel in Herder, *Acta Horti Petropolitani* 12(1): 34, 1892.
= C. indica var. kafiristanica Vavilov in Vavilov & Bukinich, *Trudy Po Prikladnoi Botanike*, *Genetike i Selektsii* 33 (Suppl.): 381, 1929.
≡ C. sativa subsp. indica var. kafiristanica (Vavilov) Small & Cronquist, *Taxon* 24: 429, 1976.
≡ C. kafiristanica (Vavilov) Chrtek, *Časopis Národního Muzea v Praze*, *Rada Přírodovědna* 150(1–2): 22, 1981.

##### Lectotype.

**Figure 6.**
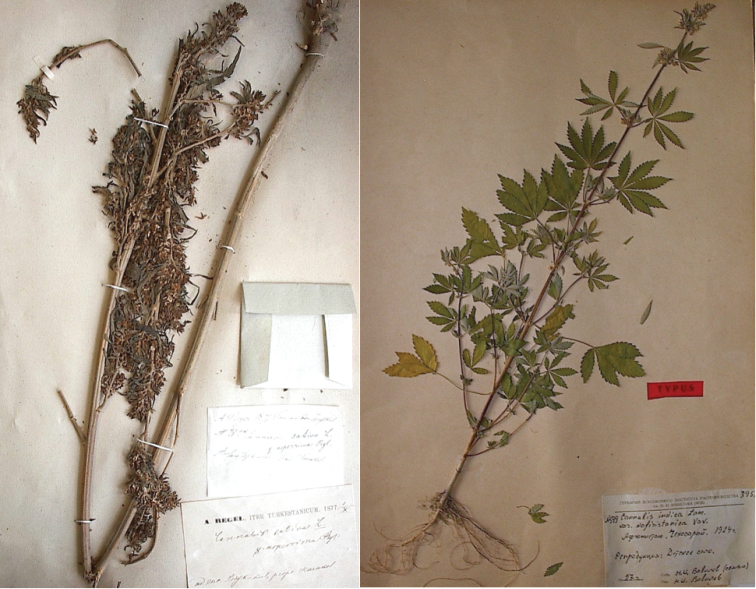
Type specimens of C. sativa subsp. indica var. asperrima. Lectotype on left (**a**), epitype on right (**b**).

Designated herein: Kyrgyzstan, Issyk-Kul Region, near Karakol, leg.: A. Regel; det.: E. Regel, 1.X.1877 (LE). Fig. 6a.

##### Epitype.

Designated herein, explicitly supporting the neotype: Afghanistan, Kunar Province, Chekhosarai (now Asadābād), *Vavilov*, 1924, from seeds sown by Serebriakova in 1927 at Pushkin Experiment Station, Detskoye Selo, St. Petersburg (WIR 599, 3952). Fig. 6b.

##### Diagnosis.

Plants with THC% ≥0.3% in inflorescences and a THC/CBD ratio <7 (almost always >1); central leaflet length:width ratio <6 in fan leaves near the base of inflorescences; mature achenes usually <3.6 mm long, with a persistent perianth and a protuberant base, and readily disarticulating from plant by a well-developed abscission zone.

##### Morphology.

Plants usually < 1.5 m tall. Central stem (stalk) internodes short (often 5–11 cm, shorter in shorter plants), mostly solid, central hollow, if present, usually less than 20% of stalk diameter. Branches in well-developed plants begin close to ground level, at an angle sometimes nearly 90° from the stalk axis, producing a menorah-shaped habitus. Leaf palmately compound, dark green, larger leaves with 5–7 leaflets, sometimes overlapping. Central leaflet relatively short and broad, often oblanceolate in shape; margins with coarse serrations, secondary serrations rarely seen. Female inflorescence small but somewhat compact, with moderately prominent sugar leaves (a moderate perigonal bract-to-leaf index). Sugar leaves with moderately dense CSGTs on the proximal half. Perigonal bract densely covered with CSGTs. Perianth membranous, with dark brown pigmentation in a mottled or sometimes linear pattern; persistent but easily flaked off with manual manipulation. Achene small, oval to elongate, exocarp dark olive colored, with an elongated base.

##### Phytochemistry.

Dried female inflorescences: THC ≥0.3, literature weighted x¯ = 1.49%, range between 0.4% and 4.47%. THC/CBD ratio literature weighted x¯ = 2.23%, range 0.77 to 4.75 (one outlier 9.43). Terpenoid profile likely approximates that of the Central Asian domesticate, but has not been reported in the literature.

##### Provenance and uses.

Herbarium specimens resembling *afghanica*, but with a wild-type phenotype, have provenance from northwestern Pakistan, Afghanistan, Tajikistan, Uzbekistan, Kyrgyzstan, Kazakhstan, and Xīnjiāng Region in China. The mountains in this region are a biodiversity “hotspot,” harboring significant numbers of wild crop relatives, and over 1000 species of endemic plant species (Critical Ecosystem Partnership Fund 2017).

##### Comments.

Herder (1892) retained *C. sativa γ asperrima* as a distinct variety, whereas he synonymized *C. erratica* and *C. sativa β davurica* under *C. sativa*. This taxon’s publication date has priority over Vavilov’s *kafiristanica*, but Vavilov’s specimen is much better preserved, and serves as an epitype.

## Discussion

*Cannabis* populations have undergone both natural and human selection. Fossil pollen studies show that Central and South Asian populations occupied their separate ecological niches for at least 32,600 years (McPartland et al. 2019). Their phenotypes may be presumed to have diverged, due to environmental adaptation and natural selection. Generally, Central Asia has cooler and drier Köppen climates, and shorter growing seasons. South Asia has warmer and wetter Köppen climates, and longer growing seasons (Kottek et al. 2006).

Ecological adaptions to Central and South Asian conditions probably gave rise to habitat isolation, a prezygotic reproduction barrier. Central Asian plants transplanted to South Asian conditions suffer reduced fitness (reproductive success). When their heavily-flowered branches are exposed to monsoonal rainfall, they may snap under the load, because of their brittle, menorah-shaped branching habitus. This does not occur in South Asian plants, whose branches are more flexible, and come off the stalk at more acute angles. The dense, leafy inflorescences of Central Asian plants have poor resistance to fungi that proliferate in high humidity, such as *Botrytis cinerea*. In comparison, the looser, less leafy inflorescences of South Asian plants better tolerate necrotrophic fungi (McPartland et al. 2000). See Suppl. material 1: SF.1 for more examples of prezygotic reproduction barriers.

**Figure 7.**
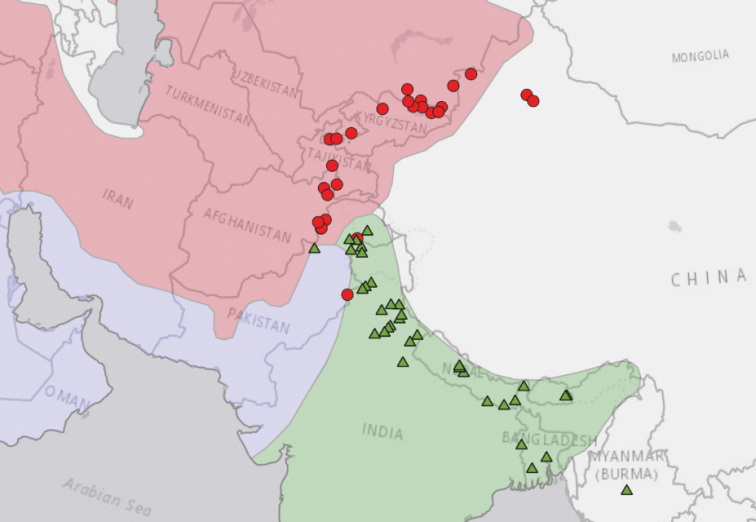
Distribution of herbarium specimens. Red circles: var. asperrima; green triangles: var. himalayensis. Floristic zones based on Djamali et al. (2012): Red area: Irano-Turanian region; green area: Indian region; lilac area: Saharo-Sindian region. Other floristic regions not demarcated and unlabeled. Background base map by Natural Earth, free open-source map data (https:// www.naturalearthdata.com).

We mapped the distribution of herbarium specimens identified as wild-typevar. asperrima and var. himalayensis, using ArcGISPro 2.2 (Fig. 7). The distribution of *himalayensis* and *asperrima* herbarium specimens can be compared to two previous publications that mapped these geographic ranges, by Indian Hemp Drugs Commission (1894) and Breckle and Koch (1982), reproduced in Suppl. material 1: SF.4.

The distributions of *himalayensis* and *asperrima* are parapatric – their ranges do not significantly overlap, but are adjacent to each other. Their interface lies between the Indus River watershed (the northwestern border of var. himalayensis) and the Kunar/Chitral River watershed (the southeastern border of var. asperrima). Parapatry supports our hypothesis of habitat isolation. The distribution of wild-type plants sweeps through an arc of mountains in Central Asia (Hindu Kush, Karakoram, Pamir, and Tian Shan) and in South Asia (Himalaya and Purvanchal Range).

Contrasting climates in Central Asia and South Asia give rise to distinctive flora, and biogeographers assign Central Asia and South Asia to separate floristic regions. Floristic regions are well-defined areas of the world, recognized by their relatively uniform composition of plants species, including endemic flora. The floristic regions mapped in Fig. 7 are based on Djamali et al. (2012). Herbarium specimens of var. asperrima localize in the Irano-Turanian region, whereas herbarium specimens of var. himalayensis localize in the Indian region. Their parapatric interface lies in the Saharo-Sindian region. Outliers in other floristic regions likely represent herbarium specimens of naturalized escapes (formerly domesticated plants that reacquired wild-type traits).

Note that the Indian floristic region by Djamali et al. (2012) was updated and simplified from White and Léonard (1991), who separated peninsular India from the Himalaya range. They, in turn, simplified Takhtajan (1986), who split the Himalaya range into eastern and western provinces, with Kali Gandaki in Nepal at the divide. Takhtajan separated the “Eastern Himalayan Province” due to an influx of flora from China. We hypothesize that this was the route taken by *Cannabis* into the Himalaya, hence into peninsular India. It arrived relatively recently, the oldest fossil pollen in all of South Asia dates back only 32,600 years (McPartland et al. 2019). The morphology of var. himalayensis shares traits with East Asian hemp, such as tall height, relatively hollow shoots with a high percentage of bast fiber and little wood; leaflets with moderately coarse serrations; inflorescences elongated and somewhat loose, with a high perigonal bract-to-leaf index. Himalayan plants and East Asian hemp share similar THC/CBD ratios (Suppl. material 1: Table S11) and terpenoid profiles (Suppl. material 1: Table S15).

Early agriculturalists launched *Cannabis* on its next round of evolution. Floristic regions became “centers of diversity” (CODs), where wild-type plants were domesticated. Vavilov (1935) named eight CODs around the world, and mapped them. He presciently named two separate CODs for *Cannabis indica*: the “Central Asiatic COD,” which corresponds with the Irano-Turanian floristic region, and the “Indian COD,” which corresponds with the Indian floristic region.

Central and South Asian populations diverged further, under different human management regimes (which were also under climatic selection). Central Asians produced sieved *hashīsh*, where bulk processing likely limited the selection of individual high-THC plants (de Meijer 1999). Thus THC/CBD ratios remained close to wild-type. South Asians produced *gañjā*, where plants could be individually harvested, and South Asians selected seeds from choice, high-THC plants, thereby increasing THC/CBD ratios over the course of a millennium (Clarke and Merlin 2013).

South Asian germplasm was carried to Southeast Asia and East Africa by the 13^th^ century, and to Brazil during the African slave trade (Clarke and Merlin 2013). The Central Asian domesticate had a restricted range prior to the 1970s, limited to Afghanistan, Pakistan, and Turkestan. Plants from Turkestan are sometimes classified as South Asian domesticates (Clarke and Merlin 2013; Small 2015), although Clarke (1987) erected C. afghanica var. turkistanica [sic] for Turkestani domesticates. Herbarium collections from the 19^th^ century indicate that cultivated Turkestani plants were Central Asian domesticates, not South Asian domesticates.

The goal of this investigation was to identify “practical and natural” taxa within C. sativa subsp. indica. Our decision to cleave the subspecies into four varieties raises debates regarding nomenclatural priorities, nested hierarchies, and practical applications. We address these issues in Suppl. material 1: SF.13. Our emphasis has been on the domesticates, representing landraces of South Asian heritage (C. sativa subsp. indica var. indica), and Central Asian landraces (C. sativa subsp. indica var. afghanica). Several features tend to differentiate these taxa (Table 1). They are best segregated by their THC/CBD ratios and terpenoid profiles.

**Table 1.** Trends distinguishing the domesticated high-THC varieties C. sativa subsp. indica var. indica and C. sativa subsp. indica var. afghanica.^1^

Character	C. s. var. indica	C. s. var. afghanica
THC/CBD ratio	≥7	<7
THCV+CBDV content	Often present	Often absent
terpenoid profile	“herbal” or “sweet” aroma, with no sesquiterpene alcohols	acrid or “skunky” aroma, with the presence of guaiol, *γ*-eudesmol, and *β*-eudesmol
height, branching	well-grown plants usually ≥ 2 m; branching flexible (with upward-angled habitus)	well-grown plants usually < 2 m; branching inflexible (with menorah-shaped habitus)
leaves at the base of inflorescences	lighter green, usually 7 leaflets, with gaps between leaflet margins	darker green, usually 9 leaflets, with overlapping margins
central leaflets of multifoliolate leaves	long and narrow, lanceolate or linear-lanceolate in shape; margins finely serrate, biserrate margins sometimes seen	long and broad, often oblanceolate in shape; margins coarsely serrate, biserrate margins rarely seen
pistillate inflorescences	relatively diffuse & open, sugar leaves relatively obscure (with a high perigonal bract-to-leaf index)	compact and with prominent sugar leaves (with a low perigonal bract-to-leaf index)
stalked glandular trichome density	few on the proximal end of floral leaves; moderately dense on perigonal bracts	many on the proximal end of floral leaves, extending at least half way down floral leaves; very dense on perigonal bracts
perianth	perianth with mottled pigmentation, sometimes persistent over entire achene	perianth with mottled pigmentation, rarely persistent, limited to base of achene
achene	exocarp color green brown (darker than *afghanica*), lower range of size smaller than *afghanica*; loosely embedded in perigonal bract and sugar leaves	exocarp color olive green to gray (lighter than *indica*), upper range of size larger than *indica*; tightly embedded in perigonal bract and sugar leaves
maturation time	later maturing	earlier maturing
other characters	susceptible to black mildew (*Schiffnerula cannabis*), monoecious plants occasionally seen	susceptible to gray mold (*Botrytis cinerea*) and powdery mildew (*Golovinomyces cichoracearum*), monoecious plants rarely seen

^1^ As emphasized in the text, the differences presented here represent the historical, unhybridized forms of “Indica” and “Sativa” landraces, before extensive recent hybridization between them.

Few trends in Table 1 that distinguish the landraces remain true for “Indica” and “Sativa” strains in commerce today. In particular, THC/CBD ratios have converged in material allegedly representing “Indica” and “Sativa” (Fig. 2). Some recent studies of “Indica” and “Sativa” show reversals from their landrace ancestors. Whereas landraces from Central Asia expressed THC/CBD ratios lower than landraces from South Asia; six recent studies reported the reverse in “Indica” and “Sativa” (Fischedick et al. 2010; Hazekamp and Fischedick 2012; Elzinga et al. 2015; Hazekamp et al. 2016; Lynch et al. 2016; Jikomes and Zoorob 2018). This prompted Hazekamp and Fischedick (2012) to abandon “Indica”/“Sativa” nomenclature, in favor of “chemovars.”

Terpenoid profiles, surprisingly, have largely remained distinct. “Indica” hybrids uniquely express sesquiterpene alcohols, like their Central Asian ancestors. These are absent in South Asian landraces and their “Sativa” descendants (Suppl. material 1: SF.9). Centuries of artificial selection for THC content apparently did not alter sesquiterpene alcohol content. The same may be true for THCV. Limited evidence suggests that THCV, a marker of South Asian landraces and South Asian wild-types (Hillig and Mahlberg 2004), is retained in “Sativa” (Hazekamp and Fischedick 2012; Aizpurua-Olazizolo et al. 2016).

Intermediate forms are often observed between varieties, which are capable of interbreeding and gene exchange under the biological species concept. Where varieties overlap geographically, they frequently generate intermediate forms. Intermediate forms are commonly seen in herbarium specimens from Pakistan, which is the center of diversity for subspecies *indica* – all four varieties occur there. Many herbarium specimens from the Middle East (Turkey, Syria, Lebanon, Palestine, Israel, Jordan, Iraq, western Iran) and north Africa (Egypt to Morocco) also show intermediate phenotypes. Clarke and Merlin (2013) classified Middle Eastern and north African populations as ancestors of South Asian landraces. However, Central Asian germplasm may have reached the Middle East in the 1200s, and again in the 1600s (Suppl. material 1: SF.11).

Several quantitative phenotypic traits await measurement in *Cannabis*, such as glandular trichome density per mm^2^ surface area, glandular trichome size, and gland head abscission. An unambiguous genetic “barcode” differentiating *C. indica* and *C. afghanica* awaits discovery. See “Future directions” in Suppl. material 1: SF.13. Lastly, this study has not addressed East Asian hemp. Cannabinoid and genetic data segregate East Asian *Cannabis* as a subset of the C. indica subsp. indica genepool (Hillig 2005b). See Suppl. material 1: SF.12 for more about East Asian *Cannabis*, particularly regarding biodiversity in Yúnnán.

## Conclusions

The four *Cannabis* varieties circumscribed and named here merit formal recognition. Recognizing infraspecific taxa helps to identify populations vulnerable to extinction (e.g., Ellstrand 2003; Haig et al. 2006). In the wake of the United Nations Biodiversity Convention, infraspecific variation has become a focus for conservation efforts (Coates et al. 2018). Recognizing the four *Cannabis* varieties and their unique morphological and chemical characters also provides “prior art,” thwarting claims of originality in *Cannabis* utility patents.

Collection and conservation of germplasm of indigenous populations of Central and South Asian landraces in their centers of diversity is urgently needed. The germplasm base outside their centers of diversity has become genetically contaminated by widespread crossbreeding. In the context of climate change and unpredictable future needs, in situ conservation of agrobiodiversity is much preferable for crop plants and their wild relatives, but given the precarious continued existence of unaltered aboriginal wild populations of *Cannabis* in Asia, preservation in seed banks is an immediate priority. Hopefully the unambiguous names provided may help prevent extinction of these taxa.

## Supplementary Material

XML Treatment for Cannabis sativa subsp. indica var. indica

XML Treatment for Cannabis sativa subsp. indica var. himalayensis

XML Treatment for Cannabis sativa subsp. indica var. afghanica

XML Treatment for Cannabis sativa subsp. indica var. asperrima

## Supplementary materials

Supplementary material 1 A classification of endangered high-THC cannabis (Cannabis sativa subsp. indica) domesticates and their wild relatives Data type species data Explanation note Description of species concepts, level nominalism, wild-type nominalism, protologues, nomenclatural priority, intermediate forms, and elaboration of methodology. List of taxonomic characters and their respective coding, used in the morphological and total evidence analyses. https://binary.pensoft.net/file/390918This dataset is made available under the Open Database License (http://opendatacommons.org/licenses/odbl/1.0/). The Open Database License (ODbL) is a license agreement intended to allow users to freely share, modify, and use this Dataset while maintaining this same freedom for others, provided that the original source and author(s) are credited.John M. McPartland, Ernest Small
