# Supplementary material for: A classification of endangered high-THC cannabis (Cannabis sativa subsp. indica) domesticates and their wild relatives
Source: PhytoKeys. 2020 Apr 3;144:81–112. doi: 10.3897/phytokeys.144.46700 (PMC7148385; doi:10.3897/phytokeys.144.46700)
Supplement: Supplementary material 1 — A classification of endangered high-THC cannabis (Cannabis sativa subsp. indica) domesticates and their wild relatives [file phytokeys-144-081-s001.pdf]

**A classification of endangered high-THC cannabis (*Cannabis sativa* subsp. *indica*) domesticates and their wild relatives**

John M. McPartland and Ernest Small

## Supplementary File


Introduction section
- SF.1. Species concepts p. 1
- SF.2. Notes on the other subspecies, *C. sativa* subsp. *sativa* p. 6
- SF.3. Level nominalism and wild-type nominalism p. 8

Methods section
- SF.4. Methodology, herbarium studies p. 12
- SF.5. Methodology, literature review p. 14

Results section
- SF.6. Protologues of the four varieties p. 18
- SF.7. Nomenclatural priority: debates over Persoon and Cazzuola p. 23
- SF.8. Morphological comparisons p. 24
- SF.9. Phytochemical comparisons p. 41
- SF.10. Molecular genetic comparisons p. 58

Discussion section
- SF.11. Crossbreeding *indica* and *afghanica* into "Sativa" and "Indica" p. 67
- SF.12. Intermediate forms; East Asian hemp p. 71
- SF.13. Practical applications and future directions p. 74

Representative herbarium specimens p. 76

Reference list for Supplementary File p. 82


Taxonomic abbreviations used in Supplementary File

| | |
|---|---|
| *Cannabis* = | all taxa included in the genus *Cannabis* |
| *C. sativa* = | *Cannabis sativa* subsp. *sativa* var. *sativa* |
| *C. ruderalis* = | *Cannabis sativa* subsp. *sativa* var. *spontanea* |
| *C. indica* = | *Cannabis sativa* subsp. *indica* var. *indica* |
| *C. afghanica* = | *Cannabis sativa* subsp. *indica* var. *afghanica* |
| *C. himalayensis* = | *Cannabis sativa* subsp. *indica* var. *himalayensis* |
| *C. asperrima* = | *Cannabis sativa* subsp. *indica* var. *asperrima* |

### SF.1. Species concepts

At last count, a score of "species concepts" competed for adherents. Four widely accepted species concepts, as they pertain to *Cannabis*, are elaborated below:

The **biological species concept** (BSC) defines a species as a group of interbreeding populations, reproductively isolated from other groups (Mayr 1942). Mayr tested the "species status" of two organisms with a breeding experiment. If they produce fertile offspring, they are the same species. Small (1972) crossbred 38 *Cannabis* accessions in a glasshouse experiment. He included fiber-type plants from Europe, Turkey, China, and Japan; drug-type plants from Mexico, Thailand, Syria, Cyprus, and Europe; and wild-type plants from Germany, Canada, and USA (no *C. afghanica* accessions). All $F_1$ hybrids were interfertile. Small concluded that no sterility barriers existed within the genus, which consisted of one biological species.

Some of Small's hand-pollinated crosses might not naturally hybridize in the field, due to reproductive isolation barriers. Reproductive barriers are expressed along a continuum, as populations diverge from ecotypes, to species, to distinct phylogenetic lineages (Lowry and Gould 2016). The continuum begins with "prezygotic" barriers between populations, which are based on extrinsic mechanisms, and depend on the external environment. At least two prezygotic barriers operate in *Cannabis*:

Temporal (allochronic) isolation arises in the form of separate flowering times. Janischevsky (1924) reported temporal isolation between *C. ruderalis* and neighboring *C. sativa*. Wild-type plants matured in mid-June, while domesticated plants were still in the vegetative stage. Temporal isolation thwarted Bredemann (1952) when he tried crossing German *C. sativa* with South Asian *C. indica.* By the time Indian males produced pollen, German females had already passed their fertility period. When he pollinated Indian females with German pollen, frost killed Indian females in October before setting seed.

Habitat isolation arises in the form of genetic fitness for a specific environment. Habitat isolation can be tested in a transplantation experiment. *C. himalayensis* adapted to the Himalaya (high altitude, cooler) may not survive when transplanted to the plains of India (low-altitude, hotter). *C. indica* adapted to the warm-and-wet plains of India would likely succumb in the Hindu Kush, with its arid climate, desiccating winds, high UV-B radiation, and shorter growing period. *C. afghanica* is poorly adapted to warm-and-wet conditions—the seedlings are susceptible to lethal diseases caused by *Pythium* and *Rhizoctonia* fungi in damp soil. In mature plants, roots suffer waterlogging stress, branches snap under monsoonal rainfall, and flowers perish from "bud rot" caused by *Botrytis cinerea* and *Trichothecium roseum*. Backcross experiments show that intolerance to humidity is expressed in hybrids that contain a small percentage of *C. afghanica* parentage (McPartland *et al.* 2000).

The **diagnostic species concept** (DSC) defines a species as "the smallest group that is consistently and persistently distinct, and distinguishable by ordinary means" (Cronquist 1978). Cronquist applied this concept to *Cannabis* taxonomy (Small and Cronquist 1976). Folk taxonomists employ a DSC concept when they distinguish between "Sativa" and "Indica". Distinguishable features are **taxonomic characters**—attributes of an organism that are divisible into at least two conditions (or states). For example, plant height is a character that distinguishes "Sativa" from "Indica", with two character states: ≥ 2 m for "Sativa", and < 2 m for "Indica".

DSC criteria become unreliable with sibling species (which look alike and may be "lumped"), or highly distinctive varieties (which may be "split" into separate species). Taken to the extreme, botanists in the $16^{th}$-$17^{th}$ centuries split male and female plants into separate species, calling them *Cannabis mas* and *Cannabis foemina* (*e.g.,* Boch 1546). This splitting was based on a single taxonomic character—gender, with two character states—female or male flowers.

Modern DSC-based taxonomists measure as many characters as possible, in as many organisms as possible, and give the characters equal weight. This method is known as "numerical taxonomy" or "phenetic" (as opposed to phylogenetic) taxonomy. Phenetic taxonomists apply multivariate statistics, such as **principal component analysis** (PCA) or **neighbor-joining** (NJ)

methods to discern clusters of organisms, which can be delimited as species or infraspecific groups.

Another DSC method, **canonical analysis** (CA), differs from PCA by pre-defining potential groupings on some criterion. PCA is used for pattern recognition, whereas CA is used for hypothesis testing. CA establishes whether or not a minimal discontinuity of variation exists between groups. Small *et al.* (1976) and Hillig (2005b) pre-defined their accessions as members of *C. sativa, C. indica*, or *C. ruderalis*. Small's CA analysis did not separate *C. ruderalis* as a discrete group, whereas Hillig's CA analysis showed separation.

Many *Cannabis* studies have used NJ, PCA, and CA, which we detail throughout this Supporting Information. See Fig. S1 for a phenetic NJ tree based on the results of this study.

Figure S1. Phenetic NJ tree of *Cannabis*, with putative hybridization events marked by dashed lines

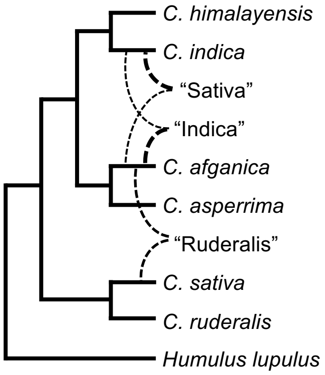


The **phylogenetic species concept** (PSC) defines a species as an *evolutionarily divergent lineage*—the smallest set of organisms that share a character state inherited from a common ancestor. Hennig (1966) differentiated between *ancestral* character states (**plesiomorphies**), and *derived* character states (**apomorphies**). Organisms with deep ancestral roots share plesiomorphies, whereas more recently derived organisms share apomorphies. A shared apomorphy (synapomorphy) is a derived and *unique* character state, present in two groups of organisms *and* their last common ancestor, and is *not* present in earlier ancestors.

Prior to Darwin, botanists intuited "primitive" and "advanced" character states, which often correlated with plesiomorphies and apomorphies. Lamarck's protégé, Augustin de Candolle, first proposed that primitive and advanced character states could be used to organize plant taxonomy. De Candolle (1813) coined the word *taxonomie*, defined as *la théorie des classifications*. Bessey (1915) erected the first avowedly evolutionary system to analyze primitive and advanced character states in plants. He composed a list of evolutionary "trends" that indicate the directionality of character changes. He used "trends" to **polarize** character states—determine which character states were ancestral, and which were derived.

Hennig (1966) polarized character states using "outgroup analysis." He compared a group under study (the ingroup) to its outgroup. For example, when we study *Cannabis* and *Humulus* as the ingroup, *Celtis* can serve as the outgroup. If a character state occurs in the ingroup *and* the

outgroup, it is plesiomorphic—ancestral, shared by distant ancestors. If a character is absent in the outgroup and unique to the ingroup, it is apomorphic.

After polarizing a series of characters, Hennig constructed a **cladogram**—a dichotomously branching diagram that represents a nested hierarchy of ingroups and outgroups. A unique synapomorphy arises at each node, a character state shared by a clade: the common ancestor and its decendants. A simplified example of this process is presented in Fig. S2.

Figure S2. Cladogram of four genera in *Rosales*. Synapomorphies that define each monphyletic group are indicated by circled letters, described in the text.

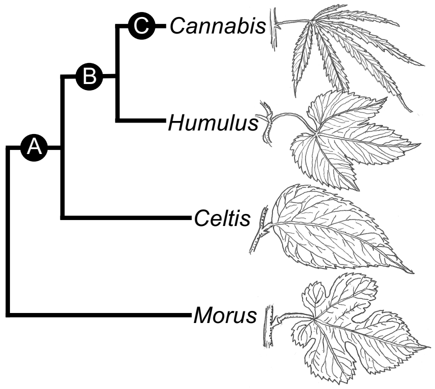


Fig. S2 illustrates three synapomorphies with respect to the direction of leaf evolution:

*Synapomorphy A*: Leaves with free stipules (monophyletic group: *Cannabis, Humulus, Celtis*). The ancestral character state is a stipule that ensheaths the petiole (*Morus*). The stipule is an appendage at the base of the leaf petiole.

*Synapomorphy B*: Leaves with a *craspedodromus* venation pattern, where veins proceed straight into the margin of the leaf (monophyletic group: *Cannabis, Humulus*). The ancestral character state is a *camptodromous* venation pattern, where major veins bend before they get to the margin of the leaf. Bessey (1915) identified craspedodromus venation as a derived state.

*Synapomorphy C*: Leaves that are *compound*, composed of several leaflets (monophyletic group: *Cannabis*). The ancestral character state is *simple* leaves—single leaf blades not divided into discrete leaflets. This was another trend identified by Bessey (1915).

The **ecological species concept** (EcSC) defines an ecotype as a population of organisms adapted to a niche (a set of resources). Van Valen (1976) argued that ecology drives evolution, and selection acts primarily on phenotypes, not genotypes. The ecological niche that a plant population occupies will shape its morphology and phytochemistry. Ecological niches give rise to reproductive isolation barriers, such as the aforementioned habitat isolation between *C. indica* (in warm-and-wet India) and *C. afghanica* (in cool-and-dry Afghanistan).

McEno *et al.* (1991) collated traits of *Cannabis* recorded in the literature, and correlated them with traits adapted to specific biomes (distinct biological communities that have formed in response to a shared physical climate). See Table S1. Based on these correlations, McEno concluded that *Cannabis* evolved in a steppe biome at a temperate latitude, in undulating terrain that was cut by seasonally-flooding streams or rivers, populated by herbivores.

This landscape was literally on the rise in the northeastern Tibetan plateau—the *Cannabis* center of origin (McPartland *et al.* 2019), when *Cannabis* and *Humulus* diverged 27.8 mya (McPartland 2018). China's first steppe communities arose in that region, at the Eocene-Oligocene boundary (Sun *et al.* 2014), and continued to develop through the Oligocene and Miocene (Wang 1996).

In contrast, Zhang *et al.* (2018b) proposed northwestern Yúnnán and adjacent southeastern Tibet as the *Cannabis* center of origin. They estimated that *Cannabis* and *Humulus* diverged 18.2 mya. However, Yúnnán at that time was warmer and wetter than today, and supported subtropical broad-leaved forests (Sun *et al.* 2011, Huang *et al.* 2016). This ecosystem would not drive the evolution of a steppe plant like *Cannabis*, according to the nearest living relative method (Mosbrugger and Utescher 1997).

Table S1. *Cannabis* ecological traits and correlations with biomes, updated from McEno *et al.* (1991).

| ecological trait | correlated biome |
|---|---|
| anemophily (wind pollination), decreased pollen viability with increased relative humidity (Bassani *et al.* 1994) | open habitat with low relative humidity (temperate steppe) |
| wild-type plants with woody, flexible stalks, able to withstanding wind shear (Small *et al.* 2003) | wind-swept habitat (steppe or exposed slopes) |
| sun-loving heliotrope (Darwin 1880), exposure to open sky the most important ecological parameter influencing seed production in wild-type hemp (Haney and Kutscheid 1975) | open habitat with few trees (steppe or exposed slopes) |
| short-day plant that flowers when daylength drops below 12 h/d; photoperiod-insensitive populations can flower under unfavorable daylengths—south of 30ºN (India, Yúnnán) and north of 60ºN (Helsinki, Komi Republic, Siberia) | habitat at mid-latitude, somewhere between 30ºN and 60ºN |
| leaf nitrogen/phosphorus ratio very low: mean ratio 9.4 measured in ten soil types (Coffman and Genter 1975) | low leaf N/P correlates with higher latitude (Kerkoff *et al.* 2005) |
| *Cannabis* utilizes $C_3$ photosynthetic pathway, with a $^{13}C$:$^{14}C$ ratio ($\delta^{13}C$) between -28 and -33‰ (Liu *et al.* 1979). $C_3$ plants have a $\delta^{13}C$ >-21‰, and $C_4$ plants have a $\delta^{13}C$ <-21‰ | $C_3$ plants are favored over $C_4$ plants where mean monthly temperature <22ºC with >25 mm rainfall at current $CO_2$ levels (Ehleringer and Cerling 2001) |
| burning down stands of wild hemp results in increased populations the following year (Badaev and Boltaev 2013). *Cannabis* fossil pollen correlates with fire history (Franklin and Tolonen 2000). Seedlings are somewhat fire dependent, because they are inhibited by shading from old undecomposed material | grasslands are fire-dependent ecosystems, with high biomass, slow decomposition, poor palatability (decreased herbivory)—all of which promote fuel build-up (Bond *et al.* 2005) |
| the "climatic variability" hypothesis suggests that seasonal variability increases at higher latitudes, and selects for species with greater phenotypic plasticity (Molina-Montenegro and Naya 2012) | *Cannabis* exhibits marked phenotypic plasticity (Darwin 1868) so it likely evolved at higher latitudes or in mountainous terrain |
| photosynthetic maximum of 25ºC for fiber-type plants and 25-30º for drug-type plants (Bazzaz *et al.* 1975); cultivated plants grow best between 14-27ºC (Small *et al.* 2003) | habitat in warm-temperate climate |
| wild-type plants are cold tolerant (Janischevsky 1924, Shaikhislamova *et al.* 2006), but cannot tolerate hard frosts (Bócsa and Karus 1997) | habitat in cool-temperate climate |
| wild-type plants are drought resistant (Janischevsky 1924, Kubešova *et al.* 2010) | habitat with seasonal aridity |

| | |
|---|---|
| deep rooting depth (Amaducci *et al.* 2008c), roots of wild-type plant up to 2 m deep in coarse-textured soils (Haney and Kutscheid 1975) | deep roots associated with water-limited ecosystems with high evapotranspiration (Schenk and Jackson 2002) |
| achenes spread by water transport (Basu 1894), survive in water longer than other plants (Ewart 1894) and can float better than most other plant species (Moravcová *et al.* 2010) | "ditchweed" adapted to hydrochory—seed dispersal through running water |
| needs soil disturbance to penetrate established stands of perennial vegetation (Haney and Bazzaz 1970, Yunusbaev *et al.* 2003) | grasslands with soil disturbed by seasonally-flooding streams or rivers, or trampling by large herbivores |
| plants thrive in soils manured by "excrements of wild animals" (Vavilov 1926), seeds can be transported via endozoochory (McPartland and Naraine 2018) | habitat grazed by large herbivores (grasslands) |
| Central Asian flora (all plants) share traits: dense flowering tops with ample glandular trichomes containing terpenoids (Breckle 2007), susceptible to fungal diseases (Vavilov 1940) | habitat in Central Asia |

## SF.2. Notes on the European subspecies, *C. sativa* subsp. *sativa*

*Cannabis sativa* has undergone two rounds of evolution—millions of years (mya) of natural selection, and thousands of years (kya) of human selection. *Cannabis* and *Humulus* diverged from a common ancestor, with a divergence time, based on molecular clock analyses, of 27.8 mya (McPartland 2018), 21 mya (Zerega *et al.* 2005), or 18.2 mya (Zhang *et al.* 2018).

The *Cannabis* center of origin has been appraised by two different methods. Based on fossil pollen data, McPartland *et al.* (2019) proposed the northeastern Tibetan plateau, near Xīníng in Qīnghǎi Province. Based on haplotype data of extant plants, Zhang *et al.* (2018) offered the southeastern Tibetan plateau, in southeastern Tibet and adjacent northwestern Yúnnán.

*C. sativa* reached Europe by at least 6.3 mya (McPartland *et al.* 2018). Pleistocene glaciations, increasing in amplitude 1 mya–800 kya (Ehlers and Gibbard 2007), likely drove vicariant divergence, splitting the European and Asian populations into discontinuous distributions. The two populations diverged due to genetic drift and environment-specific adaptations. The molecular clock analysis by McPartland (2018) estimated that *C. sativa* and *C. indica* diverged 1.05 mya, although the estimate was not robust, because the taxa differed at only one nucleotide site. Zhang *et al.* (2018) estimated the *Cannabis* crown age (divergence of haplogroups) was 2.24 mya.

Botanists have long noted that European *C. sativa* existed in two phases of domestication—wild and cultivated—but did not assign Latin names to the wild-type until the 20th century. Herodotus (2007) reported that *κάνναβις* grew both wild and cultivated in Scythia, now Ukraine, in 440 BC. Lamarck (1785) said that *C. sativa* grew *presque naturalisée* in Europe, and *croît naturellement* in Persia.

European wild-types were assigned to the taxa *C. sativa* var. *spontanea* Vavilov (1922) and *C. sativa* var. *ruderalis* Janischevsky (1924). Janischevsky also coined an alternative species rank, *C. ruderalis,* adding the caveat, "I am inclined to consider it a well-marked variety." The taxa by Vavilov and Janischevsky were based on the same population of wild-type plants growing near Saratov, Russia. At the time, both Vavilov and Janischevsky worked at Saratov

University. They botanized on field trips together along the Volga River (Korotkova 1978). Vavilov squeezed out a publication about wild hemp before Janischevsky finished his own research. Janischevsky politely mentions Vavilov's research, but always uses *ruderalis* (Janischevsky 1924, 1925). Vavilov politely mentions Janischevsky's work, but always uses *spontanea* (Vavilov 1926, 1931, Vavilov and Bukinich 1929).

The plants described by Vavilov and Janischevsky (1924) could have been wild, ruderal, naturalized, or spontaneous. See our discussion of "wild-type nominalism" below (SF.3b). Indeed, 150 years prior to Vavilov and Janischevsky, botanists encountered the same population of wild-type plants, 10 km downriver of Saratov, and debated whether they were truly wild, or escapes of formerly cultivated plants (Lepechin 1774, Pallas 1793). Full protologues of the taxa by Vavilov and Janischevsky are detailed elsewhere (McPartland and Guy 2017).

A troika of Soviet texts, team-edited by intersecting authors, chose *C. ruderalis* Janischevsky over *C. sativa* var. *spontanea* Vavilov (Nekrasova 1934, Yarmolenko 1936, Mal'tsev 1939). At that time, Soviet science writhed under T. D. Lysenko, the pet scientist of Joseph Stalin. Lysenko labeled Vavilov a Trotskyite, which led to Vavilov's arrest (Medvedev 1969). Despite political correctness—recognizing Janischevsky's taxon over Vavilov's taxon—Nekrasova and Mal'tsev spent time in prison.

After Vavilov was arrested, his assistant Tatiana Ya. Serebriakova coauthored her final *Cannabis* publication with Ivan A. Sizov. He was a Lysenkoite who "began energetically to liquidate the remnants of Vavilov traditions" (Medvedev 1969). Serebriakova and Sizov (1940) elevated Vavilov's taxon from a variety to a subspecies, but without his name in the basionym: *C. sativa* subsp. *spontanea* Serebriakova. *C. ruderalis* was synonymized under that taxon.

Schultes *et al.* (1974) treated *C. ruderalis* as a species separate from *C. sativa*. They erroneously typified it with a specimen from Central Asia (Tajikistan), not Europe. Furthermore, they described *C. ruderalis* as a very short plant with broad leaflets. To us, this morphology is not consistent with Janischevsky's taxon, but consistent with *C. asperrima*.

Small and Cronquist (1976) recognized the wild-type as a variety, *C. sativa* subsp. *sativa* var. *spontanea* Vavilov. They chose Vavilov's taxon over Janischevsky's taxon on the basis of priority, having been published two years previously.

Other authors accepted Schultes's concept, and recognized *C. ruderalis* as a short, broad-leafleted species from Central Asia (Anderson 1988, Hillig and Mahlberg 2004, Clarke and Merlin 2013). Some consider *C. ruderalis* the ancestor of *C. sativa.* However, according to phylogenetic taxonomists, an extant species cannot be the direct ancestor of another extant species (*e.g.,* extant *Canis lupus* is not the direct ancestor of *Canis familiaris,* Pennisi 2013). Subspecies arising through anagenesis (change within the same evolutionary line) may coexist, exampled by *Zea mays* subsp. *parviglumis* (wild-type teosinte) and *Zea mays* subsp. *mays* (domesticated maize).

"Ruderalis" has become a mainstay of today's vernacular taxonomy. "Ruderalis" is applied to plants that exhibit one to three characteristics: CBD≃THC, wild-type morphology, or early flowering. The latter characteristic is also called "autoflowering," that is, day-neutral, flowering

not induced by the light cycle. Hoffman (1961) first reported autoflowering in an accession from Finland. One of the first seed catalogs for drug-type strains illustrated “Ruderalis” plants growing near the Hungary-Ukraine border (Schoenmakers 1986). The plants showed strong apical dominance and little branching. These traits are consistent with a spontaneous escape of cultivated hemp, and depart from traits described by Janischevsky. Schoenmakers (1986) also sold “Shady Lady”, a cross between “Ruderalis” (25%) and “Afghani” (75%). In a genomic study, Grassa *et al.* (2018) demonstrated that high-CBD strains contain “hemp-type” CBDA synthase genes introgressed “into a backround of marijuana-type cannabis.” This sounds like Schoenmakers’s description of “Shady Lady”.

Nearly everyone recognizes European and Asian *Cannabis* populations as segregates. Issues arise over their taxonomic ranks: separate species? Or separate subspecies?

## SF.3a. Level nominalism

“Level nominalism” argues that we cannot tell where to draw a line through degrees of taxonomic distinction (Hey 2001). In other words, we cannot assign a specific taxonomic rank to the separate European and Asian populations. Debates over level nominalism became embroiled in the USA legal system in the 1970s. Botanists on behalf of the defense argued that narcotics laws cited *C. sativa,* whereas the accused possessed a different species, *C. indica,* which was statutorily overlooked and technically legal (Schultes *et al.* 1974). Taxonomists on behalf of plaintiffs argued for a single species, *C. sativa,* and therefore *C. sativa* and *C. indica* were the same legal entity (Small and Cronquist 1976).

Two centuries prior, however, debates over level nominalism erupted between Linnaeus and Buffon. Buffon (1749) argued that “Nature proceeds by unknown gradations, and, consequently, it is impossible to rely entirely on those divisions, since she passes from one species to another species, and often from one genus to another genus, by imperceptible nuances.” In contrast, Linnaeus saw sharp-cut delineations between species. He treated species as typological and immutable entities with fixed forms. “We count as many species as there were forms created in the beginning” (Linnaeus 1751).

Buffon’s protégé, Lamarck, also argued against the fixity of species. This may have influenced him to coin *C. indica*. Lamarck (1809) proposed a theory of evolution, wherein he stated that species merged into other species without clear demarcations. Similarly, Darwin could not reconcile the continuous process of evolution with the discrete concept of species. “I was much struck how entirely vague and arbitrary is the distinction between species and varieties” (Darwin 1859). He believed that species were arbitrary constructs. “I look at the term species as one arbitrarily given for the sake of convenience to a set of individuals closely resembling each other.”

The debate over taxonomic rank—whether Lamarck’s *C. indica* was a species, versus a subspecies of *C. sativa*, began with Willdenow (1805). He treated Lamarck’s *C. indica* as a full synonym of *C. sativa*. He did not reduce it to an infraspecific taxon. Willdenow argued that no diagnostic differences existed between them, because both taxa showed alternate branching

(Lamarck erroneously claimed that *C. indica* uniquely had alternate branching). Willdenow ignored Lamarck's seven other morphological differences between *C. indica* and *C. sativa*. He also ignored Lamarck's phytochemical differences, possibly because Linnaeus (1751) rejected chemotaxonomic characters, such as fragrance and taste.

McPartland and Guy (2017) found evidence of cultural bias influencing Willdenow's taxonomic decisions, arising from personality cults surrounding Linnaeus and Lamarck. Today it is difficult to fathom Linnaeus's renown, and the enmity provoked by Lamarck's deviation from Linnaean orthodoxy. Willdenow was a disciple of Linnaeus, and updated *Species Plantarum* after Linnaeus died. Willdenow (1805) rejected over half of Lamarck's new taxa (most have been reinstated by modern taxonomists).

British botanists in India were tutored by Johann König, a student of Linnaeus. They also treated *C. indica* as a full synonym of *C. sativa*, without reducing it to an infraspecific taxon. William Roxburgh became the most influential member of König's "United Bretheren" in India. Roxburgh (1832) wrote, "I perfectly agree with Willdenow in thinking all the varieties [of *Cannabis*], if even such they can be called, centre in one species."

Two centuries of arbitrary decisions followed (reviewed in McPartland and Guy 2017). Although the "species debate" continutes to bedevil *Cannabis* taxonomy, recent genetic studies—free of cultural bias—support a single-species concept. These studies are abstracted in the main manuscript (Mandolino *et al.* 2002, Gilmore *et al.* 2007, Sawler *et al.* 2015, Lynch *et al.* 2016, Grassa *et al.* 2018, McPartland 2018). Collectively, these genetic studies mitigate level nominalism, and support the segregation of *C. sativa* and *C. indica* at an infraspecific rank (below that of species).

Stuessy (2009) made recommendations regarding the infraspecific ranks of subspecies, variety, and form. He listed characteristics that distinguish them (Table S2). Stuessy stressed nomenclatural stability: if subspecies and/or variety has already been used to describe infraspecific patterns of variation within a species, then this precedent should be followed insofar as possible. Other botanists have cited historical precedence regarding their use of infraspecific ranks (*e.g.,* Spongberg 1979, Jansen 1985, Graham 1988, Holmgren 1994). Raven (1974) railed against authors who did not follow historical precedent, leading to "the highly undesirable effect of proliferating new combinations." Our treatment of *Cannabis* as a single species, with a nested hierarchy of two infraspecific ranks (subspecies and variety) follows precedent set by Small and Cronquist (1976).

Table S2. Characters useful for distinguishing subspecies, varieties, and forms (adapted from Stuessy 2009)

| Characteristic | Subspecies | Variety | Form |
|---|---|---|---|
| Morphological distinctions | several conspicuous differences | one to a few conspicuous differences | usually a single conspicuous difference |
| Geographical patterns | coehesive, largely allopatric or peripatric | coehesive, largely allopatric with some overlap | sporadic, sympatric |

| | | | |
|---|---|---|---|
| Genetic divergence | usually markedly multigenic | multigenic or with some simple control | simple control (usually single gene) |
| Likelihood of natural hybridization | possible along contact zones | probable in overlap region | always expected |
| Fertility of hybrids | markedly reduced fertility | reduced fertility | complete fertility |

## SF.3b. Wild-type nominalism

Zinger (1898) first described the wild-type phenotype in *Cannabis*, in an accession he called *Cannabis himalayana*. The characters included small achene size, a protuberant-and-tapered base with a prominent abscission zone, and a persistent perianth (Fig. S3). The perianth exhibited camouflagic coloring, with irregular dark spots. Botanists since Zinger have referred to the coloring as a mosaic, marbling, or mottling. The abscission zone has been called a horseshoe, circular torus, hilum, and callus-ring; the adjacent attenuated region has been termed a caruncle, basal constricted zone, or elaiosome.

The perianth's dark spots consist of pigmented cells, which contrast with non-pigment-containing cells. Pigmented cells differ in shape from non-pigmented cells (Briosi and Tognini 1894). Under a scanning electron microscope, pigmented cells are relatively straight and parallel to each other, compared to non-pigmented cells, which show a wavy pattern, and it appears that the pigment is associated with vascular cells (Small 1975).

According to Hillig (2005b), WLD biotypes (*i.e.,* Central Asian landraces) tend to express pigmented cells in linear stripes, whereas NLD biotypes (*i.e.,* South Asian landraces) express pigmented cells in irregular mottling. This character may not have taxonomic relevance; Crescini (1490) showed pattern variations within a single landrace, 'Carmagnola' from Italy (Fig. S3).

Figure S3. Domesticated and wild-type phenotypes. Image on left from Zinger (1898), who illustrated the achene of a domesticated plant ("7") and a wild-type plant ("8"). Image on right by Crescini (1940), showing variation in perianth ornamentation in four populations of the 'Carmagnola' landrace in Italy.

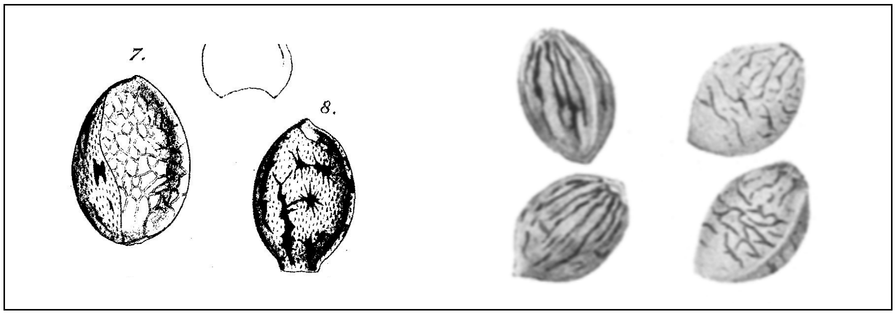


Wild-type plants were studied by Janischevsky (1924, 1925) and Vavilov (1922, 1926). They were unaware of Zinger's work, yet describe identical wild-type characters—small achenes, with a protuberant base and a broad abscission scar, and a persistent perianth. Janischevsky and Vavilov extended their descriptions to the whole plant: wild-type plants were shorter than domesticated plants, usually 0.7-1.1 m in height—although plants grown in well-irrigated garden

soil grew 2.1 m tall. They were branchier than domesticated plants, and the leaves were smaller. Janischevsky also described growth parameters: wild-type plants matured earlier than domesticated plants; they were more drought tolerant, and tolerated shade better. Achenes shattered from plants, and their germination was slow and uneven.

Small and Cronquist (1976) segregated domesticated and wild-type plants as different varieties. They did not, however, treat extant wild-type populations as the ancestors of domesticated varieties. They expressed "wild-type nominalism," because these posited ancestral-descendant relationships cannot be verified.

Domesticated *Cannabis* easily escapes cultivation and goes "feral." *Cannabis*, like cats, is barely domesticated. As a crop plant, the species is rather unique in this regard—relatively few crops can thrive outside of cultivation—rye, oats, wheat, rice, sorghum, oilseed rape, beet, radish, sunflower, and olives (Gressel 2005). Domesticated *C. sativa* reverted to a wild-type phenotype in Canada just 50 generations (years) after cultivation was prohibited (Small 1975).

This rapid phenotypic evolution makes it difficult to distinguish truly wild plants from formerly cultivated plants that have reverted to wild-type phenotypes. Small (1984) delineated a spectrum of plants that exhibit wild-type traits, ranging from truly wild (*i.e.,* native, indigenous, aboriginal), ruderal (either wild or weedy), naturalized (*i.e.,* weedy), to spontaneous (escapes of cultivated plants).

Domestication and the loss of wild-type traits is a product of human selection, which began unconsciously (selecting plants with seeds that did not shatter), followed by goal-directed breeding. Some botanists argue that plants with traits created by human selection should not be assigned taxa under the *International Code of Nomenclature for Algae, Fungi, and Plants* (*ICN*, Turland 2018), but rather be assigned cultivar status under the *International Code of Nomenclature for Cultivated Plants* (*ICNCP*, Brickell 2016). However, for pragmatic reasons, botanists use the *ICN* framework to assign taxa to artificially selected plants (*e.g.,* Hammer and Gladis 2014).

Small and Cronquist (1976) proposed an achene length of 3.8 mm as the threshold between larger domesticated varieties and smaller wild-type varieties. For Asian plants, the threshold should be reduced to 3.6 mm. Achenes recovered from Asian archaeological sites—cultivated if not domesticated plants—were sometimes even smaller than 3.6 mm (Table S3).

Table S3. Morphology of *Cannabis* achenes in Asian archaeological contexts, listed in chronological order.

| context | length (mm) | other morphological characters | reference |
|---|---|---|---|
| 8000 BC; Jōmon culture; Tateyama, Japan; possibly wild-harvested | 3.6 | oval in outline, side keel, surface carbonized, no elongated tapered base | Kobayashi *et al.* 2008 |
| 5800 BC; Péilǐgāng culture, Hénán Province, China | 3.6 | ovoid, surface carbonized, no elongated tapered base | Bestel *et al.* 2018 |
| 3000 BC; Jōmon culture; Fukui Prefecture, Japan | 3.0-3.5 | none available | Kasahara 1987 |
| 3000 BC; Yǎngsháo culture; Yánggùà, China | 5.1 | ovoid, surface carbonized, no elongated tapered base | Zhou *et al.* 2011 |

| | | | |
|---|---|---|---|
| 2600-2500 BC; early Harappan culture; Kunal, Haryana, India | 5.0 | ovoid, surface carbonized, with short tapered base | Saraswat and Pokharia 2003 |
| 2500-1500 BC; Hetapatti, Ganges River basin, India | 3.6* | oval, surface carbonized, no elongated tapered base | Pokharia *et al.* 2017 |
| 1800 BC, Qíjiā culture; Dìngxī, China | 3.7* | ovoid-elongate, surface carbonized, no elongated tapered base | Jia *et al.* 2012 |
| 2200-1600 BC; Lower Xiàjiādiàn culture; Chìfēng, China | 3.0-4.0 | round-ovoid, surface carbonized, no elongated tapered base | Zhao 2011 |
| 1820-1460 BC; Lower Xiàjiādiàn culture; Chìfēng, China | 2.6* | round-ovoid, surface carbonized, slightly elongated tapered base | Jia *et al.* 2016 |
| 1300-600 BC; Senuwar, India; possibly wild-type | 2.2-2.7 | round-ovoid, surface carbonized, slightly elongated tapered base | Saraswat 2004 |
| 766-416 cal. BC; Yánghǎi, Turfan, China | 2.2-3.6 | ovoid with side keel, adherant perianth, abscission scar ringed by lumpy outgrowths but no elongated tapered base | Jiang *et al.* 2006, Russo *et al.* 2008 |
| 800 cal. BC; Yánghǎi, Turfan, China | 3.0 | ovoid, reticulate venation with retained perianth, no elongated tapered base | Jiang *et al.* 2007 |
| 800-520 cal. BC; Jiāyī, Turfan, China | 2.3-2.7 | ovoid with side keel, reticulate venation with retained perianth, abscission scar ringed by lumpy outgrowths but no elongated tapered base | Jiang *et al.* 2016 |

*sizes estimated from photographs with scale bars.

## SF.4. Methodology, herbarium studies

Fifteen herbaria were consulted, designated by herbarium acronyms in *Index Herbariorum*: B (Berlin), BM (British Museum, London), BPI (Beltsville, MD), CUP (Cornell University), F (Field Museum, Chicago), ECON (Economic botany, Harvard), GH (Gray, Harvard), IND (Bloomington, IN), K (Kew, London), LE ("Leningrad," St. Petersburg), LINN (Linnaeus, London), NY (Bronx, New York), P (Paris), PH (Philadelphia), US (Smithsonian, Washington DC), WIR (Vavilov Institute, St. Petersburg).

Plant height cannot be measured from herbarium specimens, unless plants are very short and can fit on an herbarium sheet (43 x 29 cm), often bent in half. Thus plant height and internode length were obtained from the literature (primarily Hillig 2005b). Branching habitus included two characters: branch angle and laxity. Branch angle or divarication measured the angle, in degrees, that a branch came off the vertical shoot; it generally ranged between 45º to 90º from vertical.

Previous authors noted that Chinese hemp (*Cannabis chinensis*) expressed a wider branch angle than European hemp (Koch 1854, De Beaux 1875). Branch angle may be a function of internode length: the long internodes of "Sativa" allow development towards overhead sunlight, while the short internodes of "Indica" force the branches to grow laterally.

Branch laxity is a qualitative measure of flexibility, the ability of a branch to bend or droop. Compare branch laxity in weeping willow (*Salix babylonica*) to that in Scots pine (*Pinus*

*sylvestris*). Laxity likely reflects the ratio of bast fiber (flexible) to wood fiber (inflexible). Sharma (1979b) stated that Himalayan plants were more laxly branched than plants from the Indian plains. Hillig (2005) also observed this, adding that branching in Afghanistan plants was the least lax. Previous authors noted that branches were more lax in Chinese hemp than European hemp (Vilmorin 1851, Blen 1852, Hamm 1854)

Describing color is subjective, despite a precise nomenclature (Stern 1983). Many authors rely on a Crayola scheme. Leaf color ranged from fern (a saturated forest green) to mint (soft light green). The hue, chroma, and lightness of the achene exocarp, covered by a pigmented perianth, is particularly hard to discern. The perianth must be scraped away. Achenes varied from tan (a pale tone of brown), olive (a dark yellow-green), to artichoke (a light gray-green).

Measures of leaflet shape (L/W and WP/L ratios) were adopted from Anderson (1980). Leaflet shape was measured at the base of pistillate inflorescences whenever possible. The perigonal bract-to-leaf index was adopted from the "calyx-to-leaf ratio" (Clarke 1981). Comparing glandular trichome density on proximal versus distal areas of "sugar leaves" was adopted from Potter (2009).

Achene morphology and dimensions were obtained from small sample sizes, usually n = 5–10, to minimize herbarium specimen dissections. The size of the achene's abscission mechanism (the elongated base and abscission scar) was qualitatively scaled from little-to-much elongation. The degree of perianth persistence was qualitatively scaled from none, through little (a trace at the achene base), to complete. Perianth pigmentation was described as mottled (irregular patches of pigment) or striped (somewhat linear streaks of pigment).

In addition to specimens examined in 15 herbaria, we examined digitized images of specimens in the Chinese Virtual Herbarium (www.cvh.ac.cn). The CVH database currently has 2009 *C. sativa* specimens in its database. Most of them have been scanned as digital images. CVH shortcomings include poorly characterized location records (*e.g.,* "Beijing botanical garden" of unstated provenance), misidentifications (*e.g., Humulus* spp. as well as non-Cannabaceae plants identified as *Cannabis*), and poor quality, out-of-focus scans. A majority of specimens are immature or male plants, and some specimens have been defoliated by storage beetles. Only 99 accessions were collected in Xīnjiāng, a critical region with few specimens in Western herbaria. The source for most of these accessions was the Xīnjiāng Institute of Ecology and Geography (herb. XJBI), and they did not provide scanned images. As a result, only 27 of the 99 accessions from Xīnjiāng have digital images.

Herbarium specimens enabled us to map the range of two wild-type populations of *C. sativa* subsp. *indica* (Fig. 7 in main document). The wild-type populations are assigned to varieties *himalayensis* and *asperrima.* The distribution of *himalayensis* and *asperrima* herbarium specimens can be compared to two previous publications that mapped these geographic ranges (Fig. S4). Indian Hemp Drugs Commission (1894) mapped the range of *himalayensis*, based on dozens of field reports (Fig. S4, left). The dark hatchmarked area demarcated "where the hemp plant grows wild," and the lighter hatchmarked areas indicated "where it might grow wild under favourable circumstances." Breckle and Koch (1982) mapped "wildform des Rauschhanfes"

(wild form of drug hemp). The map shows locations of herbarium specimens, as black dots, and the hatchmarked area, “approximate distribution” (Fig. S4, right).

Figure S4. Geographic range of wild-type *C. indica.* Left: map by the Indian Hemp Drugs Commission (1894). Right: map by Breckle and Koch (1982), reproduced with kind permission.

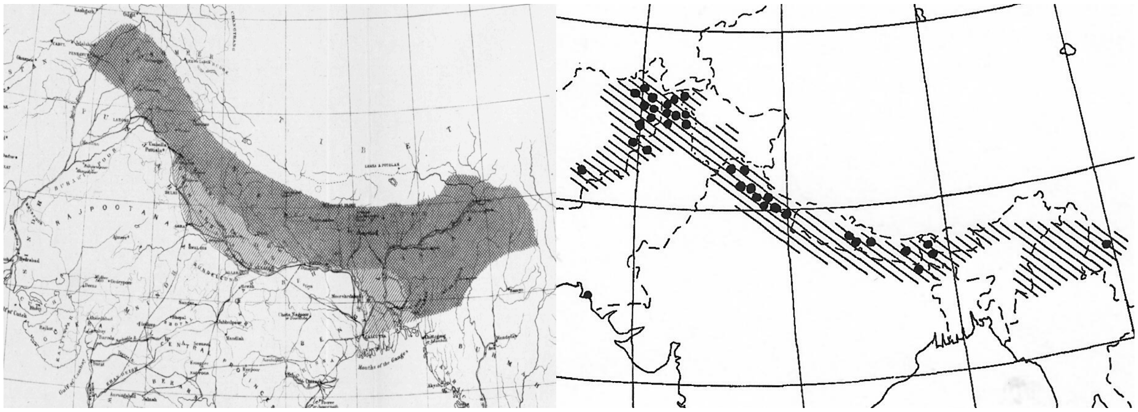

We transferred the IHDC’s distribution map to a modern map, using the georeferencing tool in ArcGISPro 2.2. To this we added the locations of *C. asperrima* and C. *himalayensis* herbarium specimens (from Fig. 7 in the main document). As illustrated in Fig. S5, the IHDC’s distribution map largely corresponds with the distribution of *C. himalayensis* herbarium specimens. The distribution mapped by Breckle and Koch (1982) included both *C. asperrima* and C. *himalayensis.*

Figure S5. The geographic range of wild-type *C. indica,* mapped by the Indian Hemp Drugs Commission (1894), transferred to a modern map. Red circles: locations of herbarium specimens corresponding to the Central Asian wild-type. Green triangles: locations of herbarium specimens corresponding to the South Asian wild-type.

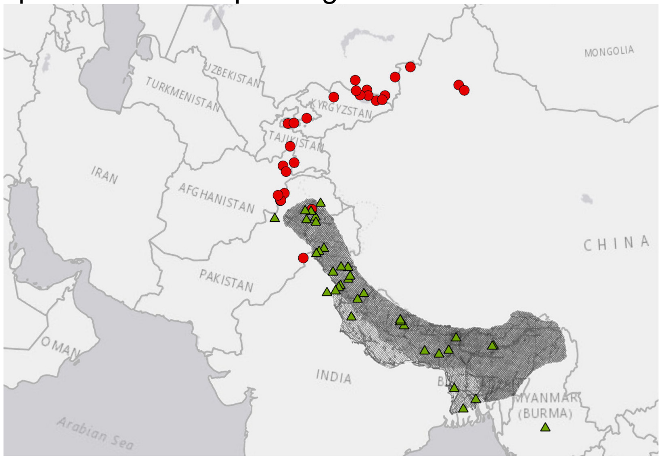


## SF.5. Methodology; Literature review

Studies based upon common garden experiments (CGEs) were given greater weight than studies that compared *Cannabis* or cannabis products from various dissimilar locations (VDLs).

In a CGE, plants are grown in a single location, under identical environmental conditions, and uniformly processed. CGEs aim to reduce environmental variables, and measure differences that reflect true genetic variation. Nevertheless, CGEs still have three sources of variance:

1. The provenance of germplasm cannot be known for certain—especially seed stock obtained from seized material or botanical gardens. For example, three accessions of "*C. indica*" obtained from botanical gardens by Small and Beckstead (1973) contained no measurable THC.

2. Tropical landraces may not reach maturity in CGE studies conducted outdoors at temperate latitudes. Frost kills them first. Achene morphology cannot be observed (unless samples of the achenes used for planting were retained), and cannabinoid levels are not the same as fully mature plants. This methodological shortcoming was noted by authors of CGEs conducted at 51º30' (Fairbairn and Liebmann 1974, Baker *et al.* 1982, Taylor *et al.* 1983), 48º17' (Fournier 1981), 45º12' (Small and Beckstead 1973), and even 35º21' (Turner *et al.* 1979).

Cannabinoid content should be measured in plants at a uniform stage of maturity. In Canada, by law, female inflorescences must be sampled "when the first seeds of 50% of the plants are resistant to compression" (Small 2017). Diverse definitions of "uniform maturity" have plagued the testing of registered hemp cultivars. Callaway (2008) reports THC levels in 'Finola' varying from 0.05 to 0.32% in plants sampled according to different definitions. Most CME studies harvested all accessions simultaneously, so they measured plants at different stages of maturity.

3. CGE studies are biased by a latitudinal "regression to the mean." Plants of northern provenance grown in southern latitudes will flower earlier, with shortened growth (Barbieri 1952, Yao *et al.* 2007, Cosentino *et al.* 2012). Plants of southern provenance grown in northern latitudes will flower later, with increased growth (de Meijer and Keizer 1996, Amaducci *et al.* 2008a, Hu *et al.* 2012, Salentijn *et al.* 2015).

VDL studies are valuable, but with inherent flaws. First and foremost, plants in VDL studies were cultivated under a range of environmental conditions. *Cannabis* responds to dissimilar environments with robust phenotypic plasticity. Darwin (1868) marvelled over the phenotypic plasticity displayed by *Cannabis*. Stevens (1878) summarized the shortfalls of making VDL comparisons, "Hemp, or *Cannabis sativa*, being a plant of rapid growth, sucks up much of the unaltered soil, and therefore differs greatly according to the soil as well as the climate and culture."

VDL studies are diminished by five additional uncontrolled sources of variance: 1. the place-of-origin of samples cannot be known for certain; 2. studies of resin (*hashīsh* or *charas*) vary by the method of production—sieved or rubbed; 3. resin potency varies by degree of adulteration—resin is easier to adulterate than herbal cannabis; 4. losses in cannabinoids and terpenoids arise from dissimilar storage and shipment conditions; 5. losses in cannabinoids and terpenoids vary due to methods in harvesting, drying, curing, and packaging.

Data from CMEs and VDLs can be supported by depositing voucher specimens in herbaria. Vouchers are critical for authenticating the identification of a specimen. They allow other researchers to retrospectively analyse accessions, especially in light of new taxonomic concepts (Culley 2013). Unfortunately, few researchers have prepared herbarium vouchers (*e.g.,* Small

and Beckstead 1973, Schultes *et al.* 1974, Turner *et al.* 1973, 1979, McPartland and Cubeta 1997, Hillig and Mahlberg 2004). Many accessions studied by de Meijer (1994) and Gilmore *et al.* (2007) were shared with Hillig, who prepared herbarium vouchers of those accessions.

Cannabinoids and terpenoids are the phytochemicals featured in our literature review, as explicated in the main manuscript. When comparing cannabinoid content, CGE studies are far more valid than VDL studies. Cannabinoid content is modulated by environmental factors, and CGE studies reduce these environmental variables.

We know of no other phytochemicals that provide useful taxonomic characters for discerning segregates within *C. sativa* subsp. *indica*. Flavonoids might have potential for distinguishing *C. sativa* subsp. *indica* from *C. sativa* subsp. *sativa* (Clark and Bohm 1979, Vanhoenacker *et al.* 2002). The same can be said for spiroindans and stilbenoids (Flores-Sanchez and Verpoorte 2008).

Phytochemicals can be differentiated by smell, taste, their effects upon our physiology, and of course by analytical instrumentation. Larmarck (1785) used smell to differentiate *C. sativa* from *C. indica*; the latter produced a strong odor “resembling somewhat that of tobacco.” He also mentioned the intoxicating properties of *C. indica,* “The principal effect of this plant consists of going to the head, disrupting the brain, where it produces a sort of drunkenness that makes one forget one’s sorrows, and produces a strong gaiety.”

Using analytical instrumentation for plant classification began much later, and its use engendered debate. Small and Cronquist (1976) used gas-liquid chromatography–flame ionization detection (GC–FIDS) to differentiate between *C. sativa* subsp. *sativa* and *C. sativa* subsp. *indica*. Emboden (1977) and de Meijer (1999) criticized “ability to intoxicate” as a taxonomic character. Emboden also criticized the impracticality of any taxonomic scheme that required expensive chromatography equipment.

In the two centuries between Larmarck (1785) and Small and Cronquist (1976), a wide variety of assays were used to gauge cannabis “potency.” A minority of researchers made comparisons based on physiological effects in personal bioassays (*e.g.,* Bergius 1785, de Courtive 1848). Animal assays came into vogue. O’Shaughnessy (1839) tested a wide variety of animals (monkeys, cattle, cats, birds, fish), but got repeatable results with dogs.

USA pharmaceutical companies used the “dog ataxia test,” beginning with Parke-Davis & Co. (Houghton 1897), followed by Nelson, Baker & Co.; Eli Lilly; Smith, Kline & French; and H. K. Mulford & Co**.** The ninth edition of the *United States Pharmacopœia* made physiological dog testing *compulsory*—cannabis was the only drug with a mandatory requirement (United States Pharmacopeial Convention 1916). Walter Siegfried Loewe standardized the dog ataxia test to the theoretical maximum. To overcome intra-individual variabilities in cannabis testing, he invented “bioassay by approximation” (Loewe 1939). Loewe (1944) compared cannabis extract potency relative to the potency of synthetic THC and CBD.

Marshall and Winger (1911) wrote about the disadvantages of testing cannabis on dogs, “A greater degree of accuracy can be obtained by experimenting on oneself.” Mukhopadhyay *et al.*

(1943) compared the dog ataxia test to "judgement by veteran smokers." The dog ataxia test has continued to provide useful information, even after direct testing with $CB_1$ receptors rendered the test obsolete (Lichtman *et al.* 1998). Rodent studies in the cannabinoid field were inaugurated by Wiechowski (1927). Loewe (1946) tested rodent "cataleptic response," and later the "tail-flick test" (Loewe 1950). These became components of Billy Martin's "cannabimimetic tetrad test" (Little *et al.* 1988).

Another metric for estimating potency was "percent resin." Procter (1864) obtained "an authentic specimen of *gunja*" from India, percolated it with alcohol, and evaporated off the alcohol. He obtained a soft, dull green resin. Then he obtained American-grown hemp, and extracted it the same way. Procter calculated the percent resin extract from each sample as a proxy for potency.

Buchman (1874) tested 16 commercial cannabis indica extracts,, and compared them to a standard (7.83% resin, obtained from dried flowering tops of *C. indica* from India). Percent resin in the commercial products varied from 7.4% down to zero. Pharmaceutical companies standardized their products this way (Parke-Davis & Co., Eli Lilly, Upjohn Co., Squibb), until switching to the dog ataxia test. Evans (1894) ended the era of "percent resin" testing. He tested samples of *gañjā*, *bhāng*, and *charas* from across British India. He showed that percent resin did not correlate with potency gauged in a physiological assay (using cats, comparing their reactions to those caused by a "standard *gañjā*").

Except for studies of historical interest, we limited results to studies that employed analytical instrumentation. Specifically, we used studies that employed gas-liquid chromatography (GC) or high-performance liquid chromatography (HPLC). We omitted studies that used thin-layer chromatography. TLC is *not* quantitative; it cannot determine that a sample has, say, 10% THC. The sizes of spots on a TLC plate merely indicate relative amounts of THC and CBD in a sample. Debruyne *et al.* (1994) demonstrated that TLC results vary widely, depending on the mobile phase reagent.

Restricting data to GC and HPLC still introduces variables. To quantify fractions as they exit GC or HPLC, various detection methods are used, such as GC-UV (ultraviolet light detector), GC-TCD (thermal conductivity detector), GC-FIDS (flame ionization detector), GC-MS (mass spectrometry), HPLC-MS, HPLC-UV (ultraviolet light detector), HPLC-DAD (diode array detector), HPLC-FLD (fluorescence detector), as well as quantitative $^1$H-NMR (nuclear magnetic resonance). See Hazekamp *et al.* (2005) for a review.

Debruyne *et al.* (1994) analyzed the same *hashīsh* specimen using different methods, and found variable peak sizes. Using GC-FID, peak sizes were THC=CBD>CBN; using HPLC-UV, peak sizes were CBN>THC=CBD.

GC studies conducted prior to the mid-1970s overestimated CBD content, because they used packed columns, which combined CBD and cannabichromene (CBC) under one peak. Turner and Hadley (1973) devised a method to separate CBD from CBC. They added reagents to cannabis extracts that created timethylsilyl ethers of CBD and CBC, which easily separated by

GC. Novotyný *et al.* (1976) pioneered the use of capillary columns instead of packed columns, which separated CBD and CBC peaks.

Combining CBC+CBD under one peak may substantially alter the THC/CBD ratio in plants with little CBD. To illustrated this, Hillig and Mahlberg (2004) measured THC%, CBD%, and CBC% in 68 accessions South Asian heritage ("NLDs"). The mean THC/CBD ratio equalled 274, whereas the mean THC/CBD+CBC ratio equalled 26.

As explicated in the main document, we employed the THC/CBD ratio, rather than THC%. THC% correlates with the density of capitate stalked glandular trichomes on bracts, $p < 0.001$ (Potter 2009). Thus THC% is a trait linked with the "velous calyx" described by Lamarck (*i.e.,* perigonal bract with a dense pubescence of capitate stalked glandular trichomes). Environmental factors also modulate THC%, such as the intensity and spectrum of light, photoperiod, soil nutrients, and temperature. Gender is another factor; female plants produce higher THC% than males. THC% is also a function of plant age and "peak maturity" (Callaway 2008).

Utilizing the THC/CBD ratio to classify plants began with Grlić (1968). He used UV spectroscopy, which regrettably is not a very quantitative method. Researchers soon turned to GC-FIDS. Most studies reported THC and CBD as w/w percentages of dried flowering tops, which we converted to ratios as THC%/CBD%. Coy Waller's group at the University of Mississippi added cannabinol (CBN) to the ratio, as THC+CBN/CBD (Fetterman *et al.* 1971).

Fairbairn and Liebmann (1974) first proposed that the "qualitative picture" (THC/CBD ratio) is a genetic trait, independent of environmental conditions. The THC/CBD ratio stays relatively consistent despite many variables—gender, maturity stage, plant part, place of cultivation, and year of cultivation (Latta and Eaton 1975, Rowan and Fairbairn 1977, Fournier 1981, Barni-Comparini *et al.* 1984, Hanuš *et al.* 1987, Hanuš and Dostálová 1994, Pacifico *et al.* 2008, Broséus *et al*. 2010, Sikora *et al.* 2011, De Backer *et al.* 2012).

## SF.6. Protologues of the four varieties

A protologue is everything associated with a taxonomic name at its first valid publication, including its diagnosis or description, synonymy, illustrations, references, and type specimen (Turland 2018). The entire protologues of *C. sativa* and *C. indica*, including photographs of their type specimens, are provided by McPartland and Guy (2017). Here is a synopsis:

**1.** The protologue of ***C. sativa*** (Linnaeus 1753) restricted that taxon to fiber-type plants of European provenance. Linnaeus listed five taxonomic synonyms, all coined by northern European botanists. He excluded taxa of Asian provenance from the synonymy. Earlier, Linnaeus (1737) synonymized several taxa assigned to psychoactive Asian *Cannabis*. These Asian taxa appeared in a 1746 draft of *Species Plantarum* (manuscript at the Linnean Society of London), but they were deleted from the final version. Linnaeus's type specimen of *C. sativa* also came from northern Europe, likely from Uppsala, Sweden.

**2.** The protologue of ***C. indica*** (Lamarck 1785) included plants from India (Goa and Kochi), Indonesia, and South Africa. Lamarck's description of *C. indica* differed from *C. sativa* by eight "very distinct" morphological characters in stalks, branching habit, leaves, leaflets, and female

flowers. Lamarck also described chemotaxonomic differences: *C. indica* produced a strong odor, and was psychoactive. "The principal effect of this plant consists of going to the head, disrupting the brain, where it produces a sort of drunkenness that makes one forget one's sorrows, and produces a strong gaiety." Linnaeus's type specimen of *C. indica* came from India, likely Pondicherry.

**3.** Vavilov's protologue of ***C. sativa* f. *afghanica*** is complex and inconsistent. It has been translated twice into English (Vavilov 1926, 1992), with subtle differences. For example, "hemp relapsed in a wild state" (Vavilov 1926) becomes "naturalized hemp" (Vavilov 1992). "Bands of weed hemp" (Vavilov 1926) becomes "Belts of 'black' hemp" (Vavilov 1992). Vavilov found *afghanica* growing along the Kunar River in Afghanistan, from Gursalik (now Taranīk), through Chekosarai (now Asadābād), to Jalālābād.

Vavilov equivocated whether *C. sativa* f. *afghanica* was truly wild or a recent escape of cultivated plants. In a chapter about "overlapping characters of wild and cultivated hemp," he wrote, "The cultivated type of the Afghani small-seeded wild hemp, with a thin perianth, is a telling instance of such overlapping." He described *afghanica* as "a morphological link between the wild and cultivated races of hemp."

Yet he said *afghanica* has "light-colored small fruits [achenes] and with a thin perianth easily taken away… The hemp forms collected on the river Kunar are distinguished by shattering, by a developed horse-shoe" [*a.k.a.,* a protuberant base with a prominent abscission zone]. The achenes "germinate very slowly and unequally when sown, *i.e.,* show all features of a typical wild plant."

Vavilov also couldn't decide whether or not *afghanica* resembled plants he saw in Turkestan. He described leaflets, "distinguished by their obovate narrow shape, not observed by us among the European, Siberian and Turkestani forms." Yet plant habit was "medium-tall growth and having many branches, which is typical also of the common Turkestani forms."

Vavilov tabulated the results of a CGE conducted by Serebriakova, who grew *Cannabis* germplasm collected in Saratov, the Altai Mountains, and Afghanistan. Afghani plants were 60-150 cm tall, with stalks of intermediate thickness, very branchy; leaves were medium in size, with 5-9 leaflets, and shaped "narrow obovate." Vavilov used "narrow" to qualify "obovate." A better descriptor for narrow obovate is oblanceolate. Vavilov photographed an herbarium specimen—the exact same specimen that appears in Fig. 6b (in the main document)—and the leaflets are oblanceolate.

Vavilov's table presents other morphological characters of Afghanistan plants in a curious series of dichotomies: either small-sized achenes (2.7-3 mm long) or medium-sized achenes (3-4 mm), either with a "horseshoe" (abscission scar) or without one, and either dark colored achenes or light colored achenes.

The either-or dichotomies were explained three years later, when Vavilov and Bukinich (1929) named a second taxon of Afghan plants: *C. indica* var. *kafirstanica*. The either-or descriptions in Vavilov (1926) referred to either *afghanica* or *kafirstanica*. Vavilov and Bukinich described *kafirstanica* with tiny achenes, dark-colored and marbled, with a horseshoe. They

described *C. indica* f. *afghanica*,with larger achenes that were light colored ("white"), and a colorless involcre (perianth), with little or no horseshoe (Fig. S6).

Figure S6. Achenes illustrated by Vavilov and Bukinich (1929). Translation of original caption: Left to right: 1. from northern Afghanistan—cultivated form—*Cannabis sativa* L.; 2. ordinary Russia hemp from Orel; 3. wild hemp from Saratov; 4., *Cannabis indica* f. *kafiristanica* Vav.; 5. *Cannabis indica* var. *afghanica* Vav. The upper row enlarged 6 times, the lower row showing the bases of achenes enlarged 10 times.

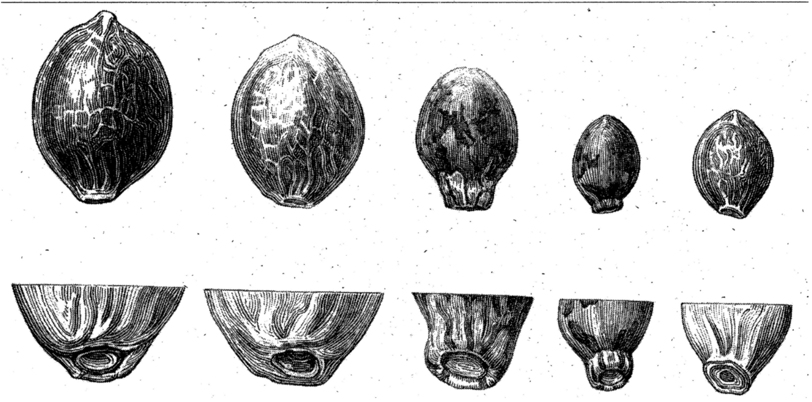

Information in Vavilov and Bukinich (1929) is not part of the *afghanica* protologue (Vavilov 1926), but it helps inform our decision to treat *afghanica* as a naturalized escape of cultivated plants, rather than an indigenous wild plant. The *afghanica* achene illustrated by Vavilov (Fig. S6), as well as achenes in his herbarium specimen (Fig. 3g in main document) show traits of domestication—the loss of a persistent perianth with camouflagic mottling, and no elongated base, drawn out in the shape of a short, tapered stub. The *afghanica* achene resembles a small version of the Afghani "cultivated form" in Fig. S6. The larger achene size of the cultivated plant would have been augmented by irrigation and fertilizer.

Vavilov (1926) claimed that *afghanica* grew "in districts where the cultivation of this crop is entirely unknown." In fact, cultivation for *hashīsh* was well-established in the Chitral River valley by the 1930s (Staley 1966). Chitral is the name of the Kunar after it crosses into Pakistan, 70 km from where Vavilov collected *afghanica*. In the nearby Kurram River valley, cultivation for *hashīsh* was observed long before that (Aitchison 1869).

Vavilov and Bukinich (1929) wrote, "Usual ecological conditions for growth of weed-wild hemp are abandoned lots or neglected plots of land, with unsodden fertilized soil, agricultural fields, fields with maize and cotton." Arable land is neither abandoned nor neglected in the Kunar River valley. Its agricultural soil is a rare and valuable resource in Afghanistan. A few years later, Vavilov (1931) made a similar statement regarding valuable land in the Yarkand oasis, "The vacant lots of Yarkand are full of hemp thickets."

Vavilov observed *afghanica* "following the sowing of corn and other cereals" (Vavilov 1926), or "emanating from crops of maize and other cereals" (Vavilov 1992). He provided a photograph of "wild hemp" growing amidst maize (Vavilov and Bukinich 1929). Intercropping

*Cannabis* with maize is a method of preventing detection around the world, including Chitral (Nüsser and Dickoré 2002) and Afghanistan (UN-ODC 2010).

Vavilov and Bukinich (1929) transferred *afghanica* from *C. sativa* to *C. indica*, but with inconsistent nomenclature: "*C. indica* var. *afghanica*" on page 380, "*C. indica* f. *afghanica*" on page 381, and "*C. indica* var. *kafirstanica* forma *afghanica*" on page 382.

Typifying Vavilov's taxa is problematic, because Vavilov collected germplasm, and did not make herbarium specimens. Serebriakova cultivated Vavilov's germplasm at the Kamenno-Stepnaya Experiment Station (Voronezh Oblast), and prepared voucher specimens. No specimen labeled *afghanica* is deposited at WIR. Seven herbarium specimens are labelled *C. sativa* var. *spontanea*, and annotated "like ruderal" (WIR 4031, 4032, 4034, 4036, 4038, 4044, 4046). Labels indicate that four came from Asadābād ("Chekosarai"), and two came from Kāfiristān. All are immature, without seeds, and could be either *afghanica* or *kafirstanica*.

The *afghanica* neotype designated herein came from germplasm collected by Vavilov at Gui-Akhen (Гуй-Ахен). Vavilov observed cultivation for *hashīsh* at Gui-Akhen (Table S8), so the germplasm came from a cultivated plant. The achenes are green and reticulated, with light brown mottling near the base, no protuberant base, length $\bar{x}$= 4.0 x 2.8 mm (Fig. 3g in main document). This falls into the range that Vavilov (1926) gave for medium-sized achenes (3-4 mm), as opposed to small-sized achenes (2.7-3 mm long) for plants he considered truly wild. The neotype's achenes closely resemble the achene that Vavilov illustrated for *afghanica* (Fig. S3). The neotype inflorescences are relatively small (they were cultivated in Russia), but compact and leafy, with ample capitate-stalked glandular trichomes, and agglutinated with trichome exudate.

**4.** The protologue of ***C. indica* var. *kafiristanica*** (Vavilov and Bukinich 1929) includes a description, line drawing, and photographs. The authors were not consistent with their nomenclature: *C. indica* f. *kafiristanica* appears in the caption of their illustration on page 380, but *C. indica* Lam. var. *kafiristanica* Vav. appears in the formal description on page 381.

Their Russian description, translated: "Races of wild hemp in eastern Afghanistan have extremely small fruits with mosaic (1000 fruits weigh 2.1-2.7 g), *i.e.,* 6-8 times smaller than small-seeded Central Russian cultivated hemp (Orel and Kursk hemp weighs 17-19 g). Characteristic for them is ready shattering of fruits due to the presence of a horseshoe, slow and uneven germination, *i.e.,* the usual attributes of a wild plant. As regards to vegetative features, Afghan wild–weedy hemp is distinguished by small leaves with obovate leaflets of narrowed shape. In general, it is characterized by short stature, profuse branching from the first internode, and by short internodes."

Vavilov and Bukinich also mention early ripening (90–100 days in Voronezh Oblast). They provided a drawing of the achene (Fig. S6). Although Vavilov and Bukinich described *kafiristanica* plants as short in stature, a photograph of plants identified as *C. indica* var. *kafiristanica* (Fig. 267 in Vavilov and Bukinich) shows plants equal in height to maize plants with tassels.

Two specimens labeled *C. sativa* var. *kafiristanica* are deposited in Vavilov's herbarium at WIR in St. Petersburg. Small and Cronquist (1976) chose one as the lectotype: WIR 3952,

germplasm collected by Vavilov at Chekhosarai (now Asadābād) in 1924, and cultivated in 1927 at Pushkin Experiment Station (Detskoye Selo, St. Petersburg). Incidently, Asadābād is in Kunar Province, not Kāfiristān (present-day Nuristān Province), so *kafiristanica* was not collected in Kāfiristān.

A photograph of the lectotype appeared in Vavilov and Bukinich (1929), and appears here (Fig. 6b in the main document). The plant is 30 cm tall, staminate (male), with tight internode spacing; nine pairs of opposite branches below, and three alternate branches near the apex. Leaves have 5–7 overlapping leaflets, petioles long and thick, leaflets broad, oblanceolate, dark green, with coarse serrations, up to 46 × 18 mm. The paratype, WIR 3953, is a pistillate plant. Although the inflorescences are immature, they are nevertheless compact and leafy, with an abundant covering of capitate-sessile glandular trichomes.

**5.** The protologue of ***C. sativa γ asperrima*** (Regel 1879) consists of a brief description (translated from Latin): “upper leaves *latere* [lateral branching?], perigonal bracts with short setae, nipple-like glands, dense and rough. Near Lake Issyk-Kul close to Karakol and to the river Dschirgalan. Legacy [collected by] A. Regel.”

Dschirgalan today is spelled Jyrgalan, a river that flows into Lake Issyk-Kul at Karakol. Regel adds that *C. sativa* is cultivated [for what—drugs or fiber—he doesn’t say] and grows quasi-spontaneously in Eastern and Western Turkestan. Regel did not designate a type specimen. Two specimens of *C. sativa γ asperrima* are deposited at herb. LE, both collected by his son, Albert Regel, a district physician in Kudja (Yīníng). Neither specimen was pressed correctly, and both are chewed up. The better-preserved specimen we designated as lectotype (Fig. 6a in the main document).

Regel (1879) contrasted *asperrima* with a plant that his son collected 200 miles away, along the upper Bortala River in Xīnjiāng Region. To this specimen Regel assigned the name *C. sativa β vulgaris*, with a brief description, “stems 3–6 feet tall, upper leaves scabrious, perigonal bracts *laxe* [loose, apart from one another] minute glands.” The taxon *C. sativa β vulgaris* had been coined by De Candolle (1869), who assigned it to an impossibly wide range of plants—those growing spontaneously in Central Asia, cultivated in Europe, and both cultivated and spontaneous plants in India. Regel’s specimen of *C. sativa β vulgaris* (herb. LE) differs little from the *asperrima* specimens, except for larger wild-type achenes, 3.5-4.0 mm long (Fig. S7). To us it represents another *C. sativa γ asperrima* specimen.

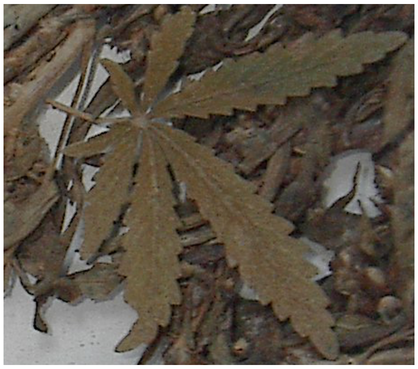

Figure S7. Herbarium specimen of *C. sativa β vulgaris* collected by A. Regel in Xīnjiāng Region (herb. LE).

**6.** The protologue of ***C. sativa* var. *himalayensis*** (Cazzuola 1875) consists of a brief description, which compares it to Chinese hemp and *C. gigantea* (Piedmont hemp of Italy). "This variety is largely similar to Chinese hemp in its height, but differs by being not so branchy, to have thin stalks, and grows taller. The fiber of this variety is very tenacious, but not as fine as that of *C. gigantea*." Cazzuola did not provide a synonymy or illustrations. No specimens of *C. sativa* var. *himalayensis* exist in Cazzuola's herbarium collection (pers. communications, Lucia Amadei, herb. PI).

## SF.7. Nomenclatural priority: debates over Persoon and Cazzuola

Plant taxa with a given circumscription and rank can only have one correct name, according to the *International Code of Nomenclature for Algae, Fungi, and Plants* (*ICN*, Turland 2018). The correct name is the earliest legitimate one (*ICN* Article 11.3). This applies to species names that are subsequently reduced to an infraspecific taxon, for example when *C. indica* is reduced to *C. sativa* subsp. *indica*. Thus, the earliest legitimate name has priority.

Persoon (1807) reduced Lamarck's taxon to an infraspecific taxon, as *C. sativa β indica* (Lam.) Persoon. He implicitly accepted the differences by which Lamarck separated *indica* from *sativa,* but he did not think the differences warranted segregation at the rank of species. Persoon's infraspecific taxon was accepted by many other botanists. Nine botanists are listed by McPartland and Guy (2017), with publication dates between 1823 and 1865. Three other botanists, unaware of Persoon, repeated his efforts. Fristedt (1870) coined *C. sativa* var. *indica* (Lam.) Fristedt. Siebert and Voss (1896) coined *C. sativa* f. *indica* (Lam.) Voss. Wehmer (1911) coined *C. sativa* var. *indica* (Lam.) Wehmer.

Small and Cronquist (1976) accepted Wehmer's synonymy. They believed that Persoon's name was not validly published because Persoon's name was not published with any indication of being based on Lamarck's *indica*. In fact, Persoon cited "Lam. ill. gen. t. 814" (Fig. S8), which is shorthand for Lamarck, *Illustration des genres*. This title is better known as *Tableau encyclopédique et méthodique Botanique* (Lamarck 1799). Persoon erred there—*C. indica* appeared in Lamarck (1785), not in Lamarck (1799). But as previously argued (McPartland 1992), the *ICN* does not invalidate pre-1953 recombinations containing bibliographic errors (see Articles 41.6 and 46.3, Turland 2018). The publication date of Lamarck (1799) is documented by Stafleu and Cowan (1979). The text was republished by Lamarck and Poiret (1823).

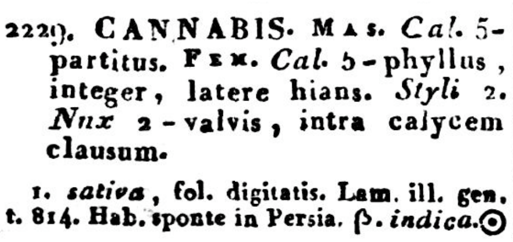
2229. CANNABIS. MAS. *Cal.* 5-partitus. FEM. *Cal.* 1-phyllus, integer, latere hians. *Styli* 2. *Nux* 2-valvis, intra calycem clausum.

1. *sativa*, fol. digitatis. Lam. ill. gen. t. 814. Hab. sponte in Persia. β. *indica*. ⊙

Figure S8. Persoon (1807) reduced Lamarck's *C. indica* to a variety under *C. sativa*.

The taxon *C. sativa* subsp. *indica* var. *himalayensis* also raises issues regarding priority. Koch (1854) wrote a lengthy description of Chinese hemp (*Cannabis chinensis*), and in one

sentence he compares Chinese hemp to Himalayan hemp. Regarding the latter, Koch applied the taxon *C. sativa* var. *himalayensis,* but he did not provide a clear diagnosis of the plant. Koch considered *C. chinensis* a genuine species, "Maxime *Cannabis sativæ* varietati *himalayensi* (*C. sativæ* Roxb. Flora indica III, 772, *C. indicæ* Rumph, Herb. Amb. V, t. 77) accredit, sed in nostris regionibus magis refrigertis nunquam maturescit."

Translated: "Very much like *Cannabis sativa* variety *himalayensi*s (*C. sativa* Roxb. fl. ind. III, 772, *C. indica* Rumph, herb. amb. V, t. 77), but in our rather cool countries will not be brought to maturity." Thus Koch parenthetically equated variety *himalayensis* with domesticated Indian hemp described by Roxburgh (1832) and *Cannabis indica* described by Rumph (1747), which is an erroneous concept. Furthermore, Koch does not provide a full description, so his taxon is judged a *nomen nudum* and not validly published (*ICN* Art. 38.2, Turland 2018).

Cazzuola (1873) also compared *canapa dell'Imalaia* (Himalayan hemp) to Chinese hemp, "since this variety has a coarse bark, you must keep it submerged a few more days more than Chinese hemp." He coined the taxon *Cannabis sativa* var. *hymalaiensis*, but his lack of a clear diagnosis is judged a *nomen nudum* and not validly published (*ICN* Art. 38.2, Turland 2018).

Subsequently, Cazzuola (1875) provided a somewhat more detailed description of *C. sativa* var. *himalayensis,* which we translate in the previous section on protologues. We consider this description adequate, and assign priority to Cazzuola (1875).

## SF.8. Morphological comparisons: South Asian *C. indica* (domesticated) and *C. himalayensis* (wild-type)

Hindu tradition maintains that Lord Shiva discovered wild *bhāng* in the Himalaya (*i.e.,* wild-type *C. himalayensis*), and brought it down to the Indian plains as a gift to humans (*i.e.,* domesticated *C. indica*) (Grierson 1894).

In historical times, people in the Indian plains cultivated *C. indica,* nearly exclusively for drugs, *bhāng* and *gañjā*. A different scenario emerged in the Himalaya, where plants supplied fiber for cordage and cloth, as well as drugs. *Rājataranginī*, the earliest history of Kashmir (written around 1150 CE), describes garments made of *bhaṅgā* (Kalhana 1989). The Himalayan drug, rather than *gañjā,* was hand-rubbed *charas* (*hashīsh*). Although present-day authors say *charas* traditionally came from wild-type plants (*e.g.,* Clarke 1998), early British explorers often described *charas* rubbed from cultivated plants (Table S4).

Table S4. Early descriptions of Himalayan plants

| citation | location; **C** = cultivated, **W** = wild-type; other observations |
|---|---|
| Eliot (1794) | Garo Hills, Meghalaya, India; **C**: for fiber only; Tibeto-Burman ethnic group |
| Hardwicke (1801) | Haridwar, Garhwal, Uttarakhand, India; **C**: fiber and "an intoxicating drug" |
| Kirkpatrick (1811) | Kathmandu valley, Nepal; **C**-or-**W** not stated; fiber and hand-rubbed *cherris* |
| Moorcroft (1816) | Garhwal, Uttarakhand; **C**: fiber, no mention of drugs; plants 12 ft. tall. |
| Hamilton (1819) | Nepal; **W**: *charas* and *gangja*, no mention of fiber |

| | |
|---|---|
| Traill (1828) | Garhwal, less so Kumaon, Uttarakhand; **C**: one *pucca bigha* (0.253 ha) yields 4 *maunds* fiber (*i.e.,* 590.0 kg/ha) worth Rs. 8, and 4 *seers* of *chiras* (14.75 kg/ha) worth Rs. 8; plants 12-14 ft. tall |
| Royle (1839) | Garhwal, Uttarakhand, and Sirmaur, Himachal Pradesh, India; **C**-or-**W** not stated; for fiber and "intoxicating drug," plants 10-12 ft tall |
| O'Shaughnessy (1839) | Nepal; **C**-or-**W** not stated; originated the myth of *churrus* "gathered on the skins of naked coolies" running through fields of plants |
| Batten (1855) letter dated 1840 | Garhwal, Uttarakhand; **C**: female plants (*goorbhunga*) for *churus* and fiber; male plants (*phoolbhunga*) for fiber; **W**: *gunarabhunga* (also called jungle hemp or *bun bhunga*), provided "a little *churus*…and an inferior rope… but in general this species is considered and treated as useless," plants 10 ft tall |
| Huddleston (1841) | Pauri District, Garhwal, Uttarakhand; **C**: *phoolbang*, 1 acre yields 4 *maunds* fiber (worth Rs. 8), 3 *seers* of *churrus* (worth Rs. 6), 30-45 *seers* of seed; rarely cultivated below 3000 ft, "the heat of the valleys being detrimental to its quality;" **W**: *khur-bhunga* or jungle bhang, yields an insignificant quantity of *churrus.* |
| Swetenham (1841) | Garhwal, Uttarakhand; **C**: for fiber and seed (no mention of drugs), plants 8 ft tall; **W**: for *churus* and not fiber |
| Kirke (1842) | Deyrah (Dēhrādūn), Uttarakhand, India; **C**: fiber, seed, *bhung*, and *churrus* |
| Madden (1848) | Kumaon, Uttarakhand; **C**: female plants (*goon-bhanga*) for *gunja* and seed oil; male plants (*phool-bhanga*) for fiber; W: *jungulee-bhanga* for *churrus* |
| Christison (1851) | district from Almora to Mussoorie, Uttarakhand, India; **C**: fiber and *churrus*; "dozens of villages… hundreds of men, women, and children, all employed in making *churrus,"* plants 10–14 ft. tall |
| Thomson (1852) | Jhelum River, Kashmir; **W**: "grows spontaneously along the banks of the river, forming dense thickets often 12 and 15 feet in height, and almost impenetrable. It is only used in the manufacture of an intoxicating drink, and for smoking" |
| Hooker (1854) | Tambur (Tamur) river, near border with Sikkim, easternmost Nepal: **C**: "the small-leaved variety of hemp (*Cannabis*) grown as a narcotic" |
| Thornton (1859) | Sirmour (Sirmaur), Himachal Pradesh; **C**: "*bang* or hemp, for narcotics" |
| Jacquemont (1861) | Kashmir Valley, Kashmir; **W**: "Collecting the wild plant to extract the intoxicating dust brings about 25,000 rupees per year for the treasury of Ranjit Singh" |
| Watson (1862) | Himachal Pradesh; C: "grows spontaneously and in abundance everywhere in the submontane tracts, but is cultivated for the fibre only in the eastern parts of Kangra, and in the Simla Hills" |
| Lawrence (1895) | Kashmir Valley, Kashmir; **W** and **C**: fibre and *charas* |
| Hooker (1890) | N.W. Himalaya, India: **W**: fiber, *bhang*, and *kief,* plants to 8 ft tall |
| Indian Hemp Drugs Commission (1894) | location records of **W** growth rubbed for *charas* include Urgum (Garhwal, Uttarakand), Almora (Almora, Uttarakhand), and Kullu (Himachal Pradesh) |

Were the cultivated plants in Table S4 simply wild-type *C. himalayensis* brought into cultivation, or were they truly domesticated? There is a difference. Cereals domesticated in the Fertile Crescent may have taken 1000 years of cultivation to fix some traits of domestication, and legumes took longer (Langlie *et al.* 2014). This protracted process was due, in part, to continued outcrossing between cultivated and wild-type plants. Among traits preserved in the archaeological record, the initial trait was enlarged seed, and nonshattering traits (reductions in abscission/dehiscence layers) came last.

Although Small (1975) observed that domesticated *C. sativa* reverted to a wild-type phenotype in just 50 years, the length of time required for wild-type *Cannabis* to acquire traits of domestication is unknown. Himalayan people have cultivated *Cannabis* for fiber since at least 1000 BCE—a fossil pollen study in Garhwal found unnaturally high levels of *Cannabis* pollen, indicative of a former hemp-retting pond (Demske *et al.* 2016).

The oldest archaeobotanical evidence comes from the plains of India near the Himalaya: at Kunal in Haryana, 2600-2500 BC; and Hetapatti in Uttar Pradesh, 2500-1500 BC (Table S3). Achenes at both sites showed a mixture wild-type and domesticated traits.

Huddleston (1841) observed wild and cultivated plants in Garhwal, "I imagine the cultivated kind must have originally been wild and rendered productive by culture…for they have both the same appearance with the exception of the cultivated kind, growing to a greater height and thickness of the stem, *producing a heavier and fuller seed* [emphasis added], and not throwing out so many branches." Batten (1855) wrote about *Cannabis* cultivation in Garhwal, "the first introduction of which into the hill agriculture, whether from the wild plant of the country, or from elsewhere, is not now discoverable."

Batten's comment, "from elsewhere," could apply to Chinese fiber-type hemp. Roxburgh (1815) tried to import Chinese germplasm into India. Earlier, Roxburgh (1804) grew English hemp for rope at Calcutta. Russian germplasm was also imported into Bengal (Deneef 1841). As early as 1807, a British trade agent named Thomas Rutherford encouraged hemp cultivation in Uttarakhand (Huddleston 1841, Bayly 1996). The British sent hemp experts to Himalayan border towns, "instructing the people in the proper mode of preparing it" (Christian 1855).

Koch (1854) and Cazzuola (1873, 1875) gave brief descriptions of *C. himalayensis,* detailed in section SF.6. According to Koch and Cazzuola, the taxon's key characteristics were its tall height, potential as a source of bast fiber, and its similarity to Chinese hemp. Zinger (1898) described wild-type achenes in *C. himalayana* (Fig. S3).

Bredemann (1952) conducted a CME with Himalaya germplasm that he named "Almora", after Almora in Uttarakhand. He compared germplasm from southern India ("Indore", "Bangalore"), and Europe (Russia, Hungary). "Almora" grew 3.5-4 m tall, and matured late in Germany—frost killed females before achenes matured. Bredemann measured bast fiber content in dry shoots; Almora contained 12.3%—a higher percentage than fiber-type landraces from Russia (9.5%) and Hungary (10.3%), as well as Indore (9.1%) and Bangalore (8.8%).

Sharma (1975) compared two populations of plants in India, from Hoshiarpur (on the Punjab plain, 250 m elevation, summer temperature 40ºC, xeric conditions) and Shimla (only 200 km away, but high in the Himalaya, 2200 m, 15ºC, humid). Sharma attributed differences to climate, but they may have been varietal—Shimla plants, *C. himalayensis;* and Hoshiarpur plants, *C. indica.* However, the Hoshiarpur plants might have been *C. afghanica*, see our comment below regarding Turner *et al.* (1979). Sharma found significant differences in stomate density and "trichome" density, and less-than-significant differences in "trichome" size (Table S5).

Table S5. Cystolith comparisons by Sharma (1975)

| character | Hoshiarpur | Shimla |
|---|---|---|
| stomates per ocular field (0.152 $mm^2$) | 48.9 | 27.1 |
| cystolith density: | | |
| upper leaf surface | 63.8/$cm^2$ | 28.3/$cm^2$ |
| lower leaf surface | 253.6/$cm^2$ | 133.6/$cm^2$ |
| cystolith length: | | |
| upper leaf surface | 70.9 µm | 47.3 µm |
| lower leaf surface | 134.1 µm | 112.1 µm |

In a subsequent publication, Sharma (1979a) revealed the "trichomes" were cystoliths. He made more comparisons: Plants at Shimla compared to Hoshiarpur were taller, more laxly branched, grew more vigorously, with larger and bright green leaves. At Shimla, seedlings emerged in March and bloomed in early June. At Hoshiarpur, seedlings emerged by early January and achenes ripened in November.

Turner *et al.* (1979) analyzed 20 populations of wild-type plants growing in northern India, from the Punjab plains to the Himalaya. Other than achene size, no wild-type traits were recorded (*e.g.,* elongated base, persistent perianth). It's hard to tell whether they collected truly ruderal plants or recent escapes from cultivation. Some extremely high THC+CBN/CBD ratios (up to 156) suggest at least some accessions were recent escapes. They divided accessions into two groups: plants above 2000 m (n = 9), or plants below 2000 m altitude (n = 11). In plants above 2000 m, leaflet L/W ratio $\bar{x}$ = 8.80; in plants below 2000 m, leaflet L/W ratio $\bar{x}$ = 8.76. Achene length $\bar{x}$ = 2.31 mm for plants above 2000 m, and $\bar{x}$ = 2.15 mm for plants below 2000 m.

Achenes from six populations were shipped to Mississippi to conduct a CGE. Curiously, achenes from two populations shipped to Mississippi were described in India as immature and lacked achenes. Plants growing *in situ* in India, compared to the Mississippi garden, were shorter (mean 1 *vs.* 2 m tall), with fewer leaflets per leaf (3-5 *vs.* 5-7), shorter central leaflets (5.9 *vs.* 8.4 cm), broader central leaflets (0.79 *vs.* 0.58 cm), and smaller achenes (2.5 *vs.* 3.2 mm).

De Meijer and Keizer (1996) compared morphological variation in 160 accessions in a CGE, and provided raw data (de Meijer 1994a), and passport information (de Meijer and van Soest 1992). Their passport data, plus our inspection of voucher specimens (via Hillig, herb. IND), enabled us to identify three non-hybridized *C. indica* accessions (891384, 891385, 910972) and three *C. himalayensis* accessions (891191, 891192, 891193). We calculated means from this small data set (Table S6).

Table S6. Comparisons of *C. indica* and *C. indica* accessions, from de Meijer (1994a)

| character | *C. indica* | *C. himal-ayensis* | statistical difference (t-test) |
|---|---|---|---|
| achene weight (g/1000) | 12.6 | 11.3 | $p$ =0.30 |
| achene length (mm) | 3.80 | 3.77 | $p$ =0.93 |
| abscission zone (rated from 1, always absent; to 7, conspicuously present | 3.0 | 5.3 | $p$ =0.18 |
| perianth marbling (rated from 1, always absent; to 7, conspicuously marbled | 5.6 | 6.0 | $p$ =0.80 |

| | | | |
|---|---|---|---|
| height of mature female plants (cm) | 224.3 | 258.3 | *p* =0.47 |
| leaflet L/W ratio, central leaflet, node 4 | 19.0 | 22.7 | *p* =0.33 |
| total bast fiber in dried stems (%) | 12.5 | 14.9 | *p* =0.09 |
| primary bast fiber in dried stems (%) | 12.2 | 13.2 | *p* =0.55 |
| secondary bast fiber in dried stems (%) | 0.57 | 1.67 | *p* =0.18 |
| wood (xylem) cell length (µm) | 543.1 | 593.3 | *p* =0.06 |
| wood in dried stem (%) | 64.8 | 64.7 | *p* =0.98 |
| time to seed maturity (days) | 315.0 | 316.7 | *p* =0.94 |
| *Meloidogyne hapla* egg masses, # per g root weight | 65.5 | 32.6 | *p* =0.08 |

Few differences in the two groups approached statistical significance. However, one accession (Dana, Nepal, 891193) was alternatively labeled wild-type (de Meijer and van Soest 1992) or a domesticated fiber-type (de Meijer 1994a). Omitting that accession, we see greater differences in achene weight (g/1000): *indica* $\bar{x}$= 12.6, *himalayensis* $\bar{x}$= 7.4 (*t*-test *p*=0.13); achene length: *indica* $\bar{x}$= 3.80 mm, *himalayensis* $\bar{x}$= 3.45 (*t*-test *p*=0.17); abscission zone: *indica* $\bar{x}$= 3.0; *himalayensis* $\bar{x}$= 7.0 (*t*-test *p*=0.07), and perianth marbling: *indica* $\bar{x}$= 5.7; *himalayensis* $\bar{x}$= 7.0 (*t*-test *p*=0.22),

Hillig (2005b) grew 135 accessions in a CGE, and analyzed 34 phenotypic characters. He grouped accessions into seven biotypes, including "narrow-leaflet drug" (NLD, *C. indica* herein), and "*C. indica* feral" biotype (*C. himalayensis* herein). Hillig assigned the latter biotype to the taxon *kafiristanica,* therefore adopting Small and Cronquist's *kafiristanica* concept. Hillig provided narrative descriptions of the biotypes (Table S7).

Table S7. Morphological differences between NLD biotype (*C. indica* herein) and "*C. indica* feral" biotype (*C. himalayensis* herein) reported by Hillig (2005b)

| character | *C. indica* (n = 25) | *C. himalayensis* (n = 5) |
|---|---|---|
| achenes | small to medium in size, tan to dark brown; perianths variably mottled, sometimes adherent to achenes. | small, dark brown, often constricted at the base, fall from the plants at maturity, germinate unevenly; perianths mottled and adherent to achenes. |
| pistillate inflorescences | usually compact | elongated and loosely structured |
| leaves and leaflets | Central leaflets long, lanceolate or linear-lanceolate; biserrate margins common, especially southeast Asian strains. | Central leaflets shorter, lanceolate or oblanceolate; biserrate margins rare. |
| petioles | low width/thickness ratio (≤ 0.95) | nearly round in cross-section |
| branching, height | lax and moderate to strong on the lower stem; height usually 1.5 to 2.5 meters | lax and usually weak on the lower stem; height usually 2 to 3 or more meters |
| other characters | Stem aroma spicy, vegetative, or candy-like; monoecious plants frequent. | Stem aroma weakly vegetative or camphory; monoecious plants rare. |

Hillig's multivariate clustering procedure (PCA) showed overlap between *C. indica* and *C. himalayensis* accessions. However, canonical analysis segregated *C. indica* and *C. himalayensis*. Seventeen of 34 phenotypic characters showed significant differences (p≤0.05) between *C.*

*indica* and *C. himalayensis*: Achene length (mm): *C. indica*, 3.95; *C. himalayensis*, 3.12. Achene thickness (mm): *C. indica*, 2.50; *C. himalayensis*, 1.89. Stem diameter at base of plant (mm): *C. indica*, 11.5; *C. himalayensis*, 9.5. Central leaflet length (at node 10, mm): *C. indica*, 178; *C. himalayensis*, 133. Petiole width (mm): *C. indica*, 2.36; *C. himalayensis*, 2.06. Petiole width/thickness ratio: *C. indica*, 0.92; *C. himalayensis*, 0.98. Number of primary serrations on central leaflet: *C. indica*, 39.1; *C. himalayensis*, 32.3. Number of secondary serrations on central leaflet: *C. indica*, 12.4; *C. himalayensis*, 1.2. Stem length (ground to node 3, mm): *C. indica*, 221; *C. himalayensis*, 111.

**SF.8. Morphological comparisons, cont.**
**South Asian *C. indica* ("Sativa") and Central Asian *C. afghanica* ("Indica")**

William Johnson, a surveyor stationed in India, made the first comparison between South Asian and Central Asian plants. He was the first European in centuries to reach Khotan (*i.e.,* Hotan or Hétián, Xīnjiāng Region, China). Johnston (1867) observed, "The plant from which the *charas* is extracted is met with in almost every field; it differs slightly from the *charas* plant found in India, having broader leaves, and growing to a larger size."

Henderson and Hume (1873) described plants cultivated in Yarkand, Xīnjiāng Region, that grew 8–10 ft tall. Henderson labeled at least one herbarium specimen with a new scientific name, *Cannabis sinensis* (herb. K). The specimen's large, dense inflorescence was agglutinated with trichome exudate. Leaflets were large, up to 120 x 28 mm (L/W = 4.3), broad but not oblanceolate. Achenes had a light brown mottled perianth, mostly sloughed off; light green in color with prominent reticulate veination, no protuberant base, 4.5–4.9 mm long (Fig. 3I in main document).

Frank Meyer, a germplasm collector for the United States Department of Agriculture, collected achenes from Xīnjiāng Region (USDA 1912). The germplasm was cultivated in Nevada—the first *C. afghanica* grown in the USA. Plants matured rapidly (109 days), grew 5-6 feet tall, branched heavily, with large resinous tops that "give off a skunky odor" (Kennedy 1915). A photograph shows densely branching plants with wide-diameter leaflets (Fig. S9).

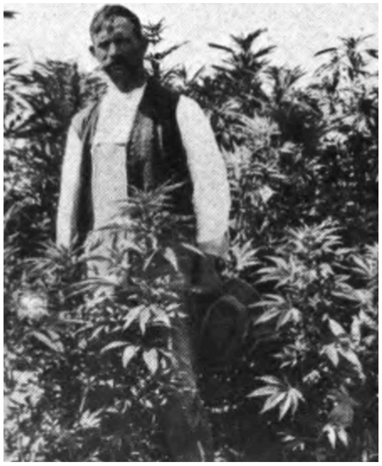

Figure S9. First Central Asian *C. sativa* cultivated in the USA. Photo reproduced from Kennedy (1915).

Nikolai I. Vavilov spent five months in Afghanistan. He encountered Afghans cultivating plants for наши or анаша (*nashi* or *anasha*), which he translated as гашиша (*gashisha, i.e.,* hashīsh). Vavilov collected germplasm at four locations and sent them to Serebriakova. She grew the accessions and assessed their morphology (Table S8). Vavilov and Bukinich (1929) summarized Serebriakova's opinion, "By all attributes it [Afghani plants cultivated for hashīsh] is linked directly with the Central Asian cultivated hemp, not with *C. indica,* which is distinguished by its small leaves, small achenes, and low height (up to 1 meter)."

Thus it was Serebriakova's decision to assign domesticated Afghani plants to *C. sativa*, based on a faulty concept of *C. indica.* Describing *C. indica* as a *diminutive* plant with *small* leaves is clearly erroneous—plants from South Asia are relatively tall with long leaflets. Serebriakova and Vavilov may not have seen a specimen of *C. indica* from India. The herbarium collection at WIR lacks specimens from India, although the LE collection, across town, did have specimens from India (McPartl., pers. observ., WIR and LE, 2010).

Table S8. Morphological measurements of *C. sativa* cultivated in Russia, from germplasm collected in Afghanistan. Table reproduced from Vavilov and Bukinich (1929).

| origin | Height (cm) | length of leaves (cm) | Number of leaflets per leaf | weight per 1000 seeds (gm) |
|---|---|---|---|---|
| Herāt | 134 | 15.7 | 9 | 22 |
| Sheberghān near Mazar-e-Sharīf | 129 | 15.8 | 9 | 18 |
| Fayzabad | 180 | 20.3 | 9 | 22 |
| Gui-Akhen near Kandahar | 134 | 15.1 | 9 | 18 |
| mean | 1.44 | 16.7 | 9 | 20 |

Herbarium specimens of these accessions (herb. WIR) showed dark green leaflets, L/W ratio 4.2 to 6.1, broadly lanceolate to oblanceolate, margins with coarse serrations, with long petioles. These characteristics are entirely consistent with our concept of *C. afghanica*.

Serebriakova and Sizov (1940) erected a special *proles* (race) for Central Asian drug plants, *C. sativa* subsp. *culta* proles *asiatica.* They described *asiatica* plants as short, strongly branched, nearly spherical in shape, leaves with many leaflets, leaflets large and wide, and achenes large. Within *asiatica* they recognized three varieties (Table S9). This excessive taxonomic splitting reflects the classic, anachronistic Russian school of taxonomy (Small 2011).

Table S9. Three varieties of "Narcotic Asiatic Hemp", *C. sativa* subsp. *culta* prol. *asiatica*, by Serebriakova and Sizov (1940)

| Taxon (not validly published) | stalks (length) | leaves | seed size | vegeta-tion cycle | geographical provenance |
|---|---|---|---|---|---|
| var. *narcotica* | 100-150 cm | large size, broad 9-13 leaflets | large size | 130-150 days | Central Asian Republics, Azerbaidzhan SSR, Iran, Afghanistan |
| var. *narco-tica* f. | 100-150 cm | large, broad, yellow-green | large size | 130-150 days | Western China |

| | | | | | |
|---|---|---|---|---|---|
| *flavo-viridis* | | 9-13 leaflets | | | |
| var. *sub-narcotica* | 120-150 cm | less large, 9-13 leaflets | less large | 130-150 days | Turkmen SSR, Uzbek SSR, Western China |

Serebriakova and Sizov (1940) segregated Indian drug plants into a different species, *C. indica*, described as 1.0-1.5 m (rarely 2-3 m), with small with narrow leaves, linear-lanceolate leaflets; female plants sometimes produce male flowers; female flowers bear “rather large” stigmas (a trait noted by Small and Naraine (2015a), as the result of insufficient pollination); achenes small and dark.

David Watson traveled to Kandahar in 1970, and collected germplasm from cultivated plants with wide-diameter leaflets. He also saw plants with narrow-diameter leaflets, but they grew spontaneously in ditches (Selgnij, pers. commun., in Clarke 1998). He utilized the germplasm to breed “Skunk #1”, a hybrid that became widely available in the early 1980s.

Richard Schultes conducted “preliminary field work” in Afghanistan. Schultes *et al.* (1974) assigned the name *C. indica* to the short, broad-leafleted plants he saw in Afghanistan. This ultimately resulted in the vernacular taxonomy of “Indica” and “Sativa” we see today. Schultes described and illustrated *C. indica* growing in Kandahar. The photo caption reads: “Pistillate plant: source of specimen *R. E. Schultes 26505*.” This is the herbarium specimen chosen as our epitype of *C. sativa* subsp. *indica* var. *afghanica* (Fig S10). The plant was an escape, growing spontaneously in a formerly irrigated field.

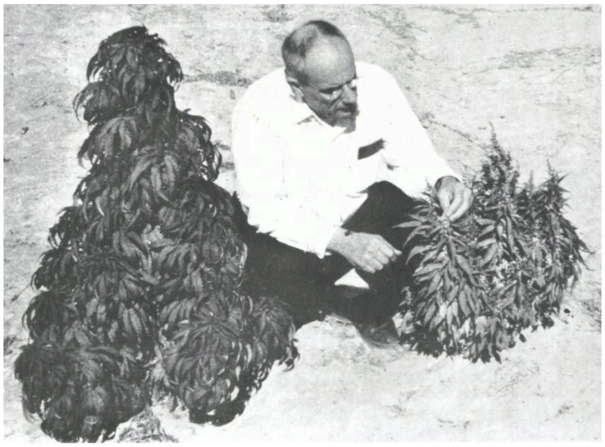

Figure S10. Pistillate plant (on left), source of herbarium specimen 26505 (ECON); photo from Schultes *et al.* (1974), courtesy of the Harvard University Herbaria and Botany Libraries.

Small and Cronquist (1976) considered the taxonomic system of Serebriakova and Sizov (1940) a “quasi-formal treatment… and appears to provide a useful, if artificial, guide to cultivars.” They noted that the Latin names coined by Serebriakova were not validly published, because the names lacked a Latin diagnosis or description.

Small (1975) analyzed morphological variation in the achenes of 399 herbarium specimens. He included accessions grown from drug seizures ($\bar{x}$=3.91 mm in length, n=39), wild-type specimens from India *and* Afghanistan ($\bar{x}$=3.21 mm, n=42), and wild-type specimens from Africa ($\bar{x}$=3.90 mm, n=25). Small *et al.* (1976) examined 232 world-wide accessions cultivated in a CGE. Their morphological analysis did not include Afghani plants. Clarke (1987) said no

Afghani accessions were present among herbarium specimens that Small sent to herb. NY. In fact, Small *did* cultivate Afghani plants, from achenes obtained in a *hashīsh* seizure (herbarium label, McPartl., pers. observ., herb NY, 2009). The Afghani plants did not progress past the vegetative stage, so Small omitted them from analysis.

Anderson (1974) measured xylem fiber cells (not phloem bast fibers of commerce). He compared an Afghan plant (the *afghanica* epitype designated herein) and a ruderal fiber-type plant from Kansas. Xylem cell length and width in the Afghan plant (0.281 mm and 18.41 μm) was significantly smaller than the fiber-type plant (0.443 mm and 14.3 μm). De Meijer (1994b) also measured xylem cell length and width. There was no significant differences between 105 accessions of fiber-type *C. sativa* ($\bar{x}$= 0.534 mm and 31 μm) and three accessions of drug-type Afghan plants ($\bar{x}$= 0.572 mm and 29 μm).

Anderson (1980) compared leaflet morphology in 60 herbarium specimens, grouped into four plant populations, of which only two concern us here: "*C. indica*" (drug plants from Afghanistan, *i.e., C. afghanica* herein) and "*C. sativa SS"* (***S***mall-***S***eeded drug plants from India and Pakistan, *i.e., C. indica* herein). He measured four aspects of leaflet morphology, taken from the central (largest) leaflet: width (W), length (L), L/W ratio (he used W/L, but we inversed it for consistency with other studies), and distance to the widest point along the entire length (WP/L).

"*C. indica*" length (mean 117.0 mm) was greater than "*C. sativa SS"* (78.8 mm), but not statistically significant. He reported significant differences in the L/W ratio: "*C. indica*" 5.49, "*C. sativa SS"* 10.64. He also found significant differences in the WP/L ratio: "*C. indica*" 0.565 (oblanceolate), "*C. sativa SS"* 0.426 (linear-lanceolate). Anderson also reported the number of leaflets per leaf, but he unfortunately lumped data from "*C. sativa SS"* and fiber-type *C. sativa* (mean 6.35), compared to "*C. indica*" (mean 8.20).

Clarke (1981) published a superb line drawing of a "Hindu Kush" plant. He gave its geographic range as Afghanistan and Pakistan. The plants matured early, and produced an acrid skunk-like smell; shoots 4-6 ft tall, thick, woody, with short internodes; leaves dark green with 5-9 leaflets, very broad and coarsely serrated; achenes large, round, and dark gray, with some mottling. Clarke highlighted morphological traits not described previously: Inflorescences "appear along the entire length of the primary limbs as very resinous leafy balls." Inflorescences have a low perigonal bract-to-leaf index, and floral leaves associated with the bracts are liberally encrusted with capitate-stalked glandular trichomes.

Cherniak (1982) provided 15 photos of Afghani plants. He described Afghani inflorescences as very compact, heavily laden with resin, and with perigonal bracts larger than "c. sativa strains." Plants rarely grew beyond 7-8 ft tall, and their branches seldom extended beyond 2 feet from the shoot. Shoots and branches were thicker than "c. sativa." Cherniak's photos show plants with broadly obovate leaflets, at least most of them. He noted that Afghani leaves have the "deepest, darkest green color of all cannabis plants."

De Meijer and Keizer (1996) compared morphological variation in 160 accessions under CGE conditions, and provided raw data (de Meijer 1994a). A multivariate clustering method (PCA) recognized four "plant-use groups": wild-type populations, fiber cultivars, fiber landraces,

and drug strains. In the latter group they lumped diverse accessions, such as hybrids (*e.g.*, "Skunk #1", "Four-way"), and accessions of questionable heritage (*e.g.,* "Nederweit"). Passport data and voucher specimens enabled us to identify three non-hybridized *C. indica* accessions (891384, 891385, 910972), and three Afghani accessions (883271, 891201, 891383).

Differences in only four traits approach statistical significance in this small data set. Leaf L/W ratio: Afghani, $\bar{x}$=3.16; *C. indica*, $\bar{x}$=4.68 ($p$=0.015). Achene marbling score: Afghani, $\bar{x}$=3.0 (moderate); *C. indica*, $\bar{x}$=5.67 (high) ($p$=0.016). Achene length: Afghani, $\bar{x}$=4.17 mm; *C. indica*, $\bar{x}$=3.80 ($p$=0.106). Xylem cell length: Afghani, $\bar{x}$=0.591 mm; *C. indica*, $\bar{x}$=0.548 mm (*t*-test $p$=0.090).

Hillig (2005b) compared 135 *Cannabis* accessions under CGE conditions, and scored them for 34 phenotypic characters. He grouped accessions into seven biotypes, including "narrow-leaflet drug" (NLD, *C. indica* herein, n=25), and "wide-leaflet drug" (WLD, *C. afghanica* herein, n=10). In his PCA scatterplot, ellipses encircling NLD and WLD accessions slightly overlapped. In his canonical analysis, the ellipses separated clearly.

Twenty-four of the 34 phenotypic characters showed significant differences between WLD and NLD accessions ($p \leq 0.05$). Here are some prominent ones: Height (m): WLD, 1.67; NLD, 2.53. Internode length, nodes 3 to 10 (mm): WLD, 109; NLD, 151. Leaflet length, node 7 (mm): WLD, 171; NLD, 188. Leaflet ratio (L/W) node 10: WLD, 4.55; NLD, 6.67. L/W node 7: WLD, 3.85; NLD, 5.88. L/W node 3: WLD, 2.56; NLD, 4.17. Leaf shape (WP/L ratio): WLD, 0.54; NLD, 0.49. Petiole length at node 10 (mm): WLD, 110; NLD, 77. Petiole width (mm): WLD, 2.74; NLD, 2.36. Petiole roundness in cross-section (width/thickness ratio): WLD, 0.97; NLD, 0.92. Number of primary serrations, node 10: WLD, 32.0; NLD, 39.1. Number of secondary serrations (*i.e,* biserrate subdivisions), node 10: WLD, 0.4; NLD, 12.4. Achene length (mm): WLD, 4.40; NLD, 3.95. Achene thickness (mm): WLD, 2.97; NLD, 2.50.

Hillig analyzed Afghani germplasm collected in the 1980s and 1990s. He commented, "Not everything from Afghanistan is Afghani" (Hillig, pers. commun., 2006). This is true regarding herbarium specimens from Afghanistan. Some resemble *C. indica* (or its wild-type *C. himalayensis*), rather than *C. afghanica* (or its wild-type *C. asperrima*). William Griffith collected "*Cannabis*" (no species name) near Jegdalek, between Kabul and Jalalabad (Griffith 1847). One of his herbarium specimens has narrow leaflets (L/W = 9 to 11), a male plant ("Affghanistan," no location, *Griffith*, K). Another has broad leaflets (L/W 3.5 to 5) and wild-type achenes (No location, "Herbarium of the late East India Company," *Griffith 4686,* K). Some Vavilov specimens collected in the heart of *C. afghanica* territory represent intermediate forms between *C. indica* and *C. afghanica* (see list of herbarium specimens).

**SF8. Morphological comparisons, cont.**
**Central Asian *C. afghanica* (domesticated) and *C. asperrima* (wild-type)**

Wild-type *C. asperrima* has been collected from Kyrgystan, Kazakhstan, Afghanistan, Uzbekistan, Tajikistan, and Xīnjiāng Region (Fig. 7 in main document). We present brief histories of *Cannabis* in these regions, which provide inferential data regarding the origin of

cultivated plants that may have de-domesticated into wild-type plants. Early historical accounts also provide data regarding the geographic range of *C. afghanica* and *C. asperrima.*

**Kyrgyzstan**

The Kyrgyz people became Islamicized in the 1630s and 1680s, and a fossil pollen study near Issyk Kul shows a surge of *Cannabis* pollen about a century later (Beer and Tinner 2005). Levitov (1909) stated that Kyrgyz people smoked нашу (*nasha*). UN-ODC (2008) noted that wild-type plants in two regions had relatively high THC content: in Issyk-Kul (where botanists collected *asperrima*) and in Jalal-Abad (part of the Ferghāna Valley, a center of 19th century *hashīsh* production). Farmers in the Issyk-Kul region have supplemented their income by hand-rubbing *hashīsh* from wild-type plants (Botoeva 2015).

Eduard Regel assigned the name *C. sativa γ asperrima* to plants collected near Lake Issyk-Kul. His brief description is translated in section SF.6. The plants were collected by his son, and two specimens exist herb. LE. The plants are short (75-90 cm) and branchy, with relatively large inflorescences, dense with glandular trichomes. The specimens were not adequately pressed, and poorly preserved—leaflets are shriveled, fragmented, and difficult to measure (Fig. 6A in main document). The achenes are small (2.8-3.3 mm long), with an elongated base, and a mottled perianth (Fig. 3J in main document).

After Regel coined *asperrima,* reports of wild-type growth near Lake Issyk-Kul were published by Krassnoff (1887) and Roborovsky (1890). Two herbarium specimens by Krassnoff were collected at Karakol ("Przhevalsky") and Uital (destroyed by earthquake in 1889). Both plants have broad leaflets (up to L/W = 4.1). The Karakol plant is male, the Uital plant is female, with a fairly dense inflorescence, achenes are immature but with a persistent perianth, protuberant base, 3.0 mm long (herb. LE). Roborovsky's specimen came from Barskoünski Pass, southeast of Issyk-Kul. The plant is stunted (30 cm tall), with little branching; leaflets are dark and broad (up to L/W = 3.0), a male specimen (herb. LE).

**Kazakhstan**

Peter Simon Pallas explored Siberia between 1768 and 1774, and penetrated Kazakhstan near Semipalatinsk (now Semey), where he found "many wild hemp" (Pallas 1794). We have seen three of Pallas's herbarium specimens (herb. LE, BM). They have no annotated locations, and lack *asperrima* morphology. In Siberia bordering Kazakhstan, Pallas (1794) wrote about indigenous people and Russian colonists growing fiber-type hemp.

Siberia is clearly out of *asperrima* range. Small and Cronquist (1976) classified Siberian hemp under *C. sativa* subsp. *sativa*, either domesticated or wild-types. They included Siberian plants from the Altai Mountains. Hillig (2005a) analyzed four wild-type accessions obtained from the Central Siberian Botanical Garden (including one from the Altai). In a PCA scatterplot of genetic variation, the wild-types overlapped with accessions of domesticated fiber-type plants from Siberia (Tyumen) and European Russia (Kirov, Perm, Mari).

Karelin and Kiriloff collected wild-type plants in Kazakhstan, from 1840 to 1845, and distributed excissati specimens to herbaria worldwide. Printed labels on their excissati read, "*In pratensibus ad fl. Irtysch frequens* [frequently in meadows near Irtysch River], *nec non in deserti*

*Soongoro-Kirghisici* [and certainly on the Kirghiz steppe], *arenosis ad lacum Noor-Saissan* [growing on sand near Lake Zaysan].” These lines were lifted by De Candolle (1883) when he described the native range of *C. sativa*.

Karelin and Kiriloff’s excissati specimens (herb. LE, BM, K, NY) vary in morphology, from small stunted plants to large plants with robust inflorescences—they were collected in different locations. They are largely consistent with naturalized *C. sativa*; fiber-type hemp was cultivated in Kazakhstan. Russians founded Orenburg near the border of Kazakhstan, and began cultivating hemp (Nartov 1767). Russians settled East Kazakhstan by at least the 1850s (Semenov 1998), and by 1881 they produced 53 tons of hemp fiber in the region (Lansdell 1885).

The mountains in southern Almaty Region, near the Kyrgystan border, harbored populations of *C. asperrima*. Pyotr P. Semenov collected wild-type plants in Trans-Ili Alatau, near its southeastern edge, as he climbed north from the Chilik River (Semenov 1998). His herbarium specimen (herb. LE) is annotated “4000-7000 ft.” The specimen is a stunted plant, 35 cm tall, very branchy, with dense inflorescences. Leaflets are small, broad (L/W = 3.9), oblanceolate, with coarse serrations, and dark green colored. Achenes have a protuberant base, a mottled and persistent perianth (Fig. 3L in main document), 3.0–3.2 mm long. Other specimens from the Trans-Ili Alatau also exhibit *asperrima* morphology (see list of herbarium specimens).

To the west of Almaty Region lies the Chuy Valley, a huge refuge for wild-type *Cannabis*. The weed is psychoactive; illicit harvesting began in the 1960s, and authorities began eradicating it in the 1970s (Nikitin 2014). Estimates of THC content vary, usually around 1% (see section SF.9). Reported acreage in the Chuy Valley varies from 125,000 ha (Tiourebaev *et al.* 2001) to 400,000 ha (Seshata 2014a). Seshata claimed that local authorities discovered “Indian hemp” cultivated in Chuy Valley by 1926, and the wild hemp is a hybrid between local landraces and imported germplasm. Kazakh researchers referred to the plants as “*C. sativa* var. *afghan*” (Anonymous 1994).

Wild-type plants in Chuy have introgressed with fiber-type hemp. Russians established the Chuy Bast Crops Experiment Station on the outskirts of Bishkek in 1932. They grew Italian hemp (L’dov 1933, Belotsky 1936) and Japanese hemp (Anonymous 1963). Many herbarium specimens from the Chuy Valley consist of either fiber-type hemp, or wild-type plants consistent with naturalized fiber-type hemp. One specimen is labeled “Japanese hemp” (Jambyl Region, Georgievskoe State Farm, *Cherniakovskaya* 1937 (LE)).

**Afghanistan**

“Afghanistan has the oldest *hashīsh* culture still in existence today” (Clarke 1998). Perhaps the oldest record of *Cannabis* in Afghanistan is a poem written in Herāt in the 1430s, *Bang-u chagir, “Bhāng and wine”* (Roxburgh 2005).

After Vavilov blazed a trail through Kāfiristān (now Nuristān), several groups followed. A German expedition travelled there in 1935, and returned with germplasm of plants with dense branching and small achenes. Hoffmann (1961) said they were consistent with Vavilov’s *afghanica* description. A Danish team went to Nuristān in 1956. They collected two *C. sativa* specimens, at “the edge of an irrigation ditch near Tshaghan Serai, lower Pech valley at the

mouth, about 820 m, and one by Mühlbach at Barikot, 850 m" (Gilli 1963). "Tshaghan Serai" is Asadābād, and Barikot is near the Bashgal River, where it joins the Kunar River. Mühlbach's research was cited in an Afghanistan vegetation survey; of six surveyed sites across Afghanistan, Barikot was the only location with wild-type *C. sativa* (Ceška and Roemer 1971).

The 1965 Chicago Field Museum Expedition reported *C. sativa* growing near "Chigha-Sarai" (Asadābād), and along watercourses near Kāmdēsh in Nuristān (Hassinger 1968). Janice Street collected three specimens in Kāmdēsh. The plants are small (they fit on herbarium sheets) and branchy; inflorescences are small and relatively loose, with ample glandular trichomes, even on floral leaves. Leaves are dark green, with 5-7 leaflets; leaflets medium sized, up to 85 x 19 (L/W = 4.5), rarely oblanceolate in shape. Achenes have an olive-brown mottled perianth; achenes are small ($\bar{x}$= 2.75 mm, range 2.3–3.0), with a protuberant base.

Four years later, Tom Hewer, a British medical professor, collected *Cannabis* at Kāmdēsh. The label on his herbarium specimen (herb. LE) reads, "One of the commonest 'weeds' grows everywhere." The specimen is small (fits on the herbarium sheet), with broad, overlapping oblanceolate leaflets, a male plant. A French expedition in Nuristān described wild *C. sativa* in the middle Kunar River valley around Asadābād. In the Bashgal River valley (between Kāmdēsh and Barikot), "it sometimes forms almost pure populations on the slopes along the road" (Hayon *et al.* 1970).

Small (1975) examined Janice Street's specimens in 1975, and annotated them as *C. sativa* subsp. *indica* var. *kafirsistanica*. He photographed an achene from Street's collection. We provide a photo from the same Street specimen (Fig. 3K in main document). Small and Cronquist (1976) assigned all drug-type plants with wild-type traits to the taxon *C. sativa* subsp. *indica* var. *kafiristanica*. They included accessions from Afghanistan, the Himalaya, China, and naturalized plants that re-acquired wild-type traits in South Africa and Columbia.

Breckle and Koch (1982) published "Afghan drugs and their ancestral plants," based on their field work. Wild-type plants grew along roadsides and streams in Nuristān, Badakhshān, and Pakyā provinces, and rarely elsewhere in Afghanistan. A close-up photograph of plants near Kandahar shows dark-covered leaves with broad, oblanceolate leaflets. A plant near Nuristān has lighter-colored leaves, whose leaflets are narrower. Plants in Kabul and Helmand are farther away in the photos; they are bushy and <1 m tall. The photos are reproduced in Breckle and Rafiqpoor (2010), who describe *Cannabis* as "an extremely polymorphic species."

**Uzbekistan**

Uzbekistan has a tradition of both fiber- and drug-type *Cannabis*. An Arab geographer mentioned Samarqand as a source of hemp paper and rope in 982 CE (Levi and Sela 2010). Two years later, Al-Muqaddasī (1994) mentioned hemp rope exported from Bukhārā and Samarqand.

In the 1660s, three French travelers in Persia described "Uzbeks" visiting Isfahān, and smoking *charas*—(*i.e.,* sieved *hashīsh*)—the first people in recorded history to smoke *Cannabis,* rather than eat it. Du Mans wrote in 1660, "Uzbeks, people of minor Tartary" taught Persians how to smoke "leaves of hemp seed" (Du Mans 1890). Persians, in contrast, still drank *bhāng.* Tavernier (1676) said Uzbeks introduced *tchouhersse* (*chars* or *charas*) into Isfahān. Chardin

(1686) wrote, "The Uzbeks have found out a way to take the smoke of that [hemp] seed, mixed with tobacco; and they have brought the mode of it into Persia."

An ambiguous account suggests earlier Uzbek *hashīsh* usage: Bukhārā was the largest city in Central Asia until Genghis Khan destroyed it in 1218. Bukhārān refugees fled west, ahead of Mongol armies, where the Damascus cleric Ibn Taymīyah (1263-1328) wrote, "About the time of the appearance of the Tatars [Mongols], *hashīsh* went forth, and with it went forth the sword of the Tatars" (Rosenthal 1971).

The Russian explorer Falck (1786a) reported that *wilder hampf, C. sativa,* grew in Bukhārā and "Soongaria" (Dzungaria). Falck (1786b) said Bukhārāns grew some *Cannabis* for rope, "and particularly for the intoxicating female flowers (*bang*)." Tax collectors in British India monitored *charas* coming from the Khanate of Bukhārā (Postans 1841, Davies 1862). The Russians invaded Bukhārā in 1866. They criticized *nasha* cultivation and consumption (Grebenkin 1872, Brodovskogo 1872, Fedchenko 1875), and completely suppressed its production (Clarke 1998). Suppression is supported by fossil pollen studies, which show a decrease in *Cannabis* pollen after the 1870s (Beer and Tinner 2005, Beer 2007).

Herbarium specimens from Uzbekistan vary in morphology. They run a gamut from domesticated *afghanica*, through intermediate forms, to wild-type *asperrima* (see list of herbarium specimens). Seshata (2014b) wrote, "*C. indica* sp. *afghanica* is the type that is dominant in Uzbekistan; it is thought to have evolved in the region straddling southern Uzbekistan, Tajikistan and northern Afghanistan… It differs from the type found in northern India, Pakistan and Nepal—this type (*C. indica* sp *indica*) actually has narrow leaves."

**Tajikistan**

Tajikistan's main ethnic group, the Tajiks, live in the Ferghāna Valley and upper Zeravshān River of Sughd Province. The Pamiri people live in the Pamir Mountains of Gorno-Badakhshan Region. Camp (1936) proposed that *Cannabis* evolved in the Pamir Mountains.

The Tajiks trace their ancestry to Sogdians; Sughd Province is a corruption of Sogdiana. The Sogdians may have descended from the Scythians (Szemerényi 1980). The Sogdian word for hemp, *kynp'*, resembles the Scythian word **kanap* (Witzel 2006). The Zeravshān River in Tajikistan flows into Samarqand and Bukhārā in Uzbekistan, which were originally Sogdian cities, conquered by Turkic Uzbeks.

In the Tajiki language, *nasha* (*naša*) means *hashīsh* or marijuana. *Nasha* has an identical meaning in the Uzbek language (Central Asian Heritage Group 2005). Tajiki is an Iranian language and Uzbek is a Turkic language, so a loan occurred one direction or the other. Steblin-Kamensky (1982) connected Tajik *nasha* with the older Middle Persian word *šan,* "hemp." This suggests the loan went from Tajiki to Uzbek. The Tajiks—an agricultural people—likely introduced the word *nasha,* as well as *nasha* itself, to the Uzbeks—a nomadic people.

French travelers in Isfahān who described "Uzbeks" smoking in the 1600s may have confused Tajiks for Uzbeks. The "Uzbeks" in Isfahān were diplomates from Bukhārā, and most of the administrators in Bukhārā were Tajiks (Subtelny 1994). Tajiks and not Uzbeks were the

big smokers in Bukhārā, as documented by Russian authors (Grebenkin 1872, Rosen 1896, Ostroumov 1896).

The Russians invaded Tajikistan, and wrote about *nasha* in the Ferghāna Valley (Middendorf 1882, Kushelevskiĭ 1891). Muminjanov (2008) listed *Cannabis* as an "indigenous" crop in Tajikistan, specifically *C. indica,* curiously designated a "fiber crop." Vavilov nearly made that mistake when he explored the Pamirs in 1916, "In some villages there are stands of hemp among the fields of cotton and along the fences. At first I thought that this hemp was used for twine. Later it became evident that the hemp was sown to obtain *hashīsh*" (Vavilov 1997).

Schultes *et al.* (1974) erroneously equated *C. ruderalis* with an herbarium specimen from Tajikistan, collected by Ul'ianova in 1969. The plants are small and stunted (three fit on an herbarium sheet), unbranched or branched; inflorescences small and loose; leaflets small, up to 40 x 12 mm (L/W = 3.3), slightly oblanceolate. Achenes have a light brown mottled persistent perianth; they are small, 3.0 mm long, with a broad protuberant base (herb. ECON). This morphology is inconsistent with Janischevsky's taxon, and more consistent with *C. asperrima*.

Ul'ianova collected his specimen in Sughd Province. Other herbarium specimens consistent with *C. asperrima* come from Sughd Province or Gorno-Badakhshan Region. A specimen lacking wild-type traits, consistent with *C. afghanica*, was also collected in Sughd (see list of herbarium specimens).

**Xīnjiāng Region**

According to *Flora of China*, "native or naturalized" populations of *C. sativa* in China are limited to Xīnjiāng Region (Zhou and Bartholomew 2003). *Flora of China* omitted mention of wild-type growth elsewhere in China, whereas Yu (2013) and Zhang *et al.* (2018b) included the Tibetan Plateau. Clarke (1999) and Zhang *et al.* (2018b) included Yúnnán.

The topography of Xīnjiāng can be described as "three mountains and three basins" (Fig. S11). The mountains from north to south are the Altai, Tiān Shān, and Kūnlún. The Tiān Shān separates the Dzungaria and Turpan basins (in the north) from the Tarim Basin (in the south). The Kūnlún separates the Tarim from Tibet.

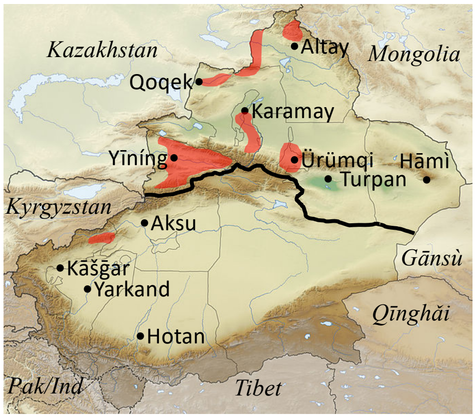


Figure S11. Xīnjiāng topographical map. The black line separates Dzungaria and Turpan basins from Tarim Basin. Red areas demarcate regions with wild-type *C. sativa* from Fu (2000). Base map courtesy of Wikipedia Commons.

In *Higher Plants of China* (Fu 2000), a plant distribution map showed that most wild-type *Cannabis* grew in Dzungaria: the Yīníng Basin (upper Ili River Valley), Altai Mountains (north of Altay), Irtysh River Basin (west of Altay), the Tarbagatai range near Qoqek (Tăchéng), the

Ürümqi oasis, and the steppe south of Karamay (Fig. S11). The Tarim and Turpan basins are mostly desert, and wild-type *Cannabis* was limited to a patch between Kāšğar and Aksu.

Tombs at Yánghǎi and Jiāyī in the Turpan Basin show unique evidence of cannabis drug use, with a calibrated age range of 800-520 BCE. Whether the plants were domesticated (*C. afghanica*) or wild-type (*C. asperrima*) is difficult to discern from photographs of achene morphology (Table S3).

Jiang *et al.* (2006) discovered *Cannabis* in a tomb in Yánghǎi. The leaves were reduced to fragments, which limited morphological data; petioles were furrowed, 5.4-15.4 mm in length; bracts were covered with cystolith trichomes and glandular trichomes, either sessile (80%) or stalked (20%); the gland head were very small (diameter $\bar{x}$= 37.4 µm). In a tomb at Jiāyī, the occupant was interred with a bundle of 13 female *Cannabis* plants laid atop him, like a shroud (Jiang *et al.* 2016). The plants were compressed and broken; petioles measured 3.7 to 23.6 mm long. Leaflets had coarsely serrated margins and acuminate tips. One nearly-intact leaflet showed a broadly lanceolate shape, possibly oblanceolate, but the tip is missing. Accounting for the missing tip, L/W = 4.6 can be estimated.

Muslim Uyghurs in the Tarim Basin were (and still are) *nasha* smokers. *Nasha* likely spread to the Tarim, along with Islam, from Samarqand and Bukhārā. According to *Tārīkh-i-Rashīdī,* written in the 1540s, when Pīr Muḥammad Barlās of Samarqand was appointed Governor of Kāšğar in 1448, the people of Kāšğar gave him the nickname *Bangí* (Mirza Muhammad Haidar 1898). Forsyth (1875) explained the nickname, "from his constant intoxication by the drug called *bang.*" A Sūfī from Bukhārā named Ahmad Kāsānī (1462–1542) spread the Naqshbandī order to Kashgaria (Babadijanov 1999). The followers of Kāsānī's grandson, Āfāq Khwāja, were *nasha* smokers (Waite 2006).

Tons of *nasha* from Kāšğar and Yarkand were shipped to British India through the 1800s until 1934. Between 1837 and 1839 alone, the amount of *nasha* exported from Yarkand increased from 8020 kg to 8990 kg (Cunningham 1844). Herbarium specimens from the Tarim and Turpan basins usually represent *afghanica,* or wild-type *asperrima* (see list of herbarium specimens).

Dzungaria, in contrast to the Tarim Basin, has a recent history of fiber-type hemp cultivation. Chinese troops conquered Dzungaria in 1755-1758. The economist Lù Fú'ēn called for colonizing Dzungaria with Chinese farmers in 1840, so that "mulberry and hemp [can] cover the countryside" (Lavelle 2012). Petzholdt (1874) mentioned hemp in Dzungaria, and Vámbéry (1874) reported a paper-making industry in Kuldja (*i.e.,* Yīníng). Lansdell (1885) also mentioned a paper factory in Kuldja that used hemp. In 1890 Katanov saw hemp crops near Ürümchi, "The Chinese produce oil from hemp and sesame" (Martynov and Martynova 2015). *C. sativa* was cultivated in the "Chinese town" of Sharasume, present-day Altay, and wild-type plants grew nearby (Price and Simpson 1913); their herbarium specimen is a robust fiber-type plant, with narrow leaflets and small achenes (Sharasume, *Price*, 1910, K).

Herbarium specimens from Dzungaria often resemble Siberian hemp, similar to specimens collected in the Altai and Irtysh Basin in northeastern Kazakhstan. Examples include: Altay

Prefecture, Qinghe County, *Qin Renchang,* 1956 (PE 00557980); Altay Prefecture, Fuhai County, *Dong Ailing,* 1996 (PE 00761677); Tacheng Prefecture, Yumin County, *Lin Yourun,* 1974 (PE 00557981); Bortala Prefecture, Bole City, *Guan Kezhen,* 1957 (PE 00557993); Changji Hui Prefecture, Manas County, *Guan Kezhen,* 1957 (PE 00557989); Ili Prefecture, Gongliu County, *Lin Yourun,* 1974 (PE 00557987); Ili Prefecture, Altay City, *Chen Jiarui,* 1986 (PE 19860825); Ili Prefecture, Huocheng County, Yang Wei, 1989 (PE 00761676).

Some specimens collected in the Tiān Shān mountains of southern Dzungaria are more consistent with wild-type *asperrima* (see list of herbarium specimens). Vavilov (1931) stated "*C. sativa* var. *spontanea* is a very common plant in northern Tiān Shān, especially on north-facing slopes and valleys." The map of his expedition indicates he was in the Dzungarian Alatau. An herbarium specimen collected on north-facing slopes of the Dzungarian Alatau 60 years later was consistent with *C. asperrima* (Fig. S12).

Figure S12. Herbarium specimen collected in the Dzungarian Alatau by Morefield in 1989 (GH).

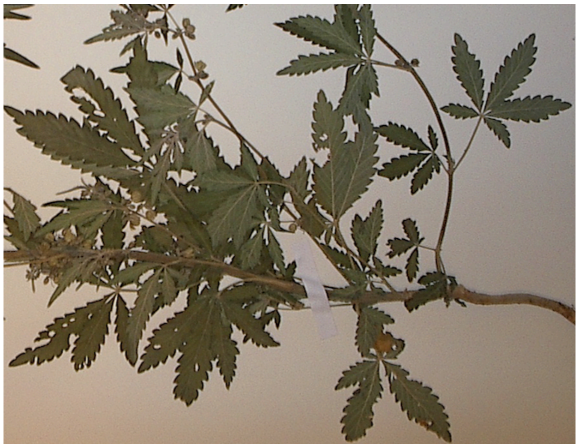

**Tibetan Plateau**

The Tibetan Plateau, with a mean altitude of three miles, is surrounded by mountain ranges that are even higher. Tibetan people, native to the Tibetan Plateau, speak Tibetic languages. There are three main dialects, spoken in three Tibet provinces, now divided into administrative areas within China: 1. The Ü-Tsang dialect corresponds to present-day Tibet Autonomous Region (officially Xīzàng Region). 2. Amdo corresponds to Qīnghǎi Province. 3. Kham is divided between eastern Xīzàng Region and western Sìchuān Province, with small pockets in Qīnghǎi and Yúnnán provinces.

*Cannabis* in the Tibetan Plateau has not received much attention, ironically, because *Cannabis* evolved in either northeastern Amdo (McPartland *et al.* 2019) or southeastern Kham (Zhang *et al.* 2018b). Agriculture began early in Amdo (Qīnghǎi), which encompasses the upper reaches of the Yellow River. *Cannabis* achenes have been recovered from Bronze Age Qíjiā Culture sites in eastern Qīnghǎi (Zhang 2013, Yang 2014).

Wild-type populations in the Tibetan Plateau may represent indigenous populations, or naturalized escapes of East Asian (Chinese) hemp. Yu (2013) reports wild-type populations along the banks of the Cháyú and Bōmì rivers (tributaries of the Brahmaputra River, in Kham),

and along the Jīnshājiāng (upper Yangtze River, the eastern border of Kham). Zhang *et al.* (2018b) genotyped wild-type populations from undisclosed locations in Tibet and Qīnghǎi. Tibetan plants shared haplotypes with accessions from Yúnnán, Inner Mongolia, and Xīnjiāng. Qīnghǎi plants shared haplotypes with accessions from the same places, plus a unique haplotype.

Most herbarium specimens from Tibet resemble East Asian hemp (domesticated or wild-type), or their stunted morphology cannot be characterized. Examples include: Tibet, Hasora Province, *Schlagintweit*, 1856 (GW). Tibet, Nyingchi, Kongbo, Shoga Dzong, 10,500 ft, *Ludlow et al.* 1947 (BM). Tibet, Nyingchi, Bayi town, *Boufford*, 2000 (GH). Tibet, west of Latung, 10,000 ft, *Chapman*, 1936 (K). Tibet, near Lhasa, Chakzam Bridge, *Walton*, 1904 (K). Tibet, Chayu County, near Xiachuan, *FLPH Tibet Expedition,* 2012 (PE 01967717).

One herbarium specimen resembles South Asian *Cannabis* (Xīzàng Region, Chumbi Valley, *Rolunoo Lepcha* 1912, GH). Unlike the rest of the Tibet, the Chumbi Valley lies on the south side of the Himalaya, tucked between Sikkim and Bhutan. Exploring near Gyantse, north of the Chumbi Valley, Waddell (1905) described "Rank plants of Indian hemp, 6 feet high, and thorn apple grew luxuriantly amongst the tall docks and nettles in neglected corners." He records the altitude as 12,000 feet above sea level, but Gyantse is actually 3977 m (13,050 ft). Either way, this holds the record for the highest *Cannabis* in the world.

## SF.9. Phytochemical comparisons
## South Asian *C. indica* versus Central Asian *C. afghanica*

Before analytical instrumentation existed, the unique qualities of Central Asian cannabis products were noted by explorers in that region:

- Chardin (1686) said that *bueng* in Persia [Central Asia] was stronger than *bueng* in India [South Asia], but Persians may have added opium and nux vomica to their *bueng*.
- Elliot (1845) wrote, "The *charas* of Bokhara [Central Asia] is most admired, and fetches double the price of the country product [of British India]."
- According to Royle (1847), "The *churrus* of the Himalayas is much esteemed, that of Herat [Afghanistan] and Yarkund [Xīnjiāng Region] still more so."
- Vámbéry (1868), a British-Hungarian explorer who travelled widely between 1858 and 1864, stated that Egyptian *hashīsh* and drugs in Constantinople and Persia were "nothing in comparison with the *bengis* of Central Asia."
- Valikhanov (1865), a Russian-Kazakh explorer who travelled throughout Central Asia between 1847 and 1865, wrote that Kāšgar (Xīnjiāng Region) was "celebrated for the *nasha* it produces."
- Masson (1844) described what now seems equivalent to the alleged "couchlock" quality of Afghani cannabis. While journeying from Afghanistan (Central Asia) to Balochistan (South Asia), his group encountered a roadblock of Balochi bandits. "The Afghans waggishly filled the chillam with [Afghani] *chirs*, and the Balochis, unaccustomed thereto, as if by enchantment, fell asleep.

Hooper (1908) made an early analytical comparison of *charas* from South Asia and Central Asia. He used “percent resin” as a proxy for cannabinoids, and “volatile percentage” as a measure of terpenoid content (Table S10). Means calculated from Hooper’s data show that “percent resin” in Central Asian samples was significantly greater than South Asia samples (unpaired $t$ test, $p = 0.02$). Of course, several variables may explain this difference—source plants, manufacturing methods (sieved vs. rubbed *charas*), time in storage, and adulteration.

Table S10. Resin percentage (~cannabinoids) and volatile percentage (~terpenoids) extracted from *charas* samples with provenance from either Central Asia or South Asia (Hooper 1908).

| Central Asian provenance | | | South Asian provenance | | |
|---|---|---|---|---|---|
| source | resin % | volatiles % | source | resin % | volatiles % |
| Kāšġar No. 3 | 48.1 | 12.0 | Nepal | 44.6 | 5.6 |
| Amistar “M”-1 | 46.5 | 3.6 | Nepal “Shah” | 44.4 | 4.2 |
| Amistar “M”-2 | 42.7 | 5.8 | Gwalior | 43.3 | 3.1 |
| Deli dust R1 | 42.6 | 4.3 | Garhwal | 41.9 | 7.7 |
| Deli dust 12as | 42.4 | 4.0 | Simla | 37.0 | 9.4 |
| Deli Mashak | 41.1 | 7.4 | Almora | 36.9 | 7.5 |
| Kāšġar No. 2 | 40.9 | 12.4 | Kumaon (cult.) | 34.2 | 7.5 |
| Kāšġar No. 1 | 40.2 | 12.7 | Baluchistan No. 2, 1905 | 26.0 | 9.3 |
| Yarkhand | 40.0 | 6.5 | Baluchistan No. 3, 1905 | 24.9 | 10.5 |
| Amistar Bhara | 38.1 | 6.4 | Kumaon (wild) | 22.3 | 9.1 |
| Bombay | 36.1 | 4.6 | Baluchistan No. 1, 1905 | 22.0 | 7.2 |
| $\bar{x}$, SD | 41.7 ±3.42 | 7.25 ±3.49 | $\bar{x}$, SD | 34.3 ±9.02 | 7.37 ±2.29 |

A pair of Eli Lilly pharmacologists, Eckler and Miller (1912), *may have* compared South Asia and Central Asia germplasm in the dog ataxia test. They obtained germplasm from British India, which included present-day India (South Asia) and Pakistan (sometimes included in Central Asia). The germplasm yielded two phenotypes. One consisted of short (1-2 feet tall), early-maturing plants with heavy, compact, leafless, and resinous pistillate flower clusters (which suggests a Central Asian phenotype). The other consisted of taller (3-7 feet tall), later-maturing plants with smaller inflorescences (which suggests a South Asian phenotype). Physiological potency was gauged against a commercial extract of *C. indica* cultivated in British India, with a potency standardized as 100%. Tall plants were more potent than short plants, 50% and 40% respectively.

**SF.9. Phytochemical comparisons, cont.**
**THC/CBD ratios in the four varieties**

Hillig and Mahlberg (2004) conducted the only CGE that measured cannabinoid content in significant number of accessions from all four varieties (Table S11). They analyzed 253 individual plants from 96 accessions. Means for each accession were obtained from individual

plants (two or three plants per accession). In contrast, previous researchers quantified cannabinoid content within each accession by mixing bulked samples for a single measurement. Hillig and Mahlberg conducted a CGE with accessions collected in the 1970s-1990s, and did their best to remove hybridized accessions from their studies. Inspecting their voucher specimens (at herb. IND), however, indicates that a few hybrids snuck into their analysis. For instrumentation they used capillary GC-FIDs, with elution peaks and retention times calibrated with standards for THC, CBD, CBC, and CBG.

Table S11. Some results by Hillig and Mahlberg (2004), means not connected by the same letter are significantly different using Student's t test (P ≤0.05). THC/CBD ratios calculated from their data are added to the bottom row.

| | WLD biotype *C. afghanica* n = 40 | NLD biotype *C. indica* n = 68 | *C. indica* feral *C. himalayensis* n =14 | East Asian hemp n =45 | European hemp n =62 |
|---|---|---|---|---|---|
| THC | 6.49%,a | 5.48%,a | 3.04%,b | 3.54%,b | 1.16%,c |
| CBD | 1.21%,c | 0.02%,d | 1.95%,bc | 1.43%,bc | 4.01%,a |
| THC+CBD | 7.70%a | 5.50%,b | 4.99%,bc | 4.97%,bc | 5.17%,bc |
| THCV+CBDV | 0.14%,bc | 0.25%,b | 0.90%,a | 0.19%,b | 0.05%,c |
| CBG | 0.19%,ab | 0.24%,a | 0.22%,ab | 0.18%,ab | 0.14%,b |
| CBC | 0.17%,b | 0.19%,b | 0.18%,ab | 0.34%,a | 0.18%,b |
| CBGM | 0.02%,b | 0.01%,c | 0.00%,c | 0.05%,a | 0.01%,bc |
| THC/CBD ratio | 5.36 | 274.0 | 1.56 | 2.48 | 0.29 |

***C. afghanica***

Several studies included accessions from Central Asia (*C. afghanica* herein) that predated the rise of widespread hybridization. Study numbers 1-13 (in unitalicized red) were applied to Fig. 2 in the main document.

1. Jenkins and Patterson (1973) used GC-FIDS to measure THC+CBN/CBD in seizures of Afghani resin, n=4, THC/CBD ratio $\bar{x}$= 1.84 ±0.258 SEM (range 1.21 to 2.46). Resin from Pakistan may have been of Afghani origin (n = 19, $\bar{x}$ =1.07), which departed from their ratios reported from South Africa, Jamaica, and Nigeria ($\bar{x}$ =10.8, 10.0, and 9.7, respectively).

2. Holley *et al.* (1975) used GC-FID with silyation to remove CBC from THC/CBD. They conducted a CME in Mississippi, but note "some cross-pollination has occurred." Afghanistan B ($\bar{x}$ of female and male, 0.94), Afghanistan B1 ($\bar{x}$ of female and male, 5.79), total $\bar{x}$ = 3.37.

3. Coffman and Genter (1975) used GC-FID with silyation to remove CBC from THC+CBN/CBD. They conducted a CME in Maryland with a single Afghanistan accession grown in 11 different soil types, $\bar{x}$ = 3.0 ±0.48 SEM (range 1.1 to 6.1).

4. Mobarak *et al.* (1978) used GC-MS to analyze *hashīsh* from "Kandeh in Petschtal, 1300 m a.s.l." That would be Kandai, Pech River valley, central Nuristān, Afghanistan. They report a THC+CBN/CBD ratio of 1.93.

5. Idilbi *et al.* (1985) used GC/FTIR to measure cannabinoids in a number of drug samples, including two from Afghanistan, with THC/CBD ratios of 1.8 and 1.9.

6. Martone and Della Casa (1990) used GC-FID to analyse THC+CBN/CBD in *hashīsh* seizures: Afghanistan (2.78), Pakistan (1.00), India (3.07), Iran (4.03).

7. De Meijer *et al.* (1992) used GC-FID to analyse THC/CBD in 97 accessions in a CGE in Holland, and provided raw data (de Meijer 1994a). As mentioned previously, passport data and voucher specimens enabled us to identify three Afghani accessions: 883271, ratio 0.72; 891201, ratio 1.40; 891383, ratio 5.12; $\bar{x}$ = 2.41.

8. Zhang and He (1992) used GC/MS to analyse nine seized samples from the western Tarim Basin in Xīnjiāng (Hotan, Kāšğar, Kizilsu prefectures). There's appreciable CBN and $\Delta^8$-THC content. They reported cannabinoids as percentages of total cannabinoid content. The mean $\Delta^9$-THC+$\Delta^8$-THC+CBN/CBD ratio = 0.73 ±0.10, range 0.25 to 1.32.

9. Liu and Shang (1992) used GC-FID to analyze five *Cannabis* plants from Ürümqi, the capital of Xīnjiāng Region. They report peak sizes, rather than w/w percentages. The mean THC+CBN/CBD peak size ratio was 1.94.

10. Cao *et al.* (1993) used GC-FID to analyse two seized samples from Korla, the second largest city in Xīnjiāng Region. They report peak sizes, rather than w/w percentages. The THC+CBN/CBD peak size ratios were 1.26 and 1.08.

11. Hillig and Mahlberg (2004) used GC-FID to analyze 40 WLD accessions, and report means of THC 6.49% and CBD 1.21%, from which we derive a THC/CBD ratio of 5.36.

12. de Meijer *et al.* (2009) used GC-FID in a CME in Holland, and measured THC and CBD as a percentage of total cannabinoid content. Their breeding lines include a non-inbred clone of an "Afghanistan hashish landrace," and an inbred line of the same. Their THC/CBD ratios were 0.07 and 0.04, respectfully.

13. Mansouri *et al.* (2011) used HPLC to measure THC/CBD in wild-type plants of Iranian provenance, prior to flowering, $\bar{x}$ =2.4

Additionally, three studies determined THC/CBD ratios in archaeological materials. Russo *et al.* (2008) tested a sample from Yánghǎi, Xīnjiāng Region, dated 630 ±95 cal. BC (Beck *et al.* 2014). Analysis with HPLC and GC-MS showed that cannabinoids had degraded into breakdown products, at low concentrations. They quantified CBN only, present at 0.7%. However, Russo's chromatogram illustrated several peaks, from which a THC/CBD ratio can be estimated by measuring peak areas: CBN + CBN-OH + cannabitrol / CBD + cannabielsoin, a ratio of 2.24.

Ma *et al.* (2011) analyzed the same material, using HPLC-MS. They report THC 0.110% and CBD 0.106%, a ratio of 1.04. Ren *et al.* (2019) used GC-MS to analyse a *Cannabis* sample from Jiāyī, about 30 km from Yánghǎi, dated 790-520 cal. BC, with a CBN/CBD ratio of 9.5, estimated from peak areas on their chromatogram. They also analyzed pyrolytic residues from sooty braziers unearthed at Jirzankal, Xīnjiāng Region, 500 cal. BC. They inexplicably detected only CBN, and no CBD.

***C. indica***

Most studies analyzed plants of South Asian heritage, without Central Asian accessions for direct comparisons. These studies are still useful, because they documented THC/CBD ratios

prior widespread hybridization. For the studies listed in Table S12, we limited reporting to drug-type plants (fiber-type plants were omitted). We also limited results to female flowers, whenever possible (some studies in the 1970s and 1980s analyzed both female and male flowering tops, as well as leaves, and even shoots). Few studies reported THC/CBD ratios, we calculated these from reported THC%, CBN%, and CBD%.

In Table S12, study numbers *1-18 (in italicized green)* were applied to Fig. 2 in the main document. Not all the studies in Table S11 are numbered, because some did not include accessions of verifiably South Asian heritage (*e.g.,* Davis *et al.* 1963, Ohlsson *et al.* 1971). We include them for historical interest. Studies that reported "no CBD detected" were omitted, because a ratio could not be calculated. Several early studies utilized GC with packed columns, so their THC/CBD ratios were artificially low, due to the inclusion of CBC in their CBD peaks. Variability in THC/CBD ratios in some of the early studies is also puzzling (*e.g.,* Doorenbos *et al.* 1971).

Table S12. THC/CBD ratios reported in studies from the late 20th century

| Citation, VDL or CGE (location if latter), instrumentation, calculated ratio, | Results |
|---|---|
| Davis *et al.* (1963), VDL, GC-FID, THC+CBN/CBD | Greece from 1924 (1.42), Morocco from 1948 (1.15), Brazil from 1936 (0.86) |
| Ohlsson *et al.* (1971), CGE in Sweden, GC-FID, THC/CBD | Morocco (10.0); Jezzine, Lebanon (1.3); Beqaa, Lebanon (0.2); Turkey (0.025); and feral plants from Beirut (0.020). |
| *1. Fetterman et al. (1971),* both CGE and VDL, in Mississippi, GC-FID, THC+CBN/CBD | CGE: Mexico fresh (12.3), Mexico stored for a year (20.5), Mexico bracts-only (25.8); VDL: Thailand 1 (13.8), Thailand 2 (11.8); $\bar{x}$ = 16.84. |
| *2. Doorenbos et al. (1971),* both CGE and VDL, in Mississippi, GC-FID, THC+CBN/CBD | CGE: India 1 (1.5), India 2 (10.0), Thailand 1 (8.1), Thailand 2 (6.1), Thailand 3 (7.8), Mexico (10.6), CME vs. VDL: Thailand in Missis (20.7), Thailand in Thailand (35.1). Mexican CME three successive seasons (12.3, 21.4, 21.8); $\bar{x}$ = 14.13 |
| *3. Small and Beckstead (1973),* CGE in Canada, GC-FID, THC/CBD; omitted accessions from hort gardens or ag stations of questionable provenance (*e.g.,* three accessions of *C. indica* with no THC); data from females unless none available | Cambodia 6.06, Gambia 13.86, India194 10.53, Jamaica 4.0, Malawi300 28.8, Malawi301 17.45, Malawi303 12.86, Mauritius 37.25, Mexico284 6.61, Mexico289 6.43, Mexico41 5.90, Mexico281 5.00, Rhodesia 12.17, Sierra Leone 12.22, South Africa11 9.72, South Africa74 14.89, South Africa162 13.20, South Africa273 24.00, Thailand10 5.5, Uganda76 7.53 Uganda77 14.00<br>$\bar{x}$ = 12.76 ±1.83 SEM (n =21) |
| *4. Chiesa et al. (1973)* VDL of domestic Argentina seizures, GC-FID, THC+CBN/CBD | Accessions "cultivated for illicit purposes," 180, 60, 50, 45 ($\bar{x}$ = 83.75); excluded: accessions "cultivated for hemp," and accessions of unknown provenance |
| *5. Jenkins and Patterson (1973)* VLD of seized material, GC-FID, THC+CBN/CBD | South Africa herb (n =6, $\bar{x}$ =10.8), Nigeria herb (n =5, $\bar{x}$ =9.7), Jamaica herb (n =7, $\bar{x}$ =10.0), Burma herb from 1950s (n=5, $\bar{x}$ =5.1); $\bar{x}$ = 9.92. Lebanon resin (n =7, $\bar{x}$ =0.60), Morocco resin or herb (n =10, $\bar{x}$ =1.92), Pakistan resin (n =19, $\bar{x}$ =1.07) |

| | |
|---|---|
| Turner and Hadley (1973) CGE in Mississippi, GC-FID | South Africa, n =2 accessions, THC $\bar{x}$ = 1.39% (mixed male and female tops), no CBD detected |
| Fairbain and Liebmann (1974) CGE in London, GC-FID, THC+CBN/CBD | South Africa, Thailand, India, Nepal, Mexico, flowering tops with THC% (range 2.4 to 7.1), CBD detected but but under their limit of quantification |
| *6. Der Marderosian and Murthy (1974)* VLD, GC-FID, THC+CBN/CBD | Mexico (12.5), India from 1965 (6.4), "Indian hemp" from 1880 (5.8) |
| *7. Holley et al. (1975)* CGE in Mississippi, but "some cross-pollination has occurred," GC-FID with silyation to remove CBC from THC/CBD | Female plants: India A (59.3), India A1 (110.5), India A2 (8.6), India B2 (136.0), India B3 (195.0), India E (165.5), Brazil (261.0), Sierra Leone A (61.5), South Africa A1 (101.0), South Africa A2 (3.6), South Africa D (184.0), South Africa E (10.5), South Africa F (33.0), Thailand B (145.5), Thailand C (0.57), Thailand D (1.2); $\bar{x}$ = 92.30. |
| *8. Mobarak et al. (1974)* both CGE and VDL, in Sweden, GC-FID, THC+CBN/CBD | CGE: South Africa (22.3), Thailand 1 (10.5), Thailand 2 (10.9). VLD: Indonesia (at different stages of maturity) n=7, $\bar{x}$ = 7.5 |
| *9. Bazzaz et al. (1975)* CGE, Illinois growroom, GC-FID, THC+CBN/CBD | Under optimal temperatures: Nepal (16.5), Panama (10.2), Jamaica (9.5), Illinois feral (1.0) |
| *10. Marshman et al. (1976)* VLD, GC-FID, THC+CBN/CBD | 36 samples of Jamaican herb (not including 3 without measurable CBD) $\bar{x}$ =31.1 ±4.52 SEM (range 11.1 to 104.4, both mature and immature plants) |
| *11. Hemphill et al. (1980)* CGE, Indiana glasshouse, GC-FID, THC+CBN/CBD | Data from excised bracts: Mexico 1 (153.3), Mexico 2 (no CBD detected), Japan "drug" from Mexico (128.0) |
| *12. Veszki et al. (1980)* CGE in Hungary, GC-FID, THC+CBN/CBD | female inflorescences 1978: Mexico (25.45), Thailand (12.23); $\bar{x}$ = 18.8 |
| *13. Field and Arndt (1980)* VDL of South African plants, GC-MS, THC+CBN/CBD+CBD+CBN3 | female inflorescences or female "growing tips": Transkei (46.3, 23.7), Pongola (12.6), Tzaneen (114.5, 17.2, 14.3, 12.1); $\bar{x}$ = 34.4 |
| Baker *et al.* (1980b) VLD of seized material from around the world coming into the United Kingdom | Baker and colleagues analyzed 304 seizures for THC content, a fascinating read, as are his follow-ups, Baker *et al.* (1982) and Pitts *et al.* (1990)—but they omitted CBD. |
| Fournier (1981) CGE in France, GC-FID, THC/CBD | Mexico (THC 1.52%, no detectable CBD), Lebanon (0.58) |
| Turner *et al.* (1982) CGE in Mississippi, GC-FID with silyation, THC+CBN/CBD | Mexican (n =9), THC% range 0.14 to 2.66, no measurable CBD content except for one accession |
| Taylor *et al.* (1983) GC-FID of seized germplasm grown outdoors | no detectable CBD: India (2.8/0), Morocco (1.4/0), Zimbabwe (1.7/0), India Zambia (0.66/0), fiber-type from Sri Lanka |
| Pitts *et al.* (1992) GC-FID, 6th generation plants from Taylor *et al.* | no detectable CBD: Sri Lanka (3.49/0), Zambia (1.27/0), Morocco (1.0/0), Morocco (1.4/2.0), fiber-type from Sri Lanka |
| *14. Brenneisen and ElSohly (1988)* GC-MS, CGE in Mississippi, $\Delta^9$THC+$\Delta^8$THC+CBN/CBD+CBE | ratio of peak areas: Mexico (11.1), Columbia (83.0), Jamaica (7.0), Thailand (24.6); $\bar{x}$ = 31.4 |

| | |
|---|---|
| *15. de Meijer et al. (1992)* GC-FID, CGE in Holland, THC/CBD | Swaziland (4.2), South Africa (14.1); excluded: hybrids ("Nederwiet", 7.4, "Skunk #1, 5.2), Spanish birdseed (4.6), Lebanon landrace (2.3) France unknown provenance (2.7) |
| *16. Hillig and Mahlberg (2004)* CGE in Indiana, GC-FID with capillary columns, THC/CBD | mean THC (5.48%) and CBD (0.02%) in NLD accessions (n =68), from which we derive a mean THC/CBD ratio of 274.0. |
| *17. Kallawicha (2008)* survey of drug-type and fiber-type plants in Thailand, GC-MS, THC/CBD | drug-type plants in Thailand (n =55) $\bar{x}$ =44.51 |
| *18. de Meijer et al. (2009)* GC-FID, CME in Holland, THC/CBD | percentage of cannabinoid content: "Haze" hybrid, but all South Asian heritage: Colombia, Mexico, Thailand, and southern India (191.2) |
| Onofri *et al.* (2015) GC-FID, CME in Holland, THC/CBD | no detectable CBD: South Africa, Malawi, Thailand |
| de Meijer and Hammond (2016) GS-FID, CME in Holland, THC/CBD | no detectable CBD: South Africa, Malawi, Thailand |
| Hanuš *et al.* (2018), GC-MS, seized hashish, THC+CBN/CBD | "tried" to evaluate confiscated samples by their geographical origin: Morocco (n =30, $\bar{x}$ =3.06 ±0.02), Lebanon (n =15, $\bar{x}$ =0.44 ±0.02), India (n =13, $\bar{x}$ =2.60 ±0.49). Large variability in Indian samples suggests diverse origins (as does the presence of *α*-bisabolol) |

***C. himalayensis***

The few cannabinoid studies of Himalayan plants present us with an enigma: History tells us that *charas* rubbed from wild-type plants was a high-quality commodity (Clarke 1998). Yet plants with very low THC appear in several modern THC/CBD studies. Over a century ago, Hooper (1908) considered Himalayan *charas* among the best available (Table S10).

Another enigma: botanists such as Strachey (1848) described "a jungle of wild hemp" in the Himalaya. But widespread cultivation also occurred in the Himalaya. Hooper's results from Kumaon include *charas* from both wild and cultivated plants (Table S10). In fact, a lot of *charas* came from cultivated plants (Table S4). Transitioning from wild-to-cultivated sources continued into the 20$^{th}$ century: In Kullu, Himachal Pradesh, *charas* was rubbed from wild-type plants (Indian Hemp Drugs Commission 1894), but by the 1960s it was rubbed from cultivated plants (Clarke 1998).

In modern THC/CBD studies, a few Himalayan accessions do not qualify as drug-type plants, having THC <0.3%, and their THC/CBD ratios suggest fiber-type plants. History also tell us that Himalayan plants were cultivated for fiber. Plants grown for fiber may have been wild-type *C. himalayensis* brought into cultivation, or foreign germplasm imported from elsewhere (text following Table S4).

Sampling bias is evident: de Meijer (1994a) analyzed two accessions from Kalopani in Nepal, described as "wild or naturalized" (de Meijer and van Soest 1992), with very low THC content, 0.06% and 0.44%. But around the same time, wild-type plants were encountered in Kalopani during a collecting trip in 1986 (McPartland and Hughes 1994); female infloresences

were collected, and a bioassay with four people revealed very potent psychoactivity (McPartl., pers. observ., 1986).

Small and Beckstead (1973) analyzed four "himalayana" accessions obtained from European botanical gardens, and one from Darjeeling. The veracity of botanical garden "himalayana" accessions has to be questioned; botanical gardens also supplied Small and Beckstead with three accessions of "*C. indica*" with no measurable THC. In contrast, the Darjeeling accession produced a THC/CBD+CBC ratio of 10.53, from male plants (females failed to flower before frost). The Darjeeling accession had wild-type achenes, see the photo in Small (1975).

Fairbain and Liebmann (1974) grew a Nepal accession obtained from the United Nations Division of Narcotics. They reported THC 0.76%, and a small CBD peak was eluted, but below their limit of quantification. Given their limit of quantification was 0.01%, the THC/CBD ratios calculated with this value equals 76. Rowan and Fairbairn (1977) clarified that the Nepal accession had wild-type traits: achenes small (2.5-3.0 mm), with a slightly elongated base, and a persistent perianth.

Turner (1974) grew a Nepal accession in Mississippi, and analyzed it with GC-FIDS, THC = 2.81%, CBD+CBC 0.21%, THC/CBD+CBC ratio 13.38. The following year they separated CBD from CBC, and reported "Nepal NE-C" with THC 2.75%, CBD 0.02%, CBC 0.28%, THC/CBD ratio 137 (Holley *et al.* 1975). Inspection of a voucher specimen of "NE-C second seed lot" [second generation?] revealed wild-type traits (McPartl., pers. observ., GH, 1997).

We omitted Bazzaz *et al.* (1975), they did not state whether their accession was domesticated or wild type, but its THC/CBD ratio was suggestive of the latter. They cultivated germplasm obtained from the mountains of Nepal at 6000 ft., outside of Kathmandu (at 4600 ft). Under optimal temperatures, they reported THC+CBN 14.51%, CBD+CBC 0.88%, a ratio of 16.48.

Turner *et al.* (1979) analyzed wild-type plants in India. As noted in the morphology section, they only reported one wild-type trait, achene size. Accessions consisted of two groups: from <2000 m altitude (Punjab plain), or from >2000 m (Himalaya). Each group was dichotomized into drug-type plants or fiber-type plants. Means for wild-type drug plants >2000 m (Himalaya) were $\Delta^9$THC+$\Delta^8$THC+CBN = 0.524%, CBD = 0.138%, ratio = 3.80. A sample from Manali, source of famous "Manali cream" *charas*, had a ratio of 6.4. Means for drug-type plants <2000 m (Punjab plain): $\Delta^9$THC+$\Delta^8$THC+CBN = 2.12%, CBD = 0.087%, ratio = 25.0.

When Himalayan plants were cultivated in Mississippi, THC/CBD ratios inexplicably increased, to $\bar{x}$ = 29.2 (due mostly to decreased CBD—one accession even dropped to "trace," which we equated to 0.005%). Mississippi-grown Punjab plants also decreased CBD content, their THC/CBD jumped to $\bar{x}$ = 107.4. No other study in the literature has reported this degree of THC/CBD variability.

De Meijer *et al.* (1992) analyzed a "wild" Nepal accession (#891103), THC 1.17%, CBD 0.63%, ratio of 1.88. De Meijer (1994) measured three Nepali accessions, characterized as wild (de Meijer and van Soest 1992). Accession 891191: THC 0.06%, CBD 0.82%, ratio 0.29; accession 891192: THC 0.44%, CBD 0.55%, ratio 0.98; accessions 891193: THC 1.17%, CBD 0.62%, ratio 1.95. Mean ratio from both studies, $\bar{x}$ = 1.28.

Hillig and Mahlberg (2004) analyzed "*C. indica* feral" accessions from Nepal and India (including several from de Meijer); $\bar{x}$ = THC 3.04%, CBD 1.95%, ratio 1.56 (Table S11). They report n =14 accessions, but passport data for their collection listed only five (Hillig 2005a). Interestingly, de Meijer's accession 891193, with the highest THC/CBD ratio, was not included in Hillig's analysis; he considered it a domesticated "East Asian fiber-type." In Hillig's allozyme study, 891193 and the other Himalayan "East Asian fiber-type" (92107) clustered together with his Himalayan wild-types (Hillig 2005a), so they are genetically related.

We omitted from consideration the study by Rigter and Niesink (2018), who analyzed 683 samples of *hashīsh* sold in Dutch coffeeshops. Nine identified as "Nepal," ultimately of unknown provenance (possibly Dutch), averaged THC 13.4%, CBD 0.6%, a ratio of 22.3.

### *C. asperrima*

Zhu *et al.* (1992) used GC-FID to analyze 27 wild-type accessions in Xīnjiāng. They measured THC+CBN/CBD in female flowering and fruiting tops (Table S13). As discussed in the morphology section, plants in Dzungaria likely represent naturalized fiber-type hemp. Consistent with this, the weighted mean of Dzungarian THC+CBN $\bar{x}$ = 0.36% (omitting one outlier, Ürümqi, which is Xīnjiāng's center of drug use). The weighted mean of Tarim/Turpan THC+CBN $\bar{x}$ = 1.52% (omitting one outlier, Hotan, Xīnjiāng's center of fiber-type cultivation); these plants likely represent *C. asperrima*.

Table S13. Cannabinoid data from Zhu *et al.* (1992), prefectures listed from north to south.

| Region | Prefecture, sample size | THC+CBN% mean | Weighted means | THC+CBN/CBD mean | Weighted means |
|---|---|---|---|---|---|
| Dzungaria | Altay, n =5 | 0.33% | 0.36% | 0.15 | 0.163 |
| | Tăchéng, n =1 | 0.43% | | 0.13 | |
| | Boltala, n =1 | 0.25% | | 0.08 | |
| | Yili, n =3 | 0.41% | | 0.22 | |
| | Changji, n =1 | 0.40% | | 0.18 | |
| | Ürümqi, n =1 | 0.82% | | 0.85 | |
| Tarim/Turpan | Hami, n =3 | 1.25% | 1.52% | 3.38 | 1.79 |
| | Turpan, n =2 | 1.10% | | 0.77 | |
| | Aksu, n =3 | 1.76% | | 0.88 | |
| | Kāšğar, n =6 | 1.67% | | 1.78 | |
| | Hotan, n =1 | 0.82% | | 0.85 | |

Two small studies analyzed plants that may represent *C. asperrima*. In the Kazakhstan part of the Chuy valley, Sarsenbaev *et al.* (2017) measured THC/CBD in four accessions of "*C. ruderalis*" (THC 1.0-1.5%), and reported ratios of 1.50, 1.52, 2.60, and 9.43. Small and Marcus (2003) analyzed a wild-type accession from Kazakhstan. They did not measure CBD, but its THC content was low, 0.41%.

### "Sativa" and "Indica"

Several studies measured cannabinoids in accessions putatively identified as "Sativa" or "Indica". Study numbers 1-9 were applied to Fig. 2 in the main document, labelled with

underlined numerals, *“Sativa” in italicized green*, and “Indica” in nonitalicized red. Some of these studies of “Indica” and “Sativa” show reversals from their landrace ancestors: whereas landraces from Central Asia expressed THC/CBD ratios lower than landraces from South Asia, six recent studies reported the reverse in “Indica” and “Sativa” (Fischedick et al. 2010; Hazekamp and Fischedick 2012; Elzinga et al. 2015; Hazekamp et al. 2016; Lynch et al. 2016; Jikomes and Zoorob 2018).

1. Fischedick *et al.* (2010) conducted a CGE in Holland with 11 chemovars bred by Bedrocan BV, analyzed with GC-FID. The collection consisted of “Indica” chemovars (considered nonhybridized, n =6), hybrid “Indica/Sativa” (n = 2), “mostly Sativa” (n = 2), and “Indica/Sativa/Ruderalis” (n = 1). “Indica” accessions were nearly devoid of CBD, either “trace” (interpreted as 0.02), or not detected; THC/CBD $\bar{x}$ = 127.0. This THC/CBD ratio in 21$^{st}$ century “Indicas” is inconsistent with Central Asian landraces from the 1970s-1990s. “Mostly Sativa” accessions had a THC/CBD $\bar{x}$ = 0.40, inconsistent with South Asian landraces from the 1970s-1990s.

2. Hazekamp and Fischedick (2012) collected coffeehouse and pharmacy samples (a VLD study) analyzed with GC-FID. They including two “Sativa dominant” samples (“Amnesia,” “Bedrobinal”) and two “Indica dominant” samples (“White Widow,” “Bedica”). Interpreting trace as 0.02, “Sativa dominant” THC/CBD $\bar{x}$ = 477.1; “Indica dominant” $\bar{x}$ = 550.2.

Piluzza *et al.* (2013) conducted a CGE in Italy, analyzed with HPLC-UV. They compared 19 accessions: one purported Afghani hybrid, two “Skunk #1” hybrids, four “Sativas” (two “Haze” hybrids, and two Thai hybrids), and an assortment of fiber-type plants and other accessions. They did not provide quantitative data, so we could not include them in Fig. 2 (in the main document), but multivariate analysis of cannabinoid content (NJ/UPGMA) clustered the “Indica” with two “Skunk” hybrids, separate from the four “Sativa” accessions.

3. Omar *et al.* (2013) measured THC+CBN/CBD using GC-MS in a CME in Spain, analyzing two “Sativa dominant” (“AK-47”, “1024”), $\bar{x}$ = 35.6; and two “Indica dominant” (“Critical”, “Somango”), $\bar{x}$ = 28.1.

4. Elzinga *et al.* (2015) used HPLC (UFLC) to analyze 35 strains obtained from “chemotypical medicinal cannabis dispensaries,” and assigned strains to “Indica,” “Sativa,” or “Hybrid” based on reports by the Leafly website. They did not report CBD content in individual accessions, but report a median value of 0.3%. For THCmax%, “Indica” (n = 13, $\bar{x}$= 17.30%) and “Sativa” (n =5, $\bar{x}$= 13.84%). Applying their median value computes THC/CBD means of “Indica” 57.6, “Sativa” 46.13.

5. Lynch *et al.* (2016) obtained VDL samples from “a variety of breeding and production facilities,” and from those sources they categorized samples as “BLDTs” (*a.k.a,* “Indica”, n =17) or “NLDTs” (*a.k.a.,* “Sativa”, n =35). They used HPLC to determine THCA% and CBDA%, presented as histograms, from which we calculated THC/CBD ratios. They showed a stunning reversal from their putative landrace ancestors: “BLDT” (*C. afghanica* herein) = 82.5, “NLDT” (*C. indica* herein) = 6.45.

6. Hazekamp *et al.* (2016) collected 460 samples (from coffeeshops, Bedrocan, and HempFlax, a VLD study), and quantified cannabinoids with GC-FID. They categorized the accessions as “Sativa” (n =68), “Indica” (n =63), “hybrid” (n =208), and “hemp” (n =121). THC/CBD means were “Sativa” 33.8, “Indica” 46.16. Multivariate clustering (OPLS-DA) produced a scatterplot that segregated “Sativa” and “Indica” into distinct clusters, with terpenoids providing the discrimination.

7. Sexton *et al.* (2018) conducted a CME of several hybrid strains, three of which have strongly Indica-dominant pedigrees, “Cherry Kush”, “Blackberry Kush”, and “Pineapple Kush”. They used HPLC to analyze THCA+THC+CBN/CBDA+CBD, mean ratio = 690.0.

8. Onofri *et al.* (2015) used GC-FID to measure THC and CBD as a percentage of total cannabinoid content in 18 accessions. Three are described as selections from Afghani landraces, but their anomalous results are consistent with inadvertent hybrids, THC/CBD ratio $\bar{x}$ =400.1.

9. Welling *et al.* (2016) used HPLC-DAD to measure THC and CBD as a percentage of total cannabinoid content, and presented data in histograms. They tested a purported Afghanistan landrace. It was likely an unstable hybrid. Two plants expressed THC/CBD ratios of 0.05 and 0.08, and the third was 95; $\bar{x}$ = 31.71.

Jikomes and Zoorob (2018) obtained data from the Washington State Liquor and Cannabis Board, for all *Cannabis* tested for THC and CBD content between 2014 and 2017. The data was linked to strain names, and they used Leafly, an on-line *Cannabis* databank, to categorize the strains as “Sativa” or “Indica”. THC/CBD ratios could not be calculated for “Sativa” and “Indica”, because Jikomes and Zoorob presented THC and CBD data separately, according to chemotype. They present THC data for chemotype I (THC/CBD ratio <5), which did not significantly vary between “Sativa” (n =26,389, $\bar{x}$= 19.4%) and “Indica” (n =35,276, $\bar{x}$= 19.0%). They present CBD data for combined chemotype I and III (THC/CBD ratio ≥5), which also did not significantly vary between “Sativa” (n =848, $\bar{x}$= 10.6%) and “Indica” (n =1014, $\bar{x}$= 9.3%).

**SF.9. Phytochemical comparisons, cont.**

**Carboxylic precursors THCA and CBDA**

As aforementioned, the carboxylic precursors of THC and CBD are tetrahydrocannabinolic acid (THCA) and cannabidiolic acid (CBDA). The carboxylic acids are unstable and readily decarboxylate into THC and CBD. This happens in storage at room temperature, and accelerates rapidly with heating (smoking, baking).

Hoffmann-La Roche & Co. (1915) first isolated THCA, but they didn’t appreciate their achievement. They “cold” extracted *hashīsh* (probably at room temperature) under vacuum with petroleum ether, and then added “aqueous alkali” (NaOH or $Na_2CO_3$). THCA was soluble in aqueous alkali, which they tossed away. The purpose of the process was to remove pharmacologically inactive substances, and we now know that THCA does not bind to $CB_1$ cannabinoid receptors (McPartland *et al.* 2017). Hoffmann-La Roche obtained a patent on the process—the first-ever cannabinoid patent.

Researchers have used the THCA/THC ratio to identify the geographic source of confiscated material (Baker *et al.* 1981, 1982a, Taylor *et al.* 1983, Pitts *et al.* 1992), when in fact that metric simply determined the age and/or storage conditions of samples. They were unaware that Grlić and Andrec (1961) already showed that old, dated *Cannabis* samples were largely bereft of carboxylic acids.

Two isomers of THCA have been isolated and characterized, 2-COOH-THC (THCA-A) and 4-COOH-THC (THCA-B) (Fig. S13). The existence of THCA-B suggests the presence of an unelucidated enzyme in the biosynthetic pathway: an enzyme that would catalyze the allylic rearrangement between THCA-A and THCA-B. The enzyme would need to act near the end of the biosynthetic pathway, because no “CBGA-B” or “CBDA-B” has been identified.

Figure S13. Chemical structures of THC (left), THCA-A (center) and THCA-B (right).

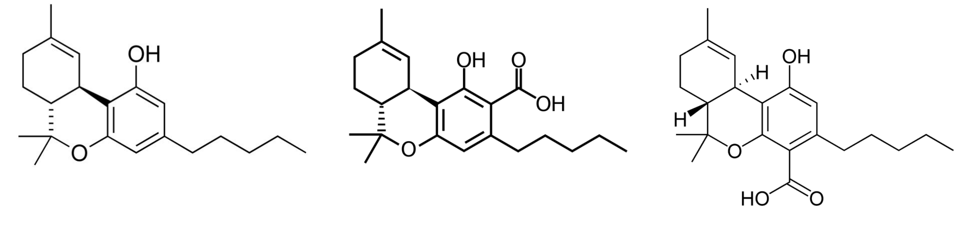


A few botanical studies reported the geographic provenance of accessions from which they obtained THCA-A or THCA-B. We tabulated these studies to see if any taxonomic signal by geographic distribution could be discerned (Table S14), and none could be discerned. Lehmann and Brenneisen (1992) isolated both THCA-A and THCA-B in all three *Cannabis* chemotypes—drug-type, intermediate-type, and fiber-type (of unstated provenance).

Table S14. Incidence of THCA-A and THCA-B in samples identified by geographic provenance

| isomers | THCA-A | THCA-B |
|---|---|---|
| geographic provenance of samples, citations | **Afghanistan** (Turner *et al.* 1974)<br>**Lebanon** (Edery *et al.* 1972)<br>**Morocco** (Holley *et al.* 1975)<br>**Jamaica** (Brenneisen & ElSohly 1988)<br>**Thailand** (Brenneisen & ElSohly 1988)<br>**Mexico** (Yamauchi *et al.* 1967,Turner *et al.* 1973b, Yotoriyama *et al.* 1980) | **Afghanistan** (Turner *et al.* 1974)<br>**Lebanon** (Mechoulam *et al.* 1969)<br>**Morocco** (Holley *et al.* 1975)<br>**Jamaica** (Brenneisen & ElSohly 1988)<br>**Turkey** (Turner *et al.* 1974)<br>**Mexico** (Yotoriyama *et al.* 1980)<br>**India** (Turner *et al.* 1974)<br>**Columbia** (Brenneisen & ElSohly 1988) |

**SF.9. Phytochemical comparisons, cont.**
**The $C_{19}$ cannabinoids THCV and CBDV**

Two $C_{19}$ cannabinoids may reflect phylogenetic relations: tetrahydrocannabivarin (THCV) and cannabidivarin (CBDV). The biosynthetic pathway leading to THCV and CBDV diverges early, along the resorcinol side of the cannabinoid biosynthetic pipeline. Instead of utilizing hexanoate ($C_5H_6COOH$), the $C_{19}$ pathway uses butanoate (butyric acid, $C_3H_7COOH$). Some

workers added $C_{19}$ cannabinoids to the cannabinoid profile, as THC+THCV/CBD+CBDV (*e.g.,* Turner *et al.* 1980). In contrast, Hillig and Mahlberg (2004) segregated THCV and CBDV from the cannabinoid profile, as a separate taxonomic character. They reported the $C_{19}$ cannabinoids as a sum, THCV+CBDV. Another metric, heretofore unreported, is the $C_{19}$ /$C_{21}$ ratio: THCV+CBDV/THC+CBD.

Vollner *et al.* (1969) first isolated CBDV, from *hashīsh* of unstated provenance. Gill *et al.* (1970) first isolated THCV, from *Cannabis indica* tincture made in Pakistan. Merkus (1971) extracted THCV from Nepali *hashīsh*, and also discovered the $C_{19}$ breakdown product of CBN, naming it cannabivarin, CBNV. De Zeeuw et al. (1972) stated that THCV and CBDV occur at higher concentrations in Asian plants than in *hashīsh* samples from Middle Eastern and Mediterranean countries. Mobarak *et al.* (1974) reported CBDV in Indonesian plants, but no CBDV in plants from South Africa or Thailand.

South African plants produce high levels of THCV, first reported by Paris *et al.* (1973). Steinberg *et al.* (1975) identified two South African chemotypes—plants grown from Pongola germplasm were THCV dominant, whereas plants from Transkei or Tzaneen germplasm were THC dominant. Boucher *et al.* (1977) also revealed two chemotypes, either THC dominant or THCV dominant. Field and Arndt (1980) recognized three chemotypes—Transkei plants had a THC/THCV ratio of 45.3, Tzaneen plants had a ratio of 5.9, and Pongola plants had a ratio of 1.0. Pongola and Tzaneen plants also produced some CBDV.

Turner *et al.* (1973b) used GC-MS in a CGE of many accessions. They report $C_{19}$ analogs as a percentage of total cannabinoid content, rather than percentage dry weight plants. Their symbol "+" indicates a range of 1-5%, "T" indicates trace (~1%), and "–" indicates little or none. We limit their THCV/CBDV results to mature females: In Central Asian landraces: Afghanistan A (5.34/8.33), Afghanistan B (7.72/+), Pakistan (+/+), Iran (+/–). In plants of South Asian heritage: India A (t/–), India B (10.63/+), India E (+/–), Nepal (+/t), Thailand B (t/–), Thailand C (t/t), Thailand D (t/t), Vietnam (t/–), South Africa (53.69/t), Mauritius (+/–), Brazil (t/–), Mexico (t/t), Ethiopia (t/t), Ghana (t/–), Sierra Leone (t/–), Sudan (+/+).

Mobarak *et al.* (1978) used GC-MS to analyze *hashīsh* from Nuristān, Afghanistan. They quantified individual cannabinoids as a percentage of total cannabinoid content, rather than percentage dry weight plants: THC+CBN = 22.4%, THCV = 13.56%, CBDV = 13.48%, CBD = 11.6%. The $C_{19}$ /$C_{21}$ ratio equals 1.26, which departs from anything reported by Hillig and Mahlberg (2004).

Turner *et al.* (1982) conducted a CGE in Mississippi, grew Mexican accessions, and used GC-FID with silyation. They reported THCV all nine accessions, $\bar{x}$ =0.054% dry weight. They reported CBDV in seven of nine accessions, $\bar{x}$ =0.01% dry weight.

Brenneisen and ElSohly (1988) conducted a CGE in Mississippi, and used GC-MS to detect a slew of $C_{21}$ and $C_{19}$ cannabinoids: $\Delta^9$-THC ($C_{21}$), $\Delta^8$-THC ($C_{21}$), CBN ($C_{21}$), cannabigerol ($C_{21}$), cannabifuran ($C_{21}$), dehydrocannabifuran ($C_{21}$), cannabielsoin ($C_{21}$), cannabicumaronone ($C_{21}$), cannabichromene ($C_{21}$), CBD ($C_{21}$), cannabicyclol ($C_{21}$), cannabivarin ($C_{19}$), THCV ($C_{19}$), and

cannabicitran ($C_{19}$). They quantified these compounds as peak areas, from which we calculated $C_{19}$ /$C_{21}$ ratios: Mexico (0.33), Columbia (0.05), Jamaica (0.22), Thailand (0.09).

Zhang and He (1992) used GC/MS to analyse nine samples seized in Xīnjiāng Region, mostly from Hotan and Kashi prefectures. They quantified eight cannabinoids, reported as percentages of the total cannabinoid content. The mean $C_{19}$ /$C_{21}$ ratio = 0.03 ±0.005 SEM, range 0.02 to 0.06.

Hillig and Mahlberg (2004) report levels of THCV+CBDV (percentage dry weight) highest in feral *C. indica,* followed by domesticated *C. indica* ("NLD"), and lowest in *C. afghanica* ("WLD"). See Table S11. De Meijer and Hammond (2016) quantified individual cannabinoids as a percentage of total cannabinoid content, rather than percentage dry weight plants. They analyzed several landraces and hybrids, from which we calculated $C_{19}$ /$C_{21}$ ratios: Malawi landrace (0.85), South Africa landrace (0.31), Thailand landrace (0.0), "California Orange" hybrid (0.78), Hawaiian hybrid (0.16).

## SF.9. Phytochemical comparisons, cont.
### Terpenoid profiles

**Terpenoids** include hydrocarbon terpenes and their oxygenated derivatives, which form alcohols, ethers, aldehydes, ketones, and esters. Terpenoids are polymers of isoprene ($C_5H_8$), so they are also called isoprenoids. *Cannabis* produces about 150 terpenoids, mostly monoterpenoids (based on a $C_{10}H_{16}$ template) and sesquiterpenoids (based on a $C_{15}$ $H_{24}$ template). Terpenoids account for up to 10% of trichome gland head contents (Malingré *et al.* 1975, Potter 2009). They are volatile, and impart the characteristic odor of *Cannabis*.

Collectively, terpenoids constitute the plant's essential oil or volatile oil. No terpenoids are unique to *Cannabis*, but various biotypes of *Cannabis* produce unique terpenoid profiles.

Terpenoid profiles vary by extraction methodology. Traditionally, terpenoids were extracted by either steam distillation or hydrodistillation. Steam distillation passes steam through a bed of plant material in a closed system. Volatile compounds are carried away in the steam, condensed and separated. Hydrodistillation is an older version of steam distillation, where plant material is soaked in water, then boiled, and volatile compounds are carried away in the water-oil vapor, condensed and separated.

Benelli *et al.* (2018) described hydrodistillation as "more aggressive" than stem distillation, producing oxidative and hydrolytic reactions. They steam distilled 'Felina 32', and their terpenoid profile consisted of 1.6% oxygenated terpenoids. When Bertoli *et al.* (2010) hydrodistilled the same cultivar, their terpenoid profile consisted of 5.67% and 7.46% oxygenated terpenoids (grown in two sequential seasons). Naz *et al.* (2017) showed differences in terpenoid profiles when the same sample was extracted by steam distillation, hydrodistillation, or supercritical $CO_2$ extraction.

The least aggressive method is "headspace" sampling: collecting the vapor emitted by a sample, and injecting it directly into a gas chromatograph to separate and identify its constituents. Hood *et al.* (1973) pioneered this method. Compared to a steam distillate, the headspace sample contained a higher percentage of extremely volatile monoterpenoids (*e.g., α-*

pinene, 3.9% vs. 55.5% of total terpenoids, respectively). Less volatile sesquiterpenoids showed a higher percentage in the steam distillate compared to the headspace (*e.g., β*-caryophyllene, 37.5% vs. 3.4%, respectively). Oxidized products, such as caryophyllene oxide, were present in the steam distillate (7.4%), but not in the headspace.

Most studies extracted terpenoids with solvents, before injecting them into a gas chromatograph. Hillig (2004b) extracted terpenoids in chloroform, as did Mansouri *et al.* (2011). Other solvents included ethanol (Fischedick *et al.* 2010, Hazekamp and Fischedick 2012, Hazekamp *et al*. 2016, Lewis *et al.* 2018), chloroform (Mansouri *et al.* 2011), ethanol and chloroform (Aizpurua-Olazizolo *et al.* 2016), methanol (Elzinga *et al.* 2015, Lynch *et al.* 2016, Fischedick 2017), hexane (Broséus *et al*. 2010), and pentane (Casano *et al.* 2011).

Brunschwig (1500) first steam distilled *hanff krut wasser,* "hemp vegetable water," from *dolden* ("umbels" or tops). In the 1800s a dozen studies were published on *Cannabis* essential oil (reviewed in Grassi and McPartland 2017). They culminated with an analysis of Indian *gañjā* by Prain (1893). Although he isolated the "narcotic fraction" of *gañjā* in a petroleum ether extract, a series of experiments led him to deduce that "to some extent the *exciting and exhilarating* effect of gánjá resides in an essential oil."

Some researchers attempted taxonomic comparisons based on essential oil. Personne (1857) found no compositional difference between *Cannabis sativa* and *Cannabis indica.* Both essential oils consisted primarily of two fractions, which he named, with erroneous formulae: *cannabène* ($C_{36}H_{20}$) and *cannabène hydrate* ($C_{12}H_{14}$). Valente (1880, 1881) also found no difference between *Cannabis sativa* (Italian hemp from Venice) and *Cannabis indica* ("*canapa gigantea* from the Indies"). Both essential oils consisted primarily of a hydrocarbon, $C_{15}H_{24}$, which he correctly identified as a sesquiterpenoid.

Hooper (1908) noted that the perceived quality and cost of three *charas* specimens correlated with their essential oil content, and *not* with their resin content: Grade No. 1: essential oil 12.7% and resin 40.2%; Grade No. 2: essential oil 12.4% and resin 40.9%; Grade No. 3: essential oil 12.0% and resin 48.1%.

Kudryashev (1932) conducted the first terpenoid analysis of plants that likely represent *C. asperrima*. He distilled essential oils from wild plants growing along the banks of streams and canals near Pokrovsky (now Kyrgyzstan). The essential oil consisted primarily of an unidentified sesquiterpene, with a boiling point of 256-258°F. Kudryashev noted the essential oil's "sharp unpleasant smell." We correlate this with Clarke (1981) describing Central Asian landraces with an acrid or skunky aroma.

Simonsen and Todd (1942) separated the essential oil of Egyptian *hashīsh* using fractional steam distillation. They identified *p*-cymene and humulene, and noted the absence of *β*-myrcene. Its absence surprised Simonsen and Todd, because they believe *β*-myrcene to be the $C_{10}H_{16}$ "terpene of low boiling point" that Wood *et al.* (1896) isolated from *charas* (obtained from Etawah, Uttar Pradesh). Martin *et al.* (1961) first used GC-FIDS to separate terpenoids, and found differences between wild hemp in Canada and samples of *hashīsh* and *charas*. They did not identify specific terpenoids other than by peak number.

Hood and Barry (1978) made the first comparison that included Central Asian accessions. They used GC-FID to quantify 17 terpenoids in 14 accessions grown in a CGE. The accessions included Central Asian plants from Afghanistan and Pakistan (n =3) and plants of South Asian heritage from India and Mexico (n = 5). Running statistics on their raw data revealed some terpenoids with statistical differences: more limonene in Af/Pak plants (mean 16.5% ±1.66 SD) than Indi/Mex plants (6.5% ±1.01, $p$ <0.001), and more *β*-farnesene in Indi/Mex (0.44% ±0.13) than Af/Pak (0.10% ±0.05, $p$ = 0.10).

Differences in three other terpenoids fell short of statistical significance in this small data sat: more *β*-caryophyllene in Indi/Mex (3.0% ±0.39) than Af/Pak (1.9% ±0.52, $p$ = 0.16), more *α*-humulene in Indi/Mex (0.76% ±0.20) than Af/Pak (0.53% ±0.15, $p$ = 0.20), and more *β*-myrcene in Af/Pak (10.0% ±0.53) than Indi/Mex (7.6% ±1.3, $p$ = 0.21).

Zhang and He (1992) used GC/MS to analyse nine samples seized in Xīnjiāng Region, mostly from Hotan and Kashi prefectures. The samples were old, and they only reported three monoterpenes: *α*-fenchene, *β*-pirene (probably *β*-pinene), and *γ*-terpinene (note: no limonene or *β*-myrcene). Among six sesquiterpenoids, they reported *β*-caryophyllene, humulene, and three unusual ones: *α*-santalene, *β*-santalene, *α*-selinen (probably *α*-selinene), and selinene (probably *β*-selinene).

Hillig (2004b) quantified terpenoids in a CGE of 82 accessions. He used GC-FID to separate 48 terpenoid peaks, and used GC-MS to positively identify 21 terpenoids. The accessions included "WLD" (n = 26), "NLD" (n =35), and "*C. indica* feral" (n =14 is stated, but only 4 appear in his scatterplot). Multivariate clustering clearly discriminated WLD and NLD accessions (canonical analysis as well as the PCA scatterplot), whereas "*C. indica* feral" accessions overlapped with the other groups in canonical analysis.

Four terpenoids with the greatest discriminatory value (*i.e.,* greatest PCA weight or eigenvector value) were **sesquiterpene alcohols**: guaiol, *γ*-eudesmol, *β*-eudesmol, and a peak tentatively identified as *α*-eudesmol. All terpenoids with significant differences ($p$ < 0.05) are presented in Table S15. Like Hood and Barry (1978), Hillig found greater *β*-myrcene content in WLD (9.0% ±6.5) than NLD (5.8% ±5.9), but the difference fell short of statistical significance because of wide variation.

Table S15. Terpenoids with statistically significant differences between "NDL" (*C. indica* herein) and "WLD" (*C. afganica* herein) reported by (Hillig 2004b), compared with "*C. indica* feral" (*C. himalayensis* herein) and East Asian hemp. Means not connected by the same letter are significantly different, Student's t test $P$ ≤0.05.

| | NDL | WLD | *C. indica* feral | East Asian hemp |
|---|---|---|---|---|
| limonene | 1.3%a | 4.0%b | 3.0%ab | 1.8%a |
| *γ*-terpinene | 0.2%a | 0.1%b | 0.1%b | 0.2%b |
| *β*-fenchol | 0.2%a | 0.8%b | 0.2%a | 0.3%a |
| terpinolene | 4.4%a | 1.0%b | 3.7%a | 1.1%a |
| *β*-caryophyllene | 15.7%a | 9.7%b | 21.9%c | 18.7%c |
| *α*-guaiene | 1.0%a | 0.4%b | 0.5%ab | 0.5% ab |
| trans *β*-farnesene | 7.6%a | 4.1%b | 4.3%b | 2.0%c |
| caryophyllene oxide | 8.9%a | 4.2%b | 2.5%b | 7.0%a |
| guaiol | 0.2%a | 3.5%b | 0.2%a | 0.5%a |

| | | | | |
|---|---|---|---|---|
| γ-eudesmol | 0.6%a | 4.8%b | 0.6%a | 1.5%c |
| β-eudesmol | 0.8%a | 7.4%b | 0.5%a | 1.0%a |
| α-eudesmol (peak 41) | 0.1%a | 1.4%b | 0.1%a | 0.3%a |

1. percentages are ratios of individual peak areas relative to the total area of all 48 terpenoid peaks

Broséus *et al*. (2010) compared 13 drug-type hybrids and five fiber-type cultivars. Their PCA analysis identified four terpenoids with "high discrimination capabilities" between the two groups: guaiol, *γ*-eudesmol, bulnesol, and *α*-bisabolol. Mansouri *et al.* (2011) analyzed terpenoids in an Iranian landrace, which expressed significant amounts of *β*-eudesmol, *γ*-eudesmol, and *α*-bisabolol, like plants of Afghani heritage. Iranian landraces also shared with Afghani plants a low THC/CBD ratio of 3.2 (Mansouri and Asrar 2012).

**"Sativa" and "Indica" terpenoids**

Fischedick *et al.* (2010) conducted a CME in Holland with 11 chemovars bred by Bedrocan BV, and analyzed 23 terpenoids using GC-FID. The collection included six chemovars considered nonhybridized "Indicas": "AD," "AF," "AM," "AN," "AO," and "Bedropuur." As mentioned in the cannabinoid section, none of the six contained more than a trace CBD—a marked divergence from their ostensible Central Asian ancestry—so they were unrecognized hybrids. Fischedick and colleagues made an interesting discovery: three of these unrecognized hybrids ("Bedropuur," "AO," and "AF") expressed guaiol, *γ*-eudesmol, and *β*-eudesmol, consistent with Central Asian ancestry.

Hazekamp and Fischedick (2011) collected coffeehouse and pharmacy samples (a VLD study) analyzed with GC-FID. They including two "Sativa dominant" samples ("Amnesia," "Bedrobinal") and two "Indica dominant" samples ("White Widow," "Bedica"). Only the "Indica dominant" hybrids contained guaiol, *γ*-eudesmol, and *β*-eudesmol.

Casano *et al.* (2011) compared 16 unnamed hybrid accessions, characterized as "mostly Indica" or "mostly Sativa." "Mostly Indica" plants produced significantly higher levels of limonene, *β*-myrcene, and camphene. "Mostly Sativa" produced significantly higher levels of sabinene, delta-3-carene, phellandrene, 1,8-cineole, cis-*β*-ocimene, trans-*β*-ocimene, and terpinolene.

Elzinga *et al.* (2015) assigned strains to "Sativa" or "Indica" according to the Leafly database, as described above. They noted that strains named *Kush*, "characteristic of the wide leaflet drug type strains originating from Hindus Kush region of Afghanistan and Pakistan," contained higher levels of guaiol, *β*-eudesmol, *β*-myrcene, trans-ocimene, and *α*-pinene.

Lynch *et al.* (2016) used a genetic analysis to differentiate strains into "BLDTs" (*a.k.a,* "Indica") and "NLDTs" (*a.k.a.,* "Sativa") in a VDL study. A subset of strains (BLDTs, n =17; NLDTs, n =35) were analyzed with HPLC-UV, to compare eight terpenoids. NLDTs produced significantly greater levels of *β*-myrcene and α-terpinolene (0.48% and 0.16%, respectively) than did BLDTs (0.35% and 0.09%). BLDTs produced greater levels of linalool (0.08%) than did NLDTs (0.02%). No statistically significant differences were seen in limonene, α-pinene, *β*-caryophyllene, and caryophyllene oxide. No sesquiterpene alcohols were measured.

Aizpurua-Olazizolo et al. (2016) cultivated seven unnamed accessions in a CME they assigned to chemotypes I, II, and II based on THC/CBD ratios, and then measured their terpenoid contents. They report higher levels of guaiol, *γ*-eudesmol, *β*-eudesmol, *α*-bisabolol, and eucalyptol in chemotype III plants.

Hazekamp *et al*. (2016) collected 460 samples (from coffeeshops, Bedrocan, and HempFlax, a VLD study), and quantified 17 monoterpenoids and 19 sesquiterpenoids with GC-FID. They categorized the accessions as "Sativa" (n =68), "Indica" (n =63), "hybrid" (n =208), and "hemp" (n =121). Multivariate clustering (OPLS-DA) produced a scatterplot that segregated "Sativa" and "Indica" into distinct clusters. "Indica" compared to "Sativa" produced more sesquiterpenoid alcohols (guaiol, *γ*-eudesmol, *β*-eudesmol, and *α*-bisabolol), as well as more monoterpenoid alcohols (*α*-terpineol, *β*-fenchol, linalool, cis-sabinene hydrate, borneol).

Sesquiterpenoid alcohols were reported in a study of European fiber-type plants: Bertoli et *al.* (2010) analyzed several Italian dioecious cultivars 'Carmagnola', 'Fibranova', 'C.S.', 'Red Petiole', and five unnamed accessions, using GC-MS. They contained trace levels of *γ*-eudesmol, *β*-eudesmol, and *α*-bisabolol. Italian dioecious cultivars are known for their relatively high THC/CBD ratios (for fiber-type plants). They likely harbor Asian genetics, via Turkey, dating back to the $14^{th}$-$15^{th}$ centuries, which may account for their sesquiterpenoid alcohols.

Fischedick (2017) divided medicinal cannabis strains into five terpenoid chemotype groups: 1. terpinolene dominant, 2. *β*-caryophyllene with alcohol-substituted terpenoids, 3. limonene/*β*-myrcene with alcohol-substituted terpenoids, 4. limonene/*β*-myrcene/*β*-caryophyllene with *α*-bisabolol, 5. *β*-myrcene dominant. Lewis *et al.* (2018) recognized eight "terpene super classes": 1. *β*-myrcene, 2. terpinolene, 3. ocimene, 4. limonene, 5. *α*-pinene, 6. humulene, 7. linalool, 8. *β*-caryophyllene. Orser *et al.* (2018) whittled the groups down to three: 1. *β*-myrcene, 2. terpinolene/*γ*-terpinene, 3. limonene/*β*-caryophyllene.

## SF.10. Molecular genetic comparisons

In the $20^{th}$ century, when unhybridized landraces were much more readily available, molecular methods were blunt instruments: Small (1972) karyotyped 38 accessions of *Cannabis*, analyzing chromosome number and appearance. He found no differences in any accessions.

Lawi-Berger *et al.* (1982) extracted proteins from achenes in five fiber strains and five drug strains. They found variations in banding patterns, but the differences did not segregate by plant use (fiber- or drug-type), or by geographical origin. De Meijer and Keizer (1996) compared achene proteins in 147 *Cannabis* accessions, grouped into fiber strains, drug strains, and wild populations. Variability in electrophoretic bands showed no patterns corresponding to plant use or geographical location.

Hillig (2005a) analyzed allozyme variation in the *Cannabis* collection he tested for cannabinoids and terpenoids. Allozymes are a subset of proteins—enzymes—that perform very basic functions, such as DNA polymerase. Homologous allozymes are coded by different alleles of the same gene. They may differ by only a single amino acid, but if the single substitution alters the electrical charge of the protein, the difference can be identified by electrophoresis.

Samples were evaluated for variation at 17 gene loci, and frequencies of 52 alleles were subjected to PCA. The PCA scatterplot segregated drug-type and fiber-type plants into distinct clusters, but the PCA ellipses for WLD (Afghani) and NLD (South Asian heritage) substantially overlapped.

Modern gene sequencing utilizes specifically-designed DNA primers, which target the amplification of a specific gene. Gene sequences are directly comparable between organisms, and they can be historically ordered (polarized) when anchored by an outgroup—enabling the construction of true phylogenetic trees. A haplotype is a set of DNA polymorphisms that tend to be inherited together from a single parent. A haplogroup is a group of similar haplotypes that share a common ancestor on the matriline (maternal line) or patriline (Y-chromosome). The matriline can be obtained from mitochondria (mtDNA), as well as chloroplasts (cpDNA), because no cpDNA is detected in *Cannabis* pollen cells (Zhang *et al.* 2003).

Gilmore *et al.* (2007) conducted a haplotype study using seven gene loci—six cpDNA sequences and one mtDNA sequence—whose polymorphisms consisted of SNPs, single base indels, or variable length repeated motifs. They examined 76 *Cannabis* accessions, including Central Asian germplasm. Unfortunately, the study's flaws are manifold. Their alignments did not consistently handle indels within homonucleotide runs and tandem repeats. The sequences they chose had essentially no useful variation. They did not trim primers from sequences. Their "*trnH-trnK*" was actually *psbA-trnH.* Sequences they deposited at GenBank revealed errors in their coding of 7-digit haplotypes. Some accession numbers and GenBank numbers were wrong. The two tree diagrams they illustrated did not agree. Sixteen accessions were police seizures of unknown provenance.

Gilmore obtained germplasm from de Meijer, as did Hillig, but Gilmore utilized accessions that Hillig rejected as hybrids. Nearly all of Gilmore's Central Asian genetics came from hybrids, such as "Skunk", "Skunk No. 1", "Four way", and "Breeder's seed". Given these shortcomings, their results are noteworthy. Parsimony analysis segregated the 76 accessions into three clades. Clade A comprised a majority of fiber-type plants. Clade B included Afghani plants along with most drug strains—hybrids and unidentified police seizures. Clade C was the most interesting—accessions of South Asian heritage—12 landraces from India, Nepal, Thailand, Jamaica, Mexico, and Africa. Gilmore (2005) gave the name *C. sativa rasta* to plants in Clade C, of South Asian heritage.

Zhang *et al.* (2018b) utilized five cpDNA sequences, *rps16, psaI-accD, rps11-rps8, rpl32-trnL, and ndhF-rpl32*, chosen after searching for highly variable regions in four completely-sequenced chloroplast genomes (Oh *et al.* 2016, Vergara *et al.* 2016a). Combined alignment of the five cpDNA sequences covered 3635 bp, harboring 19 SNPs and four indels.

They sequenced 645 plants from 52 accessions: three European fiber-types, a Korean fiber-type, 16 fiber-types from across China, 25 wild-type accessions from across China, two ostensible drug-type landraces (from Dagestan and Nigeria), and three drug-type hybrids reportedly ≥70% "Indica" ("Purple Kush", "Afghanica", "Dame Blanche"). Their study would have benefited by including non-hybridized South Asian landraces (the pedigree of the Dagestani

and Nigerian plants is unknown). They did include two “landrace” accessions from Xīnjiāng Region, which may represent drug-type Central Asian landraces (their map shows these were obtained near Hotan and Ürümqi—two hotspots of cannabis drug use in China).

The plants segregated into 25 haplotypes. Many of the 52 accessions contained individuals with different haplotypes. Polymorphic accessions usually consisted of individuals with two haplotypes. For example, individuals from two Xīnjiāng wild-type accessions harbored either H9 or H1 haplotypes. One fiber-type landrace from Gānsù included individuals spanning five different haplotypes. Only 25 accessions consisted of individuals with a single haplotype. For example, accessions with the single haplotype H9 included the three drug-type hybrids, both Xīnjiāng “landrace” accessions, and one of the five Xīnjiāng wild-type accessions.

Zhang and colleagues conducted a spatial analysis of molecular variance (SAMOVA) to determine the optimal number of haplogroups (K = n) to divide the haplotypes, based on cpDNA variation and geographical coordinates. The data best fit K = 3 haplogroups, corresponding to haplogroup H (High, mostly north of 40º N), haplogroup M (Middle, between 43º and 27º N), and haplogroup L (Low, mostly south of 30º N). Fourteen of the polymorphic accessions contained individuals that segregated into more than one haplogroup. Individuals in the aforementioned Gānsù landrace fell into all three haplogroups.

They used Bayesian inference (MrBayes) to determine phylogenetic relationships of the haplotypes, with *Humulus* and *Aphananthe* as outgroups. Branch lengths leading to three clades (the three haplogroups) were very short, and the phylogram did not include clade credibility values (posterior probability values). The phylogenetic tree was difficult to interpret. For example, haplotype H9 (with the three drug-type hybrids, both Xīnjiāng landraces, and polymorphic accessions from Xīnjiāng, Tibet, Qīnghǎi, Gānsù, Shānxī, Inner Mongolia, and Yúnnán) was sister to H12 (‘Carmagnola’, a European fiber-type cultivar). The Dagestani and Nigerian accessions formed a clade, sister to a clade with H1, which included ‘Kompolti’, Korea, and polymorphic accessions from Xīnjiāng, Inner Mongolia, and ‘Futura75’.

To identify climatic factors affecting distribution of the haplogroups, they tested correlations with bioclimatic factors. Haplogroup distribution correlated best with mean day length, followed by mean temperature and mean precipitation. These results are somewhat circular, because SAMOVA divided the haplogroups by latitude, and mean day length is a function of latitude. Zhang and colleagues also grew 43 accessions, to measure phenotypic traits (plant height, stem diameter, number of days to achene maturity). Phenotypic traits also correlated with latitudinal gradients—plants from Haplogroup H were the shortest ($\bar{x}$ =99.2 cm), with the thinnest stems ($\bar{x}$ =0.54 cm), and the shortest time to achene maturity ($\bar{x}$ =77.2 days). Plants from Haplogroup L were the tallest ($\bar{x}$ =238.0 cm), with the widest stems ($\bar{x}$ =1.14 cm), and the longest time to achene maturity ($\bar{x}$ =133.6 days).

Three nuclear DNA (nDNA) sequences have been utilized by *Cannabis* researchers: internal transcribed spacer 1 and 2 (ITS1 and ITS2), and the genes encoding THCA synthase and CBDA synthase. The proteins are abbreviated THCA-S and CBDA-S, the genes are italicized as *THCAS* and *CBDAS*.

Dai *et al.* (2012) sequenced ITS1-ITS2 from a drug-type plant in Xīnjiāng Region, and a fiber-type plant from Yúnnán. They compared the sequences to two ITS1-ITS2 sequences in Genbank (accessions from India and Germany), with *Humulus japonicus* as outgroup. The Indian and German sequences were identical, forming a clade with Xīnjiāng; longer branch lengths placed Yúnnán sister to the drug strains.

Early studies of *THCAS* polymorphisms did not include accessions from Central Asia (Kojoma *et al.* 2006, McPartland and Guy 2010, Rotherham and Harbison 2011). Russo *et al.* (2008) probed ancient DNA from a Central Asian plant, found in a tomb in Yánghǎi, Xīnjiāng Region. They cloned two *THCAS* sequences, named *China F* and *China F(h)*. *China F* was identical to other *THCAS* sequences deposited at Genbank by Kojoma *et al.* (2006). *China F(h)* differed from *China F* at two SNPs, and did not match any sequences deposited at Genbank.

Van Bakel *et al.* (2011) sequenced *THCAS* and *CBDAS* in an "Indica-dominant hybrid" named "Purple Kush". They identified two copies of *THCAS*: one with 99% nucleotide identity to the recognized *THCAS* sequence, the other with 91% identity that they considered a *THCAS* pseudogene. "Purple Kush" also contained three *CBDAS* pseudogenes, with premature stop codons and frame shift mutations. They proposed a two-loci model of cannabinoid inheritance—two tightly-linked yet separate *THCAS* and *CBDAS* loci—rather than the single-locus model (de Meijer *et al.* 2003).

Onofri *et al.* (2015) searched for SNPs in *THCAS* and *CBDAS* in 18 accessions of fiber- and drug-type plants. Several accessions harbored more than one polymorphism, indicative of multiple copy numbers. Drug-type "Haze" expressed four polymorphic *THCAS* sequences. Three Afghani "hashish landrace" accessions expressed three polymorphic sequences between them. One sequence with four SNPs was unique to Afghani plants and a Moroccan "hashish landrace." Collectively, *THCAS* averaged 2.9 SNPs per sequence, and *CBDAS* averaged 5.7 SNPs per sequence. The greater variation in *CBDAS* sequences was taken as evidence that it was the ancestral synthase gene, from which *THCAS* evolved. The authors offered alternative hypotheses: *THCAS* mutations may be more deleterious to plant survival, or *CBDAS* has been under greater positive selection pressure. Onofri also measured THC and CBD content in the 18 strains. They used this data to identify which polymorphisms expressed fully-functional enzymes, and which polymorphisms expressed enzymes with less (or no) catalytic ability.

Weiblen *et al.* (2015) sequenced *THCAS* and *CBDAS* from "Skunk #1 (a drug-type hybrid) and 'Carmen' (a fiber-type cultivar). "Skunk #1" yielded three *THCAS* homologues and two *CBDAS* pseudogenes. 'Carmen' yielded a *CBDAS* sequence and three *THCAS* pseudogenes. They constructed a gene tree of these sequences, and performed a *dN/dS* (*i.e.,* Ka/Ks) analysis. The *dN/dS* results showed that *CBDAS* was targeted by selection pressure—specifically the *CBDAS* pseudogenes found in "Skunk #1" and "Purple Kush". They proposed that selection pressure at *CBDAS* was primarily responsible for divergences in THCA/CBDA ratios, contrary to selection pressure at *THCAS,* as proposed by McPartland and Guy (2010), based on their Ka/Ks analysis.

Weiblen and colleagues grew "Skunk #1 and 'Carmen', their $F_1$ hybrid, and $F_1$-selfed $F_2$ progeny, and measured THC and CBD content (percent dry weight in female flowering tops, using capillary GC-FID). THC/CBD ratios in the $F_2$ population followed a 1:2:1 Mendelian pattern, consistent with two tightly-linked yet separate *THCAS* and *CBDAS* loci. Lastly, they genotyped the same plants using 103 AFLP markers and 16 microsatellite STRs. AFLP and STR markers, combined with cannabinoid data, enabled quantitative trait locus (QTL) mapping of several phenotypic traits: THC%, CBD%, THC/CBD ratio, and total THC+CBD quantity.

McKernan *et al.* (2016) generated amplicons for *THCAS* in thirteen medical strains, including four high-CBD strains. Only one strain had a single THCA-S copy, the rest had multiple polymorphic copies. "Chemdog" expressed five *THCAS* copies—one with a stop codon, one likely inactive, and three putatively active copies. Among the high-CBD strains, "Sour Tsunami" expressed six *THCAS* copies—three with frameshift mutations (stop codons), one inactive, one unknown, and one putatively active homologue.

Many studies have used DNA primers and PCR (polymerase chain reaction) to amplify *anonymous* DNA sequences. May of these markers show higher variability than gene sequences, thus a greater ability to discriminate between individuals. However, random and anonymous DNA sequences cannot be historically ordered (polarized), so they cannot be used to construct true phylogenetic trees.

**RAPD** (randomly amplified polymorphic DNA) utilizes random primers, usually 10 nucleotides long, in a known but arbitrarily-chosen sequence. RAPD is fast and inexpensive, although it is sensitive to small differences in the PCR technique, and can be difficult to reproduce in other laboratories. Early RAPD studies did not include accessions from Central Asia for comparison, or they analyzed police seizures of unknown provenance (*e.g.,* Sakamoto *et al.* 1995, Gillan *et al.* 1995, Jagadish *et al.* 1996, Shirota *et al.* 1998, Mandolino et al. 1997, Forapani *et al.* 2001, de Meijer *et al.* 2003).

Piluzza *et al.* (2013) used RAPD to compare 19 accessions: one Afghani, five of Indian heritage, three "Skunk" hybrids, and an assortment of fiber-type plants from Europe and East Asia. The six RAPD primers utilized by Piluzza and colleagues detected DNA polymorphisms, and the haplotypes were clustered using a NJ algorithm. Plants of Afghani and Indian heritage fell into separate clusters. Each shared interesting clade-mates. The Afghani landrace was sister to a cluster of fiber-type plants. The cluster of Indian heritage plants was sister to the "Skunk" cluster.

Tang *et al.* (2013) used 14 RAPD primers to analyze 12 wild-type plants and three hemp cultivars from across China. Two accessions from Xīnjiāng Region formed a clade with four accessions from Yúnnán—three high-altitude wild-types, and the cultivar 'Yún má 1'. An accession from Tibet was basal to all other accessions.

A **microsatellite** is a short length of repetitive DNA (six nucleotides or under) with repeated motifs, such as a single nucleotide repeat (*e.g.,* AAAAA), a dinucleotide (*e.g.,* CACACA), or a trinucleotide repeat (*e.g.,* CAGCAG). Plant geneticists refer to them as simple sequence repeats (**SSRs**), and forensic geneticists and genetic genealogist refer to them as short tandem repeats

(STRs). Early SSR studies did not include accessions from Central Asia for comparison, or they analyzed police seizures of unknown provenance (Hsieh *et al.* 2003, Alghanim and Almirall 2003, Gilmore and Peakall 2003, Howard *et al.* 2008, Mendoza *et al.* 2009).

Gilmore *et al.* (2003) used five SSR markers to genotype six fiber-type accessions (four European, one Chinese, one from the Himalaya in India), and six drug-type accessions (three African, one Mexican, two hybrids), and one wild-type accession from Nepal. PCA analysis separated drug-type plants from fiber-type plants, and the wild-type from Nepal separated from both clusters.

Knight *et al.* (2010) used five SSRs to compare six seized plants identified as “Sativa” (n= 2) or “Indica” (n= 4) based on their morphology. PCA analysis clearly segregated “Sativa” plants from three of the “Indica” plants. The fourth “Indica” exhibited a unique genotype suggestive of a polyploid condition.

Dufresnes *et al.* (2017) used 13 SSRs to compare 30 fiber-type accessions and 18 drug-type accessions. Most of the drug accessions were hybrid “strains,” characterized as “mostly Indica” or mostly Sativa.” Two with traceable pedigrees were “pure Sativa” (Swaziland, Mexico), and one was “pure Indica” (Hindu Kush). Genetic relationships were detected using PCA and STRUCTURE. The latter is probabilistic software that identifies the optimal number of clusters (K) to divide a population, based on allele frequencies.

According to STRUCTURE, the data best fit K = 2 (two populations)—fiber-type varieties and drug-type strains. In their PCA of drug accessions, Swaziland and Hindu Kush fell on opposite ends of Axis I, with Mexico in the middle with the hybrids. They found counterfit strain names: Some samples with identical strain names were genetically distinct, whereas other strains with different names were genetically identical. Fiber-type accessions shared a closer relationship with “Sativas” than with “Indicas”. Other studies show the reverse (Piluzza *et al.* 2013, Sawler *et al.* 2015). Fiber-type accessions shared a closer relationship with “Sativas” than “Indicas”. Other studies show the reverse (Piluzza *et al.* 2013, Sawler *et al.* 2015).

Schwabe and McGlaughlin (2018) created primers for 10 SSRs based on a scan of the *Cannabis* draft genome for microsatellite repeat regions. They probed 30 drug-type strains, classified along a gradient of phenotypes (“Sativa”, mixed “Hybrid”, and “Indica”), based on Wikileaf, an online strain database. PCA showed no evidence of clustering among strains classified as “Sativa”, “Hybrid”, or “Indica”. STRUCTURE divided the population into K = 2; “Sativa” they assigned to Genotype 1, and “Indica” they assigned to Genotype 2. However, the reported phenotypes for many strains matched poorly with their genotypes assigned by STRUCTURE. For example, they genotyped four samples of “Purple Kush”, reportedly a cross of two Afghani landraces (thus 100% “Indica”). The four samples were heterogenous. Their mean genotype was 71% genotype 2 (“Indica”) and 29% genotype 1 (“Sativa”). One sample was 95% genotype 1 (a “Sativa”).

**Inter-simple sequence repeat** (ISSR) is a mirror of SSR. Like SSR, it targets repeated motifs. But in this case, the primer consists of dinucleotide or trinucleotide repeats, and the amplification product is the DNA sequence that follows a microsatellite. Several ISSR studies

did not include accessions from Central Asia for comparison, or they analyzed police seizures of unknown provenance (Kojoma *et al.* 2002, Hakki *et al.* 2007, Kayis *et al.* 2010).

Punja *et al.* (2017) used seven ISSR primers to probe seven accessions that growers in British Columbia considered genuine landraces. Five were accessions of South Asian heritage, plus "Ketoma" (*i.e.,* Ketama) was from Morocco, and "Afghani" was from Afghanistan. A dendrogram (NJ tree) of banding patterns placed "Ketoma" and "Afghani" in a clade, sister to accessions from Mexico ("Jarilla") and Africa ("Kilimanjaro").

Zhang *et al.* (2013) used ISSRs to sort genetic relationships among 27 "Chinese native hemp cultivars." A NJ tree (UPGMA) segregated the cultivars in a pattern consistent with geographic distribution. The 27 cultivars segregated into three groups at a genetic distance of 0.366; group B included only two cultivars—the only Xīnjiāng accession, and one from neighboring Qīnghǎi. The results are peculiar; Zhang (2009) previously analyzed the 27 accessions using RAPD markers, and published an entirely identical NJ tree.

**Amplified fragment length polymorphism** (AFLP) uses restriction enzymes to cut DNA into fragments. For example, the *Eco*RI enzyme slices DNA wherever the sequence 5'-GAATC-3' occurs. Then short single-stranded segments of DNA called adapters are attached to a subset of fragments. Primers for the adapters are used to amplify the fragments ligated to the adapters. Amplified products differ in size because of nucleotides substitutions in the restriction sites, which add or eliminate enzyme targets. The various-sized fragments are separated into bands by electrophoresis.

Early AFLP studies did not compare accessions that inform our research (Peil *et al.* 2003, Miller Coyle *et al.* 2005, Datwyler and Weiblen 2006, Kriese 2007, Weiblen *et al.* 2015). Liu *et al.* (2010) used AFLP to analyze 49 accessions from across China. Two accessions from Xīnjiāng Region were sister to two accessions from Gānsù. That clade was sister to a pair of accessions from Shǎnxī. Unlike their RAPD study results (Tang *et al.* 2013), the Xīnjiāng accessions were distal to accessions from Yúnnán.

**Whole genome sequencing (WGS)**

Advances in WGS have come rapidly: Sanger sequencing → Shotgun sequencing → Bridge PCR → Pyrosequencing → Illumina sequencing → High-throughput sequencing (HTS). HTS or "Next-generation" sequencing uses massively parallel arrays which produce millions of short reads. The reads are assembled into longer contigs and, ultimately, into scaffolds and complete genomes. The human genome was sequenced by 2001. Its haploid size is currently estimated to be 3,234.8 Mb, with 20,412 protein-coding genes. A decade later, the *Cannabis* genome was sequenced by two groups. Van Bakel *et al.* (2011) assembled Illumina reads into scaffolds of "Purple Kush", 'FINOLA', and 'USO-31'. Medicinal Genomics Corporation (2011) assembled "Chemdawg" using Illumina GA IIx, and assembled "LA Confidential" using Roche 454.

"Next-Gen" is now considered "Second-Generation," replaced by "Third-Generation" sequencing. Third-Gen, also known as long read sequencing, improves the assembly of repetitive elements for gene identification. A Third-Gen-assembled genome identified 42,052 protein coding genes in *Cannabis* (Grassa *et al.* 2018). Long reads have identified *THCAS* and *CBDAS*

sequences embedded in repetitive elements, and found significant variation in the copy number of *THCAS* and *CBDAS* among different strains and cultivars (Grassa *et al.* 2018, McKernan *et al.* 2018, Laverty *et al.* 2019).

Van Bakel *et al.* (2011) published the first classification of *Cannabis* utilizing WGS data. They assembled a SNP database by aligning the "Purple Kush" sequence to SNPs in 'FINOLA', 'USO-31', and "Chemdawg". A subset of SNPs filtered for quality was utilized to generate a NJ tree for the four accessions. The dendrogram separated the two fiber-type cultivars from the two drug-type strains. Since the dendrogram was based on a clustering technique (the NJ algorithm), it was phenetic, rather than a true phylogenetic analysis.

"Reduced representation" approaches can be used to obtain an evenly distributed sample of SNPs across the genome. Reduced representation shotgun (RRS) utilizes random ligation to shear the genome into fragments, which are amplified and sequenced with an Illumina platform. Genotyping-by-sequencing (GBS) utilizes restriction enzymes to cleave the genome at sequence-specific cut sites. The resulting fragments are amplified and sequenced with an Illumina platform. Sequenced reads can then be analyzed *de-novo* or aligned to a reference genome. The RRS method samples a far greater percentage of the genome than GBS.

Sawler *et al.* (2015) obtained 124 samples (43 fiber-type cultivars and 81 drug-type strains); drug-type strains were classified along a gradient of ancestry proportions (percent "Sativa" vs. percent "Indica"), based on reports in online strain databases. They used GBS for SNP discovery and genotyping, by coupling *Ape*KI restriction enzymes with Illumina machines, and aligning GBS fragments to the "Purple Kush" genome sequence. After quality filtering, they identified an astounding 14,031 SNPs. Analysis of SNPs with PLINK 1.9 (a WGS analysis toolset) segregated fiber-type samples from drug-type samples on Axis 1 of the PCA. The clusters of "Sativa" and "Indica" overlapped on Axis 1. Similar results were obtained with fastSTRUCTURE, where data from all 124 samples best fit K = 2 (fiber-type plants and drug-type plants).

A second analysis limited to "Sativa" and "Indica" (9,776 SNPs) still showed overlap in the PCA; proportional ancestry in each sample correlated moderately ($r^2$= 0.36) with PCA axis 1. Sawler concluded that the two populations "may represent distinguishable pools of genetic diversity, but that breeding has resulted in considerable admixture between the two." The inability to separate "Sativa" and "Indica" and the poor correlation of reported ancestry was also due to counterfeit strain names: In a comparison of 17 paired samples with the same strain name, six pairs (35%) were dissimilar, and shared more genetic similarity with other strain names. Some "Sativa" strains, reportedly landraces of South Asian heritage (*e.g.,* Jamaican, South African), expressed a genetic structure determined as 100% "Indica" by fastSTRUCTURE.

Medicinal Genomics Corporation (2015) used RRS sequencing to identify 100,000-200,000 SNPs. These data were used to generate a NJ tree with "Purple Kush," 'Finola,' 'USO-31,' and 50 hybrid strains. Henry (2015) utilized open-access RRS data to evaluate 28 hybrid strains, using Adegenet 2.0. K-partition optimized at K = 1. PCA clustering with a subset of 42 most-informative SNPs, however, clearly segregated three clusters: "Sativa" (n= 17) "Indica" (n= 9), and two fiber-type strains.

Lynch *et al.* (2015) sequenced 60 accessions using WGS, and added to this dataset seven previous WGS reads (Van Bakel *et al.* 2011, Medicinal Genomics Corporation 2011). For SNP-calling they aligned sequences with the "Purple Kush" genome sequence. Then they sequenced 182 accessions using GBS, with *ECo*RI and *Mse*I restriction enzymes, for SNP-calling. A subset of 195 accessions from WGS and GBS shared 2,894 SNPs for analysis.

Two algorithms were used to K-partition the 195 accessions. FLOCK recognized K = 3 groups, and fastSTRUCTURE optimized the data at K = 2. The authors went with FLOCK, because of perceived shortcomings in fastSTRUCTURE, although these perceived differences are contentious (Anderson and Barry 2015). The K = 3 groups were recognized as WLD biotypes (*e.g.,* "Afghan Kush," "Chemdawg"), NLD biotypes (*e.g.,* "Durban Poison," "Easy Sativa"), and a polyphyletic "hemp" group (*e.g.,* 'Finola,' "AC/DC," Chinese hemp, Dagestan plants).

Phylogenetic relationships between the 195 accessions were visualized in an unrooted NJ *network*—a dendrogram with reticulation (divergence and hybridization among ancestral lineages). The network revealed aspects of ancestry not captured by a simple bifurcating tree, such as genetic admixtures between Chinese hemp and feral hemp plants in the USA.

Next they pooled WGS data with GBS data from Sawler *et al.* (2015), with 4,105 SNPs in common, and generated a neighbor-joining network with 210 accessions. These data revealed a second NLD biotype clade, consisting of Indian, Southeast Asian, and South African populations, along with various "Haze" hybrids. This clade may represent accessions of Indian heritage with minimal admixture from WLD biotypes. Lastly, they pooled WGS data with both GBS datasets, a total of 289 accessions, filtered for overlapping SNPs (only 45 SNPs in common—the two GBS datasets were generated with different restriction enzymes), and used MEGA6 to generate a NJ tree.

Soornie *et al.* (2017) collected germplasm from naturalized plants in Iran. They genotyped a female and male from 35 locations. They also genotyped an accession from Afghanistan (a female and male), plus 13 fiber-type accessions from the Wagenengin and IPK-Gatersleben seed banks (a female and male from each). They used GBS, with the *Apek*I restriction enzyme and an Ilumina platform, mapped to the "Purple Kush" and 'FINOLA' genomes, to identify 24,710 high-quality SNPs.

For a PCA they added GBS data from Sawler *et al.* (2015)—43 fiber-type and 71 drug-type accessions. The scatterplot clearly separated fiber-type and drug-type accessions, with Iranian plants between the two, splitting into two clusters—a larger cluster closer to drug-type accessions, and a smaller cluster closer to fiber-type accessions. Collectively, $F_{ST}$ was higher (more genetic isolation) between Iranian and fiber-type accessions ($F_{ST} = 0.086$) than between Iranian and drug-type accessions ($F_{ST} = 0.039$). A second PCA, limited to Iranian samples, revealed two clusters. The smaller one consisted of samples from Iran's western states (bordering Europe). The larger cluster consisted of samples from central and eastern Iran, "these accessions likely represent remnants of cultivated germplasm from other regions, possibly through migration of *Cannabis* from neighboring countries like Afghanistan and Pakistan." The authors

concluded that Iranian cannabis "may represent a distinct genetic lineage… Although Iranian cannabis is not likely a subspecies, it does represent a genetically unique variety of marijuana."

Grassa *et al.* (2018) combined data from 367 HTS genomes (Sawler *et al.* 2015, Lynch *et al.* 2015, Soornie *et al.* 2017, and three of their own), mapped to their reference genome ("CBDRx"). They filtered SNPs that failed the Hardy-Weinberg test, or showed linkage disequilibrium, arriving at 2,051 SNPs. PCA segregated three populations, labeled "hemp," "marijuana," and "naturalized" (the latter consisting mostly of Soornie's Iranian accessions) along Axis 1. ADMIXTURE (K = 3) modeled the 367 admixed genomes based on idealized donor populations from Axis 1 of the PCA. Based on this modeling, the ancestry of "Skunk#1" was estimated to be 78% "marijuana" and 22% "naturalized." Pretty accurate: "Skunk #1" is a hybrid of (Afghani x Colombian Gold) x Acapulco Gold (de Meijer 1999).

## Discussion

### SF.11. Crossbreeding *C. indica* and *C. afghanica* into "Sativa" and "Indica"

Many phenotypic differences between South Asian and Central Asian *Cannabis* are due to environmental adaptation and natural selection, as detailed in the main document. The two populations evolved under different climatic conditions, in separate floristic regions harboring unique flora (Fig. 7 in the main document).

Floristic regions became "centers of diversity" (CODs), where wild-type plants were domesticated by humans. Vavilov (1935) named eight CODs around the world. Vavilov's "Indian COD" (most of India excluding the northwest) corresponds geographically to the Indian floristic region (Djamali et al. 2012). Vavilov's "Central Asiatic COD" (northwest India, Pakistan, Afghanistan, Tajikistan, Uzbekistan, and western Xīnjiāng), corresponds geographically to the Irano-Turanian floristic region (Djamali et al. 2012). Compare Fig. S14 below with Fig. 7 in the main document).

Vavilov presciently named *both* the Indian COD and the Central Asiatic COD as two locations where *Cannabis indica* was domesticated separately. Wild-type *C. himalayensis* and domesticated *C. indica* came from the Indian COD. Wild-type *C. asperrima* and domesticated *C. afghanica* came from the Central Asiatic COD.

Figure S14. Geographic range of Vavilov's Indian COD (labeled II) and Central Asiatic COD (labled III). Image cropped from Vavilov (1935).

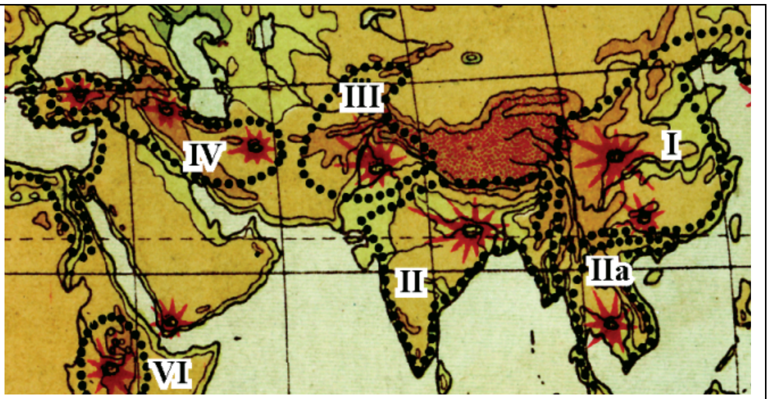

Phenotypic traits of *C. indica* and *C. afghanica* diverged further under human selection, for example the THC/CBD ratio (Fig. 2 in main document). This arose, in part, to differences in drug manufacturing: In Central Asia, bulk processing of *hashīsh* likely did not permit the selection of individual high-THC plants (de Meijer 1999). Arid and cold climatic conditions in Central Asia enabled the invention of sieved *hashīsh.* As early as 850 AD physicians in Baghdad used Chinese silk fabric, traded via Central Asia, to sieve mixtures of herbal medicines (Sābūr ibn Sahl 2003).

Making sieved *hashīsh* was not possible in South Asia: warm and humid conditions caused glandular trichomes to burst readily, and gum-up sieves. Instead of sieved *hashīsh*, people in India manufactured *gañjā*. Growers could select stray seeds from choice plants with potent psychoactivity, thereby increasing the THC/CBD ratio over the course of a millennium (Clarke and Merlin 2013). This practice may be surmised from instructions given in *Ānandakanda*, written *ca.* 1300 AD, which describes how to manipulate individual plants to make *gañjā* (Meulenbeld 1989). The first attestation of *gañjā*, as *gañjākinī*, appears in *Dhanvantarī Nighaṇṭu,* where it is synonymized with *bhaṅgā,* and described as an intoxicant (Meulenbeld 2002). The text is difficult to date—somewhere between the 10$^{th}$ and 13$^{th}$ centuries (Sharma 1970).

*C. indica* disseminated from India around the same time. Ibn al-Baitār was a Muslim physician from Spain who moved to Egypt in the 1230s. He wrote a large pharmacopoeia, which included two kinds of European hemp—*qinnab* and *qinnab barrī* (Dioscorides's *κάνναβις* and *κάνναβις ἀγρία*). He added, "There is a third kind of hemp called *qinnab hindī* [Indian hemp]. I met it in Egypt where it was planted in gardens, where it also known as *al-hashīsha*, the herb" (Ibn-al-Baitār 1883). His text strongly suggests that Indian landraces had reached the Middle East by then.

This was the first documentation, but not the last. Fast-forwarding to the 20$^{th}$ century, Clarke (1998) interviewed a Lebanese farmer from the Bekaa Valley who introduced germplasm from India into the Middle East.

Germplasm reached Africa at an early date. Archaeological evidence in Africa (pipes unearthed in Ethiopia that tested positive for cannabinoids) dates to 1320 ±80 (Dombrowski 1971, van der Merwe and Blank 1975).

Al-Bīrūnī, who lived in India from 1020-1030, commented on extensive trade between India and East Africa at that time: "The reason why in particular *Somanath* has become so famous is that it was a harbor for seafaring people, and a station for those who went to and fro between *Sufala* in the country of the *Zanj* and China" (Al-Bīrūnī 1910). *Somanath* is on the Gujărāt coast, *Sufala* is likely Sofala (Mozambique), and *Zanj* is where the Zanzibar coast gets its name.

Crawfurd (1856) proposed that *Cannabis* was introduced to Southeast Asia (Sumatra) by Telugu-speaking sailors from southeastern India. They used the word *gañjā*, a word still attested in Sumatra. Crawfurd adds that this occurred prior to the arrival of Arabs, who used the word *hashīsh*. Sūfīs brought Islam to Indonesia by the 12$^{th}$-13$^{th}$ centuries (Azra 1994).

Backer (1948) says the Chinese made note of *Cannabis* cultivation in Java 900 years ago, but he does not cite a primary source. Burney *et al.* (2004) hypothesized a far-older date: *Cannabis* fossil pollen appeared in Madagascar around 200 BCE, and Madagascar was settled by Malagasy-speaking people from Borneo; Burney suggested that colonists in outrigger canoes brought *Cannabis* to Madagascar from Borneo. The hypothesis has one hitch—there's no evidence of *Cannabis* growing in Borneo two millennia ago.

Mott (1986) suggested that *Cannabis* arrived in Brazil around 1650, a century after the African slave trade began. The names for *Cannabis* in Brazil—*diamba, liamba, riamba, maconha, pango*—have origins in the languages of Angola. Spix and Martius (1828) observed *Cannabis* in Brazil, "The seed is probably imported from India and Africa, the plant is different from Europe by elongated and relatively narrower leaves." *Cannabis* likely spread north from Brazil, to Columbia and Mexico. Jamaican *gañjā* traces to indentured servants from India, who began arriving in Jamaica in 1845 (Rubin and Comitas 1975).

The Middle East is likely the first place where South Asian and Central Asian landraces were crossbred. As mentioned in section SF.8, Ibn Taymīyah (1263-1328) described refugees from Central Asia with *hashīsh* flooding into Damascus ahead of Mongol armies. Ibn Taymīyah mentions a specific type of *hashīsh* called *ghubayrā*'. Rosenthal (1971) points out that Ibn Taymīyah implied that *ghubayrā*' was something different from plain *hashīsh*. Rosenthal gives its etymological meaning as "little dust-colored one," which sounds like the powdery texture of sieved *hashīsh*.

Three centuries after Ibn Taymīyah, several French travelers in Persia described "Uzbeks" from Bukhārā visiting Isfahān, who introduced *tchouhersse* or *chars* (section SF.8). Tavernier (1676) described *tchouhersse*, "qui est comme la fleur ou plûtost un cotton laineux qui se trouve sur la cheneviere" [which is like the flower or rather a woolly cotton which is on the hemp seed], which to us sounds like glandular trichomes sieved for *hashīsh*. Kaempfer (1712) clearly described *hashīsh* hash in Persia. *Tsjers*, "the pollen of flowers," was shaken off plants and sieved through a cloth.

Perhaps Central Asian germplasm was carried to the Middle East much earlier: The Scythians came from Central Asia, and penetrated the Neo-Assyrian Empire during the reign of King Sargon II (722-705 BC). Around when the Scythians arrived in Assyria, a new word appeared in Neo-Assyrian cuneiform, which transliterates as *qunubu.* Linguists consider *qunubu* a loanword from the Scythians (Loewenthal 1926, Chantraine 1968, Seidel 1989). Herodotus, writing in 440 BC, famously described the Scythians cultivating *κάνναβις* and fumigating it for psychoactive effects (Herodotus 2007).

Documents from the 19th century hint at germplasm of *C. indica* and *C. afghanica* moving between South and Central Asia. Johnson (1867) stated that "Kashmirees and Baltees of Iskárdo…have settled in Yarkand in large numbers, for the cultivation of the *charas* plant, which they have brought to great perfection." It would not surprise us if "Kashmirees and Baltees" (of Kashmir and Baltistan, in South Asia) transported germplasm to Yarkand (Xīnjiāng Region, Central Asia), or vice versa.

It is certain that Kashmiris adopted Central Asian sieving techniques. Jacquemont (1861) said that Kashmiris "extract the intoxicating dust." Baden-Powell (1868) described *garda charas,* produced by shaking dried plants and collecting the dust that settled on a fine cloth. A Kashmiri dictionary by Grierson (1916) stated that *gard bang* was also called *churu charas, churu* meaning "powdered."

Clarke (1998) proposed that *hashīsh* producers fled the Khanate of Bukhārā (present day Uzbekistan) to Afghanistan, after Russians invaded Bukhārā in 1866. Burkhārā and Samarqand were famed for excellent *hashīsh* (known as *charas* or *nasha*). Perhaps the fleeing *hashīsh* producers carried germplasm. Clarke (1998) suggested another wave of *hashīsh* producers fled from Xīnjiāng (China) to Afghanistan in 1935, after the Chinese cracked down on *hashīsh* production. He proposed they took the Chitral route, over the Baroghil Pass, down the Chitral and Kunar rivers, onward to Mazar-e Sharīf. As they migrated though the Kunar River valley, Clarke claims they acquired germplasm (Kunar is where Vavilov collected *C. indica* var. *afghanica*), and they grew it for *hashīsh* in Mazar-e Sharīf. A nice hypothesis, but Vavilov's herbarium contains *afghanica* plants that he collected in Mazar-e Sharīf a decade earlier, in 1924 (herb. WIR).

Afghanis and Indians also migrated between Xīnjiāng and India, and may have transported germplasm. Bellew (1873a) met a caravan of Bajaur Afghanis with 16 horses laden with *charas*, enroute from Yarkand to Ladakh in India. Rizvi (1999) described a small community of Indian merchants living in Yarkand involved in the *nasha* trade back in the 1930s. Rizvi interviewed an old man who recalled that the traders were mostly from Hoshiarpur in Punjab. The morphology of plants from Hoshiarpur, examined by Turner *et al.* (1979), was consistent with *C. afghanica*: a branchy habit, dull green leaves, and broad leaflets. The THC+CBN/CBD ratio of Hoshiarpur plants ($\bar{x} = 0.25$) was well below the mean for all 20 accessions ($\bar{x} = 22.5$), again suggestive of *C. afghanica*.

**Recent (post-1970) crossbreeding of *C. indica* and *C. afghanica***

The conscious hybridization of *C. indica* and *C. afghanica* began in California, after Sam Selgnij collected Afghanistan germplasm in 1971 (Clarke 1998). Afghani plants expressed unique traits compared to "traditional" USA drug plants, of South Asia heritage, obtained from Mexico, Columbia, and Thailand (Clarke 1981). Crossing Afghani plants with plants of South Asian heritage yielded offspring with hybrid vigor (Clarke 1998, de Meijer 1999). This heterosis effect arises between genetically distant crosses. However, indiscriminate breeding of hybrids x hybrids often resulted in "garbage" (Clarke 1998). Within 15 years of Afghani germplasm reaching California, unhybridized plants of Indian heritage, as well as unhybridized Afghani landraces, had become difficult to obtain (Clarke 1987).

Alarmingly, foreign germplasm has corrupted South Asian and Central Asian landraces in their former centers of diversity (see the main document). Beisler (2006) boasted of importing "Mexican Gold" into Kabul around 1972. Pietri (2009) stated that Beisler crossed "Acapulco Gold" with Afghani landraces in Afghanistan. Evidence suggests hybrids have penetrated

Xīnjiāng Region. Sun *et al.* (2017) analyzed the cannabinoid content in eight samples from Xīnjiāng. Three had no measurable CBD content, and the THC/CBD ratio of others was as high as 32.9. This ratio is 25-fold higher than Xīnjiāng plants measured 25 years ago (Zhang and He 1992, Zhu *et al.* 1992, Cao *et al.* 1993).

Samuels (2008) reported, "growers in Califonia and elsewhere are producing hundreds of exotic new strains…the percentages [of "Indica" versus "Sativa" in a hybrid] are arbitrary, because of all the cross-breeding. You take a 'Blueberry' and you cross with a 'Kush' and you go back into 'Trainwreck,' and how do you get a percentage from that?" Samuels summarizes, "The variety of buds being sold as 'Kush' has proliferated to the point where even the most catholic-minded botanist would be hard pressed to identify a common plant ancestor."

**SF.12. Intermediate forms; East Asian hemp**

**Intermediate forms**

Subspecies and varieties are capable of interbreeding and gene exchange, by definition under the BSC, which gives rise to intermediate forms. This issue was addressed by Small and Cronquist (1976), who described intermediate forms between domesticated *C. sativa* var. *sativa* and its wild-type *C. sativa* var. *spontanea.*

Intermediate forms between domesticated plants and wild-type plants are commonly seen in herbarium specimens: between domesticated *C. indica* and wild-type *C. himalayensis*, and between domesticated *C. afghanica* and wild-type *C. asperrima* (see list of herbarium specimens). Intermediate forms in may represent domesticated populations in the process of de-domestication, or true hybrid forms. Measuring their THC/CBD ratios might address this question—domesticated populations in the process of regaining wild-type achenes should retain their THC/CBD ratios, whereas hybrids would not.

Intermediate forms also arise between domesticated *C. indica* and domesticated *C. afghanica* (see list of herbarium specimens). Hybrids of *C. indica* and *C. afghanica* could have arisen naturally. Their ranges are not far apart, and migratory animals could have carried seeds from one area to the other. For example, the Himalayan Greenfinch (*Hypacanthis spinoides*), whose "favourite food is the seed of the wild hemp," ranges from the Samana Mountains (on the border of Afghanistan) to Manipur in northeastern India (Whistler and Kinnear 1949). Wilson and Korovin (2003) counted 793 seeds of *Cannabis sativa* in the crop of an Oriental turtledove, *Streptopelia orientalis,* caught on the border of Russia and Kazakhstan. *S. orientalis* is migratory, and its range includes northern India, Afghanistan, Turkestan, and China.

More likely, humans orchestrated crosses between *C. indica* and *C. afghanica*, as we detailed in the previous section. These hybridization events, and their taxonomic consequences, arise in crop plants with multiple domestication events, such as beans, wheat, and rice (Pickersgill *et al.* 2001). "It is probable that the cultivation of hemp arose simultaneously and independently in several places" (Vavilov 1926).

Intermediate forms between *C. indica* and *C. afghanica* are seen in herbarium specimens from Pakistan, which is the center of diversity for *C. sativa* subsp. *indica*—all four varieties occur there. Pakistan is a crossroads of three floristic regions (Ali and Qaiser 1985).

Intermediate forms dominate herbarium specimens from the Middle East (Egypt, Turkey, Syria, Lebanon, Palestine, Israel, Jordan, Iraq, western Iran, see list of herbarium specimens). They could be hybrids between *C. indica* and *C. afghanica,* or hybrids of either with European fiber-type hemp (*C. sativa* subsp. *sativa*). Dewey (1914) noted that the Middle East is one of the few places where farmers grew *Cannabis* for three products: fiber, seed, and drugs. The plants likely hybridized. Pulewka (1950) observed that the narcotic content in Turkish plants could not be determined by their morphology. European hemp has grown in the Middle East for millennia. Pliny, writing 70 AD, said the best hemp came from Rosea (northwest of Rome) and two Roman colonies in Turkey, Alabandius and Mylasa (Pliny 1961).

Hybridization between European and Asian *Cannabis* may have predated human intervention. Much of the Middle East is located in the Mediterranean Floristic Region (Takhtajan 1986). The MFR hugs Mediterranean coastlines in Europe (Portugal to Greece), Asia (western Turkey, Lebanon, Israel, Palestine), and Africa (from Morocco to Egypt). Manafzadeh *et al.* (2013) proposed that xerophytic plants from the Irano-Turanian Floristic Region (ITFR) migrated into the MFR, with range shifts facilitated by climate change and topographic heterogeneity. The ITRF encompasses arid regions in West Asia (eastern Turkey, Syria, northern Iraq) and Central Asia—from Iran to Xīnjiāng (Takhtajan 1986). The ITRF is the center of origin of *C. asperrima*, so it could have migrated with other ITRF plants to the MFR.

Mayari et al. (2008) describe a westward migration of "oriental" steppe plants into Bulgaria, facilitated by arid conditions during the Weichselian Late-Glacial period, 15,000–13,000 BP. During that same period, fossil pollen of *Cannabis* spread throughout Europe, including Bulgaria (McPartland *et al.* 2018). The range of European *Cannabis* would have overlapped with "oriental" *Cannabis*. The Weichselian Late-Glacial period was followed by the warm-and-wet late Holocene, when pollen consistent with wild-type *Cannabis* retreated from Bulgaria.

**East Asian hemp**

Vavilov (1931) considered *C. sativa* one of the few plants indigenous to Central Asia that spread to China. Some botanists assigned Chinese fiber-type hemp to *Cannabis indica* (Humboldt 1811, Dupin 1831, Itier 1846, Hedde 1848, Tatarinov 1856). Others recognized it as a separate species, named *Cannabis chinensis* (Fisher 1810, Winterschmidt 1818, Delile 1849, Koch 1854, Heuzé 1860), or *Cannabis gigantea* (Naudin 1850, Vilmorin 1851, Jomard 1852), or *Cannabis sinensis* (Macgowan 1850, Vilmorin 1892).

The trinomial *Cannabis sativa gigantea* has been used (Martius 1832, Alefeld 1866). De Candolle (1869) reduced the taxon to a variety or sub-variety, as *C. sativa δ chinensis*. Pabst (1887) erected the varietal name *C. sativa* var. *chinensis*. Siebert and Voss (1896) used the taxon *C. sativa* f. *gigantea.* Hoffmann (1944) coined the taxon *Cannabis* var. *indica* subvar. *gigantea.*

Hillig (2004) gave the name "*C. indica* hemp biotype" to East Asian hemp. He worked hard to parse hybrids from his studies, because East Asian hemp has been extensively crossbred with European hemp. Genetic evidence (allozyme variation) revealed that East Asian hemp was genetically diverse, and comprised a subset of the *C. indica* genepool, rather than European *C. sativa* (Hillig 2005a).

Morphologically, East Asian hemp separated from European hemp in a canonical analysis, (Hillig 2005b). In a canonical analysis of terpenoids, East Asian hemp segregated from European hemp (Hillig 2004). The cannabinoid profile of East Asian hemp differed from from European hemp (Small and Beckstead 1973, Hillig and Mahlberg 2004), particularly regarding high levels of cannabichromene (CBC) and cannabigerol monomethylether (CBGM).

It is worth clarifying that Central Asian *Cannabis*, not East Asian hemp, is the drug consumed in Xīnjiāng Region. Archaeological evidence of cannabis drug use in Xīnjiāng is the oldest in the world. At Yánghǎi, a drug-use context is secured by the presence of processed leaves and female flowering tops—no branches, and no male flowers. Some plant material was stored in a wooden bowl with a smooth inner surface, suggesting its use in a mortar and pestle arrangement. The material may have been pulverized, like *bhāng* is prepared today (Jiang *et al.* 2006). The tomb dates to 766-416 cal. BCE (Flad *et al.* 2010), or 630 cal. BCE (Beck *et al.* 2014). Only 30 km away at Jiāyī, which dates to 800-520 cal. BCE, the tomb's occupant was covered with a ceremonial "burial shroud" of thirteen nearly whole female *Cannabis* plants (Jiang *et al.* 2016).

On the other side of Xīnjiāng, Ren *et al.* (2019) unearthed 10 wooden braziers at Jirzankal necropolis, near Qūshìmàn Village, that date to 500 cal. BCE. The charred braziers contained burnt stones and pyrolyzed residues, from which cannabinol (CBN) was identified by gas chromatography-mass spectrometry. The authors report no CBD in the pyrolyzed residues, which seems questionable, and their methods have shortfalls. They used GC, rather than LC (HPLC, UPLC), which is the method of choice for cannabinoids. They obtained GC-MS spectra and ran the spectra through the National Institute of Standards and Technology (NIST) database for identification. The NIST database can be unreliable for small peaks. Dos Santos *et al.* (2019) found that spectral peaks in their GC-MS analysis of CBD only showed 75% similarity with the NIST database. Ren *et al.* (2019) report no peaks at m/z 231, identified by NIST as characteristic for CBD. However, m/z 231 is part of the THC fragmentation pathway (Leghissa *et al.* 2018), so the absence of m/z 231 seems peculiar. Lastly, CBD present in the plant may have been lost in the pyrolyzed residue—pyrolysis converts some CBD to CBN (Küppers *et al.* 1973).

Ren *et al.* (2019) interpret the lack of CBD as evidence of careful selective breeding, which is an over-interpretation of their data. The *Cannabis* plants at Yánghǎi, which predate the Jirzankal site by perhaps a century, had nearly equal amounts of THC and CBD (Russo *et al.* 2008, Ma *et al.* 2011). People from South Asia selectively bred landraces with potent psychoactivity for 900 years, but they never completely eliminated CBD. That kind of breeding effort wasn't achieved until the 20th century.

Drug use in China also occurs in Yúnnán. Tourist spots in Yúnnán frequented by western trekkers have gained a reputation for local weed, although the plants are low potency (Clarke 1999). Zhu *et al.* (1992) analyzed 28 plant populations throughout Yúnnán, and measured THC+CBN content in female flowering tops, which averaged 0.78%.

Clarke and Merlin (2016) proposed that the Héngduàn Mountains served as a glacial refugium for *Cannabis indica* during the Pleistocene. The Héngduàn covers much of present-day

western Sìchuān, extending into parts of Tibet, Yúnnán, and Burma. They hypothesized that *Cannabis indica* germplasm moved from its Héngduàn refugium to Central Asia, under the aegis of humans. This hypothesis was adopted by Duvall (2015), despite his overall savaging of Clarke and Merlin (2016) in a book review (Duvall 2014).

We propose carriage in the opposite direction, under the aegis of Sayyid Ajjal Šams al-Din ʿOmar (1211-1279). Sayyid Ajall came from Bukhārā, and was appointed Yúnnán's provincial governor by Kublai Khan. Several lineages of Huí people (Chinese Muslims) trace back to him. Sayyid Ajall founded Kūnmíng, introduced new agricultural technologies, and constructed irrigation systems (Chén 1997). Perhaps his entourage introduced Bukhārān germplasm.

We have examined a score of herbarium specimens from Yúnnán. Although phenotypically variable, their morphology is not consistent with Central Asian landraces: some have broad leaflets, but oblanceolate shapes are lacking, with short petioles. The pistillate inflorescences are elongated and loosely structured, with a high perigonal bract-to-leaf index. Yúnnán plants are not consistent with South Asian plants either: their leaflets are rarely narrow, and the serrations are too coarse. Their seeds are too large (see list of herbarium specimens).

Drug-type plants grown in Southeast Asia may represent introgression between *C. indica* and East Asian hemp. Crévost (1917) erected *C. gigantea* for plants grown in Tonkin and North Annam, Vietnam. Chevalier (1944) considered *C. sativa* var. *macrosperma* from Vietnam a unique variety, separate from var. *chinensis* and var. *indica.* Likely it was a hybrid. Some herbarium specimens from Tibet resemble hybrids of var. *chinensis* and var. *indica.*

## SF.13 Practical applications and future directions

For a classification to have practical application, it needs to work. Central and South Asian landraces are best distinguished by phytochemistry—their THC/CBD ratios and terpenoid profiles—and this requires chromatography, which is not very practical. Botanists aimed this criticism at Small and Cronquist (1976). However, folk taxonomists can distinguish between "Sativa" and "Indica" by their organoleptic properties, without resorting to chromatography.

Classification is best served if plants can be distinguished by morphology. Folk taxonomists claim to discriminate between "Sativa" and "Indica" (Fig. 1 in main document). This discrimination, however, is based on a suite of morphological characters—plant height, branching habitus, inflorescence density, leaflet shape, and leaflet color.

Any one trait by itself exhibits a continuum amongst populations, and no single trait meets the "75% rule." Patten and Unitt (2002) proposed that if 75% or more of a population expresses a character that separates it from all (98%) individuals of an overlapping population, it qualifies as a subspecies. *C. sativa* subspecies meet the 75% rule with phytochemistry. A morphological marker that approximates the 75% rule is the leaflet L/W ratio. Oblanceolate leaf shape (the WP/L measure) distinguishes most Central Asian landraces from South Asian landraces. A biserrate leaflet margin, although not always present, is largely restricted to South Asian landraces.

**Future directions**

Molecular analyses of unhybridized *C. indica* and *C. afghanica* will provide information no longer available in "Sativa" and "Indica". An unambiguous genetic "barcode" differentiating *C. indica* and *C. afghanica* awaits discovery. Onofri *et al.* (2015) identified a THCA synthase gene with four SNPs that was unique in two Afghani accessions and a Moroccan "hashish landrace." It was not present in 16 other accessions of fiber- and drug-type plants, but it was also absent in a third Afghani accession.

In taxonomic groups subject to hybridization and introgression, differences in morphology may be more reliable than DNA sequences (Moffat *et al.* 2015). Similarly, phytochemical profiles may differentiate between hybrid taxa that go undetected with molecular markers (Kirk *et al.* 2004).

Several quantitative phenotypic traits await measurement. In three high-THC hybrids, Potter (2009) examined floral leaves (he called them bracts), and compared their proximal and distal areas in regards to capitate-stalked glandular trichome (CSGT) density. Proximal areas had a mean of 2.75 CSGTs /$mm^2$, and distal areas had none. We *qualitatively* compared proximal and distal areas in regards to CSGT density. A *quantitative* comparison of *C. indica* and *C. afghanica* should come next.

The CSGT density on perigonal bracts has not been adequately quantified. Qualitatively, CSGT density is visibly greater in *C. afghanica* than *C. indica.* This correlates with total cannabinoid content per weight of inflorescence biomass—which is significantly greater in *C. afghanica* than *C. indica* (Hillig and Malberg 2004). CSGT *size* also awaits a careful analysis. Small and Naraine (2015) found that CSGT gland heads averaged 129 µm diameter in "high-THC medical marijuana" (hybrids) compared to 81 µm in fiber cultivars.

Perianth structure deserves more attention. The perianth flakes off easily in Central Asian accessions, even in wild-type plants (Fig. S15). In South Asian accessions, the perianth remains adherent to achenes, even in many domesticated plants (Fig. 3c in main document). The adherent perianth is a synapomorphy (ancestral trait) shared with *Humulus lupulus* (Fig. S15). The derived (flaky) state is seen in *indica-afghanica* hybrids (Fig S15 C, D). Only two microscopic studies on perianth pigmentation have been done (Briosi and Tognini 1894, Small 1975).

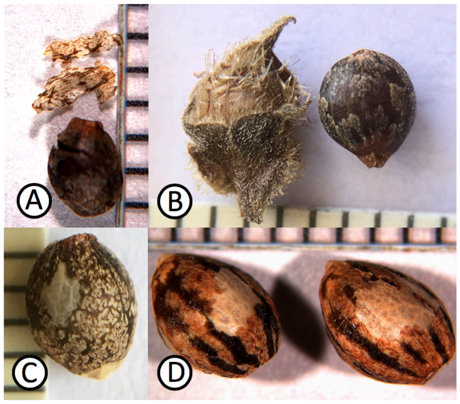


Figure S15. Perianth structure.
A. Perianth flaked off achene in *asperrima* lectotype. B. *Humulus lupulus* achenes, one enclosed in its cystolith-covered bract, the other exposed with adherent perianth (China, *Davis* 1962, K). C. Flaky perianth in "Skunk #1" hybrid (via Watson, *Hillig* 1996, IND). D. Flaky perianth in Lebanese landrace, likely a hybrid (via CPRO, *Hillig* 1996, IND).

CSGT gland head abscission should be investigated. Hammond and Mahlberg (1977) showed that CSGTs "possess a dehiscence mechanism whereby the gland head separates from the multicellular stalk when physically disrupted." They used the phrase "dehiscence mechanism," although "abscission mechanism" would be more accurate. Potter (2009) reported two mechanisms by which gland heads detach from stalk cells. In Central Asian plants cultivated for *hashīsh*, Ledbetter and Krikorian (1975) noted "a very tenous connection attaches the globoid head to the multicellular stalk."

Clarke (1987) stated that CSGT gland heads of *C. afghanica* abscised after a hard rain, whereas they remained attached in *C. indica.* This may reflect natural selection—Autumn rains are rare in Afghanistan, compared to monsoonal South Asia. Artificial selection may have also played a part—freely abscising gland heads are favorable for producing sieved *hashīsh*. In South Asia, where *gañjā* is consumed, persistant gland heads are favorable.

Lastly, cystolith trichomes might also provide taxonomically useful characters. Sharma (1975) reported differences in cystolith size and density (Table S5). Ballard (1915) reported differences in cystolith morphology between "native American *Cannabis*" and "foreign *Cannabis*." In contrast, Nakamura (1969) found no significant differences in cystolith structure amongst different populations of *Cannabis*, "with respect to geographic origin or whether it is called *C. indica, C. americanus*, or *C. sativa."*

## Representative herbarium specimens

*"Taxonomic work based on herbarium specimens is the essential first step in any systematic study"* (McAllister 1999).

### *Cannabis sativa* subsp. *indica* var. *indica*

**Holotype:** INDIA: likely Pondicherry, *Lamarck*, no date, annotated "*Chanvre rapporte de l'Inde par M. Sonnerat*" (P).

ANGOLA: Huila, *Powell-Cotton*, 1937 (BM).

BANGLADESH: Rajshahi ("Bengal, Nowgong, Rajshahye"), *Clarke,* 20.II.1877 (BM). Dhaka, Manik Ganj, *Soejarto & Rahma*, 7.VI.1978 (K).

BRAZIL: banks of Nhamunda River near the Amazon, *Traill*, VI.1874 (K, sinsemilla specimen). Bahia State, *Glocker in herb. Shuttleworth*, 1842 (BM, sinsemilla specimen). São Paulo, Campinas, *Dias*, 25.II.1987 (US).

CHINA: Xīzàng Region, Chumbi Valley, Lingmuthang, *Rolunoo Lepcha*, 12.IX.1912 (GH).

COLOMBIA: Antioquia Department, near Medellín, *Gabriel*, I.1941 (US). Cundinamarca Department, Bogotá. *Idrobo*, 14.IV.1955 (US). Cundinamarca Department, Bogotá, *Plowman*, 6.VI.1972 (GH).

ETHIOPIA: Harar, *ICRO*, 27.III.1959 (K).

GABON: bank of Gabon River, *Mann*, VII.1861 (K).

INDIA: No location, "*Cannabis indica trifolia*, Bangue Indorum," *Plukenet*, undated, *ca.* 1696 (BM). No location, "*Cannabis indica*," *Willdenow B-W 18367*, undated, *ca.* 1800 (B). Uttar Pradesh, Kanpur ("Sudallapur"), *Buchanan-Hamilton,* no date (K, Wallich 4665a). Chennai ("Madras"), *Herb.Madras,* no date (K, Wallich 4665d). Karnataka State, Kodagu ("Coorg"), Metz, 1849, Hohenacker exsiccate

(LE). Kerla, Munnar, reprod. by *Hillig*, 1996, Indiana University (IND In-1). Meghalaya, Shillong, *Choudhury*, 21.II.1962 (GH). Sikkim, “Herb. Ind. Dr. Hook. fil & Thomson, alt. 0-7000 feet, Regio: trop.”, *J. D. Hooker*, no date (BM). Tamil Nadu (“Madas”), Coimbatore, *Bircher*, 28.VII.1893 (K). Tamil Nadu, Dindigul, *Matthew*, 12.XII.1986 (K). Tamil Nadu, Hosur, *Yeshoda*, 2.VIII.1932 (NY). West Bengal, Kolkata (“Calcutta”), *Wallich*, no date (K, Wallich 4665f).

IRAN: No location, *E.A. Willmott*, 31.VII.1854 (K). Tabriz, *A.C.Trott*, 1934 (K).

JAMAICA: No location, *W. March,* 1896 (K). No location, reprod. by *Hillig*, 1996, Indiana University (IND Jm-2).

MALAWI: Mulanje, *Phippa*, 29.III.1960 (K).

MEXICO: Paringaricutiri (possibly Parangaricutiro, Michoacán), *Lumholtz,* VIII.1896 (US). No location, cult.@ Univ.Mississippi, *Quimby M-A, Schultes, Plowman & Lockwood 64M*, 31.VII.1972 (ECON).

MOZAMBIQUE: Luaho, bank of Zambesi River, *Kirk & D. Livingston*, 25.V.1858 (K).

NEPAL: Kathmandu, *Wallich*, 1821 (K, Wallich 4665g). Jumla District, Karnali Valley, Lapha, *Polunn*, 1952 (GH). No location, cult.@ Univ.Mississippi, *Quimby Ne-B, Schultes, Plowman & Lockwood 58B*, 31.VII.1972 (ECON).

PAKISTAN, Punjab, banks of Chenab river, “Herb. Ind. Or. Hook. fil & Thomson”, *Thomson*, X.1846 (K). Punjab, Rawalpindi, *Steward,* II.1913 (NY).

RÉUNION: No location, *Boivin*, Plantae insula Borboniae no. 1107, 1847-1852 (LE).

RUSSIA: Dagestan, Hassav-jurt, *Nekrassova,* 13.IX.1928 (GH).

SIERRA LEONE: No location, cult.@ Univ.Mississippi, *Quimby Si-A, Schultes, Plowman & Lockwood 69,* 31.VII.1972 (ECON).

SINGAPORE: grown from confiscated seed, *Henderson*, IX.1930 (K).

SOUTH AFRICA: No location, cult.@ Univ.Mississippi, *Quimby A-A, Schultes, Plowman & Lockwood 50A*, 31.VII.1972 (ECON). Orange Free State & Transvaal, *Molyneux*, 1880 (BM). Transkei region, reprod. by *Hillig*, 1996, Indiana University (IND SA-2).

TANZANIA: Kahama District, Mininga, *Speke & Grant,* IV.1867 (K). Ubena, *Davies,* 10.IV.1932 (K). Mbeya, *Burt,* 18.X.1936 (K).

THAILAND: Chang Mai, *Smith* - 21.VIII.1993 (GH). No location, cult.@ Univ.Mississippi, *Quimby Ti-C, Schultes, Plowman & Lockwood 88E*, 31.VII.1972 (ECON). Sakon Nokhon, reprod. by *Hillig*, 1996, Indiana University (IND Th-2).

UGANDA: No location, reprod. by *Hillig*, 1996, Indiana University (IND Ug-1).

UNITED STATES: Massachusetts, Boston, “Michoacan”, *Plowman,* 1970 (GH). Boston, “Alcapulgo gold”, *Plowman,* 1970 (GH). Boston, “Columbian,” *Plowman,* 1972 (GH).

VIETNAM: No location, cult.@ Univ.Mississippi, *Quimby V-A, Schultes, Plowman & Lockwood 86G*, 31.VII.1972 (ECON).

ZIMBABWE: Northern Rhodesia, *Fanshawe,* 22.III.1958 (K). No location, reprod. by *Hillig*, 1996, Indiana University (IND Zm-1).

### ***Cannabis sativa*** **subsp.** ***indica*** **var.** ***himalayensis***

**Neotype:** INDIA: Himachal Pradesh, Shimla or Kinnaur (“Himalaya Boreal. Occident., Regio Temp.”), *T. Thompson*, 1847 (GH).

BANGLADESH: Dhaka, *C.B.Clarke*, 20.V.1872 (LE). East Bengal, *Griffith*, ca. 1835 (LE). East Bengal, Jessore, *Clarke*, 30.VI.1874 (US).

BHUTAN: Gamri Chu (east of Trashigang), *Grierson & Long*, 19.VI.1979 (K). Trashigang, *Grierson & Long*, 18.VI.1979 (K).

BURMA: Mandalay Region, Toong Dong Mountains near Inwa, *Wallich*, 24.XI.1826 (K, Wallich 4665h).

INDIA: No location, cult.@ Univ.Mississippi, *Schultes, Plowman & Lockwood 78A*, 31.VII.1972 (ECON). No location, "Herb. Ind. Or. Hook. fil. & Thomson, Gangetic plain, Regio trop., alt. 1000 ft." *Thomson*, no date (GH). Himachal Pradesh, Dalhousie, *C.B. Clarke,* 30.IX.1874 (LE). Himachal Pradesh ("Punjab"), Bashahr State, *Koelz*, 6.X.1933 (US). Haryana ("Punjab"), Karnal jungle, *Drummond* - 27.IV.1886 (K). Himachal Pradesh, Simla, *Felding*, 1867 (K). Jammu & Kashmir, above Bandipora 6500', *B.B. Osmaston*, 4.VIII.1928 (K). Jammu & Kashmir, near Srinagar, *Schlagintweit,* "Herbarium Schlagintweit from India and High Asia: Western Himálaya", X.1856 (LE). Punjab, Pathankot, *Koelz,* 1936 (GH). Punjab, Keshopur, *Koelz*, 7.III.1931 (NY). Sikkim, Lhonak valley *G.H. Cave*, IV.1915 (BM). Uttarakhand, Kumaon, *Strachey & Winterbottom*, 1846-1849 (GH). Uttarakhand, Garhwal, Sukhi, *Schlagintweit*, 5.X.1855 (GH). Uttarakhand, Kumaon, Tejum-Girgaon, *Rao*, 10.VI.1958 (GH). Uttarakhand, Tehri-Garhwal, Harsil, *Huggins*, 24.IX.1953 (BM). Uttarakhand, Garhwal, Dehra Dun, *Singh,* 1928 (NY). Uttarakhand, Garhwal, Dehra Dun, *Naithani*, 15.V.1973 (GH). Uttarakhand, Sivalik Hills near Dehradun, *Skvorksov & Proskuriakova*, 24.IX.1972 (LE). Uttar Pradesh, Bareilly, *Roxburgh*, 1796 (K, Wallich herbarium 4665 b). Uttar Pradesh, Saharanpur district, reprod. by *Hillig*, 1996, Indiana University (IND In-5).

NEPAL: East Nepal, Myong Valley, *J. D. Hooker,* 21.X.1848 (K). East Nepal, Janakpur, Choarma, *Yonekura*, 3.VIII.1985 (BM). Kaski District, Pokhara, *McPartland*, X.1986 (BPI). Myagdi District, Tatopani, *McPartland*, X.1986 (BPI). Mustang District, Kalopani, *McPartland*, X.1986 (BPI). Mustang District, Kalopani, reprod. by *Hillig*, 1996, Indiana University (IND 891192). Mustang District, Lete, *Miehe*, 12.X.1977 (BM). Sunsari District, *Himal. Bor. Occ. regio. temp., Hooker & Thomson*, 1848 (GH).

PAKISTAN: Gilgit-Baltistan ("Tibet"), Hasóra, Das via Góltere or Nugá, *Schlagintweit,* 20.IX.1856 (GH). Azad Kashmir, Chakothi ("Cashmere, Chakath to Uri"), *Young*, VIII.1880 (BM). Azad Kashmir, Shekh Bela, *Steward*,14.VIII.1953 (BM). Federally Administered Tribal Area, Tirah, reprod. by *Qazilbash*, at Khyber Pakhtunkhwa, Peshawar, XII.1975 (GH). Khyber Pakhtunkhwa, Kaghan Valley, Mahandri (next to Kashmir), *Afzel & Chudhari,* 29.IX.1977 (GH).

**Intermediate forms, *indica—himalayensis***

Many of these herbarium specimens contained fruits truly intermediate between domesticated forms (var. *indica*) and wild-type forms (var. *himalayensis*), such as Wallich's collection in Burma. Other herbarium specimens lacked diagnostic fruits; they were either male plants or immature plants. Lacking fruits, their status as *indica* or *himalayensis* could not be clearly determined, such as Wallich's collection in India.

AFGHANISTAN: "Affghanistan," No location, *Griffith 1304*, Herb. Lemann (K).

INDIA: Karnataka, Sudallapur, *Hamilton*, 5.I.1809 (K, Wallich 4665a). West Bengal, Kolkata ("H.B.C."), *Hamilton*, 21.I.1815 (K, Wallich 4665a). Chennai ("Madras"), *Wallich*, *Herb.Madras,* no date (K, Wallich 4665d). Himalaya region ("1-7000 ft"), *T. Thompson* ("Herb. Ind. Or. Hook. fil. & Thomson"), no date (LE). Karnaka (Mysore), Sudallapur, *Hamilton*, 5.I.1809 (K, Wallich 4665a). Kashmir, No location, *Aitchison 15,* 8.XII.1875 (K). Sikkim, "East Nepal," *Hooker*, 1851 (K). Uttarakhand, Kumaon, *Strachey & Winterbottom* - 1846-1849 (K). Uttarakhand, Almora District,

reprod. by *Hillig*, 1996, Indiana University (IND In-2). Uttarakhand, Pauri Garhwal, reprod. by *Hillig*, 1996, Indiana University (IND In-4).

PAKISTAN: Chitral, Drosh, *Stainton*, 3.VI.1958 (BM).

SOUTH AFRICA: Pretoria, *Codd* - 1.IV.1954 (K).

***Cannabis sativa* subsp. *indica* var. *afghanica***

**Neotype:** AFGHANISTAN: Ghazni Province (formerly in Kandahar Province), near Gui-Akhen (Гуй-Ахен) village near Qala-i Murvardar (Кала-и Мурвардар), on the Ghazni-Kandahar road, *Vavilov*, 1924, from seed sown by *Serebriakova* in 1926 at North Caucasus Experiment Station, Maikop, Krasnodar Krai (WIR 609, 3945; labeled *C. sativa*).

**Epitype:** AFGHANISTAN: Kandahar Province, near Kandahar, *Schultes*, XII.13-20.1971 (ECON 26505).

AFGHANISTAN: No location, cult.@ Univ.Mississippi, *Quimby Af-A, Schultes, Plowman & Lockwood 52T*, 31.VII.1972 (ECON). No location, "G13" strain, reprod. by *Hillig*, 1996, Indiana University (IND Af-4). Badakhshan Province, Fayzabad, *Vavilov*, 1924, reprod. by *Serebriakova* in 1925 at Kamennaya-Stepnaya Exp. Station, Voronezh Oblast (labeled *C. sativa*, WIR 589,3948). Ghazni Province (formerly in Kandahar Province), Gui-Akhen (Гуй-Ахен), *Vavilov*, 1924, reprod. by *Serebriakova* in 1925 at Kamenno-Stepnaya Exp. Station, Voronezh Oblast (labeled *C. sativa*, WIR 609, 7380). Ghazni, reprod. by *Hillig*, 1996, Indiana University (IND Af-2). Herat Province, Mārwā, *Vavilov*, 1924, reprod. by *Serebriakova* in 1928 at Ukrainian Station (labeled *C. sativa*, WIR 586, 3940). Jowzjan Province, Sheberghān, *Vavilov*, 1924, reprod. by *Serebriakova* in 1926 at North Caucasus Experiment Station, Maikop, Krasnodar Krai (labeled *C. sativa*, WIR 587, 3951). Kandahar Province, near Kandahar, *Schultes*, XII.13-20.1971 (ECON 26504). Kandahar Province, near Kandahar, *Schultes*, XII.13-20.1971 (ECON 26508). Nangarhār Province, Jalālābād, *Schultes*, XII.13-20.1971 (ECON 26500). Nangarhār Province, Jalālābād, garden of Hotel Spinghar, *Uotila*, 29.V.1972 (ECON).

CHINA: Xīnjiāng region, Shache (Yarkant), *Henderson,* 30.VIII.1870 (K, labeled *Cannabis sinensis*). Xīnjiāng Region, Turpan archaeological site (images in Jiang *et al.* 2006, Russo *et al.* 2008, Jiang *et al.* 2016). Xīnjiāng, near Ürümqi, Zimuquan Sheep Farm, *Guan Kezhen,* 7.VII.1957 (PE 00557992). Xīnjiāng, Aksu Prefecture, Kalpin County, *Li & Zhu,* 8.IX.1958 (PE 00761678).

IRAN: South Khorasan Province, Mud-e Dahanab, *Cherniakovskaya,* no date, reprod. by *Serebriakova* in 1928 at Ukrainian Exp. Station (WIR 695,3978). Khorasan Province, Nauzad, *Cherniakovskaya,* 22.IX.1925 (LE).

KYRGYZSTAN: Osh Oblast, Kurbantal-Mady, *Brshesijky*, 26.VI.1891 (LE)

PAKISTAN ("Afghanistan, Kurrum Valley"): Federally Administered Tribal Areas, Kurram Valley, Shalizan, *Aitchison*, 26.VI.1879 (K). No location, cult.@ Univ.Mississippi, *Quimby P-A, Schultes, Plowman & Lockwood 56H*, 31.VII.1972 (ECON). Khyber Pakhtunkhwa ("North-West Frontier Province"), reprod. by *Hillig*, 1995, Indiana University (IND Pk-1).

RUSSIA: Krasnodar Krai, Sochi Exp. Station, *Serebriakova*, collected 1922, reprod. 1925 at Kamennaya-Stepnaya Exp. Station, Voronezh Oblast, labeled *C. indica* (WIR 307,5573). Crimea, Yalta, Nikitsky Botanical Garden, *Serebriakova*, 1922 (WIR 3423).

TAJIKISTAN, Khujand District, Histevarz, *Cherniakovskaya 118* - 19.VII.1914 (LE)

TURKMENISTAN: Daşoguz Province, Shorkala, *Bukinich,* 23.VI.1924 (LE).

UZBEKISTAN: Tashkent, *Regel*, VIII.1876, labeled *C. sativa β vulgaris* (LE). Tashkent, *Vavilov*, reprod. 1925 at Kamenno-Stepnaya Exp. Station, Voronezh Oblast, *Serebriakova* (WIR). Ferghana Valley,

Andijan Region, *von Knorring & von Minkwitz 1423,* 4.VII.1911 (LE). Kokand, Bekabad; cult. *Hillig*, Bloomington Indiana, 11.X.1996, no. 00314 (IND).

### ***Cannabis sativa*** **subsp.** ***indica*** **var.** ***asperrima***

**Lectotype**: KYRGYZSTAN, Issyk-Kul Region, east of Lake Issyk Kul near Karakol, leg.: *A. Regel*; det.: *E. Regel*, 1.X.1877 (LE).

**Epitype**: AFGHANISTAN, Kunar Province, Chekhosarai (now Asadābād), *Vavilov*, 1924, from seeds sown by *Serebriakova* in 1927 at Pushkin Experiment Station, Detskoye Selo, St. Petersburg (WIR 599, 3952).

AFGHANISTAN: No location, "Herbarium of the late East India Company," *Griffith 4686*, 1863-4 (K). Badakhahan Province, near Fayzabad, *Podlech 1277453,* 25.IX.1965 (LE). Badakhahan Province, between Bahrak and Zebak, *Gibbons,* 13.VI.1971 (K). Kunar Province, "Chekosarai," *Vavilov*, 1924, reprod. by Serebriakova in 1927 at Pushkin Experiment Station, Detskoye Selo, St. Petersburg (WIR 599, 3953). Nuristān Province, Radam Obe near Wama, 1400 m, *V.N. Lebedev,* 14.VIII.1924 (LE). Nuristān, *J. K. Street,*13.X.1965, no. 235 (F). Nuristān, Kamdesh, *J. K. Street,*13.X.1965, no. 226 (F).

CHINA: Xīnjiāng Region, northeast slopes of Tiān Shān, 1650 m, Hūtúbì River drainage, *Morefield*, 25.VI.1989 (GH). Xīnjiāng Region, upper Bortala River, 5-6000 ft, *A. Regel,* 4.VIII.1878, labelled "*C. sativa β vulgaris"* (LE). Xīnjiāng Region, Ürümqi, *Qin Renchang,* 14.VII.1956 (PE 00557984).

KAZAKHSTAN: Almaty Province, Zailiyskiy Alatau, Karkara River, "Thian Shan Alatau transiliensis 4000-7000 ft," *Semenov-Tyan-Shansky No. 163,* 7.1857 (LE, label *H. lupulus* corrected to *C. sativa* by Herder). Almaty ("Vyernyi") Region, Zailiyskiy Alatau, *Sokalsky*, 14.VI.1907 (BM). Almaty Province, Kordai mountain pass, *Shuvalov & Bagmet,* 3.VII.1994 (WIR). Almaty Region, Zharkent, *Divnogorski*, 12.V.1907 (LE).

KYRGYZSTAN: Issyk-Kul Region, hills above Dschirgalan (Jyrgalan) River, leg.: *A. Regel*; det.: *E. Regel*, XI.1877 (LE). Issyk Kul Oblast, near Uital, Flora Iliensis, *Krassnow*, 1886 (LE). Issyk Kul Oblast, near Karakol ("Przhevalsky"), *Krassnow*, 1886 (LE). Issyk Kul Province, Karasay-Sirt valley, *Roborovsky*,1889 (LE). Batken Province, Leilek District, Katran, *von Minkwitz 731* - 19.VI.1913 (LE). Chuy Region, Suusamyr Valley, *Roshevitz,* 25.VII.1908 (LE). Chuy Region, Kemin District, Ak-Tüz, *Shuvalov & Smekalova,* 6.VIII.2006 (WIR).

PAKISTAN: Azad Kashmir ("Cachemire"), Shekh Bela, *Stewart*, 14.VIII.1953 (BM). Punjab, Lahore, *Chaudhuri*, III.1938 (GH).

TAJIKISTAN, Sughd Province, Chorku, 1150 m, *Ul'ianova,* 15.VII.1969 (ECON). Gorno-Badakhshan region, Darwas, Wanj ("Wandsch"), *Regel*, 13-25.XI.1881 (LE). Gorno-Badakhshan region, Shughnon, between Khorog and Dashtitem, *Fedtschenko* - 10.VIII.1904 (LE).

UZBEKISTAN: Fergana Region, Margilan, *Rodin & Arkad'ev 451* - 22.VI.1948 (LE).

### **Intermediate forms,** ***afghanica—asperrima***

Many of these herbarium specimens lacked diagnostic fruits; they were either male plants or immature plants. Lacking fruits, their status as *afghanica* or *kafiristanica* could not be clearly determined.

AFGHANISTAN: No location, cult.@ Ottawa, Canada, *Small,* 1973 (NY). Badakhshan Province, Wardij Valley near Fayzabad, *Furse* - 24.V.1964 (LE). Badakhshan Province, Bahrek village, *Balfour,* 9.VIII.1955 (BM). Laghman Province, *Koelz,* 24.V.1937 (US). Kunar Province, Asadābād ("Chekosarai"), *Vavilov*, 1924, reprod. by *Serebriakova* in 1927 at Ukrainian Exp. Station (labeled *C. sativa*, WIR 607,3954). Kunar Province, "near Chekosarai," *Vavilov*, 1924, reprod. by *Serebriakova* in

1925 at Kamennaya-Stepnaya Exp. Station, Voronezh Oblast (labeled *C. sativa* var. *spontanea*, WIR 606,4038). Kunar Province, "near Chekosarai," *Vavilov*, 1924, reprod. by *Serebriakova* in 1925 at Kamennaya-Stepnaya Exp. Station, Voronezh Oblast (labeled *C. sativa* var. *spontanea*, WIR 595,4044). Kunar Province, Barkandai ("Barkundi"), *Vavilov*, 1924, reprod. by *Serebriakova* in 1925 at Kamennaya-Stepnaya Exp. Station, Voronezh Oblast (labeled *C. sativa* var. *spontanea*, WIR 596,4046). Nuristān Province ("Kāfiristān"), No location, *Vavilov*, 1924, reprod. by *Serebriakova* in 1925 at Kamennaya-Stepnaya Exp. Station, Voronezh Oblast (labeled *C. sativa* var. *spontanea*, WIR 607,4034). Nuristān Province, Kāmdēsh, *Hewer*, 8.VI.1969 (K, LE). Nuristān Province, Wadau Valley mouth, *Thesieger,* 3.IX.1956 (BM). Balkh Province, Mazar-e Sharif, reprod. by *Hillig*, 1996, Indiana University (IND Af-7, 00010).

CHINA: Xīnjiāng Region, "by Ili River," *A. Regel,* 27.V.1877, labelled "*C. sativa β vulgaris"* (BM).

INDIA: Himachal Pradesh, Chamba, *Nair*, 20.VII.1964 (GH).

KAZAKHSTAN: East Kazakhstan Oblast, Kokbekty River, *Herder,* 10.VII.1863 (LE); Zhambyl Oblast, Moyunqum Desert, *Golbek,* 20.V.1910 (LE).

KYRGYZSTAN: Issyk Kul Oblast, near Karakol ("Przhevalsky"), *Roshevitz,* 25.VII.1908 (LE).

PAKISTAN: Chitral, *S.M. Toppin 534*, VII.1908 (K, "abundant 4500-9000"). Khyber Pakhtunkhwa, Hazara, Sarai Saleh, *no name,* 1975 (ECON).

UZBEKISTAN: Fergana Region, Kokand, near the town, *Verner,* 23.V.1910 (LE). Fergana Region, near Margilan, *Dessiatoff*, 4.V.1913 (LE). Samarqand Region, near Samarkand, *Nevsky,* 15.V.1878 (LE).

## Intermediate forms, *indica—afghanica*

Intermediate forms between var. *indica* and var. *afghanica* occur in Pakistan, a crossroads of three floristic regions. Herbarium specimens with intermediate forms from Afghanistan are also fairly common.

AFGHANISTAN: No location, cult.@ Univ.Mississippi, *Quimby Af-A1, Schultes, Plowman & Lockwood 51J*, 31.VII.1972 (ECON). Nuristān Province ("Kāfiristān"), *Vavilov*, 1924, reprod. by *Serebriakova* in 1925 at Kamennaya-Stepnaya Exp. Station, Voronezh Oblast (LE 607,5667, labeled *C. sativa* var. *spontanea*).

INDIA: Kashmir, Bandipora, *Osmaston*, 4.VIII.1928 (K). Himachal Pradesh, Chamba, *Nair*, 20.VII.1964 (GH).

IRAN: Tabriz, 5500 ft alt, *A.C.Trott*, 1934 (K, "cultivated for drugs").

LEBANON: unknown location, via CPRO, reprod. by *Hillig*, 5.X.1996 (IND).

PAKISTAN: Khyber Pakhtunkhwa ("NWFP"), Swāt district, Saidu-Sharif, *Rodin,* 10.VIII.1952 (US). No location, cult.@ Univ.Mississippi, *Quimby A-A, Schultes, Plowman & Lockwood 55H*, 31.VII.1972 (ECON).

UZBEKISTAN: Bekabad, 5 km from Kokand, reprod. by *Hillig*, 1996, Indiana University (IND 00314).

## Examples of intentionally hybridized "strains"

NETHERLANDS: Wageningen, "Nederwiet", reprod. by *Hillig*, 1996, Indiana University (IND 891195).

UNITED STATES: California, "California Orange", reprod. by *Hillig*, 1996, Indiana University (IND 00137). California, "Skunk No. 1", reprod. by *Hillig*, 1996, Indiana University (IND 00143). California, "Haze", reprod. by *Hillig*, 1996, Indiana University (IND 00136). Washington, Seattle area, "Northern Lights", reprod. by *Hillig*, 1996, Indiana University (IND Af-9). Illinois, Champaign, *McPartland*, VIII.1981 (BPI).

**Examples of non-assignable collections: Mediterranean Floristic Region**

IRAQ: Baghdad, Rustam Experimental Farm, *Luest* - XI.1929 (K).

LEBANON: No location, reprod. by *Hillig*, 1996, Indiana University (IND 0072). Bekaa valley, Deir El Ahmar, *Harding et al.*, 24.VII.1945 (BM). Bekaa Valley, Baalbek, *A.C.Trott* - 26.VIII.1956 (K).

MOROCCO: South Marocco - *J.D.Hooker* - IV-V.1871 (K). Ketama, *Davis*, 19.VIII.1970 (BM). No location, *Vavilov,* 1926, reprod. 1927 at Pushkin Experiment Station, Detskoye Selo, St. Petersburg (WIR 1232, 3850). No location, cult.@ Univ.Mississippi, *Quimby Mo-B, Schultes, Plowman & Lockwood 71*, 31.VII.1972 (ECON, S:Cii);

PALESTINE: Jersusalem, "The American Colony," *Dinsmore*, 12.X.1913 (K, F).

SYRIA: Damascus, *Vavilov,* 1926, reprod. 1927 at Ukrainian Exp. Station (WIR, 3985,5682)

TURKEY: Eastern Anatolia Region, Erzurum, *Serebriakova,* 1925-27 reprod. 1928 at Ukrainian Station (WIR, 1264, 3901)

**Examples of non-assignable collections: Yúnnán Province, China**

Out of ~20 herbarium specimens examined from Yúnnán province, only a few have morphological characters consistent with drug-type plants: Yúnnán, Chiapieh, *Yü,* 3.X.1937 (GH). Yúnnán, Snow range, *Rock,* 1923-1924 (GH). Yúnnán, *Wang,* 1935-1935 (GH).

**Reference list for Supplemental File**


Ahmad M, Khan MA, Zafar M, Sultana S. 2013. *Practical handbook on biodiesel production and properties.* CRC Press, Boca Raton FL.

Aitchison JET. 1869. *A catalogue of the plants of the Punjab and Sindh*. Taylor and Francis, London.

Aizpurua-Olazizolo O, Sodaner U, Öztürk E, Schibano D, Simsir Y, Navarro P, Etxebarria N, Usobiaga A. 2016. Evolution of the cannabinoid and terpene content during the growth of *Cannabis sativa* plants from different chemotypes. *Journal of Natural Products* 79: 324-331.

Al-Bīrūnī (Sachau E, *trans*). 1910. *Alberuni's India : an account of the religion, philosophy, literature, chronology, astronomy, customs, laws and astrology of India.* K. Paul, Trench & Trübner, London.

Alefeld F. 1866. *Landwirthschaftliche Flora.* Wiegandt & Hempel, Berlin.

Alghanim HJ, Almirall JR. 2003. Development of microsatellite markers in *Cannabis sativa* for DNA typing and genetic relatedness analyses. *Analytical and Bioanalytical Chemistry* 376: 1225-1233.

Ali SI, Qaiser M. 1986. A phytogeographical analysis of the phanerogams of Pakistan and Kashmir. *Proceedings of the Royal Society of Edinburgh* 89B: 89-101.

Al-Muqaddasī (Collins BA, *trans*). 1994. *The best divisions for knowledge of the regions.* Garnet Publishing, Reading, UK.

Amaducci S, Colauzzi M, Bellocchi G, Venturi G. 2008a. Modelling post-emergent hemp phenology (*Cannabis sativa* L.): theory and evaluation. *European Journal of Agronomy* 28: 90-102.

Amaducci S, Zatta A, Raffanini, Venturi G. 2008b. Characterisation of hemp (*Cannabis sativa* L.) roots under different growing conditions. *Plant Soil* 313: 227-235.

Amaducci S, Zatta A, Raffanini M, Venturi G. 2008. Characterisation of hemp (*Cannabis sativa* L.) roots under different growing conditions. *Plant and Soil* 313: 227-235.

Anderson LC. 1974. A study of systematic wood anatomy in *Cannabis*. *Harvard Botanical Museum Leaflets* 24(2): 29-36.

Anderson LC. 1980. Leaf variation among *Cannabis* species from a controlled garden. *Harvard University Botanical Museum Leaflets* 28(1): 61-69.

Anderson EC, Barry PD. 2015. Interpreting the FLOCK algorithm from a statistical perspective. Molecular Ecology Resources 15: 1020–1030.

Anonymous. 1963. Экспозиция льна и конопли в Главном ботаническом саду (Exposition of flax and hemp in the main botanical garden). Бюллетень Главного ботанического сада 1963: 89-93.

Anonymous. 1994. *Alma Ata fusaria pathogenicity testing in Cannabis sativa. USDA report, May, 1994.* Document released under FOIA to Jeremy Bigwood.

Azra A. 1994. The transmission of Islamic reformism to Indonesia: networks of Middle Eastern and Malay-Indonesian ʿUlamā in the seventeenth and eighteenth centuries. Doctoral thesis, Columbia University, NY.

Babadijanov B. 1999. Biographies of Makhdūm-i A'zam al-Kāsānī al-Dahbīdī, Shaykh of the sixteenth-century Naqshbandīya. *Manuscripta Orientalia* 5(2): 3-8.

Backer CA. 1948. Cannabinaceae. *Flora Malesiana* 4(1): 222-223.

Badaev EA, Boltaev MD. 2013. Каннабиноидная сорная конопля Шуской долины (Cannabinoid weed hemp Chuska Valley). *Защита и карантин растений* 2013(3): 32-34.

Baden-Powell BH. 1868. *Hand-book of the economic products of the Punjab, Vol. 1.* Thomason Civil Engineering College Press, Roorkee.

Baker PB, Gough TA, Taylor BJ. 1980a. Illicitly imported cannabis products: some physical and chemical features indicative of their origin. *Bulletin on Narcotics* 32(2): 31-40.

Baker PB, Bagon KR, Gough TA. 1980b. Variation in the THC content in illicitly imported cannabis products. *Bulletin on Narcotics* 32(4): 47-84.

Baker PB, Taylor BJ, Gough TA. 1981. The tetrahydrocannabinol and tetrahydrocannabinolic acid content of cannabis products. *Journal of Pharmacy and Pharmacology* 33: 369-372.

Baker PB, Gough TA, Taylor BJ. 1982. The physical and chemical features of *Cannabis* plants grown in the United Kingdom of Great Britain and Northern Ireland from seeds of known origin. *Bulletin on Narcotics* 34(1): 27-36.

Ballard CW. 1915. Notes on the histology of an American cannabis. *Journal of the American Pharmaceutical Association* 4: 1299-1303.

Barbieri R. 1952. La "prefioritura" della canapa in Campania nell'annata 1952. *Agricoltura Napoletana* 19(7): 5-16 and (9): 5-29.

Barni-Comparini I, Ferri S, Centini F. 1984. Cannabinoid level in the leaves as a tool for the early discrimination of *Cannabis* chemiovariants. *Forensic Science International* 24: 37–42.

Bassani M, Pacini E, Franchi GG. 1994. Humidity stress responses in pollen of anemophilous and entomophilous species. *Grana* 33: 146-150.

Basu BC. 1894. Reports by Mr. B. C. Basu, assistant to the director of the Department of Land Records and Agriculture, Bengal, on the spontaneous growth of the wild hemp plant in the districts of Bhagalpur and Purnea. *Indian Hemp Drugs Commission Report* 3: 233-235.

Batten JH. 1855. From J. H. Batten, Esq., Senior Assistant Commissioner, to G. T. Lushington, Esq., Commissioner of Kumaon, No. 24, dated the 12th September 1840," pp. 32-37 in *Papers regarding the cultivation of hemp in India*. Secundra Orphan Press, Agra.

Bayly CA. 1996. *Empire and information: intelligence gathering and social communication in India, 1780-1870.* Cambridge University Press, Cambridge UK.

Bazzaz FA, Dusek D, Seigler DS, Haney AW. 1975. Photosynthesis and cannabinoid content of temperate and tropical populations of *Cannabis sativa. Biochemical Systematics and Ecology* 3: 15-18.

Beck U, Wagner M, Xiao Li X, Durkin-Meisterernst D, Tarasov PE. 2014. The invention of trousers and its likely affiliation with horseback riding and mobility: A case study of late 2nd millennium BC finds from Turfan in eastern Central Asia. *Quaternary International* 348: 224-235.

Beer R. 2007. *Reconstruction of the Holocene vegetation and fire history in the different forest types of Kyrgyzstan, Central Asia.* Doctoral thesis, University of Bern.

Beer R, Tinner W. 2005. *The Holocene vegetation history of Kyrgyzstan—first report about the coring expedition June 2003.* University of Bern, Switzerland.

Beisler J. 2006. *The bandit of Kabul.* Regent Press, Oakland, CA.

Bellew HW. 1873a. *Kashmir and Kashghar. A narrative of the journey of the Embassy to Kashghar in 1873-74.* Trübner & Co., London.

Belotsky M. 1936. Киргизская Республика: популярный очерк (*Kyrgyz Republic: a popular essay*). State Social and Economical Publishing House, Moscow.

Benelli G, Pavela R, Petrelli R, Cappellacci L, Santini G, *et al.* (5 additional authors). 2018. The essential oil from industrial hemp (*Cannabis sativa* L.) by-products as an effective tool for insect pest management in organic crops. *Industrial Crops and Products* 122: 308-315.

Bergius B. 1785. *Tal, om Läckerheter.* Johan Georg Lange, Stockholm.

Bertoli A, Tozzi S, Pistelli L, Angelini LG. 2010. Fibre hemp inflorescences: from crop-residues to essential oil production. *Industrial Crops and Products* 32: 329-337.

Bessey CE. 1915. The phylogenetic taxonomy of flowering plants. *Annals of the Missouri Botanical Garden* 2: 109-164.

Bestel S, Bao YJ, Zhong H, Chen XC, Liu L. 2018. Wild plant use and multi-cropping at the early Neolithic Zhuzhai site in the middle Yellow River region, China. *The Holocene* 28: 195-207.

Black H, Capler R. 2003. *Operational standards for the distribution of medicinal cannabis.* British Columbia Compassion Club Society, Vancouver, BC.

Blen L, *ed.* 1852. Ueber den chinesischen hanf tching-ma. *Archiv der Pharmazie (Zweite Reihe)* 72: 241-242.

Boch H. 1546. *Kreüter Bůch.* Wendel Rihel, Strassburg.

Bòcsa I, Karus M. 1997. *Der Hanfanbau: Botanik, Sorten, Anbau und Ernte.* C.F. Müller, Heildelberg.

Bond WJ, Woodward FI, Midgley GF. 2005. The global distribution of ecosystems in a world without fire. *New Phytologist* 165: 525–538.

Borna T, Salami SA, Shokrpour M. 2017. High resolution melting curve analysis revealed SNPs in major cannabinoid genes associated with drug and non-drug types of cannabis. *Biotechnology & Biotechnological Equipment* 31: 839-845.

Botoeva G. 2015. The monetization of social celebrations in rural Kyrgyzstan: on the uses of hashish money. *Central Asian Survey* 34: 531-548.

Boucher F, Paris M, Cosson L. 1977. Mise en évidence de deux types chimiques chez le *Cannabis sativa* originaire d'Afrique Du Sud. *Phytochemistry* 16: 1445-1448.

Breckle SW. 2007. Flora and vegetation of Afghanistan. *Basic and Applied Dryland Research* 1: 155-194.

Breckle SW, Koch M. 1982. Afghanische Drogen und ihre Stammpflanzen III. Haschisch und hanf. *Afghanistan Journal* 9: 115-123.

Breckle SW, Rafiqpoor MD. 2010. *Field guid to Afghanistan flora and vegetation.* Scientia Bonnensis, Bonn.

Bredemann G. 1952. Weitere Beobachtungen bei Züchtung des Hanfes auf Fasergehalt. *Der Züchter* 22: 257-269.

Brenneisen R, ElSohly MA. 1988. Chromatographic and spectroscoic profiles of *Cannabis* of different origins: Part 1. *Journal of Forensic Sciences* 33: 1385-1404.

Brickell CD, Alexander C, Cubey JJ, David JC, Hoffman MHA, *et al.* (3 additional authors). *eds.* 2016. *International Code of Nomenclature for Cultivated Plants, Ninth Edition.* International Society for Horticultural Sciences, Leuven, Belgium.

Briosi G. Tognini F. 1894. Intorno alla anatomia della canapa (*Cannabis sativa* L.) parte prima - organi sessual. *Atti della Instituto Botanico di Pavia* (Serie II) 3: 91-209.

Brodovskogo MI. 1872. "Замѣтки о земледѣліи въ Самаркандскомъ раіонѣ" (Notes on farming in the Samarkand region), pp. 233-261 in Trotskago VN, *ed. Русский Туркестан: Сборник изданный по поводу политехнической выстарки.* Katkov and Kom, Moscow.

Broséus J, Anglada F, Esseiva P. 2010. The differentiation of fibre- and drug type *Cannabis* seedlings by gas chromatography/mass spectrometry and chemometric tools. *Forensic Science International* 200: 87-92.

Brunschwig H. 1500. *Liber de arte distillandi de simplicibus : Das Buch der rechten Kuntst zü distilieren die eintzigē Ding*. Johann Grüninger, Strassburg.

Buchman HW. 1874. Examination of the fluid preparations of cannabis indicæ. *The Pharmacist* 7: 97-99.

Buffon GLL compte de. 1749. *Histoire naturelle, générale et particulière: : avec la description du Cabinet du Roi.* Imprimerie Royale, puis Plassan, Paris.
Burney DA, Burney LP, Godfrey LR, Jungers WL, Goodman SM, Wright HT, Jull AJT. 2004. A chronology for late prehistoric Madagascar. *Journal of Human Evolution* 47: 25-63.
Callaway JC. 2008. A more reliable evaluation of hemp THC levels is necessary and possible. *Journal of Industrial Hemp* 13: 117-144.
Camp WH. 1936. The antiquity of hemp as an economic plant. *Journal of the New York Botanical Garden* 37: 110-114.
Cao XP, Sun J, Yu ZS. 1993. 大麻毒品的检验方法. *Forensic Science and Technology* 1993(6): 15-18.
Casano S. 2005. *Ottimizzazione dell' agrotecnica della canapa (Cannabis sativa L.) per applicazioni di tipo farmaceutico.* Doctoral thesis, Università degli studi di Palermo.
Casano S, Grassi G, Martini V, Michelozzi M. 2011. Variations in terpene profiles of different strains of *Cannabis sativa* L. *Acta Horticulturae* 925: 115-121.
Cazzuola F. 1873. Relázione di alcuni esperimenti fatti sopra fibre tessili. *Nuovo Giornale Botanico Italiano* 5: 261-265.
Cazzuola F. 1875. *Il Regno vegetale tessile e tintoriale.* G. Barbèra, Firenze.
Central Asian Heritage Group. 2005. *O'zbekcha-Inglizcha Lug'at* [*Uzbek-English Dictionary*]. Bibl-Khanum Mosque, Samarkand, Uzbekistan.
Ceška A, Roemer H. 1971. A computer program for identifying species-relevé groups in vegetation studies. *Vegetatio* 23: 255-277.
Chantraine P. 1968. *Dictionnaire étymologique de la langue grecque, Tome I.* Éditions Klincksieck, Paris.
Chardin J. 1686**.** *Journal du voyage du chevalier Chardin en Perse et aux Indes Orientales.* Moses Pitt, London.
Chén BZ. 1997 [1623]. *Yuánshǐ Jìshì Běnmò* [*Major events in Yuán dynasty*]. Zhōnghuá shūjú, Běijīng.
Chevalier AJB. 1944. Histoire de deux plantes cultivées d'importance primordiale. Le lin et le chanvre. *Revue de Botanique Appliquée et d'Agriculture Coloniale* 24: 51-71.
Christison A. 1851. On the natural history, action, and uses of Indian hemp. *Monthly Journal of Medical Science* 13: 26-45, 117-121.
Christian GJ. 1855. "From G. J. Christian, Esq., Secretary to the Sudder Board of Revenue, N. W. Provinces, Agra, to H. C. Tucker, Esq., Commissionr of the Benares Division, No. 346, dated Agra, the 14th July 1854," pp. 13-14 in *Papers regarding the cultivation of hemp in India*. Secundra Orphan Press, Agra.
Clark MN, Bohm BA. 1979. Flavonoid variationin *Cannabis* L. *Botanical Journal of the Linnean Society* 79: 249-257.
Clarke RC. 1981. *Marijuana Botany.* And/Or Press, Berkeley, CA.
Clarke RC. 1987. *Cannabis evolution.* MS thesis, Indiana University, Bloomington, IN.
Clarke RC. 1998. *Hashish!* Red Eye Press, Los Angeles, CA.
Clarke RC. 1999b. Confusion of Cannabis in Yunnan. *Journal of the International Hemp Association* 6(2): 77-80.
Clarke RC. 2007. Traditional *Cannabis* cultivation in Darchula District, Nepal—seed, resin and textiles. *Journal of Industrial Hemp* 12(2): 19-42.
Clarke RC, Merlin MD. 2013. *Cannabis evolution and ethnobotany.* University of California Press, Berkeley.
Clarke RC, Merlin MD. 2016. *Cannabis* domestication, breeding history, present-day genetic diversity, and future prospects. *Critical Reviews in Plant Sciences* 35: 293-327.
Coffman CB, Genter WA. 1975. Cannabinoid profile and elemental uptake of *Cannabis sativa* L. as influenced by soil characteristics. *Agronomy Journal* 67: 491-497.
Cosentino SL, Testa G, Scordia D, Copani V. 2012. Sowing time and prediction of flowering of different hemp (*Cannabis sativa* L.) genotypes in southern Europe. *Industrial Crops and Production* 37: 20–33.
Crawfurd J. 1856. *A descriptive dictionary of the Indian Islands and adjacent countries.* Bradbury & Evans, London.

Crescini F. 1940. Formen von Hanf (*Cannabis sativa* L.). *Der Züchter* 12(5): 105-115.
Crévost C. 1917. Chanvre-*Cannabis sativa* (Lin.) et *Cannabis gigantea*. *Bulletin Économique de l'Indochine* n.s. 19: 613-614.
Culley TM. 2013. Why vouchers matter in botanical research. *Applications in Plant Sciences* 1(11): apps.1300076.
Cunningham JD. 1844. Notes on Moorcroft's travels in Ladakh and on Gerard's account of Kunawar. *Journal of the Asiatic Society of Bengal* 13(1): 172-253.
Dai J, Jiang L, Pi JH, Sun Q. 2012. 不同产地大麻的 ITS 全序列分析. *Journal of Luzhou Medical College* 35: 364-367
Darwin CR. 1859. *On the origin of species.* John Murray, London.
Darwin CR. 1868. *The variation of animals and plants under domestication,* 2 vols. John Murray, London.
Darwin CR (assisted by Darwin F). 1880. *The power of movement in plants.* John Murray, London.
Datwyler SL, Weiblen GD. 2006. Genetic variation in hemp and marijuana (*Cannabis sativa* L.) according to amplified fragment length polymorphisms. *Journal of Forensic Science* 51: 371-375.
Davies RH. 1862. *Report on the trade of the countries on the north-western boundary of British India.* Government Press, Lahore.
Davis TWM, Farmilo CG, Osadchuk M. 1963. Identification and origin determinations of *Cannabis* by gas and paper chromatography. *Analytical Chemistry* 35: 751-755.
De Backer B, Maebe K, Werstraete AG, Charlier C. 2012. Evolution of the content of THC and other major cannabinoids in drug-type *Cannabis* cuttings and seedling during growth of plants. *Journal of Forensic Sciences* 57: 918-922.
De Beaux O. 1875. Aspect general du territoire de Shang-Haï et de sa vegetation. *Actes de la Société Linnéenne de Bordeaux* 30: 59-130.
Debruyne D, Albessard F, Bigot MC, Moulin M. 1994. Comparison of three advanced chromatographic techniques for cannabis identification. *Bulletin on Narcotics* 44(2): 109-121.
de Candolle AP. 1813. *Théorie élémentaire de la botanique*. Deterville, Paris.
de Candolle ALP. 1869. *Prodromus systematis naturalis regni vegetabilis* 16 (1): 30-31.
de Candolle ALP. 1883. *Origine des Plantes Cultivées.* Baillière, Paris.
de Courtive 1848. *Hashisch étude historique, chimique et physiologique. Thèse a l'École de Pharmacie de Paris.* Edouard Bautruche, Paris.
Delile AR. 1849. *Cannabis chinensis. Annales des Sciences Naturelles; Botanique* 12: 365-366.
de Meijer EPM. 1993. Evaluation and verification of resistance to *Meloidogyne hapla* Chitwood in a *Cannabis* germplasm collection. *Euphytica* 71: 49-56.
de Meijer EPM. 1994a. *Diversity in Cannabis.* Doctoral thesis, Wageningen Agricultural University, Wageningen, The Netherlands.
de Meijer EPM. 1994b. Variation of *Cannabis* with reference to stem quality for paper pulp production. *Industrial Crops and Products* 3: 201–211.
de Meijer EPM. 1999. "*Cannabis* germplasm resources," pp. 133-151 in: Ranalli P, ed. *Advances in Hemp Research.* Haworth Press New York.
de Meijer EPM, Keizer LCP. 1996. Patterns of diversity in *Cannabis*. *Genetic Resources and Crop Evolution* 43: 41-52.
de Meijer EPM, Hammond KM. 2016. The inheritance of chemical phenotype in *Cannabis sativa* L. (V): regulation of the propyl-/pentyl cannabinoid ratio, completion of a genetic model. *Euphytica* 210: 291-307.
de Meijer EPM, van Soest LJM. 1992. The CPRS *Cannabis* germplasm collection. *Euphytica* 62: 201-211.
de Meijer EPM, van der Kamp, HJ, van Eeuwijk FA. 1992. Characterization of *Cannabis* accessions with regard to cannabinoid content in relation to other plant characters. *Euphytica* 62: 187-200.
de Meijer EPM, Bagatta M, Carboni A, Crucitti P, Cristiana Moliterni VM, Ranalli P, Mandolino G. 2003. The inheritance of chemical phenotype in *Cannabis sativa* L. *Genetics* 163: 335–346.

de Meijer EPM, Hammond KM, Micheler M. 2009. The inheritance of chemical phenotype in *Cannabis sativa* L. (III): variation in cannabichromene production. *Euphytica* 165: 293-311.

Demske D, Tarasov PE, Leipe C, Kotlia BS, Joshi LM, Long TW. 2016. Record of vegetation, climate change, human impact and retting of hemp in Garhwal Himalaya (India) during the past 4600 years. *The Holocene* 26: 1661-1675.

Deneef G. 1841. Encouraging prospects for attending to the hemp cultivation of Bengal. *Transactions of the Agricultural & Horticultural Society of India* 8: 449-451.

Der Marderosian A, Murthy S. 1974. Analysis of old samples of *Cannabis sativa* L. *Journal of Forensic Sciences* 19: 670-675.

Dewey LH. 1914. "Hemp," pp. 283-347 in: *USDA Yearbook 1913.* United States Department of Agriculture, Washington D.C.

Dewey LH. 1928. "Hemp varieties of improved type are result of selection," pp. 358-361 in: *USDA Yearbook 1927.* United States Department of Agriculture, Washington D.C.

de Zeeuw RA, Wijsbeek J, Breimer DD, Vree TB, van Ginneken CA, van Rossum JM. 1972. Cannabinoids with a propyl side chain in cannabis: occurrence and chromatographic behavior. *Science* 175:778-779.

Djamali M, Brewer S, Breckle SW, Jackson ST. 2012. Climatic determinism in phytogeographic regionalization—a test from the Irano-Turanian region SW and Central Asia. *Flora* 207: 237-249.

Dombrowski JC. 1971. *Excavations in Ethiopia: Lalibela and Natchabiet Caves, Begemeder Province.* Ph.D. Dissertation, Department of Anthropology, Boston University.

Doorenbos NJ, Fetterman PS, Quimby MW, Turner CE. 1971. Cultivation, extraction and analysis of *Cannabis sativa* L. *Annals of the New York Academy of Sciences* 191: 3-14.

dos Santos NA, Tose LV, da Silva SRC, et al. 2019. Analysis of isomeric cannabinoid standards and Cannabis products by UPLC-ESI-TWIM-MS: a comparison with GC-MS and GC x GC-QMS. *Journal of the Brazilian Chemical Society* 30: 60-70.

Dufresnes C Jan C, Bienert F, Goudet J, Fumagalli L. 2017. Broad-scale genetic diversity of *Cannabis* for forensic applications. *PLoS One* 12(1): e0170522.

Du Mans R. (Schefer C, *ed.*) 1890. *Estat de la Perse en 1660.* E. Leroux, Paris.

Dupin C. 1831. Note sur l'introduction de quelques plantes nouvelles dans l'industrie et l'agriculture. *Journal des Connaissances Usuelles et Pratiques* 14: 19-20.

Duvall CS. 2014. Review of *Cannabis ethnobotany and evolution* by Robert C. Clarke and Mark D. Merlin. *The Geographical Review* 104: 523-526.

Duvall CS. 2015. *Cannabis*. Reaktion Books, Ltd, London.

Eckler CR, Miller FA. 1912. A study of American-grown *Cannabis* in comparison with samples from other sources. *Eighth International Congress of Applied Chemistry* 17: 23-30 (also: *American Journal of Pharmacy* 84: 488-495).

Edery H, Grunfeld Y, Porath G, Ben-Zvi Z, Shani A, Mechoulam R.1972. Structure-activity relationships in the tetrahydrocannabinol series. *Arzneimittel-Forschung* 22: 1995-2003.

Ehleringer JR, Cerling TE. 2001. "Photosynthetic pathways and climate," pp. 267-277 in Schulze ED, *et al., eds. Global biogeochemical cycles in the climate system.* Academic Press, San Diego.

Ehlers J, Gibbard PL. 2007. The extent and chronology of Cenozoic global glaciation. *Quaternary International* 164–165: 6–20.

Eliot J. 1794. Observations on the inhabitants of the Garrow Hills, during a public deputation in the years 1788 and 1789. Asiatick Researches 3: 17-38.

Elliot HM. 1845. *Supplemental glossary of terms used in the North Western Provinces. A-J.* Secundra Orphan Press, Agra.

Elzinga S, Fishchedick J, Podkolinski R, Raber JC. 2015. Cannabinoids and terpenes as chemotaxonomic markers in *Cannabis. Natural Products Chemistry Research* 3:4

Emboden WA. 1977. A taxonomy for *Cannabis. Taxon* 26: 110.

Evans JF. 1894. Report by Surgeon-Captain J. F. Evans, chemical examiner to the Government of Bengal, regarding physiological investigations concerning hemp drugs. *Report of the Indian Hemp Drugs Commission* 3: 206-231.
Ewart AJ. 1894. Vitality and germination of seeds. *Proceedings and Transactions of the Liverpool Biological Society* 8: 207-247.
Fairbairn JW, Liebmann JA. 1974. The cannabinoid content of *Cannabis sativa* L grown in England. *Journal of Pharmacy and Pharmacology* 26: 413-419.
Falck JP (Georgi JG, *ed*). 1786a. *Beyträge zur Topographischen Kenntniß des Russischen Reichs, Zwenter Band.* Akademie der wissenschaften, St. Petersburg.
Falck JP (Georgi JG, *ed*). 1786b. *Beyträge zur Topographischen Kenntniß des Russischen Reichs, Dritter Band.* Akademie der wissenschaften, St. Petersburg.
Fedchenko A. 1875. Путешествие въ Туркестанъ (*Travels in Turkestan*). Gos. izd-vo Geograficheskoi Literatury, Moscow.
Fetterman PS, Turner CE. 1972. Constituents of *Cannabis sativa* L. I: propyl homologs of cannabinoids from an Indian variant. *Journal of Pharmaceutical Sciences* 61: 1476-1477.
Fetterman PS, Seith ES, Waller CW, Guerrero O, Doorenbos NJ, Quimby MW. 1971. Mississippi-grown *Cannabis sativa* L.: preliminary observation on chemical definition of phenotype and variations in tetrahydrocannabinol content versus age, sex, and plant part. *Journal of Pharmaceutical Sciences* 60: 1246-1249.
Field BI, Arndt RR. 1980. Cannabinoid compounds in South African *Cannabis sativa* L. *Journal of Pharmacy and Pharmacology* 32: 21-24.
Fischedick JT. 2017. Identification of terpenoid chemotypes among high (-)-*trans*-$\Delta^9$-tetrahydrocannabinol-producing *Cannabis sativa* L. cultivars. *Cannabis and Cannabinoid Research* 2(1): 34-47.
Fischedick JT, Hazekamp A, Erkelens T, Choi YH, Verpoorte R. 2010. Metabolic fingerprinting of *Cannabis sativa* L., cannabinoids and terpenoids for chemotaxonomic and drug standardization purposes. *Phytochemistry* 71: 2058-2073.
Fischer JB. 1810. "*Cannabis chinensis*," pp. 45 and 131 in *Auf vierzehnjährige Erfahrungen und Beobachtungen gegründete Anweisung zum Anbau ausländischer Getraidarten und einiger Oelgewächse.* Grattenauerschen Buchhandlung, Crailsheim.
Flad R, Li SC, Wu XH, Zhao ZJ. 2010. Early wheat in China: results from new studies at Donghuishan in the Hexi Corridor. *The Holocene* 20: 955-965.
Flores-Sanchez IJ, Verpoorte R. 2008. Secondary metabolism in *Cannabis*. *Phytochemistry Reviews* 7: 615–639.
Flores-Sanchez IJ, Verpoorte R. 2008. PKS activities and biosynthesis of cannabinoids and flavonoids in *Cannabis sativa* L. plants. *Plant and Cell Physiology* 49: 1767-1782.
Forapani S, Carboni A, Paoletti C, Moliterni VMC, Ranalli P, Mandolino G. 2001. Comparison of hemp varieties using random amplified polymorphic DNA markers. *Crop Sciences* 41: 1682-1689.
Forsyth JD. 1874. "Report on the first fair held at Palampur, by J. D. Forsyth, Esq., C.B., Commissioner, Jalandhar Division," pp 5-12 in *Reports on trade routes and fairs on the northern frontiers of India.* Her Majesty's Stationery Office, London.
Forsyth TD. 1875. *Report of a Mission to Yarkund in 1873.* Foreign Department Press, Calcutta.
Fournier G. 1981. Les chimiotypes du chanvre (*Cannabis sativa* L.) Intérêt pour un programme de selection. *Agronomie* 1: 679-688.
Franklin SB, Tolonen M. 2000. Temporally-explicit models of fire and forest. *Plant Ecology* 146: 145-168.
Fristedt R.F. 1870. Om hampan i medicinskt hänseende. *Upsala Läkareförenings Förhandlingar* 5: 499-530.
Fu LG, *ed*. 2000. "*Cannabis* Linn." *Higher plants of China* 4: 26. Qingdao Publishing House, Beijing.
Gaoni Y, Mechoulam R. 1964. Isolation, structure, and partial synthesis of an active constituent of hashish. *J. American Chemical Society* 86: 1646-1647.

Ghose MN, Bhattacharjee SN. 1935. Determination of physiological activity of hemp resin by a polarimetric methods. *Analyst* 60: 313-316.
Gilbert MT, Hansen AJ, Willerslev E, Rudbeck L, Barnes I, Lynnerup N, Cooper A. 2003. Characterization of genetic miscoding lesions caused by postmortem damage. *American Journal of Humun Genetics* 72(1): 48–61.
Gill EW, Paton WDM, Pertwee RG. 1970. Preliminary experiments on the chemistry and pharmacology of *Cannabis. Nature* 228: 134-136.
Gillan R, Cole MD, Linacre A, Thorpe JW, Watson ND. 1995. Comparison of *Cannabis sativa* by random amplification of polymorphic DNA (RAPD) and HPLC of cannabinoids: a preliminary study. *Science and Justice* 35(3): 169-177.
Gilli A. 1963. Beiträge zur Flora Afghanistans III. *Feddes Repertorium* 63: 239-259.
Gilmore S. 2005. Rasta lends its name to a third 'race' of cannabis. *New Scientist.* 187 (Sep 17-Sep 23): 12.
Gilmore S, Peakall R. 2003. Isolation of microsatellite markers in *Cannabis sativa* L. (marijuana). *Molecular Ecology Notes* 3: 105-107.
Gilmore S, Peakall R, Robertson J. 2003. Short tandem repeat (STR) DNA markers are hypervariable and informative in *Cannabis sativa*: implications for forensic investigations. *Forensic Science International* 131: 65-74.
Gilmore S, Peakall R, Robertson J. 2007. Organelle DNA haplotypes reflect crop-use characteristics and geographic origins of *Cannabis sativa. Forensic Science International* 172: 179-190.
Graham SA. 1988. Revision of *Cuphea* section *Heterodon* (Lythraceae). *Systematic Botany Monographs* 20: 1-168.
Grassa CJ, Wegner JP, Dabney C, Poplawski SH, Motley ST, Michael TP, Schwartz CJ, Weiblen GD. 2018. A complete *Cannabis* chromosome assembly and adaptive admixture for elevated cannabidiol (CBD) content. BioRxiv Preprint, https://doi.org/10.1101/458083.
Grassi G, McPartland JM. 2017. "Chemical and morphological phenotypes in breeding of *Cannabis sativa* L.," pp. 137-160 in Chandra S, Lata H, ElSohly MA, eds. *Cannabis sativa: Botany and Biotechnology.* Springer International Publishing, Cham, Switzerland.
Grebenkin AD. 1872. "Таджики" ("Tajiks"), pp. 1-50 in Trotskago VN, *ed. Русский Туркестан: Сборник изданный по поводу политехнической выставки.* Katkov & Kom., Moscow.
Gressel J, *ed.* 2005. *Crop ferality and volunteerism.* CRC Press, Boca Raton FL.
Grierson GA. 1894. The hemp plant in Sanskrit and Hindi literature. *The Indian Antiquary* 23: 260-262.
Grierson GA. 1916. *A dictionary of the Kāshmīrī language.* Asiatic Society of Bengal, Calcutta.
Griffith W. 1847. *Journals of Travels in Assam Burma Bootan Affghanistan and the Neighbouring Countries* Bishop's College Press, Calcutta.
Grlić L. 1968. A combined spectrophotometric differentiation of samples of cannabis. *Bulletin on Narcotics* 20: 25-30.
Grlić L, Andrec A. 1961. The content of acid fractions in cannabis resin of various age and provenance. Experientia 17: 325-326.
Hakki EE, Kayis SA, Pinarkara E, Sag A. 2007. Inter simple sequence repeats separate efficiently hemp from marijuana (*Cannabis sativa* L.). *Electronic Journal of Biotechnology* DOI: 10.2225.
Hamilton F. 1819. *An Account of the Kingdom of Nepal.* Archibald Constable, Edinburgh
Hamm W. 1854. Die culture des hanfs. *Agronomische Zeitung* 9: 97-103 (also *Die Grundzüge der Landwirthschaft, Zweiter Band.* Vieweg & Sohn, Braunschweig).
Hammer K, Gladis T. 2014. Notes on infraspecific nomenclature and classifications of cultivated plants in Compositae, Cruciferae, Cucurbitaceae, Gramineae (with a remark on *Triticum dicoccon* Schrank) and Leguminosae. *Genetic Resources & Crop Evolution* 61: 1455–1467.
Haney A, Bazzaz FA. 1970. "Some ecological implications of the distribution of hemp (*Cannabis sativa* L.) in the United States of America," pgs 39-48 *in* Joyce CRB, Curry SH, *eds. The botany and chemistry of Cannabis.* J & A Churchill, London.

Haney A, Kutscheid BB. 1975. An ecological study of naturalized hemp (*Cannabis sativa* L.) in east-central Illinois. *American Midland Naturalist* 93:1-24.
Hanuš L, Dostálová M. 1994. The effect of soil fertilization on the formation and the amount of cannabinoid substances in *Cannabis sativa* L. in the course of one vegetation period. *Acta Universitatis Palackianae Olomucensis* 138: 11-15.
Hanuš L, Turner CE, ElSohly MA. 1987. Isolation and identification of propylcannabinoids from Soviet variety of hemp grown in Mississippi. *Acta Universitatis Palackianae Olomucensis Facultatis Medicae* 116:25-30.
Hanuš LO, Levy R, La Vega D, Katz L, Roman M, Tomíček P. 2016. The main cannabinoids content in hashish samples seized in Israel and Check Republic. *Israel Journal of Plant Sciences* 63: 182-190. Hassinger JD. Introduction to the mammal survey of the 1965 Street expedition to Afghanistan. *Fieldiana Zoology* 55: 1-81.
Hardwicke T. 1801. Enumeration of plants noticed in the preceeding tour, between Hurdwar and Sirinagur, in the months of April and May 1796. *Asiatick Researches* 6 (Miscellaneous tracts): 264-276.
Hayon JC, Pelt JM, Younos C. 1970. Les formations vegetales de la moyenne vallee du Kaboul et des massifs montagneux du Nuristan (Afghanistan). *Vegetatio* 20: 279-306.
Hazekamp A, Fischedick JT. 2012. *Cannabis*—from cultivar to chemovar. *Drug Testing and Analysis* 4: 660-667.
Hazekamp A, Giroud C, Peltenburg A, Verpoorte R. 2005. Chromatographic and spectroscopic data of cannabinoids from *Cannabis sativa* L. *Journal of Liquid Chromatography & Related Technologies* 28: 2361-2382.
Hazekamp A, Tejkalová K, Papadimitriou S. 2016. *Cannabis*: from cultivar to chemovar II—a metabolomics approach to *Cannabis* classification. *Cannabis and Cannabinoid Research* 1(1):202–215.
Hebert PD, Stoeckle MY, Zemlak TS, Francis CM. 2004. Identification of birds through DNA barcodes. *PLoS Biology* 2(10): e312.
Hedde JCPI. 1848. *Description méthodique des produits divers recueillis dans un voyage en China, 1843-1846.* Théolier Ainé, Saint-Étienne.
Hemphill JK, Turner JC, Mahlberg PG. 1980. Cannabinoid content of individual plant organs from different geographical strains of *Cannabis sativa* L. *Journal of Natural Products* 43: 112-122.
Henderson G, Hume AO. 1873. *Lahore to Yarkand : incidents of the route and natural history of the countries traversed by the expedition of 1870.* L. Reeve & Co., London.
Hennig W. 1966. *Phylogenetic systematics.* University of Illinois Press, Chicago.
Henry P. 2015. Genome-wide analyses reveal clustering in *Cannabis* cultivars: the ancient domestication trilogy of a panacea. *PeerJ PrePrints* https://peerj.com/preprints/1553.pdf
Herodotus (Strassler RB, *ed*., Purvis AL, *trans*). 2007. *The landmark Herodotus: the Histories.* Pantheon Books, NY.
Heuzé G. 1860. *Les Plantes Industrielles, Vol. 2.* Hachette, Paris.
Hey J. 2001. *Genes, categories, and species.* Oxford University Press, Oxford, UK.
Hey J, Pinho C. 2012. Population genetics and objectivity in species diagnosis. *Evolution* 66: 1413-1429.
Hijwegen T. 1979. Fungi as plant taxonomists. *Symbolae Botanicae Upsalienses 22*: 146-165.
Hillig KW. 2004a. A multivariate analysis of allozyme variation in 93 *Cannabis* accessions from the VIR germplasm collection. *Journal of Industrial Hemp* 9(2): 5-22.
Hillig KW. 2004b. A chemotaxonomic analysis of terpenoid variation in *Cannabis*. *Biochemical Systematics and Ecology* 32: 875-891.
Hillig KW. 2005a. Genetic evidence for speciation in *Cannabis* (Cannabaceae). *Genetic Resources and Crop Evolution* 52: 161-180.
Hillig KH. 2005b. *A systematic investigation of Cannabis*. Doctoral Dissertation, Department of Biology, Indiana University, Bloomington, IN.

Hillig KW, Mahlberg PG. 2004. A chemotaxonomic analysis of cannabinoid variation in *Cannabis* (Cannabaceae). *American Journal of Botany* 91(6): 966-975.
Hoffmann W. 1961. "Hanf, *Cannabis sativa* L.," pp. 204-263 in Kappert H, Rudorf W, Roemer T, *eds. Handbuch der Pflanzenzüchtung, 2nd Edition, Vol.* 5. Paul Parey, Berlin.
Hoffmann-La Roche & Co. 1915. Verfahren zur Trennung der pharmakologisch wirksamen Bestandteile der *Cannabis indica*. *Chemisches Zentralblatt* 86(2): 508-509.
Holley JH, Hadley KW, Turner CE. 1975. Constituents of *Cannabis sativa* L. XI: cannabidiol and cannabichromene in smaples of known geographical origin. *Journal of Pharmaceutical Sciences* 64: 892-895.
Holmgren NH. 1994. Redefinition of *Dodecotheon dentatum* (Primulaceae) and rationale for use of varietal rank. *Brittonia* 48: 87-94.
Hood LVS, Barry GT. 1978. Headspace volatiles of marihuana and hashish: gas chromatographic analysis of samples of different geographic origin. *Journal of Chromatography* 166: 499-506.
Hood LVS, Dames ME, Barry GT. 1973. Headspace volatiles of marijuana. *Nature* 242:402-3.
Hooker JD. 1854. *Himalayan journals; or, notes of a naturalist, Vol. I.* John Murray, London.
Hooker JD. 1890. *The Flora of British India, Vol. 5.* L. Reeve & Co., London.
Hooper D. 1908. Charas of Indian hemp. *Year-Book of Pharmacy* 1908: 435-444.
Houghton EM. 1897. How can we increase the therapeutic reliability of medicinal agents? *Journal of the American Medical Association* 28: 634-636.
Howard C, Gilmore S, Robertson J, Peakall R. 2008. Developmental validation of a *Cannabis sativa* STR multiplex system for forensic analysis. *Journal Forensic Science* 53: 1061-1067.
Hsieh HM, Hou RJ, Tsai LC, Wei CS, Liu SW, Huang LH, Kuo YC, Linacre A, Lee JC. 2003. A highly polymorphic STR locus in *Cannabis sativa*. *Forensic Science International* 131(1): 53-58.
Hu XL, Guo HY, Liu XY, Hu GW, Xu YP, Guo MB, Yang M. 2012. 云南工大麻品种在黑江大麻安岭地区的适性研究 (Study on adaptability of Yunnan industrial hemp varieties in Daxinganling region of Heilongjiang province). *Southwest China Journal of Agricultural Sciences* 25: 838-841.
Huang YJ, Jia LB, Wang Q, Mosbrugger V, Utescher T, Su T, Zhou ZK. 2016. Cenozoic plant diversity of Yunnan: a review. *Plant Diversity* 38: 271-282.
Huddleston H. 1841. Report on hemp cultivation, etc. in British Gurhwal. *Transactions of the Agricultural & Horticultural Society of India* 8: 260-278.
Humboldt A. von. 1811. *Essai politique sur le royaume de la Nouvelle-Espagne, Tome troisième.* F. Schoell, Paris.
Ibn-al-Baitār (Leclerc L, *trans*). 1883. "Traité des simples par Ibn El-Beïthar. Tome Troisième," in *Notices et extraits des manuscrits de la Bibliothèque Nationale.* Imprimerie Nationale, Paris.
Idilbi MM, Huvenne JP, Fleury G, Tran Van P, Muller PH, Moschetto Y. 1985. Application de la chromatographie en phase gazeuse couplée à la spectrométrie infra-rouge par transformée de Fournier. Bulletin de la Société de Pharmacie de Lille 41(4): 27-32.
Indian Hemp Drugs Commission. 1894. *Report of the Indian Hemp Drugs Commission, 1893-94.* Government Central Printing Office, Simla.
Itier J. 1846. Notes sur quelques produits de l'industrie chinoise récueillis par M. Jules Itier, inspecteur principal des douanes. *Bulletin de la Société d'Encouragement pour l'Industrie Nationale* 45: 238-240.
Jacquemont V. 1861. *Correspondance de Victor Jacquemont avec sa famille et plusieurs de ses amis pendant son voyage dans l'Inde, Tome second.* Garnier Frères, Paris.
Jagadish V, Robertson J. Gibbs A. 1996. RAPD analysis distinguishes *Cannabis sativa* samples from different sources. *Forensic Science International* 79: 113-121.
Janischevsky DE. 1924. Форма конопли на сорных местах в Юго-Восточной России (A form of cannabis in wild areas of south-eastern Russia). Ученые записки Саратовского государственного университета имени Н. Г. Чернышевского 2(2): 3-17.
Janischevsky DE. 1925. Энтомохория у *Cannabis ruderalis* Janisch. (*Entomohoriya in Cannabis ruderalis Janisch.*). Saratov Society of Naturalists, Saratov.

Jansen RK. 1985. The systematics of *Acmella* (Asteraceae-Heliantheae). *Systematic Botany Monographs* 8: 1-115.

Jenkins RW, Patterson DA. 1973. The relationship between chemical composition and geographical origin of *Cannabis. Foresnsic Science* 2: 59-66.

Jia X, Dong GH, Li H, Brunson K, Chen FH, Ma MM, Wang H, An CB, Zhang K. 2012. The development of agriculture and its impact on cultural expansion during the late Neolithic in the Western Loess Plateau, China. *The Holocene* 23: 85-92.

Jia X, Sun YG, Wang L, Sun WF, Zhao ZJ, Lee HF, Huang WB, Wu SG, Lu HY. 2016. The transition of human subsistence strategies in relation to climate change during the Bronze Age in the West Liao River Basin, Northeast China. *The Holocene* 26: 781-789.

Jiang HE, Li X, Zhao YX, Ferguson DK, Hueber F, Bera S, Wang YF, Zhao LC, Liu CJ, Li CS. 2006. A new insight into *Cannabis sativa* (Cannabaceae) utilization from 2500-year-old Yanghai Tombs, Xinjiang, China. *J Ethnopharmacology* 108: 414-422.

Jiang HE, Li X, Ferguson DK, Wang YF, Liu CJ, Li CS. 2007. The discovery of *Capparis spinosa* L. (Capparidaceae) in the Yanghai Tombs (2800 years b.p.), NW China, and its medicinal implications. *Journal of Ethnopharmacology* 113: 409-420.

Jiang HE, Wang L, Merlin MD, Clarke RC, Pan Y, Zhang Y, Xiao GQ, Ding XL. 2016. Ancient *Cannabis* burial shroud in a Central Eurasian cemetery. *Economic Botany* 70: 213-221.

Jikomes N, Zoorob M. 2018. The cannabinoid content of legal cannabis in Washington State varies systematically across testing facilities and popular consumer products. *Scientific Reports* 8: 4519.

Johnson WH. 1867. Report on his journey to Ilchi, the capital of Khotan, in Chinese Tartary. *Journal of the Royal Geographical Society of London* 37: 1-47.

Jomard M. 1846. Rapport sur le concours au prix d'Orléans pour l'importation la plus utile à agriculture, à l'industrie ou à l'humanité. *Bulletin de la Société de Géographie* (Series 3) 5: 300-311.

Kalhana (Stein MA, *trans*). 1989. *Kalhana's Rajatarangini: A Chronicle of the Kings of Kashmir, 3 vols.* Motilal Banarsidass, Delhi.

Kallawicha K. 2008. Survey of *Cannabis sativa* L. "fiber type" cultivated in Thailand using gas chromatography-mass spectrometry data. *Srinakharinwirot Science Journal* 24(1): 154-164.

Kasahara Y. 1987. "鳥浜貝塚(第 7 次発掘)における種子集中層から出土種実の同定—アサ,クマヤナギ,ヒルムシロ 類,その他—," pp. 1-6 in 鳥浜貝塚 1985 年度調査概報・研究の成果—縄文前期を主とする低湿地遺跡の調 査 6—. Wakasa Historical Folk Museum, Fukui Prefecture.

Kayis SA, Hakki EE, Pinarkara E. 2010. Comparison of effectiveness of ISSR and RAPD markers in genetic characterization of seized marijuana (*Cannabis sativa* L.) in Turkey. *African Journal of Agricultural Research* 5: 2925-2933.

Kennedy PB. 1915. Common hemp as a medicinal plant. *Annual Report of the Agricultural Experiment Station, University of Nevada* 21: 44-47.

Kerkoff AJ, Enquist BJ, Elser JJ, Fagan WF. 2005. Plant allometry, stoichiometry and the temperature-dependence of primary productivity. *Global Ecology and Biogeography* 14: 585–598.

Kirke H. 1842. On Indian hemp and the inducements for speculating therein. *Journal of the Agri-Horticultural Society of India* 1: 41-48.

Kirkpatrick WK. 1811. *An Account of the Kingdom of Nepaul: being the substance of observations made during a mission to that country, in the year 1793.* W. Miller, London.

Knight G, Hansen S, Conner M, Poulsen H, McGovern C, Stacy J. 2010. The result of an experimental indoor hyroponic *Cannabis* growing study using the 'screen of green' (ScOG) method: yield, tetrahyrocannabinol (THC) and DNA analysis. *Forensic Science International* 202: 36-44.

Kobayashi M, Momohara A, Okitsu S, Yanagisawa S, Okamoto T. 2008. Fossil hemp fruits in the earliest Jomon period from the Okinoshima site, Chiba Prefecture. *Shokuseishi kenkyū (Japanese Journal of Historical Botany)* 16(1): 11-18.

Koch K(C). 1854. Cannabis chinensis. *Annales des Sciences Naturelles Botanique* (Series 4) 1: 352.

Køie M, *ed*. 1954. *Symbolae Afghanicae: enumeration and descriptions of the plants collected by L. Edelberg and M. Køie on the 3rd Danish Expedition to Central Asia and by W.Koelz, H. F. Neubauer, O. H. Volk, and others in Afghanistan.* I kommission hos Munksgaard, København.

Kojoma M, Iida O, Makino Y, Sekita S, Satake M. 2002. DNA fingerprinting of *Cannabis sativa* using inter-simple sequence repeat (ISSR) amplification. *Planta Medica* 68(1): 60-63.

Kojoma M, Seki H, Yoshida S, Muranaka T. 2006. DNA polymorphisms in the tetrahydrocannabinolic acid (THCA) synthase gene in "drug-type" and "fiber-type" *Cannabis sativa* L. *Forensic Science International* 159(2-3):132-40.

Korotkova TI. 1978. Н.И. Вавилов в Саратове, 1917-1921: документальные очерки (*N.I. Vavilov in Saratov, 1917-1921: feature stories*). Privolzhskoye Book Publishing House, Saratov.

Krassnoff A. 1887. Список растений, собранных в восточном Тянь-Шане летом 1886 года (*Enumeratio plantarum anno 1886 in Tain Schan orientali lectarum*). V. Demakova, St. Petersburgh.

Kriese U. 2007. *Genotypendifferenzierung und Erstellung von Kreuzungspopulationen bei Hanf* (*Cannabis sativa* L.). Dissertation, Martin-Luther-Universität, Halle-Wittenberg.

Kubešova M, Moravcová L, Suda J, Jarošík V, Pyšek P. 2010. Naturalized plants have smaller genomes than their non-invading relatives: a flow cytometric analysis of the Czech alien flora. *Preslia* 82: 81-96.

Kudryashev SN. 1932. Дикорастущие эфирно-масличные растения центральной части Гиссарского хребта (Wild-growing ethereal oil plants in the central part of the Gissar Range). *Труды Среднеазиатской опытной станции эфирно-масличных растений* 1: 1-112.

Küppers FJEM, Bercht CAL, Salemink CA, Lousberg RJJC, Terlouw JK, Heerma W. 1973. Cannabis—XV: pyrolysis of cannabidiol. Structure elucidation of four pyrolytic products. *Tetrahedron* 31: 1513-1516.

Kushelevskiĭ VI. 1891. Материалы для медицинской географии и санитарного описания Ферганской области, том 2 [*Materials for medical geography and the sanitary description of the Fergana area, vol. 2*]. Tipographia Ferganskoi oblasti, New Margelan.

Lamarck JB. 1799. *Tableau encyclopédique et methodique des trois règnes de la nature: Botanique, Planches 801-900.* Panckoucke, Paris.

Lamarck JB. 1785. *Encyclopédie Méthodique, Botanique, Tome premier,* part 2, pp. 345-752. Panckoucke, Paris.

Lamarck JB. 1809. *Philosophie Zoologique,* 2 vols. Dentu, Paris.

Lamarck JB, Poiret JLM. 1823. *Tableau encyclopédique et methodique des trois règnes de la nature: Botanique, Tome troisième.* Agasse, Paris.

Langlie BS, Mueller NG, Spengler RN, Fritz GL. 2014. Agricultural origins from the ground up: archaeological approaches to plant domestication. *American Journal of Botany* 101: 1601-1617.

Lansdell H. 1885. *Russian Central Asia, vol. 1*. Houghton, Mifflin & Co., Boston.

Latta RP, Eaton BJ. 1975. Seasonal fluctuatons in cannabinoid content of Kansas marijuana. *Economic Botany* 29: 153-163.

Lavelle PB. 2012. *"Profits of nature": the political ecology of agrarian expansion in a nineteenth-century Chinese borderland.* Doctoral thesis, Cornell University.

Laverty KU, Stout JM, Sullivan MJ, Shah H, Gill N, *et al.* (six additional authors). 2019. A physical and genetic map of *Cannabis sativa* identifies extensive rearrangements at the *THC/CBD acid synthase* loci. *Genomic Research* 29: 146-156.

Lawrence WR. 1895. *The valley of Kashmir.* H. Frowde, London.

Lawi-Berger C, Miège MN, Kapétanidis I, Miège J. 1982. Contribution a l'etude chimiotaxonomique de *Cannabis sativa* L. *Comptes Rendus de l'Académie des Sciences Paris* 295(Série III): 397-402.

L'dov N. 1933. Культура лубяных растений в Советской Азии—За индустр (Culture of bast plants in Soviet Asia—for industry). *За индустриализацию Советского Востока* 1933(2): 103-128.

Leghissa A, Hildenbrand ZL, Schug KA. 2018. A review of methods for the chemical characterization of cannabis natural products. *Journal of Separation Science* 41: 398-415.

Lehmann T, Brenneisen R. 1992. A new chromatographic method for the isolation of (–)-$\Delta^9$-(*trans*)-tetrahydrocannabinolic acid A. *Phytochemical Analysis* 3: 88-90.

Lehmann T, Brenneisen R. 1995. High performance liquid chromatographic profiling of cannabis products. *Journal of Liquid Chromatography* 18: 689-700.

Lepechin II (Hare CH, *trans*). 1774. *Tagebuch der Reise durch verschiedene Provinzen des Russischen Reiches, Erster Theil.* Richterischen Buchhandlung, Altenburg.

Levi SC, Sela R, *eds*. 2010. *Islamic Central Asia: an anthology of historical sources.* Indiana University Press, Bloomington, IN.

Lewis MA, Russo EB, Smith KM. 2018. Pharmacological foundations of *Cannabis* chemovars. *Planta Medica* 84: 225-233

Lichtman AH, Wiley JL, LaVecchia KL, Neviaser ST, Arthur DB, Wilson DM, Martin BR. 1998. Effects of SR141716A after acute or chornic cannabinoid administration in dogs. *European Journal of Pharmacology* 357: 139-148.

Linnaeus C. 1737. *Hortus Cliffortianus.* Publisher not identified, Amsterdam.

Linnaeus C. 1751. *Philosophia Botanica.* Kiesewetter, Stockholm.

Linnaeus C. 1753. *Species Plantarum, Tomus II*. Laurentii Salvii, Stockholm.

Little PJ, Compton DR, Johnson MR, Melvin LS, Martin BR. 1988. Pharmacology and stereoselectivity of structurally novel cannabinoids in mice. *Journal of Pharmacology and Experimental Therapeutics* 247: 1046–1051.

Liu TC, Shang YF. 1992. 新疆大麻植物中Δ9-四氢大麻酚, 大麻二酚, 大麻酚的气相色谱测定 (Determination of $\Delta^9$-tetrahydrocannabinol, cannabidiol and cannabinol in Xinjiang *Cannabis* plants by gas chromatography). 中草药 (*Chinese Traditional and Herbal Drugs*) 23(9):463-464.

Liu JH, Lin WF, Fitzgerald MP, Saxena SC, Shieh YN. 1979. Possible characterization of samples of *Cannabis sativa* L. by their carbon isotopic distributions. *Journal of Forensic Sciences* 24(4):814–6.

Liu XY, Guo MB, Zhang QY, Xu YP, Guo LF, Yang M. 2010. 大麻品种遗传多样性的 AFLP 分析. *Journal of Plant Genetic Resources* 13: 555-561.

Loewe S. 1939. Principle of "bioassay by approximation" and its application to the assay of marihuana (dog) and laxatives (monkey). *Journal of Pharmacology and Experimental Therapeutics* 66: 23-24.

Loewe S. 1944. "Studies on the pharmacology of marihuana," pp. 149-212 in LaGuardia Committee, *eds. The Marihuana Problem in the City of New York*. Jaques Cattell Press, Lancaster, PA.

Loewe S. 1946. Studies on the pharmacology and acute toxicity of compounds with marihuana activity. *Journal of Pharmacology and Experimental Therapeutics* 88: 154-161.

Loewe S. 1950. Cannabiswirkstoffe und Pharmakologie der Cannabinole. *Naunyn-Schmiedebergs Archiv für experimentelle Pathologie und Pharmakologie* 211(2): 175-193.

Loewenthal J. 1926. *Wirtschaftsgecshichtliche Parerga.* Carl Winter, Heidelberg.

Lowry DB, Gould BA. 2016. Speciation continnum. *Encyclopedia of Envolutionary Biology* 4: 159-165.

Lynch RC, Vergara D, Tittes S, White K, Schwartz CJ, Gibbs MJ, Ruthenburg TC, deCesare K, Land DP, Kane NC. 2016. Genomic and chemical diversity in *Cannabis. Critical Reviews in Plant Sciences* 35: 349-363.

Ma QY, Jiang HE, Zhao YX. 2011. 新疆洋海古代大麻叶的大麻酚分析. *Natural Products Research and Development* 23: 82-84.

Macgowan DJ. 1850. Notice regarding the plants yielding the fibre from which the grass-cloth of China is manufactured. *American Journal of Science and Arts* (Series 2) 10: 207-213.

Madden E. 1848. The Turaee and outer mountains of Kumaoon. *Journal of the Asiatic Society of Bengal* 17: 349-450.

Magyari EK, Chapman JC, Gaydarska B, Marinova E, Deli T, Huntley JP, Allen JRM, Huntley B. 2008. The 'oriental' component of the Balkan flora: evidence of presence on the Thracian Plain during the Weichselian late-glacial. Journal of Biogeography 35: 865–883.

Malingré TM, Hendriks H, Batterman S, Bos R, Visser J. 1975. The essential oil of *Cannabis sativa. Planta Medica* 28: 56-61.

Mal'tsev AI. 1939. Атлас важнейших видов сорных растений СССР, Том II (*Atlas of major species of weed plants of the USSR, Vol. II*). Sel'khozgiz, Moscow.
Manafzadeh S, Salvo G, Conti E. 2013. A tale of migrations from east to west: the Irano-Turanian floristic region as a source of Mediterranean xerophytes. *Journal of Biogeography* 41: 366-379.
Mandolino G, Faeti V, Carboni A, Ranalli P. 1997. A 400 bp RAPD marker tightly linked to the male phenotype in dioecious hemp. *Proceedings of the 2$^{nd}$ International Symposium "Bioresource hemp,"* Frankfurt am Main, p. 195-196.
Mandolino G, Carboni A, Bagatta M, Cruicitti P, Ranalli P. 2002. Variabilità e mappatura di marcatori molecolari RAPID in *Cannabis sativa* L. *Agroindustria* 1(1): 3-7.
Mann EM. 1911. *Nineteenth annual report from the analytical laboratories of Southall Bros. & Barclay.* Private press of Southall Bros. & Barclay, Birmingham UK.
Mansouri H, Asrar Z, Amarowicz R. 2011. The response of terpenoids to exogenous gibberellic acid in *Cannabis sativa* at flowering stage. *Acta Physiologiae Plantarum* 33: 1085-1091
Mansouri H, Asrar Z. 2012. Effects of abscisic acid on content and biosynthesis of terpenoids in *Cannabis sativa* at vegetative stage. *Biologia Plantarum* 56: 153-156.
Marshall CR, Winger JH. 1911. Report on the standardization of preparations of Indian hemp. *British Medical Journal* 1(2629): 1171.
Marshman JA, Popham RE, Yawney CD. 1976. A note on the cannabinoid content of Jamaican ganja. *Bulletin on Narcotics* 28: 63-68.
Martin L, Morison Smith D, Farmilo CG. 1961. Essential oil from fresh *Cannabis sativa* and its use in identification. *Nature* 191: 774-776.
Martius TWC. 1832. *Grundriss der Pharmakognosie des Pflanzenreiches.* Palm & Enke, Erlangen.
Martone G, Della Casa E. 1990. Analysis of the ageing processes in hashish samples from different geographic origin. *Forensic Science International* 47: 147-155.
Martynov DE, Martynova YA. 2015. Дневники Н. Ф. Катанова как источник по социально-экономической обстановке в Синьцзяне 90-х годов XIX в (N.F. Katanov's diaries as a source on the socioeconomic situation in Xinjiang during the 1890s). *Ученые записки Казанского университета* 157: 262-271.
Masoud AN, Doorenbos NJ, Quimby MA. 1973. Mississippi-grown *Cannabis sativa* L. IV: effects of gibberellic acid and indoleacetic acid. *Journal of Pharmaceutical Sciences* 62: 316-318.
Masson C. 1844. *Narrative of Various Journeys in Balochistan, Afghanistan, the Panjab, & Kalat, 3 vols.* Richard Bentley, London.
Mayev NA. 1887. туркестанская Выставка 1886. *Вѣстник финансов, промышленности и торговли* 1887(2): 454-459.
Mayr E. 1942. *Systematics and the Origin of Species*. Columbia University Press, NY.
Mazumi M. 2006. The Zhufoyaojijing at the Lüshun Museum. *International Dunhuang Project Newsletter* No. 28. Available at: http://idp.afc.ryukoku.ac.jp/archives/news28/idpnews_28.a4d
McAllister HA. 1999. "The importance of living collections for taxonomy," pp. 3-10 in Andrews S, Leslie A, Alexander C*, eds. Taxonomy of cultivated plants: Proceedings of the Third International Symposium.* Kew, London.
McEno J, McPartland J, Doctor B, Canapasemi G. 1991. *Cannabis Ecology: a Compendium of Diseases and Pests.* AMRITA Press, Middlebury, VT. 254 pp.
McKernan KJ, Helbert Y, Tadigotla V, McLaughlin S, Spangler J, Zhang L, Smith D. 2016. Single molecule sequencing of THCA synthase reveals copy number variation in modern drug-type *Cannabis sativa* L. *BioRxiv* http://dx.doi.org/10.1101/028654.
McKernan KJ, Helbert Y, Kane LT, Ebling H, Zhang L, *et al.* (9 additional authors). 2018. Cryptocurrencies and zero mode wave guides: an unclouded path to a more contiguous *Cannabis sativa L*. genome assembly. *OSF Preprint* DOI 10.17605/OSF.IO/N98GP
McPartland JM. 1983. *Phomopsis ganjae* sp. nov. on *Cannabis sativa. Mycotaxon* 18:527-530.
McPartland JM. 1984. Pathogenicity of *Phomopsis ganjae* on *Cannabis sativa* and the fungistatic effect of cannabinoids produced by the host. *Mycopathologia* 87:149-153.

McPartland JM. 1992. C.H. Persoon: a phanerogamic footnote. *Mycotaxon* 45: 257-258.
McPartland JM. 2017. "*Cannabis sativa* and *Cannabis indica* versus "Sativa" and "Indica,"" pp. 101-121 in Chandra S, Lata H, ElSohly MA, eds. *Cannabis sativa: Botany and Biotechnology.* Springer International Publishing, Cham, Switzerland.
McPartland JM. 2018. *Cannabis* systematics at the levels of family, genus, and species. *Cannabis and Cannabinoid Research* 3(1): 203-212.
McPartland JM, Cubeta MA. 1997. New species, combinations, host associations and location records of fungi associated with hemp (*Cannabis sativa*). *Mycological Research* 101:853-857.
McPartland JM, Guy GW. 2010. THC synthase in *Cannabis* has undergone accelerated evolution and positive selection pressure. *Proceedings of the 20th Annual Symposium on the Cannabinoids.* International Cannabinoid Research Society, Research Triangle Park, NC. p. 43.
McPartland JM, Guy GW. 2017. Models of Cannabis taxonomy: a systematic review. *The Botanical Review* 83: 327-381.
McPartland JM, Hughes S. 1994. *Cannabis* pathogens VII: a new species, *Schiffnerula cannabis. Mycologia* 86: 867-869.
McPartland JM, Naraine SG. 2018. Experimental endozoochory of *Cannabis sativa* achenes. *Medical Cannabis and Cannabinoids* 1(2): 96-103.
McPartland JM, Pruitt PL. 1999. Side effects of pharmaceuticals not elicited by comparable herbal medicines: the case of tetrahydrocannabinol and marijuana. *Alternative Therapies Health and Medicine* 5: 57-62.
McPartland JM, Clarke RC, Watson DP. 2000. *Hemp diseases and pests - management and biological control.* CABI Publishing, Wallingford, UK.
McPartland JM, MacDonald C, Young M, Grant PS, Furkert DP, Glass M. 2017. Affinity and efficacy studies of tetrahydrocannabinolic acid A at cannabinoid receptor types one and two. *Cannabis and Cannabinoid Medicine* 2(1): 87-95.
McPartland JM, Guy GW, Hegman W. 2018. *Cannabis* is indigenous to Europe and cultivation began during the Copper or Bronze age: a probabilistic synthesis of fossil pollen studies. *Vegetation History and Archaeobotany* 27: 635-648.
McPartland JM, Hegman W, Long TW. 2019. *Cannabis* in Asia: its center of origin and early cultivation, based on a synthesis of subfossil pollen and archaeobotanical studies. *Vegetation History and Archaeobotany, Springer epub ahead of print.*
Mechoulam R, Ben-Zvi Z, Yagnitinsky B, Shani A. 1969. A new tetrahydrocannabinolic acid. *Tetrahedron Letters* 10: 2339-2341.
Medicinal Genomics Corporation. 2011. The cannabis sativa genome. Available at: https://aws.amazon.com/datasets/the-cannabis-sativa-genome/
Medicinal Genomics Corporation. 2015. KannaPedia. The distributed consensus on cannabis genetics. Available at: www.medicinalgenomics.com/kannapedia/
Medvedev Z. 1969. *The rise and fall of T.D. Lysenko.* Columbia University Press, NY.
Mendoza MA, Mills DK, Lata H, Chandra S, El Sohly MA, Almirall JR. 2008. Genetic individualization of *Cannabis sativa* by a short tandem repeat multiplex system. *Analytical and Bioanalytical Chemistry* 393: 719-726.
Merkus FWHM. 1971. Cannabivarin and tetrahydrocannabivarin, two new constituents of hashish. *Nature* 232: 579-580.
Meulenbeld GJ. 1989. The search for clues to the chronology of Sanskrit medical texts, as illustrated by the history of bhangá. *Studien zur Indologie und Iranistik* 15: 59-70.
Meulenbeld GJ. 2002. *A History of Indian Medical Literature, Vol. 3.* Forsten, Groningen.
Middendorf AT. 1882. Очерки Ферганской долины [*Essays on the Fergana Valley*]. Tipografiya Imperial akademii nauk, St. Petersberg.Ostroumov NP. 1896. *сарты. этнографическіе материалы* (*Sarts. Ethnographic materials*). Kamenskie, Tashkent.

Miller Coyle H, Shutler G, Abrams S, Hanniman J, Neylon S, Ladd C, Palmbach T, Lee HC. 2003. A simple DNA extraction method for marijuana sample used in amplified fragment length polymorphism (AFLP) analysis. *Journal of Forensic Science* 48: 343-347.

Mirza Muhammad Haidar (Ross ED, *trans*, Elias N, *ed*). 1898. *A history of the Moghuls of Central Asia: being the Tarikh-i-Rashidi of Mirza Muhammad Haidar, Dughlát.* Sampson Low, Marston & Co., London.

Mobarak Z, Bieniek D, Korte F. 1974. Studies on non-cannabinoids of hashish II. *Chemosphere* 6: 265-270.

Mobarak Z, Bieniek D, Korte F. 1978. Some chromatographic aspects of hashish analysis II. *Forensic Science* 11: 189-193.

Molina-Montenegro MA, Naya DE. 2012. Latitudinal patterns in phenotypic plasticity and fitness-related traits: assessing the climatic variability hypothesis (CVH) with an invasive plant species. *PLoS One* 7(9): e47620.

Moravcová L, Pyšek P, Jarošik V, Havlíčová V, Zákravský P. 2010. Reproductive characteristics of neophytes in the Czech Republic: traits of invasive and non-invasive species. *Preslia* 82: 365-390.

Moreau JJ. 1845. *Du Hachisch et de l'aliénation mentale.* Bourgogne et Martinet, Paris.

Mosbrugger V, Utescher T. 1997. The co-existence approach—a method for quantitative reconstructions of Tertiary terrestrial palaeoclimate data using plant fossils. *Palaeogeography, Palaeoclimatology, Palaeoecology* 134: 61-86.

Mott L. 1986. “A maconha na história do Brasil,” pp. 117, 132-133 in Henman A, Pessoa O, *eds. Diamba sarabamba: coletânea de textos brasileiros sobre a maconha.* Ground, São Paulo.

Mukhopadhyay BK, Subramanian KS, Dunnicliff HB. 1943. A colorimetric method for estimating the narcotic power of hemp drugs. *Analyst* 804: 70-74.

Muminjanov H. 2008. State of plant genetic resources for food and agriculture (PFRFA) in the Republic of Tajikistan. Dushanbe.

Nakamura GR. 1969. Forensic aspects of cystolith hairs of *Cannabis* and other plants. *Journal of the Association of Official Analytical Chemists* 52(1): 5-16.

Nartov AA (*ed*). 1767. Не опвѣдывалиль сѣяпь привезенное изъ другихъ провинціи сѣмя льняное и конопляное? Продолжение трудовъ Вольнаго экономическаго общества, къ поощрению в России земледелия и домостроительства 7: 155.

Naudin C. 1850. Observations sur un certain nombre de plantes nouvelles ou peu connues. *Revue horticole* (Serie 3) 4:114-120.

Naz S, Hanif MA, Ansari TM, Al-Sabahi JN. 2017. A comparative study on hemp (*Cannabis sativa*) essential oil extraction using traditional and advanced techniques. *Spectroscopy and Spectral Analysis* 37: 306-311.

Nekrasova VL. “Семейство 18. Moraceae,” pp. 43-48 in Keller BA, Lyubimenno VI, Mal'tsev AI, Fedtschenko BA, Schischkin BC, Roshevitz RJ, Kamensky KV, *eds*. 1934. Сорные растения СССР, Том II (*Weed plants of the USSR, Vol. II*). USSR Academy of Sciences, Leningrad.

Nikitin E. 2014. Что даст нам легализация марихуаны и как это сделать? (*What will legalize marijuana give us and how to do it?*). Available at: www.zakon.kz/4618509-chto-dast-nam-legalizacija-marikhuany-i.html

Nüsser M, Dickoré WB. 2002. A tangle in the triangle: vegetation map of the eastern Hindukush (Chitral, northern Pakistan). *Erdkunde* 56: 37-59.

Oh H, Seo B, Lee S, Ahn DH, Jo E, Park JK, Min GS. 2016. Two complete chloroplast genome sequences of *Cannabis sativa* varieties. *Mitochondrial DNA Part A* 27: 2835-2837.

Ohlsson A, Abou-Chaar CI, Agurell S, Nilsson IM, Olofsson K, Sandberg F. 1971. Cannabinoid constituents of male and female *Cannabis sativa. Bulletin on Narcotics* 23(1): 29-32.

Omar J, Olivares M, Alzaga M, Etxebarria N. 2013. Optimisation and characterisation of marihuana extracts obtained by supercritical fluid extraction and focused ultrasound extraction and retention time locking GC-MS. *Journal of Separation Science* 36: 1397-1401.

Onofri C, de Meijer EPM, Mandolino G. 2015. Sequence heterogeneity of cannabidiolic- and tetrahydrocannabinolic acid-synthase in *Cannabis sativa* L. and its relationship with chemical phenotype. *Phytochemistry* 116: 57-68.

Orser C, Johnson S, Speck M, Hilyard A, Afia I. 2018. Terpenoid chemoprofiles distinguish drug-type *Cannabis sativa* L. cultivars in Nevada. *Natural Products Chemistry & Research* 6(1): 1-7.

O'Shaughnessy WB. 1839 (or 1838-1840). On the preparations of the Indian hemp, or gunjah *(Cannabis indica)*; their effects on the animal system in health, and their utility in the treatment of tetanus and other convulsive diseases. *Transactions of the Medical and Physical Society of Bengal* 1838-1840: 71-102, 421-61.

Pabst G, *ed.* 1887. "*Cannabis sativa* L.," p. 13 in *Köhler's Medizinal-Pflanzen Band 1.* Eugen Köhler, Gera-Untermhaus.

Pacifico D, Miselli F, Micheler M, Carboni A, Moschella A, Mandolino G. 2008. Time course of cannabinoid accumulation and chemotype development during the growth of *Cannabis sativa* L. *Euphytica* 160: 231–240 .

Pallas PS. 1793. *Voyages de M. P. S. Pallas, en différentes provinces de l'empire de Russie. Tome Cinquième.* Maradan, Paris.

Pallas PS. 1794. *Voyages du Professeur Pallas, dans plusieurs provinces de l'empire de Russie. Tome Quatrième.* Maradan, Paris.

Pantusov NN. 1881. *Свѣдѣнія о Кульджинском районѣ: за 1871-1877 годы* (*Knowledge about Kuldzhinsky district: for 1871-1877*). University tipographii, Kazan.

Paris M, Ghirlanda C, Chaigneau M, Ciry L. 1973. L'acide $\Delta^1$-tétrahydrocannabivarolique, nouveau constituant du *Cannabis saliva* L. *Comptes Rendus de l'Académie des Sciences* Série C 276: 205-207.

Patten MA, Unitt P. 2002. Diagnosability versus mean differences of sage sparrow subspecies. *Auk* 119: 26-35.

Pennisi E. 2013. Old dogs teach a new lesson about canine origins. *Science* 342: 785-786.

Personne J (Robiquet E, *ed.*). 1857. Rapport sur le concours relatif à l'analyse du chanvre présente au nom de la Société de Pharmacie, *Journal de Pharmacie et de Chimie* (Serie 3) 31: 46-51.

Persoon CH. 1807. *Synopsis plantarum seu enchiridium botanicum, Pars secunda*. Treuttel & Würtz, Parisiis Lutetiorum, et J. G. Cottam, Tubingae.

Petzholdt A. 1874. *Turkestan: Auf Grundlage einer im Jahre 1871 unternommenen Bereisung des Landes.* Schliche, Leipzig.

Pickersgill B, Chacón Sánchez MI, Debouk DG. 2001. "Multiple domestications and their taxonomic consequences: the example of *Phaseolus vulgaris*," pp. 71-83 in Knüpffer H, Ochsmann J, *eds. Rudolf Mansfeld and plant genetic resources*. ZADI, Bonn, Germany.

Pierson D. 2016. Why chocolope? To sell marijuana, you need a clever name. *Los Angeles Times*, Feb. 9, 2016.

Pietri JR. 2009. Who is Dr. Frankenbeanstein. *Treating Yourself Magazine* 17: 23-29.

Piluzza G, Delogu G, Cabras A, Marceddu S, Bullitta S. 2013. Differentiation between fiber and drug types of hemp (*Cannabis sativa* L.) from a collection of wild and domesticated accessions. *Genetic Resources and Crop Evolution* 60: 2331-2342.

Pitts JE, O'Neal PJ, Leggo KP. 1990. Variation in the THC content of illicitly imported cannabis products—1984-1989. *Journal of Pharmacy and pharmacology* 42: 817-820.

Pitts JE, Neal JD, Gough TA. 1992. Some features of *Cannabis* plants grown in the United Kingdom from seeds of known origin. *Journal of Pharmacy and Pharmacology* 44: 947-951.

Pliny (Jones WHS, *transl.*). 1961. *Natural History*. Harvard University Press, Cambridge MA.

Pokharia AK, Sharma S, Tripathi D, Mishra N, Pal JN,Vinay R, Srivastava A. 2017. Neolithic—early historic (2500-200 BC) plant use: the archaeobotany of Ganga Plain, India. *Quaternary International* 443: 223-237.

Postans T. 1841. Memorandum on the trade between the towns of Shikarpore and Candahar. *Journal of the Asiatic Societyof Bengal* 10: 12-16.

Potter D. 2009. *The propagation, characterisation and optimisation of Cannabis sativa L. as a phytopharmaceutical.* Doctoral thesis, King's College, London.

Prain D. 1893. *Report on the cultivation and use of gánjá.* Bengal Secretariat Press, Calcutta.

Price MP, Simpson ND. 1913. An account of the plants collected by Mr. M. P. Price on the Carruthers-Miller-Price Expedition through North-West Mongolia and Chinese Dzungaria in 1910. *Journal of the Linnean Society of London, Botany* 41: 385-456.

Procter W. 1864. On a test for the resin of *Cannabis indica. Proceedings of the American Pharmaceutical Association* 12: 244-248.

Pulewka P. 1950. Über die relative Wirksamkeit türkischer Hanfpflanzen. *Naunyn-Schmiedebergs Archiv für experimentelle Pathologie und Pharmakologie* 211: 278-286.

Punja ZK, Rodriguez G, Chen S. 2017. "Assessing genetic diversity in *Cannabis sativa* using molecular approaches," pp. 395-418 in Candra S, Lata H, ElSohly MA, *eds. Cannabis sativa L. botany and biotechnology.* Springer, Cham, Switzerland.

Raven PH. 1974. Nomenclature proposals to the Leningrad Congress. Proposals for the simplification of infraspecific terminology. *Taxon* 23: 828-831.

Regel EA. 1879. Cannabineae. *Acta Horti Petropolitani* 6 (1): 476.

Ren M, Tang ZH, Wu XH, Spengler R, Jiang HG, Yang YM, Boivin N. 2019. The origins of cannabis smoking: chemical residue evidence from the first millennium BCE in the Pamirs. *Science Advances* 5: eaaw1391.

Rigter S, Niesink R. 2018. Analyse van het aanbod van hasjsoorten en wietvariëteiten in de Nederlandse coffeeshop. Netherlands Institute of Mental Health and Addiction, Utrecht.

Rizvi J. 1999. *Trans-Himalayan Caravans.* Oxford University Press, Oxford, UK.

Roborovsky VI. 1890. Progress of the Russian Expedition to Central Asia under Colonel Pievtsoff. *Proceedings of the Royal Geographical Society and Monthly Record of Geography* 12: 19-36.

Rosen BR. 1896. Двадцатипятилетие "Туркестанскихъ Ведомостей." Записки Восточнаго отдѣленія Императорскаго русскаго археологическаго общества 9: 243-262.

Rosenthal F. 1971. *The herb—hashish versus medieval Muslim society.* Brill, Leiden.

Rotherham D, Harbison SA. 2011. Differentiation of drug and non-drug *Cannabis* using a single nucleotide polymorphism (SNP) assay. Forensic Science International 207: 193-197.

Rowan MG, Fairbairn JW. 1977. Cannabinoid patterns in seedlings of *Cannabis sativa* L. and their use in the determination of chemical race. *Journal of Pharmacy and Pharmacology* 29: 491-494.

Roxburgh DJ. 2005.*The Persian album, 1400-1600: from dispersal to collection.* Yale University Press, New Haven CT.

Roxburgh W. 1804. Communication on the culture, properties, and comparative strength of hemp, and other vegetable fibres, the growth of the East Indies, "Papers in colonies and trade," *Transactions of the Society, Instituted at London, for the Encouragement of Arts, Manufactures, and Commerce* 22: 363-396.

Roxburgh W. 1815. *Observations of the late Dr. William Roxburgh, Botanical Superintendent of the Honourable East-India Company's Garden at Calcutta, on the various specimens of fibrous vegetables, the produce of India*. J. Darling, London.

Roxburgh W (Carey W, *ed.*). 1832. *Flora Indica; or, Descriptions of Indian plants*, *Volume 3.* W. Thackery, Serampore and Calcutta.

Royle JF. 1839. *Illustrations of the botany and other branches of the natural history of the Himalayan Mountains, and of the flora of Cashmere, Vol. I.* W. H. Allen, London.

Royle JF (Carson J, *ed*). 1847. *A manual of materia medica and therapeutics*. John Churchill, London.

Rubin VD, Comitas L. 1975. *Ganja in Jamaica*. Mouton Publishers, The Hague.

Rosenthal F. 1971. *The herb—hashish versus medieval Muslim society.* Brill, Leiden.

Russo EB, Jiang HE, Li X, Sutton A, Carboni A, *et al.* (13 additional authors). 2008. Phytochemical and genetic analyses of ancient cannabis from Central Asia. *Journal of Experimental Botany* 59: 4171-4182.

Sābūr ibn Sahl (Kahl O, *trans*). 2003. *The small dispensatory.* Koninklijike Brill, Leiden.

Sakamoto K, Shimomura K, Komeda Y, Kamada H, Satoh S. 1995. A male-associated DNA sequence in a dioecious plant, *Cannabis sativa* L. *Plant and Cell Physiology* 36: 1549-1554.

Salentijn EMJ, Zhang Q, Amaducci S, Yang M, Trindade LM. 2015. New developments in fiber hemp (*Cannabis sativa* L.) breeding. *Industrial Crops and Products* 68: 32–41.

Samuels D. 2008. Dr. Kush—how medical marijuana is transforming the pot industry. *The New Yorker* July 28 p 49-61

Saraswat KS. 2004a. "Plant economy of early farming communities at Senuwar, Bihar," pp. 416-535 in: Singh BP, ed. *Early Farming Communities of the Kaimur, Vol. 2.* Publication Scheme, Jaipur.

Saraswat KS, Pokharia AK. 2003. Palaeoethnobotanical investigations at Early Harappan Kunal. *Pragdhara* 13:105–139.

Saraswat KS. 2004a. "Plant economy of early farming communities at Senuwar, Bihar," pp. 416-535 in: Singh BP, ed. *Early Farming Communities of the Kaimur, Vol. 2.* Publication Scheme, Jaipur.

Saraswat KS. 2004b. Agricultural background of the early farming communities in the Middle Ganga Plain. *Prāgdharā* 15: 145-177.

Sarsenbaev KN, Kozshamzhavora LS, Yessimititova Z, Seitbayev KZH. 2013. Polymorphisms of DNA and accumulation of cannabinoids by the cultivated and wild hemp in Chu Valley. *World Applied Sciences Journal* 26: 744-769.

Sarsenbaev KN, Kozhamzharova LS, Sultanaliyeva AA, Kozhamzharova BS. 2017. Polymorphism of DNA and accumulation of cannabinoids by the cultivated and wild hemp in Chu Valley. научной конференции в рамках I Международного конгресса 1: 255-262.

Sawler J, Stout JM, Gardner KM, Hudson D, Vidmar J, Butler L, Page JE, Myles S. 2015. The genetic structure of marijuana and hemp. *PLoS One* 10(8): e0133292.

Schenk HJ, Jackson RB. 2002. The global biogeography of roots. *Ecological Monographs* 72: 311-328.

Schoenmakers N. 1986. *The Seed Bank 1986/1987 Catalogue.* Drukkerij Dukenburg Printers, Nijmegen, The Netherlands.

Schultes RE, Klein WM, Plowman T, Lockwood TE. 1974. *Cannabis*: an example of taxonomic neglect. *Harvard University Botanical Museum Leaflets* 23: 337-367.

Schwabe AL, McGlaughlin ME. 2018. Genetic tools weed out misconceptions of strain reliability in *Cannabis sativa*: implicaitons for a budding industry. *BioRxiv Preprint* https://www.biorxiv.org/content/10.1101/332320v1

Seedfinder. 2019. *Seedfinder online database.* Available at: https://en.seedfinder.eu/

Seidel U. 1989. Studien zum Vokabular der Landwirtschaft im Syrischen II. *Altorientalische Forschungen* 16: 89-139.

Semenov PP (Thomas C, *trans*). 1998. *Travels in the Tian'-Shan', 1856–1857*. The Hakluyt Society, London.

Serebriakova TY, Sizov IA. 1940. "Cannabinaceae," pp. 1-53 in: Vavilov NI, Vul'f EV, *eds.,* Культурная Флора СССР, Том V (*Cultural Flora of the USSR, Vol. 5*). State Publishing House of Collective-farm and State-farm Literture, Moscow-Leningrad.

Seshata. 2014a. *Cannabis in Kazakhstan.* Sensi Seeds website. Available at: https://sensiseeds.com/en/blog/cannabis-kazakhstan/.

Seshata. 2014b. Cannabis in Uzbekistan. Sensi Seeds website. Available at: https://sensiseeds.com/en/blog/cannabis-uzbekistan/

Sexton M, Shelton K, Haley P, West M. 2018. Evaluation of cannabinoid and terpenoid content: *Cannabis* flower compared to supercritical $CO_2$ concentrate. *Planta Medica* 84: 234-241.

Shaikhislamova EF, Suyundukov YT, Mirkin BM. 2006. Statistical analysis of factors determining the composition of segetal communities in the Bashkir Transural Region. *Russian Journal of Ecology* 37: 284–287.

Sharma GK. 1975. Altitudinal variation in leaf epidermal patterns of *Cannabis sativa. Bulletin of the Torrey Botanical Club* 102: 199-200.

Sharma GK. 1979a. Significance of eco-chemical studies of cannabis. *Science and Culture Calcutta* 45(8): 303-307.

Sharma GK. 1979b. A botanical survey of *Cannabis* in the Himalayas. *Journal of the Bombay Natural History Society* 76: 17-20.
Sharma PV. 1970. The date of *Dhanvantarī Nighaṇṭu.* Indian Journal of History of Science 5(2): 364-370.
Shirota O, Watanabe A, Yamazaki M, Saito K, Shibano K, Sekita S, Satake M. 1998. Random amplified polymorphic DNA and restriction fragment length polymorphism analyses of *Cannabis sativa. Natural Medicines* 52(2): 160-166.
Siebert A, Voss A, *ed.* 1896. *Vilmorin's Blumengärtnerei, 3rd editon, Vol 1.* Paul Parey, Berlin.
Sikora V, Berenji J, Latkovic D. 2011. Influence of agroclimatic conditions on content of main cannabinoids in industrial hemp (*Cannabis sativa* L.). *Genetika* 43: 449-456.
Simonsen JL, Todd AR. 1942. *Cannabis indica*, Part X. The essential oil from Egyptian hashish. *Journal of the Chemical Society (London)* 1942(1): 188-191.
Singh RN, Petrie CA, Joglekar PP, Neogi S, Lancelotti C, Pandey AK, Pathak A. 2013. Recent excavations at Alamgirpur, Meerut District: a preliminary report. *Man and Environment* 38: 32-54.
Small E. 1972. Interfertility and chromosomal uniformity in *Cannabis*. *Canadian Journal of Botany* 50: 1947-1949.
Small E. 1975. Morphological variation of achenes of *Cannabis*. *Canadian Journal of Botany* 53: 978-987.
Small E. 1984. "Hybridization in the domesticated-weed-wild complex," pp. 195-210 in Grant WF, *ed. Plant biosystematics.* Academic Press, NY.
Small E. 2007. "Cannabis as a source of medicinals, nutraceuticals, and functional foods.," pp. 1-39 in Acharya SN, Thomas Je, eds. *Advances in Medicinal Plant Research*. Research Signpost, Trivandrum, Kerala, India.
Small E. 2011. *Alfalfa and relatives. Evolution and classification of Medicago.* CABI Press, Wallingford UK.
Small E. 2017. *Cannabis: a complete guide.* CRC Press, Boca Raton, FL.
Small E, Beckstead HD. 1973. Common cannabinoid phenotypes in 350 stocks of *Cannabis*. *Lloydia* 36: 144-16.
Small E, Cronquist A. 1976. A practical and natural taxonomy for *Cannabis*. *Taxon* 25(4): 405-435.
Small E, Marcus D. 2003. Tetrahydrocannabinol levels in hemp (*Cannabis sativa* L.) germplasm resources. *Economic Botany* 57: 545–558.
Small E, Naraine SGU. 2015a. Expansion of female sex organs in response to prolonged virginity in *Cannabis sativa* (marijuana). *Genetic Resources and Crop Evolution* 63: 339-348.
Small E, Naraine SGU. 2015b. Size matters: evolution of large drug-secreting resin glands in elite pharmaceutical strains of *Cannabis sativa* (marijuana). *Genetic Resources and Crop Evolution* 63: 349-359.
Small E, Jui P, Lefkovitch LP. 1976. A numerical taxonomic analysis of *Cannabis* with special reference to species delimitation. *Systematic Botany* 1: 67-84.
Small E, Pocock T, Cavers PB. 2003. The biology of Canadian weeds. 119. *Cannabis sativa* L. *Canadian Journal of Plant Science* 83: 217-237.
Soornie A, Fatahi R, Haak DC, Salami SA, Bombarely A. 2017. Assessment of genetic diversity and population structure in Iranian *Cannabis* germplasm. *Scientific Reports* 7: 15668.
Spengler RN. 2013. *Botanical resource use in the Bronze and Iron Age of the Central Eurasian mountain/steppe interface: decision making in multiresource pastoral economies*. PhD thesis, Washington University in St. Louis. St. Louis, MO.
Spix JB von, Martius CFP von. 1828. *Reise in Brasilien, Zweiter Theil.* Lindauer, Münich.
Spongberg SA. 1979. Notes from the Arnold arboretum: the collections policy of the Arnold arboretum: taxa of infraspecific rank, and cultivars. *Arnoldia* 39: 370-376.
Stafleu FA, Cowan RS. 1979. *Taxonomic literature, Vol. II, H–Le.* Bohn, Scheltema & Holkema, The Hague.

Staley J. 1966. *Economy and society in Dardistan. Traditional systems and the impact of changes.* Thesis submitted for PhD, University of the Panjab, Lahore.

Steblin-Kamensky IM 1982. *Очерки по истории лексики памирских языков. Названия культурных растений* [*Studies on the lexical history of the Pamir languages. Names of cultivated plants*]. Akademiya Nauk SSSR, Moscow.

Steinberg S, Offermeier J, Field BI, Jansen van Ryssen FW. 1975. Investigation of the influence of soil types, environmental conditions, age and morphological plant parts on the chemical composition of *Cannabis sativa* (dagga) plants. *South African Medical Journal* 49: 279.

Stern WT. 1983. *Botanical Latin, 3rd ed.* David & Chalres, London.

Stevens RW. 1878. *On the stowage of ships and their cargoes, 7th edition.* Longmans, Green, Reader & Dyer, London.

Strachey H. 1848. Narrative of a journey to Cho Lagan (Rákas Tal), Cho Mapan (Mánasarówar), and the valley of Pruang in Gnari, Húndés. *Journal of the Asiatic Society of Bengal* 17(2): 98-120.

Stuessy TF. 2009. *Plant taxonomy; the systematic evaluation of comparative data, 2nd edition.* Columbia University Press, NY.

Subtelny ME. 1994. "The symbiosis of Turk and Tajik," pp. 49-52 in: Manz BF, *ed. Central Asia in historical perspective.* Westview Books, Boulder, CO.

Sun BN, Wu JY, Liu YS, Ding ST, Li XC, Xie SP, Yan DF, Lin ZC. 2011. Reconstructing Neogene vegetation and climates to infer tectonic uplift in western Yunnan, China. *Palaeogeogr. Palaeoclimatol. Palaeoecol.* 304: 328-336.

Sun JM, Ni XJ, Bi SD, Wu WY, Ye J, Meng J, Windley BF. 2014. Synchronous turnover of flora, fauna, and climate at the Eocene-Oligocene boundary in Asia. *Scientific Reports* 4:7463.

Sun WL, Zheng XY, Zhao YB, Zeng LH, Gao LS, Xheng H, Liu Y. 2017. Determination of three kinds of cannabinoids in *Cannabis* using ultra-high performance liquid chromatography–tandem mass spectrometry and analysis of phenotype of *Cannabis*. *Chinese Journal of Analytical Chemistry* 45: 1052-1058.

Swetenham E. 1841. Memorandum on the cultivation and manufacture of hemp, etc., as practiced in the hills in the north of India. *Transactions of the Agricultural & Horticultural Society of India* 8: 74-75.

Szemerényi O. 1980. *Four Old Iranian ethnic names: Scythian - Skudra - Sogdian – Saka.* Verlag der Österreichischen Akademie der Wissenschaften, Vienna.

Takhtajan AL (Crovello TJ *transl*, Cronquist A, *ed*). 1986. *Floristic regions of the world*. University of California Press, Berkeley.

Tang ZC, Chen X, Zhang QY, Guo HY, Yang M. 2013. 野生大麻种质资源表型及其 RAPD 遗传多样性分析. *Journal of West China Forestery Science* 2013(3): 61-66.

Tatarinov A. 1856. *Catalogus Medicamentorum Sinensium*. Pechatano, St. Petersburg.

Tavernier JB. 1676. *Les six voyages de Jean-Baptiste Tavernier.* G. Clouzier et C. Barbin, Paris.

Taylor BJ, Neal GD, Gough TA. 1983. The physical and chemical features of *Cannabis* plants grown in the United Kingdom of Gret Britain and Northern Ireland from seeds of known origin—Part II: second generation studies. *Bulletin on Narcotics* 35(1): 51-62.

Tejkalová K. 2015. *Cannabis sativa—objasnění vztahu mezi domnělými poddruhy*. Master's thesis, Faculty of Natural Science, Masaryk University, Brno, Czech Republic

Thomson T. 1852. *Western Himalaya and Tibet: a narrative of a journey through the mountains.* Reeve & Co., London.

Thornton E. 1858. *A gazetteer of the territories under the Government of the East-India Company.* W.H. Allen & Co., London.

Tiourebaev KS, Semenchenko GV, Dolgovskaya M, McCarthy MK, Anderson TW, Carsten LD, Pilgeram AL, Sands DC. 2001. Biological control of infestations of ditchweed (*Cannabis sativa*) with *Fusarium oxysporum* f.sp. *cannabis* in Kazakhstan. *Biocontrol Science and Technology* 11: 535-540.

Traill GW. 1828. Statistical sketch of Kamaon. *Asiatick Researches* 16: 137-236.

Turland NJ, chair. 2018. *International Code of Nomenclature for Algae, Fungi, and Plants (Shenzhen Code).* Koeltz Scientific Books, Königstein, Germany.
Turner CE. 1974. Active substances in marijuana. *Archivos de Investigacion Médica* 5: 135–140.
Turner CE, Hadley KW. 1973. Constituents of *Cannabis sativa* L. III. Clear and discrete separation of cannabidiol and cannabichromene. *Journal of Pharmaceutical Sciences* 62: 1083–1086.
Turner CE, Hadley KW, Fetterman PS, Doorenbos NJ, Quimby MW, Waller C. 1973a. Constituents of *Cannabis sativa* L. IV: Stability of cannabinoids in stored plant material. *Journal of Pharmaceutical Sciences* 62: 1601-1605.
Turner CE, Hadley K, Fetterman PS. 1973b. Constituents of *Cannabis sativa* L. VI. Propyl homologs in samples of known geographical origin. *Journal of Pharmaceutical Sciences* 62: 1739-1741.
Turner CE, Hadley KW, Henry J, Mole ML. 1974. Constituents of Cannabis sativa L. VII: use of silyl derivatives in routine analysis. *Journal of Pharmaceutical Sciences* 63: 1872-1876.
Turner CE, Cheng PC, Lewis GS, Russell MH, Sharma GK. 1979. Constituents of *Cannabis sativa.* XV: botanical and chemical profile of Indian variants. *Planta Medica* 37: 217-225.
Turner CE, ElSohly MA, Boeren EG. 1980. Constituents of *Cannabis sativa* L. XVII. A review of the natural constituents. *Journal of Natural Products* 43: 169-234.
Turner CE, ElSohly HN, Lewis GS, Lopez-Santibanez I, Carranza J. 1982. Constituents of *Cannabis sativa* L., XX: the cannabinoid content of Mexican variants grown in Mexico and in Mississippi, United States of America. *Bulletin on Narcotics* 34(1): 45-59.
United States Pharmacopeial Convention. 1916. *The Pharmacopœia of the United States of America, 9th decennial revision.* P. Blakiston's Son & Co., Philadelphia.
UN-ODC. 2008. *Illicit drug trends in Central Asia.* United Nations Office on Drugs and Crime, Regional Office for Central Asia, Tashkent, Uzbekistan.
UN-ODC. 2010. *Afghanistan Cannabis survey 2009*. United Nations Office on Drugs and Crime. Vienna, Austria.
USDA (U.S. Department of Agriculture). 1912. *Seeds and plants imported during the period from July 1 to September 30, 1911: Inventory No. 28.* Bureau of Plant Industry—Bulletin No. 248. Government Printing Office, Washington, DC.
Valente L. 1880. Sull'essenza di canapa. *Gazzetta Chimica Italiana* 10: 479-481.
Valente L. 1881. Sull'idrocarburo estratto dalla canapa. *Gazzetta Chimica Italiana* 11: 196-198.
Valikhanov C (Michell J, Michell R, *trans*). 1865. *The Russians in Central Asia*. Edward Stanford, London.
Vámbéry Á. 1875. Kulja. *The Geographical Magazine* 2(1): 175-176.
Vámbéry Á. 1868. *Sketches of Central Asia.* Allen & Co., London.
Van Bakel H, Stout JM, Cote AG, Tallon CM, Sharpe AG, Hughes TR, Page JE. 2011. The draft genome and transcriptome of *Cannabis sativa. Genome Biology* 12(10): R102.
Van der Merwe NJ, Blank LB. 1975. "Cannabis smoking in 13th-14th century Ethiopia: chemical evidence," pp. 77-80 in: Rubin V, ed. *Cannabis and Culture.* Mouton, The Hague.
Vanhoenacker G, Van Rompaey P, De Keukeleire D, Sandra P. 2002. Chemotaxonomic features associated with flavonoids of cannabinoid-free cannabis (*Cannabis sativa* subsp. *sativa* L.) in relation to hops (*Humulus lupulus* L.). *Natural Product Letters* 16(1): 57-63.
Van Valen L. 1976. Ecological species, multispecies, and oaks. *Taxon* 25: 233-239.
Vavilov NI. 1926. происхождение культурной конопли и воэникновение культуры группы «первичных» растений. *Труды по прикладной ботанике, генетике и селекции* 16(2): 107-121. English translation: 1926. The origin of the cultivation of "primary" crops, in particular cultivated hemp. *Bulletin of Applied Botany and Plant-Breeding* 16(2): 221-233.
Vavilov NI. 1931. The role of Central Asia in the origin of cultivated plants. Труды по прикладной ботанике, генетике и селекции 26(3): 1-44. Reprinted in Vavilov (1992), pp. 184-206.
Vavilov NI. 1940. "The new systematics of cultivated plants," pp. 549-566 in Huxley J, *ed. The new systematics.* Oxford University Press, Oxford UK.

Vavilov NI (Dorofeyev VF, *ed.,* Löve D, *transl.*). 1992. *Origin and Geography of Cultivated Plants.* Cambridge University Press, Cambridge UK.

Vavilov NI (Rodin LE, *ed.,* Löve D, *transl.*). 1997. *Five Continents.* International Plant Genetic Resources Institute, Rome.

Vavilov NI, Bukinich DD. 1929. Konopli. Труды по прикладной ботанике, генетике и селекции 33 (Suppl.): 380-382. Supplement published separately as: Земледельчиский Афганистан (*Agricultural Afghanistan*). Izdanija Vsesojuznyj Instituta Prikladnoj Botaniki i Novych Kul'tur pri SNK. SSSR. i Gosudarstvennogo Instituta Opytnoj Agronomii NKZ, Leningrad.

Veszki P, Verzár-Petri G, Mészáros S. 1980. Comparative phytochemical study on the cannabinoid composition of the geographical varieties of *Cannabis sativa* L. under the same conditions. *Herba Hungarica* 19: 95-103.

Vilmorin L. 1851. Chanvre de China: Tsing-Ma, *Cannabis gigantea. Revue Horticole* (Series 3) 5: 109-111.

Vilmorin H. 1892. *Les plantes de grande culture. Céréales, plantes fourragéres, industrielles et économiques.* Vilmorin-Andrieux & Co., Paris.

Vollner L, Bieniek D, Korte F. 1969. Haschisch XX: Cannabidivarin, ein neurer Haschisch-inhaltsstoff. *Tetrahedron Letters* 10(3): 145-147.

Von Middendorf AT. 1882. Очерки Ферганской долины [*Essays on the Fergana Valley*]. Tipografiya Imperial akademii nauk, St. Petersberg.

Yunusbaev UB, Musina LB, Suyundukov YT. 2003. Dynamics of steppe vegetation under the effect of grazing by different farm animals. *Russian Journal of Ecology* 34: 43-47.

Waddell L. 1905. *Lhasa and its mysteries: with a record of the Expedition of 1903-1904.* John Murray, London.

Waite E. 2006. From Holy Man to National Villain: popular historical narratives about Apaq Khoja amongst Uyghurs in contemporary Xinjiang. *Inner Asia* 8: 5-28.

Wang WM. 1996. On the origin and development of steppe vegetation in China. *Palaeobotanist* 45: 447–456.

Watson JF. 1862. *International exhibition, 1862. India. Classified and descriptive catalogue, Part II.* Spottiswoode & Co., London.

Wehmer CFW. 1911. *Die Pflanzenstoffe. Band 1, Phanerogamen.* G. Fischer, Jena.

Weiblen GD, Wenger JP, Craft KJ, ElSohly MA, Mehmedic Z, Treiber EL, Marks MD. 2015. Gene duplication and divergence affecting drug content in *Cannabis sativa*. *New Phytologist* 208: 1241-1250.

Welling MT, Liu L, Shapter T, Raymond CA, King GJ. 2016. Characterisation of cannabinoid composition in a diverse *Cannabis sativa* L. germplasm collection. *Euphytica* 208: 463–475.

Whistler H, Kinnear NB. 1949. *Popular handbook of Indian birds.* Gurney and Jackson, London.

Wiechowski W. 1927. Haschisch. *Naunyn-Schmiedebergs Archiv für experimentelle Pathologie und Pharmakologie* 119: 49-50.

Willdenow CL. 1805. *Caroli Linné Species Plantarum* IV (2): 768-769. G. C. Nauk, Berlin.

Wilson MG, Korovin VA. 2003. Oriental turtle dove breeding in the Western Palearctic. *British Birds* 96: 234-241.

Winterschmidt JS. 1818. *Naturgetreue Darstellung aller inn- und ausländischen Material-Samen und getrockneten früchte.* Herausgeber, Nürnberg.

Witzel M. 2006. “Indocentrism: autochthonous vision of ancient India,” pp. 341-404 in: Bryand EF, Patton LL. *The Indo-Aryan Controversy.* Routledge, London.

Wood TB, Spivey WTN, Easterfield TH. 1896. Charas, the resin of Indian hemp. *Journal of the Chemical Society* 6: 539-546.

Yamauchi T, Shoyama Y, Aramaki H, Azuma T, Nishioka I. 1967. Tetrahydrocannabinolic acid a genuine substance of tetrahydrocannabinol. *Chemical & Pharmaceutical Bulletin* 15: 1075-1076.

Yang Y. 2014. *The analysis of charred plant seeds at Jinchankou site and Lijiaping site during Qijia Culture period in the Hehuang region, China*. Master thesis, Dept. Physical Geography, Lanzhou University.

Yao QJ, Xiong YN, Peng F, He SL, Xia B. 2007. 不同生态类型大麻品种在南京引种的生育表(Growth and developmental character of different ecotypic hemp varieties naturalized in Nanjing). *Plant Fiber Sciences in China* 29: 270–275.

Yarmolenko AV. 1936. "Род 375. Конопля—*Cannabis* L.," pp. 383-384 in Komarov VL, *ed.* Флора СССР, Том. V (*Flora USSR, Vol. 5*). USSR Academy of Sciences, Leningrad.

Yotoriyama M, Ito I, Takashima D, Shoyama Y, Nishioka I. 1980. Plant breeding of *Cannabis*. Determination of cannabinoids by high-pressure liquid chromatography. *Yakagaku Zasshi* 100: 611-614.

Yu XL, *ed.* 2013. 西藏麻类作物种质资源 (Tibetan hemp crop germplasm resources). Agricultural and Pastoral College of Tibet Autonomous Region. Available at: http://www.taaas.org/images/14/03/21/3n2vv5y9j0/511BAB19B5BF869CBB7C31FBFA7CBBA4.pdf

Zerega NJ, Clement WL, Datwyler SL, Weiblen GD. 2005. Biogeography and divergence times in the mulberry family (Moraceae). *Molecular Phylogenetics & Evolution* 37: 402-416.

Zhang C. 2013. *Archaeobotanical remains from Lajia Site, Minhe, Qinghai*. Master thesis, Northwest University.

Zhang LG. 2009. 27 种大麻资源的 RAPD 聚类分析. Heilongjiang Agricultural Sciences 2009(2): 14-16.

Zhang LG, Chang Y, Zhang XF, Guan FZ, Yuan HM, Yu Y, Zhao LJ. 2014. Analysis of the genetic diversity of Chinese native *Cannabis sativa* cultivars by using ISSR and chromosome markers. *Genetics and Molecular Research* 13: 10490-10500.

Zhang FY, He PG. 1992. GC 和 GC/MS 对新疆不同产地大麻成分的分析研究(Analysis of cannabis components from different producing areas in Xinjiang by GC and GC/MS). *Journal of Mass Spectrometry* 1992(3): 1-6.

Zhang Q, Liu Y, Sodmerson S. 2003. Examination of the cytoplasmic DNA in male reproductive cells to determine the potential for cytoplasmic inheritance in 295 Angiosperm species. *Plant Cell Physiology* 44: 941-951.

Zhang QY, Chen X, Gou HY, Trindade LM, Salentijn EMJ, Guo R, Guo MB, Xu YP, Yang M. 2018b. Latitudinal adaptation and genetic insights into the origins of *Cannabis sativa* L. *Frontiers in Plant Science* 9: 1876.

Zhao ZJ. 2011. "Plant remains," pp. 27-34 in *Settlement patterns in the Chifeng region.* University of Pittsburgh Press, Pittsburgh PA.

Zhou Z, Bartholomew B. 2003. "Cannabaceae," pp. 74-75 in Wu ZY, Raven PH, *eds*. *Flora of China, vol. 5.* Science Press, Beijing; Missouri Botanical Garden, St. Louis, MO.

Zhou XY, Li XQ, Zhao KL, Dodson J, Sun N, Yang Q. 2011. Early agricultural development and environmental effects in the Neolithic Longdong basin (eastern Gansu). *Chinese Science Bulletin* 56: 762-771.

Zhu YH, Li YQ, Bao XH. 1992. 大麻中活性成分的气相色谱分析法及国内主要产区大麻活性成分含量的普查 (GC determination of active cannabinoids in *Cannabis* of Chinese origin and survey of their contents). *Journal of Chinese Pharmaceutical Sciences* 1(2): 73-80.

Zinger NV. 1898. Beiträge zur Kenntnis der weiblichen Blüthen und Inflorescenzen bei Cannabineen. *Flora oder Allgemeine Botanische Zeitung* 85: 189-253.
